# Enantioselective Generation of Adjacent Stereocenters in a Copper‐Catalyzed Three‐Component Coupling of Imines, Allenes, and Diboranes

**DOI:** 10.1002/anie.201606710

**Published:** 2016-08-19

**Authors:** Kay Yeung, Rebecca E. Ruscoe, James Rae, Alexander P. Pulis, David J. Procter

**Affiliations:** ^1^ School of ChemistryUniversity of ManchesterOxford RdManchesterM13 9PLUK

**Keywords:** amines, asymmetric catalysis, boron, copper, multicomponent reactions

## Abstract

A highly enantio‐ and diastereoselective copper‐catalyzed three‐component coupling affords the first general synthesis of homoallylic amines bearing adjacent stereocenters from achiral starting materials. The method utilizes a commercially available NHC ligand and copper source, operates at ambient temperature, couples readily available simple imines, allenes, and diboranes, and yields high‐value homoallylic amines that exhibit versatile amino, alkenyl, and boryl units.

Enantiomerically enriched homoallylic amines are keystone building blocks, crucial to the production of many important compounds, such as pharmaceuticals, natural products, catalysts, and ligands.^1^ Traditionally, they are synthesized by the treatment of imines with allyl metal reagents or metalloids under reagent or substrate control, which provides homoallylic amines with magnificent stereoselectivity (Scheme 1 A).^2^ However, general methods for the enantioselective allylation of imines that forge adjacent stereocenters from achiral building blocks are rare, and typically use exotic imines.^3^, ^4^ This is likely due to the difficulty in forming isomerically defined allyl nucleophiles^5^ and the low reactivity^6^ and possible *E*/*Z* geometries of the imine coupling partner,^7^ all of which affect the regio‐ and stereoselectivity during the subsequent coupling event.

**Scheme 1:**
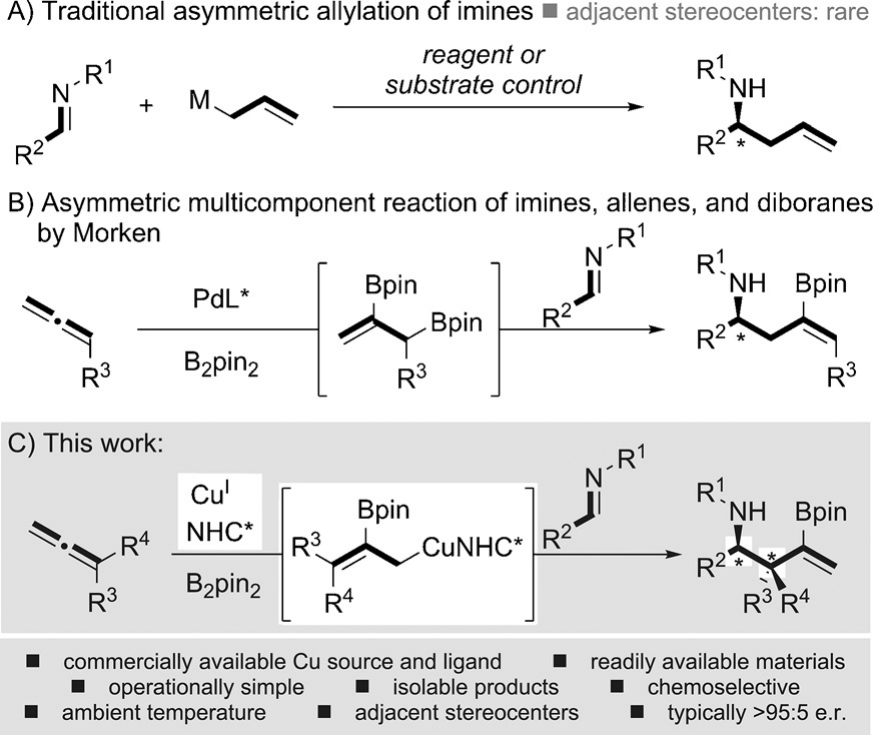
Important homoallylic amines from the enantioselective allylation of imines.

To keep apace with the demands of modern synthetic chemistry, multicomponent reactions must address challenging couplings in which numerous stereocenters are installed selectively:^8^ If complex chiral products could be assembled from several readily available achiral components in a one‐pot process, then diverse collections of high‐value compounds could be quickly constructed by simple variation of the material inputs. In recent times, enantioselective multicomponent couplings with allenes have provided efficient methods for the synthesis of highly functionalized homoallyl stereocenters.^9^ For example, the coupling of allenes and diboranes^10^ with aldehydes or ketones^11^ and allylic electrophiles^12^ has been explored.^13^, ^14^, ^15^, ^16^ However, application to the synthesis of chiral non‐racemic homoallylic amines has been surprisingly scarce,^17^, ^18^ given their importance, and this is likely due to the poor reactivity of imines.^6^, ^7^ Morken's seminal report is the only example of an enantioselective multicomponent reaction coupling allenes, imines, and B_2_pin_2_ (Scheme 1 B).^19^ The palladium‐catalyzed process utilizes a TADDOL phosphoramidite ligand, and initial asymmetric diboration13c of the allene component furnishes allylic boronic esters that are subsequently coupled with imines. The resultant highly enantiomerically enriched linear homoallylic amines were then oxidized and acylated to form isolable β‐amino ketones. However, if a process could be developed that employs a low‐cost metal and a commercially available ligand, as well as utilizing a convergent assembly of readily available achiral starting materials to generate homoallylic amines bearing adjacent stereocenters, it would be a significant advance in the synthesis of such high‐value products.

Recently, we reported preliminary findings on the copper‐catalyzed diastereoselective multicomponent coupling of imines, allenes, and diboranes for the synthesis of racemic branched homoallylic amines.17i, 20 Herein, we report an operationally simple, one‐pot, copper‐catalyzed enantioselective and diastereoselective union of imines, allenes, and diboranes, utilizing a low‐cost copper catalyst and a commercially available NHC ligand. The multicomponent coupling typically affords enantiomerically enriched homoallylic amines displaying versatile amino, vinyl, and boryl units and bearing α‐ and β‐amino stereocenters with selectivities of >95:5 e.r. (Scheme 1 C). The general enantioselective construction of homoallylic amines bearing adjacent stereocenters from simple imines and allyl copper species is unprecedented.^3^ This is also the first example of an enantioselective process that combines borocupration with the allyl cupration of imines.^21^

We began by surveying various commercially available phosphine ligands to selectively induce asymmetry in the three‐component coupling of imine **1 a**, cyclohexylallene (**2 a**), and B_2_pin_2_ mediated by catalytic CuI. However, the reaction proceeded with poor conversion (≤30 %) and enantioselectivity (≤60:40 e.r.).^22^ We then turned our attention to chiral NHCs, which were formed in situ from the corresponding commercially available imidazolium salts (Scheme 2). The NHC ligand derived from **4** 23 failed to provide any of the desired product, whereas Kündig's NHC ligand (derived from **5**, Ar=*o*‐tolyl),^24^ afforded high diastereoselectivity (>95:5 d.r.), yield (84 %), and enantioselectivity (93:7 e.r.). Gratifyingly, upon replacing the *o*‐tolyl substituent with 1‐naphthyl (**6**),^25^ another one of Kündig's imidazolium salts, the enantioselectivity (98:2 e.r., >95:5 d.r.) was further improved. Interestingly, copper‐based asymmetric transformations with NHCs derived from **5** and **6** have not been previously reported. Furthermore, the addition reaction of the intermediary Cu−Bpin species to the imine, which might be expected based on prior literature, was not observed.^26^

**Scheme 2:**
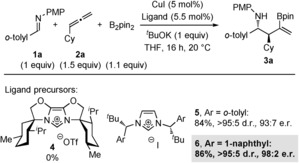
Optimization of the enantioselective copper‐catalyzed three‐component coupling of imines, allenes, and diboranes. Yields of isolated products are given. The d.r. values were determined by ^1^H NMR analysis of the crude reaction mixtures. The e.r. values were determined by HPLC analysis on a chiral stationary phase. Cy=cyclohexyl, pin=pinacolato, PMP=*para*‐methoxyphenyl, Tf=trifluoromethanesulfonyl.

With the reaction conditions established, we set about assessing the scope of the asymmetric three‐component coupling (Scheme 3–4, 5). Generally, the reaction proceeded with a variety of imines and allenes in high yield and enantioselectivity with low catalyst loading (5 mol %). We first explored the scope of the imine carbon substituent, and observed excellent tolerance with regard to steric and electronic variation (Scheme 3). Pleasingly, the use of more hindered *ortho*‐substituted imines **3 a**–**3 d** gave high yields (>70 %) and excellent enantioselectivities (98:2–97:3 e.r.) with the exception of *ortho*‐SMe (**3 e**), which was obtained in 84:16 e.r. Electron‐neutral (**3 f**), electron‐rich (**3 b**, **3 c**, **3 g**, and **3 h**), and electron‐deficient arenes (**3 i**), including those bearing functionalizable bromo substituents (**3 j**), all gave the corresponding products in high yield and exceptional enantioselectivity (≥96:4 e.r.). Heterocyclic imines, including 2‐ and 3‐furyl (**3 k** and **3 l**) and 2‐thienyl (**3 t** and **3 w**; Scheme 4), were also tolerated, but gave the corresponding products with lower yield (34–60 %) and diastereoselectivity, albeit with high enantioselectivity (≥90:10 e.r.).

**Scheme 3:**
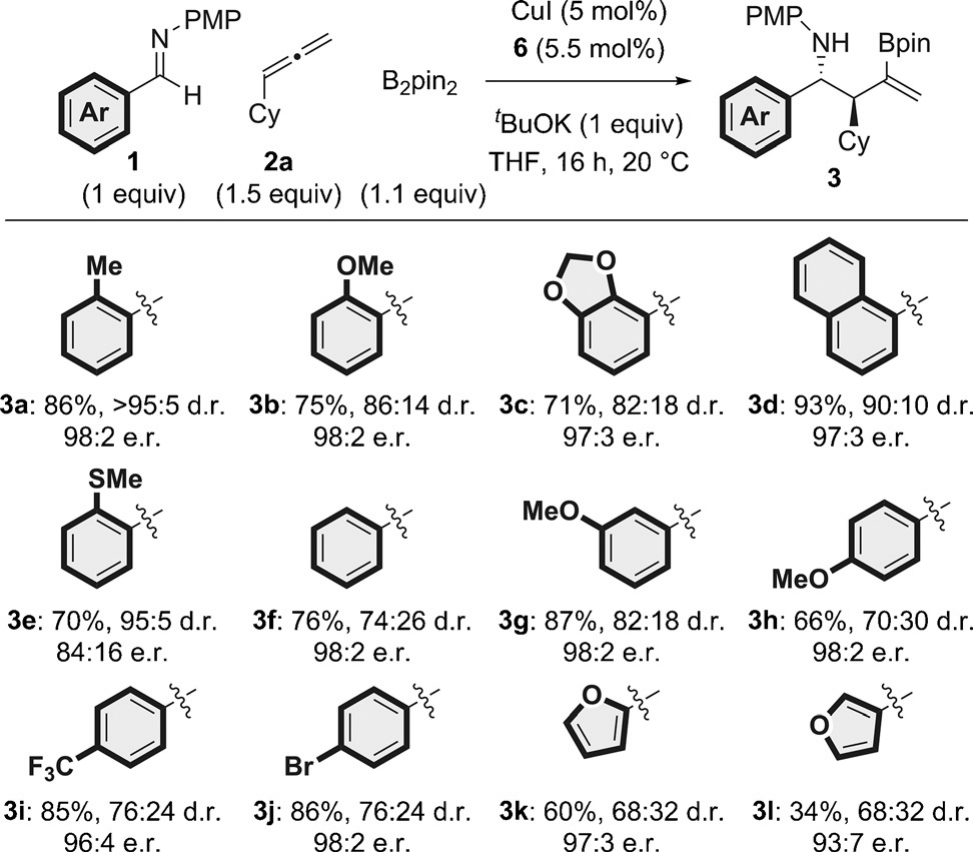
Variation of the imine carbon substituent in the enantioselective copper‐catalyzed three‐component approach to complex homoallylic amines. The d.r. values were determined by ^1^H NMR analysis of the crude reaction mixtures. The e.r. values were determined by HPLC analysis on a chiral stationary phase.

**Scheme 4:**
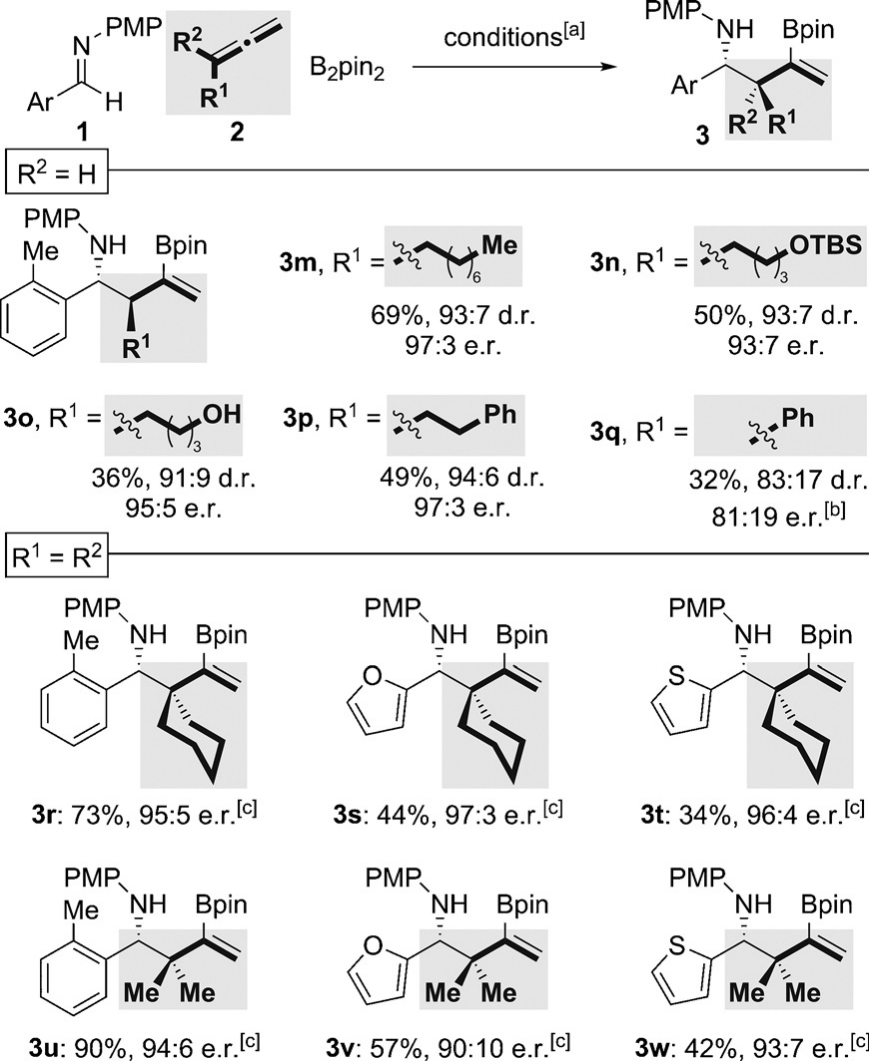
Variation of the allene in the enantioselective copper‐catalyzed three‐component coupling. The d.r. values were determined by ^1^H NMR analysis of the crude reaction mixtures. The e.r. values were determined by HPLC analysis on a chiral stationary phase. [a] See Scheme 3 for the reaction conditions. [b] Conditions as in Scheme 3, but with imidazolium salt **5** at −15 °C. [c] Conditions as in Scheme 3, but with B_2_pin_2_ (2 equiv), CuI (10 mol %), **6** (11 mol %), and ^*t*^BuOK (2 equiv). TBS=*tert*‐butyldimethylsilyl.

We next examined the allene component of the reaction (Scheme 4). 1‐Substituted allenes bearing primary and more hindered secondary alkyl groups gave the expected coupling products in universally high enantioselectivity (≥93:7 e.r.). Alkyl substituents bearing no substitution (**3 m**), a silyl ether (**3 n**), a free alcohol (**3 o**), or a phenyl group (**3 p**), were tolerated.

The use of 1‐phenylallene under the standard conditions gave the coupled product **3 q** in low yield (40 %) and in virtually racemic form (53:47 e.r., 68:32 d.r.). Use of the *o*‐tolyl ligand precursor **5**, however, gave **3 q** in 81:19 e.r. When 1,1‐disubstituted allenes were employed, poor conversions were observed under the standard conditions. Increasing the loading of Cu salt and ligand (10 and 11 mol %, respectively) restored synthetically useful yields, and highly enantioenriched homoallylic amines bearing quaternary carbon atoms in the β‐position were obtained across a range of imines (**3 r**–**3 w**, 97:3–90:10 e.r.).

We also explored various nitrogen substituents on the imine by incorporating medicinally relevant and functionalizable motifs (Scheme 5). Methyl ester (**3 x**), 5‐quinolinyl (**3 y**), morpholino (**3 z**), and (pinacolato)boryl (**3 aa**) moieties were successfully incorporated into the coupling products. To further probe the utility of the asymmetric three‐component coupling, we employed Procaine, a classic local anesthetic,^27^ in imine formation. Procaine was condensed with *o*‐tolualdehyde and submitted to the standard conditions for the enantioselective three‐component coupling. The complex homoallylic amine **3 ab** was obtained in 30 % yield (2 steps) in 94:6 d.r. and 96:4 e.r. It is interesting to note that basic nitrogen atoms, moieties that are often avoided in synthetic methodology, are well tolerated by the copper‐catalyzed process (**3 y**, **3 z**, and **3 ab**).

**Scheme 5:**
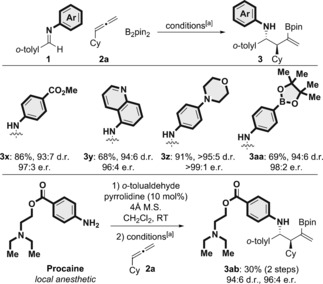
Synthetically and medicinally relevant functional groups in the imine nitrogen substituents: Enantioselective copper‐catalyzed approach to complex homoallylic amines. The d.r. values were determined by ^1^H NMR analysis of the crude reaction mixtures. The e.r. values were determined by HPLC analysis on a chiral stationary phase. [a] See Scheme 3 for the reaction conditions.

Single‐crystal X‐ray crystallography of **3 w** revealed its *R* configuration.^28^ Analysis of the X‐ray crystal structures of complexes of Kündig's *C*_2_‐symmetric ligands^24^, ^25^, ^29^ with other metals has allowed us to propose a model for the stereochemical outcome of the enantioselective three‐component coupling reaction where the flanking naphthyl rings allow approach of the imine towards the allyl copper species from one face (Scheme 6). The *anti* selectivity arises from a six‐membered‐ring chair transition state.17i

**Scheme 6:**
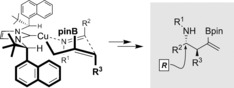
Model for the stereochemical outcome of the copper‐catalyzed enantioselective three‐component coupling of imines, allenes, and diboranes.

Finally, the scalability of the process was assessed. Using just 1.0 mol % of CuI and 1.1 mol % of ligand precursor **6**, 1 g of imine **1 a** was converted into 2 g of product **3 a**, with high levels of efficiency and selectivity (98 %, >95:5 d.r., 99:1 e.r.; Scheme 7).

**Scheme 7:**
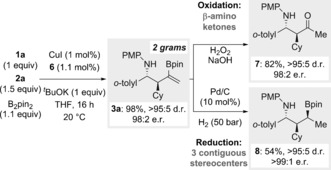
Gram‐scale enantioselective three‐component coupling, and oxidation and reduction of **3 a**.

To demonstrate the synthetic utility of products **3**, we oxidized **3 a** under standard H_2_O_2_/NaOH conditions and obtained β‐amino ketone **7**, which bears α‐ and β‐stereocenters, in high yield (82 %) and importantly without erosion of the stereochemical integrity (>95:5 d.r., 98:2 e.r.; Scheme 7). The B−N interaction present in the products of the copper‐catalyzed three‐component coupling make them particularly amenable to highly stereoselective manipulation. For example, by simply using Pd/C, **3 a** underwent a substrate‐controlled highly stereoselective hydrogenation, and gave secondary boronic ester **8**, which exhibits three contiguous stereocenters (54 %, >95:5 d.r., >99:1 e.r.).^30^

In conclusion, we have developed the first general method for the enantioselective and diastereoselective synthesis of homoallylic amines containing adjacent stereocenters from achiral starting materials, utilizing an unprecedented sequence of allene borocupration followed by allyl cupration of imines. The active allyl metal intermediate is formed in situ concurrent with C−B bond formation; therefore, prefunctionalized allyl metal derivatives are not required, and simple allenes can be used. The process exploits a low‐cost, commercially available copper(I) salt and chiral NHC catalytic system and readily available imines, allenes, and diboranes to forge molecules bearing versatile amino, alkenyl, and boryl motifs at ambient temperature. The reaction tolerates a broad range of functional groups, including basic amines. We have demonstrated the versatility of the method through a late‐stage asymmetric functionalization of Procaine, and in easily elaborating our products to β‐amino ketones and boronic esters bearing three contiguous stereocenters.

## Supporting information

As a service to our authors and readers, this journal provides supporting information supplied by the authors. Such materials are peer reviewed and may be re‐organized for online delivery, but are not copy‐edited or typeset. Technical support issues arising from supporting information (other than missing files) should be addressed to the authors.

Supplementary
