# Supplementary material for: Enantioselective Generation of Adjacent Stereocenters in a Copper‐Catalyzed Three‐Component Coupling of Imines, Allenes, and Diboranes
Source: Angew Chem Int Ed Engl. 2016 Aug 19;55(39):11912–6. doi: 10.1002/anie.201606710 (PMC5103189; doi:10.1002/anie.201606710)

Angewandte Chemie
Eine Zeitschrift der Gesellschaft Deutscher Chemiker

Supporting Information

Enantioselective Generation of Adjacent Stereocenters in a Copper-Catalyzed Three-Component Coupling of Imines, Allenes, and Diboranes

*Kay Yeung, Rebecca E. Ruscoe, James Rae, Alexander P. Pulis, and David J. Procter**

anie_201606710_sm_miscellaneous_information.pdf

Table of Contents

General Information S2

Allene and Imine Starting Materials S2

Enantioselective Copper-Catalysed Borylative Coupling of Allenes and Imines S6

Derivatisation of 3a S42

^{1}H and ^{13}C NMR spectra S45

X-ray structure of 3w S81

General Information

All experiments were performed under an atmosphere of nitrogen, using anhydrous solvents, unless stated otherwise. THF was distilled from sodium / benzophenone. Dichloromethane and trimethylamine were distilled from CaH_2. ^{1}H NMR and ^{13}C NMR spectra were recorded using 400 and 500 MHz spectrometers, with chemical shift values being reported in ppm relative to residual chloroform (δ_H = 7.27 or δ_C = 77.0) as internal standards. All coupling constants (J) are reported in Hertz (Hz). Mass spectra were obtained using positive and negative electrospray (ES±) or gas chromatography (GC) methodology. Infra-red spectra were recorded as evaporated films or neat using a FT/IR spectrometer. Column chromatography was carried out using 35 – 70 m, 60Å silica gel. Routine TLC analysis was carried out on aluminium sheets coated with silica gel 60 F254, 0.2 mm thickness and plates were viewed using a 254 mm ultraviolet lamp and dipped in aqueous potassium permanganate or *p*-anisaldehyde. Enantiomeric ratios were determined by HPLC analysis (Phenomenex® Lux 5 µm Amylose-1 (4.6 x 250 mm), Chiral Technologies Chiralpak® IA (4.6 x 250 mm), Chiralcel® OD-H (4.6 x 250 mm)) in comparison with authentic racemic materials. Specific rotations were measured on a Rudolph Research Analytical Autopol I Automatic Polarimeter. Melting points were measured on a Stuart Scientific capillary melting point apparatus and are uncorrected.

Allene and Imine Starting Materials

Reagents were either purchased directly from commercial suppliers or prepared according to literature procedures.

Allenes: propa-1,2-dien-1-ylcyclohexane (**2a**) (Aldrich), undeca-1,2-diene (**2m**)[1], *tert*-butyl(hepta-5,6-dien-1-yloxy)dimethylsilane (**2n**)[2], hepta-5,6-dien-1-ol (**2o**)[3], penta-3,4-dien-1-ylbenzene (**2p**)[4], propa-1,2-dien-1-ylbenzene (**2q**)[5], vinylidenecyclohexane (**2r**) (Aldrich), 3-methylbuta-1,2-diene (**2u**) (Aldrich).

---

[1] J. Kuang, S. Ma, *J. Org. Chem.* **2009**, *74*, 1763.
[2] J. K. Crandall, D. J. Batal, F. Lin, T. Reix, G. S. Nadal, R. A. Ng, *Tetrahedron* **1992**, *48*, 8511.
[3] V. Gobé, X. Guinchard, *Chem. Eur. J.* **2015**, *21*, 8511.
[4] S. Chanthamath, H. W. Chua, S. Kimura, K. Shibatomi, S. Iwasa, *Org. Lett.* **2014**, *16*, 3408.
[5] K. Semba, M. Shinomiya, T. Fujihara, J. Terao, Y. Tsuji, *Chem. Eur. J.* **2013**, *19*, 7125.

Imines: (*E*)-*N*-(4-methoxyphenyl)-1-(*o*-tolyl)methanimine (**1a**)[6], (*E*)-1-(2-methoxyphenyl)-*N*-(4-methoxyphenyl)methanimine (**1b**)[7], (*E*)-*N*-(4-methoxyphenyl)-1-(naphthalen-1-yl)methanimine (**1d**)[8], (*E*)-*N*-(4-methoxyphenyl)-1-phenylmethanimine (**1f**) [9], (*E*)-1-(3-methoxyphenyl)-*N*-(4-methoxyphenyl)methanimine (**1g**)[10], (*E*)-*N*,1-bis(4-methoxyphenyl)methanimine (**1h**)[1], (*E*)-*N*-(4-methoxyphenyl)-1-(4-(trifluoromethyl)phenyl)methanimine (**1i**)[1], (*E*)-1-(4-bromophenyl)-*N*-(4-methoxyphenyl)methanimine (**1j**)[11], (*E*)-1-(furan-2-yl)-*N*-(4-methoxyphenyl)methanimine (**1k**)[12], (*E*)-1-(furan-3-yl)-*N*-(4-methoxyphenyl)methanimine (**1l**)[13], (*E*)-*N*-(4-methoxyphenyl)-1-(thiophen-3-yl)methanimine (**1t**)[14], (*E*)-*N*-(quinolin-5-yl)-1-(*o*-tolyl)methanimine (**1y**)[15].

General Procedure 1 for Imine Formation

Aldehyde (3.19 mmol, 1.0 equiv.) was added to a solution of amine (3.19 mmol, 1.0 equiv.) and 4 Å molecular sieves (1 g/mmol) in anhydrous dichloromethane (10 mL) at room temperature under an atmosphere of argon. The reaction was left to stir for 18 hours and then filtered through a pad of Celite®, washed with dichloromethane and evaporated to afford the product.[16]

(*E*)-1-(Benzo[*d*][1,3]dioxol-4-yl)-*N*-(4-methoxyphenyl)methanimine (1c)

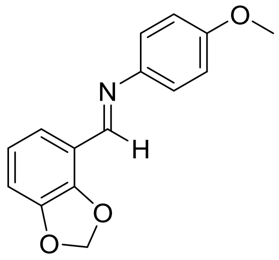

[6] K. Yamada, T. Konishi, M. Nakano, S. Fujii, R. Cadou, Y. Yamamoto, K. Tomioka, *J. Org. Chem.* **2012**, *77*, 1547.
[7] T. Yamakawa, N. Yoshikai, *Tetrahedron* **2013**, *69*, 4459.
[8] S. E. Denmark, N. Nakajima, C. M. Stiff, O. J.-C. Nicaise, M. Kranz, *Adv. Synth. Catal.* **2008**, *350*, 1023.
[9] A. M. Seayad, B. Ramalingam, K. Yoshinaga, T. Nagata, C. L. L. Chai, *Org. Lett.* **2010**, *12*, 264.
[10] G. Dagousset, J. Zhu, G. Masson, *J. Am. Chem*. Soc. **2011**, *133*, 14804.
[11] M. B. Shaghafi, R. E. Grote, E. R. Jarvo, *Org. Lett.* **2011**, *13*, 5188.
[12] I. Ojima, I. Habus, M. Zhao, M. Zucco, Y. H. Park, C. M. Sun, T. Brigaud, *Tetrahedron* **1992**, *48*, 6985.
[13] M. Sickert, C. Schneider, *Angew. Chem. Int. Ed.* **2008**, *47*, 3631.
[14] M. Patel, M. Chhasatia, P. Parmar, *Eur. J. Med. Chem.* **2010**, *45*, 439.
[15] K. Saeki, T. Matsuda, T. Kato, S. Matsui, K. Fukuhara, N. Miyata, *Biol. Pharm. Bull.* **2003**, *26*, 448; N. G. Kozlav, A. B. Tereshko, *Russ. J. Org. Chem.* **2013**, *49*, 1335.
[16] S. Morales, F. G. Guijarro, J. L. G. Ruano, M. Belén Cid, *J. Am. Chem. Soc.* **2014**, *136*, 1082.

Prepared according to General Procedure 1, on a 3.19 mmol scale, afforded the title compound as a green powder (836 mg, 2.98 mmol, 94%). Mp: 89.8-91.9 °C; MS (ES$^+$) *m/z*: 256 (M+H$^+$). HRMS calcd for $C_{15}H_{14}O_3N$: 256.0968. Found: 256.0962; ν_{max} (thin film/cm^{-1}) 2895, 1623, 1503, 1453, 1356, 1294, 1245, 1207, 1035; ^{1}H NMR (400 MHz, $CDCl_3$) δ ppm 3.84 (s, 3 H, OCH_3), 6.09 (s, 2 H, OCH_2O), 6.89 - 6.96 (m, 4 H, ArCH), 7.23 - 7.26 (m, 2 H, ArCH), 7.47 (dd, *J* = 6.27, 3.01 Hz, 1 H, ArCH), 8.60 (s, 1 H, HC=N); ^{13}C NMR (101 MHz, $CDCl_3$) δ ppm 55.5 (OCH_3), 101.7 (CH_2), 110.4 (ArCH), 114.3 (ArCH), 119.1 (ArC), 120.2 (ArCH), 121.8 (ArCH), 122.3 (ArCH), 145.1 (ArC), 147.5 (ArC), 148.2 (ArC), 153.1 (C=N), 158.4 (ArC).

(*E*)-*N*-(4-Methoxyphenyl)-1-(2-(methylthio)phenyl)methanimine (1e)

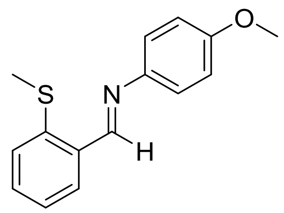

Prepared according to General Procedure 1, on a 3.19 mmol scale, afforded the title compound as a brown solid (831 mg, 3.23 mmol, quant. yield). Mp: 77.6-79.1 °C; MS (ES$^+$) *m/z*: 258 (M+H$^+$). HRMS calcd for $C_{15}H_{14}O_3N$: 258.0947. Found: 258.0942; ν_{max} (thin film/cm^{-1}) 2919, 2834, 1615, 1595, 1501, 1464, 1438, 1296, 1245, 1198, 1162, 1066, 1034; ^{1}H NMR (400 MHz, $CDCl_3$) δ ppm 2.53 (s, 3 H, SCH_3), 3.86 (s, 3 H, OCH_3), 6.94 - 6.99 (m, 2 H, ArCH), 7.27 - 7.28 (m, 1 H, ArCH), 7.29 - 7.32 (m, 2 H, ArCH), 7.37 - 7.46 (m, 2 H, ArCH), 8.09 (dd, *J* = 7.78, 1.51 Hz, 1 H, ArCH), 9.00 (s, 1 H, HC=N); ^{13}C NMR (101 MHz, $CDCl_3$) δ ppm 17.0 (SCH_3), 55.5 (OCH_3), 114.4 (ArCH), 122.4 (ArCH), 125.6 (ArCH), 127.5 (ArCH), 128.4 (ArCH), 131.0 (ArCH), 134.5 (ArC), 140.1 (ArC), 145.0 (ArC), 156.3 (C=N), 158.4 (ArC).

Methyl (*E*)-4-((2-methylbenzylidene)amino)benzoate (1x)

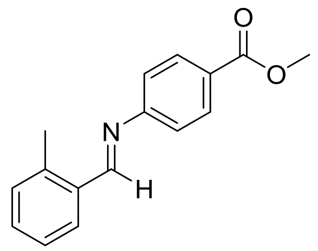

Prepared according to General Procedure 1, on a 3.19 mmol scale, afforded the title compound as a pale orange solid (816 mg, 3.22 mmol, quant. yield). Mp: 76.0-78.4 °C; MS (ES$^+$) *m/z*: 254 (M+H$^+$). HRMS calcd for $C_{16}H_{16}O_2N$: 254.1176. Found: 254.1171; ν_{max} (thin film/cm^{-1}) 3406, 2978, 2248, 1604, 1510, 1364, 1307, 1235, 1137, 1039; ^{1}H NMR (400 MHz, $CDCl_3$) δ ppm 2.62 (s, 3 H, Ar-CH_3), 3.94 (s, 3

H, CO_2CH_3), 7.17 - 7.23 (m, 2 H, ArCH), 7.18 – 7.23 (m, 1 H, ArCH), 7.30 - 7.36 (m, 1 H, ArCH), 7.37 - 7.43 (m, 1 H, ArCH), 8.05 - 8.13 (m, 3 H, ArCH), 8.73 (s, 1 H, HC=N); ^{13}C NMR (101 MHz, $CDCl_3$) δ ppm 19.5 (Ar-CH_3), 52.1 (CO_2CH_3), 120.7 (ArCH), 126.5 (ArCH), 127.3 (ArC), 128.2 (ArCH), 130.9 (ArCH), 131.2 (ArCH), 131.5 (ArCH), 133.70 (ArC), 139.0 (ArC), 156.8 (ArC), 160.4 (C=N), 166.9 (C=O).

(*E*)-*N*-(4-Morpholinophenyl)-1-(*o*-tolyl)methanimine (1z)

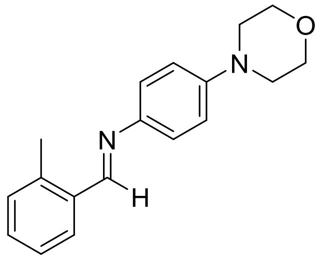

Prepared according to General Procedure 1, on a 3.19 mmol scale, afforded the title compound as a brown solid (836 mg, 2.98 mmol, 94%). Mp: 113.3-115.0 °C; MS (ES^+) *m/z*: 281 ($M+H^+$). HRMS calcd for $C_{18}H_{21}ON_2$: 281.1648. Found: 281.1642; ν_{max} (thin film/cm^{-1}) 2959, 2854, 1619, 1602, 1499, 1448, 1379, 1210, 1172, 1121, 1068, 1050; ^{1}H NMR (400 MHz, $CDCl_3$) δ ppm 2.59 (s, 3 H, Ar-CH_3), 3.17 - 3.22 (m, 4 H, CH_2), 3.89 (m, 4 H, CH_2), 6.96 (d, *J* = 8.7 Hz, 2 H, ArCH), 7.21 - 7.26 (m, 3 H, ArCH), 7.29 - 7.37 (m, 2 H, ArCH), 8.08 (d, *J* = 7.6 Hz, 1 H, ArCH), 8.79 (s, 1 H, HC=N); ^{13}C NMR (101 MHz, $CDCl_3$) δ ppm 19.4 (Ar-CH_3), 49.6 (CH_2), 66.9 (CH_2), 116.2 (ArCH), 122.1 (ArCH), 126.3 (ArCH), 127.5 (ArCH), 130.6 (ArCH), 130.9 (ArCH), 134.5 (ArC), 138.3 (ArC), 144.9 (ArC), 149.9 (ArC), 156.6 (C=N).

(*E*)-*N*-(4-(4,4,5,5-Tetramethyl-1,3,2-dioxaborolan-2-yl)phenyl)-1-(*o*-tolyl)methanimine (1aa)

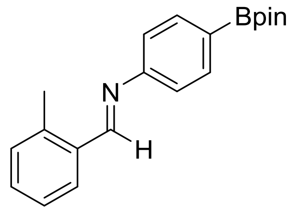

Prepared according to General Procedure 1, on a 1.60 mmol scale, afforded the title compound as a pale brown gum (544 mg, 1.69 mmol, quant. yield). MS (ES^+) *m/z* 322: ($M+H^+$). HRMS calcd for $C_{20}H_{25}BO_2N$: 322.1973. Found: 322.1966; ν_{max} (thin film/cm^{-1}) 3373, 2977, 1695, 1622, 1592, 1458, 1396, 1350, 1314, 1270, 1202, 1141, 1087, 1015; ^{1}H NMR (400 MHz, $CDCl_3$) δ ppm 1.37 (s, 12 H, 4 x CH_3), 2.60 (s, 3 H, Ar-CH_3), 7.18 (d, *J* = 8.2 Hz, 2 H, ArCH), 7.24 (d, *J* = 7.5 Hz, 1 H, ArCH), 7.39-7.33 (m, 1 H, ArCH), 7.38 (d, *J* = 1.1 Hz, 1 H, ArCH) 7.86 (d, *J* = 8.1 Hz, 2 H, ArCH), 8.08 (d, *J* = 7.0 Hz, 1 H, ArCH), 8.74 (s, 1 H, HC=N); ^{13}C NMR (101 MHz, $CDCl_3$) δ ppm 19.4 (Ar-CH_3), 24.9 (CH_3), 24.9 (CH_3), 83.3 (OC),

83.8 (OC), 114.1 (ArCH), 120.2 (ArCH), 126.4 (ArCH), 128.0 (ArCH), 131.0 (ArCH), 131.2 (ArCH), 134.0 (ArC), 135.9 (ArCH), 136.4 (ArCH), 138.8 (ArC), 155.4 (ArC), 159.6 (C=N), (ArC-Bpin not observed); ^{11}B NMR (128 MHz, $CDCl_3$) δ ppm -7.0, 30.0.

2-(Diethylamino)ethyl (*E*)-4-((2-methylbenzylidene)amino)benzoate (1ab)

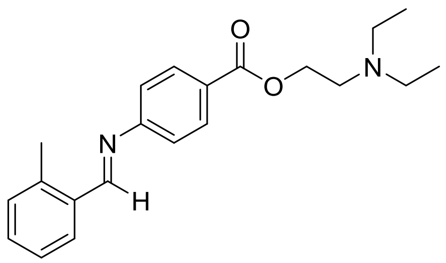

Prepared according to General Procedure 1, on a 3.19 mmol scale, afforded the title compound as an orange gum (1.06 g, 3.13 mmol, 98%). MS (ES$^+$) *m/z*: 339 (M+H$^+$). HRMS calcd for $C_{21}H_{27}O_2N_2$: 339.2067. Found: 339.2064; ν_{max} (thin film/cm^{-1}) 3371, 2968, 2815, 1699, 1602, 1456, 1270, 1203, 1169, 1113, 1014; ^{1}H NMR (400 MHz, $CDCl_3$) δ ppm 1.09 (t, *J* = 7.2 Hz, 6 H, 2 x CH_2CH_3), 2.59 - 2.70 (m, 7 H, Ar-CH_3 + 2 x CH_2CH_3), 2.88 (t, *J* = 6.3 Hz, 2 H, CH_2), 4.41 (t, *J* = 6.3 Hz, 2 H, CH_2), 7.16 - 7.23 (m, 2 H, ArCH), 7.25 (s, 1 H, ArCH), 7.29 - 7.43 (m, 2 H, ArCH), 8.05 - 8.11 (m, 3 H, ArCH), 8.73 (s, 1 H, HC=N); ^{13}C NMR (101 MHz, $CDCl_3$) δ ppm 12.2 (CH_2CH_3), 19.5 (Ar-CH_3), 47.9 (CH_2CH_3), 51.1 (CH_2), 63.5 (CH_2), 113.8 (ArCH), 120.7 (ArCH), 126.5 (ArCH), 127.4 (ArC), 128.2 (ArCH), 130.9 (ArCH), 131.2 (ArCH), 133.7 (ArC), 139.0 (ArC), 156.8 (ArC), 160.3 (C=N), 166.4 (C=O).

Enantioselective Copper-Catalysed Borylative Coupling of Allenes and Imines

Racemic standards for the copper-catalysed borylative coupling of allenes and imines were prepared according to literature procedures.[17]

Following that procedure, novel compounds were obtained:

3b: 60%, 92:8 d.r.; **3c**: 88%, 92:8 d.r.; **3e**: 34%, >95:5 d.r.; **3g**: 57%, 80:20 d.r.; **3l**: 47%, 60:40 d.r.; **3n**: 27%, 91:9 d.r.; **3o**: 69%, 93:7 d.r., **3p**: 73%, 92:8 d.r.; **3q**: 29%, 56:44 d.r.; **3r**: 89%; **3v**: 72%; **3w**: 81%; **3x**: 59%, 90:10 d.r.; **3y**: 56%, 94:6 d.r.; **3z**: 28%, >95:5 d.r., **3aa**: 41%, 69:31 d.r.; **3ab**: 16%, 94:6 d.r.

[17] J. Rae, K. Yeung, J. J. W. McDouall, D. J. Procter, *Angew. Chem. Int. Ed.* **2016**, *55*, 1102; *Angew. Chem.* **2016**, *128*, 1114.

General Procedure 2 for the enantioselective copper-catalysed borylative coupling of allenes and imines

***N*-((1*R*,2*R*)-2-Cyclohexyl-3-(4,4,5,5-tetramethyl-1,3,2-dioxaborolan-2-yl)-1-(*o*-tolyl)but-3-en-1-yl)-4-methoxyaniline (3a)**

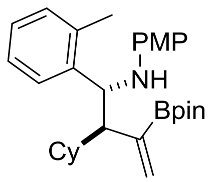

To a solution of CuI (2.5 mg, 0.013 mmol, 5 mol%) and NHC ligand **6** (8.4 mg, 0.014 mmol, 5.5 mol%) in THF (0.8 mL), was added *t*-BuOK (0.26 mL of a 1 M THF solution, 0.258 mmol, 1 equiv), and the reaction was stirred for 75 minutes at room temperature. B_2Pin_2 (72 mg, 0.284 mmol, 1.1 equiv) in THF (0.75 mL) was then added and the resulting mixture stirred for 30 minutes. A solution of propa-1,2-dien-1-ylcyclohexane (**2a**) (47 mg, 0.387 mmol, 1.5 equiv) and (*E*)-*N*-(4-methoxyphenyl)-1-(*o*-tolyl)methanimine (**1a**) (58 mg, 0.258 mmol, 1 equiv) in THF (1 mL) was then added dropwise at room temperature with stirring overnight. The mixture was then filtered through a silica plug, concentrated *in vacuo* and the crude product mixture (>95:5 d.r.) was purified by chromatography (3% EtOAc in hexanes) to afford the title compound as a white foam (106 mg, 0.223 mmol, 86%).

A pure sample of the major diastereoisomer was obtained; MS (ES^+) *m/z*: 476 ($M+H^+$). HRMS calcd for $C_{30}H_{42}NBO_3$: 476.3341. Found: 476.3357; ν_{max} (thin film/cm^{-1}): 3408, 2976, 2927, 2850, 1525, 1462, 1447, 1421, 1371, 1300, 1277, 1233, 1180, 1165, 1142, 1041; ^1H NMR (400 MHz, $CDCl_3$) δ ppm 0.71 - 1.23 (m, 5 H, CH_2), 1.31 (s, 6 H, 2 x CH_3), 1.36 (s, 6 H, 2 x CH_3), 1.55 - 1.79 (m, 4 H, CH_2), 1.88 - 2.08 (m, 2 H, CH_2 + CH), 2.20 (dd, *J* = 10.1, 4.0 Hz, 1 H, C*H*C=CH_2), 2.46 (br. s, 3 H, Ar-CH_3), 3.68 (s, 3 H, OCH_3), 4.88 (d, *J* = 3.5 Hz, 2 H, CHN + C=CH_2), 5.56 (br. s, 1 H, NH), 5.70 (d, *J* = 3.5 Hz, 1 H, C=CH_2), 6.30 (d, *J* = 8.8 Hz, 2 H, ArCH), 6.66 (d, *J* = 8.8 Hz, 2 H, ArCH), 6.94 - 7.15 (m, 4 H, ArCH); ^{13}C NMR (101 MHz, $CDCl_3$) δ ppm 19.1 (Ar-*C*H_3), 24.4 (CH_3), 25.0 (CH_3), 26.4 (CH_2), 26.5 (CH_2), 26.6 (CH_2), 31.0 (CH_2), 33.0 (CH_2), 36.9 (CH), 54.4 (CHN), 55.8 (OCH_3), 58.2 (*C*HC=CH_2), 83.7 (OC), 112.9 (ArCH), 114.8 (ArCH), 125.4 (ArCH), 125.9 (ArCH), 127.8 (ArCH), 130.1 (ArCH), 134.2 (ArC), 134.8 (C=*C*H_2), 140.9 (ArC), 142.5 (ArC), 150.8 (ArC), (B*C*=CH_2 not observed); ^{11}B NMR (128 MHz, $CDCl_3$) δ ppm 8.0; Specific rotation: $[\alpha]_D^{27}$ -54.2 (*c* 1.06, $CHCl_3$) for an enantiomerically enriched sample of 98:2 e.r.

Enantiomeric purity of **3a** was determined by HPLC analysis in comparison with authentic racemic material (98:2 e.r. shown; Lux 5 µm Amylose-1 column, 99:1 hexanes:*i*-PrOH, 0.3 mL/min, 20 °C, 254 nm).

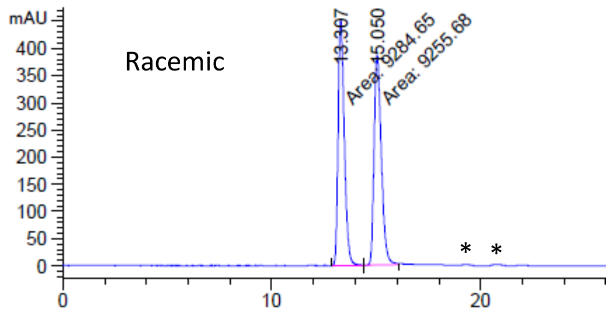

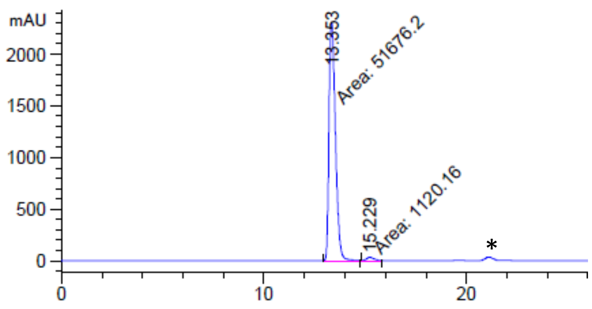

* = minor diastereoisomer

Peak #	RetTime [min]	Type	Width [min]	Area [mAU*s]	Height [mAU]	Area %
1	13.307	MF	0.3429	9284.65332	451.34103	50.0781
2	15.050	FM	0.3987	9255.67578	386.94217	49.9219

Peak #	RetTime [min]	Type	Width [min]	Area [mAU*s]	Height [mAU]	Area %
1	13.353	MM	0.3724	5.16762e4	2312.75049	97.8783
2	15.229	MM	0.5273	1120.15515	35.40219	2.1217

**Peak #**	**Time (min)**	**Area (%)**	**Peak #**	**Time (min)**	**Area (%)**
1	13.307	50.0781	1	13.353	97.8783
2	15.050	49.9219	2	15.229	2.1217

***N*-((1*R*,2*R*)-2-Cyclohexyl-1-(2-methoxyphenyl)-3-(4,4,5,5-tetramethyl-1,3,2-dioxaborolan-2-yl)but-3-en-1-yl)-4-methoxyaniline (3b)**

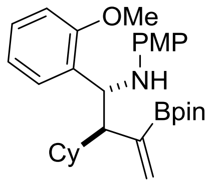

Prepared according to General Procedure 2, on a 0.258 mmol scale (86:14 d.r. of crude material), column chromatography (2-3% EtOAc in Hexanes) afforded the title compound as an orange gum (95 mg, 0.194 mmol, 75%).

A pure sample of the major diastereoisomer was obtained; MS (ES^+) *m/z*: 492 ($M+H^+$). HRMS calcd for $C_{30}H_{43}NBO_4$: 492.3280. Found: 492.3267; ν_{max} (thin film/cm^{-1}): 3408, 2976, 2924, 2849, 1601, 1512, 1487, 1464, 1438, 1421, 1371, 1300, 1277, 1233, 1142, 1109, 1089, 1031; ^{1}H NMR (400 MHz, $CDCl_3$) δ ppm 0.75 - 0.86 (m, 1 H, CH_2), 0.99 - 1.24 (m, 4 H, CH_2), 1.28 (s, 6 H, 2 x CH_3), 1.32 (s, 6 H, 2 x CH_3), 1.57 - 1.76 (m, 4 H, CH_2), 1.90 (d, *J* = 2.5 Hz, 2 H, CH_2 + CH), 2.45 (dd, *J* = 9.8, 4.5 Hz, 1 H, *CH*C=CH_2), 3.68 (s, 3 H, OCH_3), 3.90 (s, 3 H, OCH_3), 4.98 (d, *J* = 3.8 Hz, 1 H, C=CH_2), 5.09 (d, *J* = 4.5 Hz, 1 H, CHN), 5.40 (br. s, 1 H, NH), 5.68 (d, *J* = 3.8 Hz, 1 H, C=CH_2), 6.33 - 6.40 (m, 2 H, ArCH), 6.63 - 6.68 (m, 2 H, ArCH), 6.76 (td, *J* = 7.4, 1.0 Hz, 1 H, ArCH), 6.84 (dd, *J* = 8.2, 0.9 Hz, 1 H, ArCH), 7.03 (dd, *J* = 7.7, 1.6 Hz, 1 H, ArCH), 7.11 (m, 1 H, ArCH); ^{13}C NMR (101 MHz, $CDCl_3$) δ ppm 24.4 (CH_3), 24.9 (CH_3),

26.3 (CH_2), 26.4 (CH_2), 26.6 (CH_2), 31.4 (CH_2), 32.2 (CH_2), 36.9 (CH), 55.1 (CHN), 55.8 (OCH_3), 58.2 (OCH_3), 61.2 (*C*HC=CH_2), 83.6 (OC), 111.4 (ArCH), 113.0 (ArCH), 113.3 (ArCH), 114.7 (ArCH), 119.8 (ArCH), 128.8 (ArCH), 134.5 (ArC), 142.6 (C=*C*H_2), 146.0 (ArC), 150.9 (ArC), 159.4 (ArC), (B*C*=CH_2 not observed); ^{11}B NMR (128 MHz, $CDCl_3$) δ ppm 29.8; Specific rotation: $[\alpha]_D^{26}$ -31.9 (*c* 0.74, $CHCl_3$) for an enantiomerically enriched sample of 98:2 e.r.

Enantiomeric purity of **3b** was determined by HPLC analysis in comparison with authentic racemic material (98:2 e.r. shown; Lux 5 µm Amylose-1 column, 99:1 hexanes:*i*-PrOH, 0.3 mL/min, 20 °C, 254 nm).

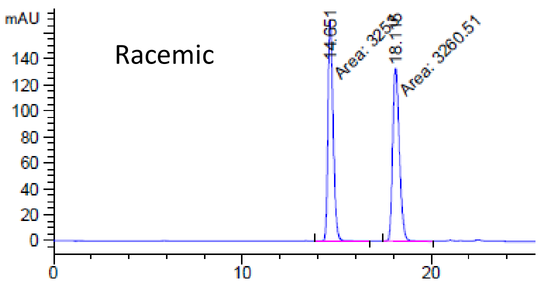

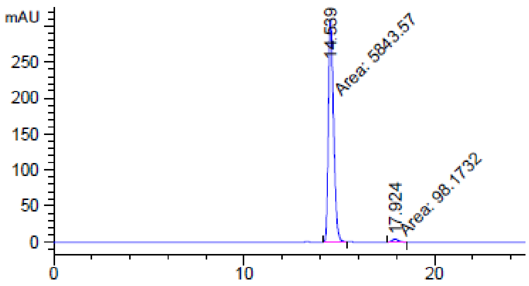

Peak #	RetTime [min]	Type	Width [min]	Area [mAU*s]	Height [mAU]	Area %
1	14.651	MM	0.3192	3252.99951	169.85100	49.9423
2	18.115	MM	0.4093	3260.51440	132.77136	50.0577

Peak #	RetTime [min]	Type	Width [min]	Area [mAU*s]	Height [mAU]	Area %
1	14.539	MM	0.3140	5843.57422	310.13324	98.3477
2	17.924	MM	0.3965	98.17323	4.12685	1.6523

**Peak #**	**Time (min)**	**Area (%)**	**Peak #**	**Time (min)**	**Area (%)**
1	14.651	49.9423	1	14.539	98.3477
2	18.115	50.0577	2	17.924	1.6523

***N*-((1*R*,2*R*)-1-(Benzo[*d*][1,3]dioxol-4-yl)-2-cyclohexyl-3-(4,4,5,5-tetramethyl-1,3,2-dioxaborolan-2-yl)but-3-en-1-yl)-4-methoxyaniline (3c)**

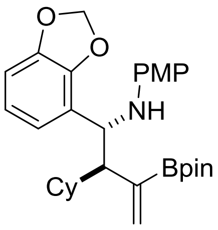

Prepared according to General Procedure 2, on a 0.258 mmol scale (82:18 d.r. from crude material), column chromatography (10% EtOAc in Hexanes) afforded the title compound as a brown gum (92 mg, 0.182 mmol, 71%).

A pure sample of the major diastereoisomer was obtained; MS (ES$^+$) *m/z*: 506 (M+H$^+$). HRMS calcd for $C_{30}H_{41}NBO_5$: 506.3072. Found: 506.3069; ν_{max} (thin film/cm^{-1}): 3406, 2977, 2925, 2650, 1511, 1455, 1371, 1302, 1246, 1141, 1053, 1040; ^{1}H NMR (400 MHz, $CDCl_3$) δ ppm 0.66 - 0.76 (m, 1 H, CH_2), 0.92 - 1.11 (m, 4 H, CH_2), 1.14 – 1.24 (2 x s, 12 H, 4 x CH_3), 1.50 - 1.64 (m, 4 H, CH_2), 1.69 - 1.80 (m, 2 H, CH_2 + CH), 2.32 (dd, *J* = 8.8, 5.5 Hz, 1 H, C*H*C=CH_2), 3.61 (s, 3 H, OCH_3), 4.78 (d, *J* = 5.5 Hz, 1 H, CHN), 5.09 - 5.16 (m, 2 H, C=CH_2 + NH), 5.68 (d, *J* = 3.8 Hz, 1 H, C=CH_2), 5.85 (apparent q, *J* = 1.5 Hz, 2 H, OCH_2O), 6.31 - 6.37 (m, 2 H, ArCH), 6.51 - 6.63 (m, 5 H, ArCH); ^{13}C NMR (101 MHz, $CDCl_3$) δ ppm 24.5 (CH_3), 25.0 (CH_3), 26.3 (CH_2), 26.5 (CH_2), 26.6 (CH_2), 31.2 (CH_2), 32.1 (CH_2), 36.9 (CH), 53.4 (CHN), 55.8 (OCH_3), 58.2 (*C*HC=CH_2), 83.6 (OC), 100.5 (OCH_2O), 106.6 (ArCH), 113.3 (2 x ArCH), 114.7 (2 x ArCH), 121.1 (2 x ArCH), 126.0 (ArC), 134.0 (C=*C*H_2), 142.4 (ArC), 144.5 (ArC), 146.9 (ArC), 151.0 (ArC), (B*C*=CH_2 not observed); ^{11}B NMR (128 MHz, $CDCl_3$) δ ppm -3.0, 31.0; Specific rotation: $[\alpha]_D^{27}$ -17.6 (*c* 1.11, $CHCl_3$) for an enantiomerically enriched sample of 97:3 e.r.

Enantiomeric purity of **3c** was determined by HPLC analysis in comparison with authentic racemic material (97:3 e.r. shown; Lux 5 µm Amylose-1 column, 99:1 hexanes:*i*-PrOH, 0.3 mL/min, 20 °C, 254 nm).

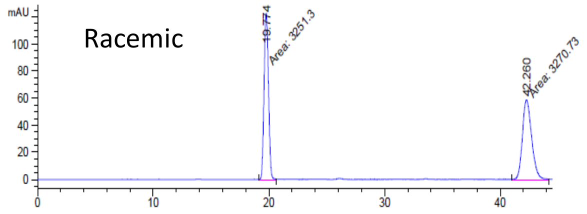

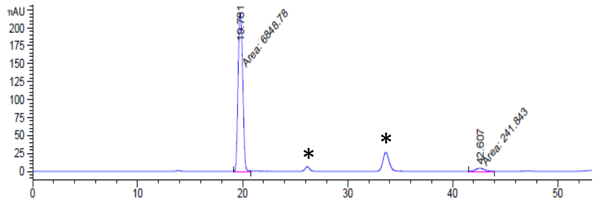

Peak #	RetTime [min]	Type	Width [min]	Area [mAU*s]	Height [mAU]	Area %
1	19.774	MM	0.4427	3251.29614	122.41525	49.8510
2	42.260	MM	0.9264	3270.73267	58.84336	50.1490

Peak #	RetTime [min]	Type	Width [min]	Area [mAU*s]	Height [mAU]	Area %
1	19.781	MM	0.5149	6848.78125	221.69031	96.5893
2	42.607	MM	0.9592	241.84343	4.20238	3.4107

Peak #	Time (min)	Area (%)	Peak #	Time (min)	Area (%)
1	19.774	49.8510	1	19.781	96.5893
2	42.260	50.1490	2	42.607	3.4107

***N*-((1*R*,2*R*)-2-Cyclohexyl-1-(naphthalen-1-yl)-3-(4,4,5,5-tetramethyl-1,3,2-dioxaborolan-2-yl)but-3-en-1-yl)-4-methoxyaniline (3d)**

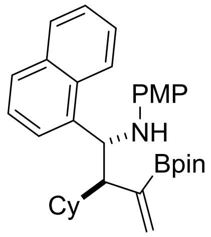

Prepared according to General Procedure 2, on a 0.258 mmol scale (90:10 d.r. of crude material), column chromatography (3% EtOAc in Hexanes) afforded the title compound as a mixture of diastereoisomers, as an orange liquid (122 mg, 0.239 mmol, 93%, 93:7 d.r.).

Data for the major diastereoisomer; MS (ES^+) *m/z*: 512 ($M+H^+$). HRMS calcd for $C_{33}H_{43}NBO_3$: 512.3336. Found: 512.3332; ν_{max} (thin film/cm^{-1}): 3408, 2976, 2926, 2850, 1598, 1511, 1422, 1371, 1302, 1234, 1173, 1142, 1040; ^{1}H NMR (500 MHz, $CDCl_3$) δ ppm 0.75 - 0.68 (m, 2 H, CH_2), 1.17 - 1.26 (m, 3 H, CH_2), 1.32 (s, 6 H, 2 x CH_3), 1.37 (s, 6 H, 2 x CH_3), 1.61 (d, *J* = 13.6 Hz, 1 H, CH_2), 1.69 (t, *J* = 9.3 Hz, 2 H, CH_2), 1.81 (d, *J* = 11.7 Hz, 1 H, CH_2), 2.07 (m, 1 H, CH), 2.23 (d, *J* = 12.0 Hz, 1 H, CH_2), 2.53 (dd, *J* = 10.6, 3.9 Hz, 1 H, C*H*C=CH_2), 3.65 (s, 3 H, OCH_3), 4.53 (d, *J* = 3.2 Hz, 1 H, C=CH_2), 5.52 (d, *J* = 3.2 Hz, 1 H, C=CH_2), 5.55 (dd, *J* = 3.8 Hz, 1 H, CHN), 5.77 (br. s, 1 H, NH), 6.34 (d, *J* = 9.1 Hz, 2 H, ArCH), 6.62 (d, *J* = 8.8 Hz, 2 H, ArCH), 7.50 (m, 2 H, ArCH), 7.57 (ddd, *J* = 8.4, 6.9, 1.4 Hz, 2 H, ArCH), 7.64 - 7.68 (m, 1 H, ArCH), 7.89 (d, *J* = 7.3 Hz, 1 H, ArCH), 8.20 (d, *J* = 8.5 Hz, 1 H, ArCH); ^{13}C NMR (101 MHz, $CDCl_3$) δ ppm 24.4 (CH_3), 25.0 (CH_3), 26.5 (2 x CH_2), 26.6 (CH_2), 31.1 (CH_2), 33.2 (CH_2), 37.1 (CH), 54.0 (CHN), 55.8 (OCH_3), 58.8 (*C*HC=CH_2), 83.8 (OC), 113.1 (ArCH), 114.8 (ArCH), 122.4 (ArCH), 124.9 (ArCH), 125.2 (ArCH), 125.4 (ArCH), 125.6 (ArCH), 126.7 (ArCH), 129.2 (ArCH), 130.9 (ArC), 134.0 (ArC), 134.7 (C=*C*H_2), 138.1 (ArC), 142.4 (ArC), 150.8 (ArC), (B*C*=CH_2 not observed); ^{11}B NMR (128 MHz, $CDCl_3$) δ ppm 5.1; Specific rotation: $[\alpha]_D^{26}$ -91.5 (*c* 0.67, $CHCl_3$) for an enantiomerically enriched sample of 97:3 e.r.

Enantiomeric purity of **3d** was determined by HPLC analysis in comparison with authentic racemic material (97:3 e.r. shown; Lux 5 µm Amylose-1 column, 99:1 hexanes:*i*-PrOH, 0.3 mL/min, 20 °C, 254 nm).

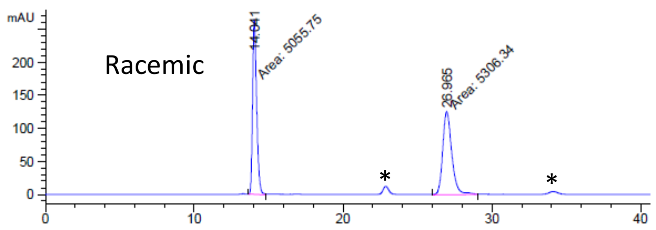

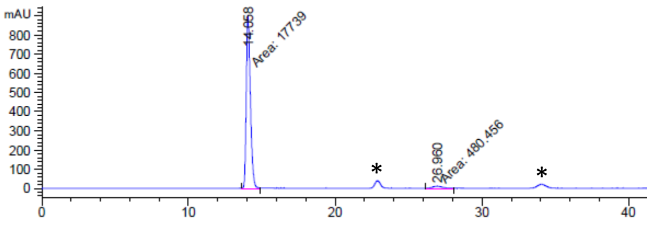

* = minor diastereoisomer

Peak #	RetTime [min]	Type	Width [min]	Area [mAU*s]	Height [mAU]	Area %
1	14.041	MM	0.3173	5055.75488	265.59125	48.7909
2	26.965	MM	0.7057	5306.34131	125.31339	51.2091

Peak #	RetTime [min]	Type	Width [min]	Area [mAU*s]	Height [mAU]	Area %
1	14.058	MM	0.3272	1.77390e4	903.45294	97.3630
2	26.960	MM	0.7429	480.45569	10.77880	2.6370

Peak #	Time (min)	Area (%)	Peak #	Time (min)	Area (%)
1	14.041	48.7909	1	14.058	97.3630
2	26.965	51.2091	2	26.960	2.6370

***N*-((1*R*,2*R*)-2-Cyclohexyl-1-(2-(methylthio)phenyl)-3-(4,4,5,5-tetramethyl-1,3,2-dioxaborolan-2-yl)but-3-en-1-yl)-4-methoxyaniline (3e)**

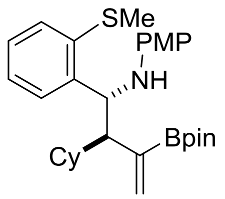

Prepared according to General Procedure 2, on a 0.258 mmol scale (95:5 d.r. of crude material), column chromatography (3% EtOAc in Hexanes) afforded the title compound as a brown gum (92 mg, 0.181 mmol, 70%).

A pure sample of the major diastereoisomer was obtained; MS (ES^+) *m/z*: 508 ($M+H^+$). HRMS calcd for $C_{30}H_{43}NBSO_3$: 508.3051. Found: 508.3057; ν_{max} (thin film/cm^{-1}): 3406, 2977, 2922, 2849, 1511, 1439, 1371, 1299, 1278, 1233, 1141, 1039; ^{1}H NMR (400 MHz, $CDCl_3$) δ ppm 1.12 - 1.25 (m, 4 H, CH_2), 1.30 - 1.39 (m, 12 H, 4 x CH_3), 1.54 - 1.79 (m, 5 H, CH_2), 1.90 - 2.08 (m, 2 H, CH_2 + CH), 2.43 (dd, J = 10.3, 4.0 Hz, 1 H, C*H*C=CH_2), 2.56 (s, 3 H, SCH_3), 3.68 (s, 3 H, OCH_3), 4.88 (d, *J* = 3.5 Hz, 1 H, C=CH_2), 5.10 (d, *J* = 3.8 Hz, 1 H, CHN), 5.66 (d, *J* = 3.5 Hz, 1 H, C=CH_2), 7.72 (br. s, 1 H, NH), 6.32 (d, *J* = 8.8 Hz, 2 H, ArCH), 6.67 (d, *J* = 9.03 Hz, 2 H, ArCH), 6.93 - 6.98 (m, 1 H, ArCH), 7.04 (dd, *J* = 7.8, 1.3 Hz, 1 H, ArCH), 7.13 (td, *J* = 7.5, 1.5 Hz, 1 H, ArCH), 7.18 - 7.22 (m, 1 H, ArCH); ^{13}C NMR (101 MHz, $CDCl_3$) δ ppm 16.1 (SCH_3), 24.4 (CH_3), 25.1 (CH_3), 26.4 (CH_2), 26.5 (CH_2), 26.6 (CH_2), 30.5 (CH_2), 33.1 (CH_2), 37.1 (CH), 54.7 (CHN), 55.8 (OCH_3), 57.4 (*C*HC=CH_2), 83.8 (OC), 112.9 (ArCH), 114.9 (ArCH), 124.3 (ArCH), 125.0 (ArCH), 126.7 (ArCH), 128.2 (ArCH), 135.0 (C=*C*H_2), 135.5 (ArC), 140.9 (ArC), 142.3 (ArC), 150.8 (ArC), (*C*=CH_2 not observed); ^{11}B NMR (128 MHz, $CDCl_3$) δ ppm -4.0, 31.5; Specific rotation: $[\alpha]_D^{27}$ -41.1 (*c* 0.68, $CHCl_3$) for an enantiomerically enriched sample of 84:16 e.r.

Enantiomeric purity of **3e** was determined by HPLC analysis in comparison with authentic racemic material (84:16 e.r. shown; Lux 5 µm Amylose-1 column, 99:1 hexanes:*i*-PrOH, 0.3 mL/min, 20 °C, 254 nm).

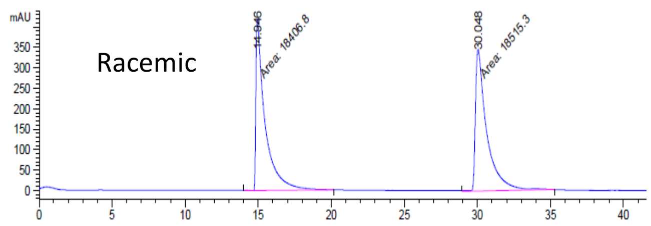

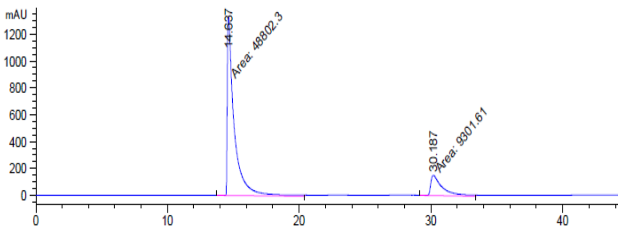

Peak #	RetTime [min]	Type	Width [min]	Area [mAU*s]	Height [mAU]	Area %
1	14.946	MM	0.7311	1.84068e4	419.63699	49.8531
2	30.048	MM	0.8911	1.85153e4	346.30432	50.1469

Peak #	RetTime [min]	Type	Width [min]	Area [mAU*s]	Height [mAU]	Area %
1	14.637	MM	0.6111	4.88023e4	1331.06421	83.9914
2	30.187	MM	1.0252	9301.60840	151.21303	16.0086

Peak #	Time (min)	Area (%)	Peak #	Time (min)	Area (%)
1	14.946	49.8531	1	14.637	83.9914
2	30.048	50.1469	2	30.187	16.0086

***N*-((1*R*,2*R*)-2-Cyclohexyl-1-phenyl-3-(4,4,5,5-tetramethyl-1,3,2-dioxaborolan-2-yl)but-3-en-1-yl)-4-methoxyaniline (3f)**

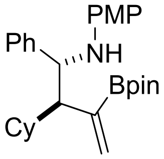

Prepared according to General Procedure 2, on a 0.258 mmol scale (74:26 d.r. of crude material), column chromatography (3% EtOAc in Hexanes) afforded the title compound as an orange gum (91 mg, 0.197 mmol, 76%).

A pure sample of the major diastereoisomer was obtained; MS (ES^+) *m/z*: 462 ($M+H^+$). HRMS calcd for $C_{29}H_{41}NBO_3$: 462.3179. Found: 462.3162; ν_{max} (thin film/cm^{-1}): 3407, 2976, 2925, 2850, 1510, 1450, 1421, 1389, 1371, 1300, 1233, 1218, 1140, 1115, 1069, 1040; ^{1}H NMR (400 MHz, $CDCl_3$) δ ppm 0.73 - 0.85 (m, 1 H, CH_2), 1.02 (m, 1 H, CH_2), 1.09 - 1.23 (m, 3 H, CH_2), 1.28 (s, 6 H, 2 x CH_3), 1.31 (s, 6 H, 2 x CH_3), 1.60 - 1.75 (m, 4 H, CH_2), 1.75 - 1.89 (m, 2 H, CH_2 + CH), 2.24 (dd, *J* = 9.0, 5.3 Hz, 1 H, C*H*C=CH_2), 3.68 (s, 3 H, OCH_3), 4.67 (d, *J* = 5.3 Hz, 1 H, CHN), 5.02 (d, *J* = 3.5 Hz, 1 H, C=CH_2), 5.33 (br. s, 1 H, NH), 5.72 (d, *J* = 3.5 Hz, 1 H, C=CH_2), 6.38 (d, *J* = 8.8 Hz, 2 H, ArCH), 6.66 (d, *J* = 9.0 Hz, 2 H, ArCH), 7.13 (tt, *J* = 6.9, 1.3 Hz, 1 H, ArCH), 7.16 - 7.21 (m, 2 H, ArCH), 7.21 - 7.26 (m, 2 H, ArCH); ^{13}C NMR (101 MHz, $CDCl_3$) δ ppm 24.4 (CH_3), 25.0 (CH_3), 26.4 (CH_2), 26.4 (CH_2), 26.6 (CH_2), 31.4 (CH_2), 32.3 (CH_2), 36.9 (CH), 55.8 (CHN), 58.1 (OCH_3), 61.4 (*C*HC=CH_2), 83.6 (OC), 113.3 (ArCH), 114.8 (ArCH), 126.1 (ArCH), 127.3 (ArCH), 127.9 (ArCH), 134.6 (C=*C*H_2), 142.6 (ArC), 144.0 (ArC), 150.9

(ArC), (B*C*=CH_2 not observed); ^{11}B NMR (128 MHz, $CDCl_3$) δ ppm 1.5; Specific rotation: $[\alpha]_D^{27}$ -31.7 (*c* 0.90, $CHCl_3$) for an enantiomerically enriched sample of 98:2 e.r.

Enantiomeric purity of **3f** was determined by HPLC analysis in comparison with authentic racemic material (98:2 e.r. shown; Lux 5 µm Amylose-1 column, 99:1 hexanes:*i*-PrOH, 0.3 mL/min, 20 °C, 254 nm).

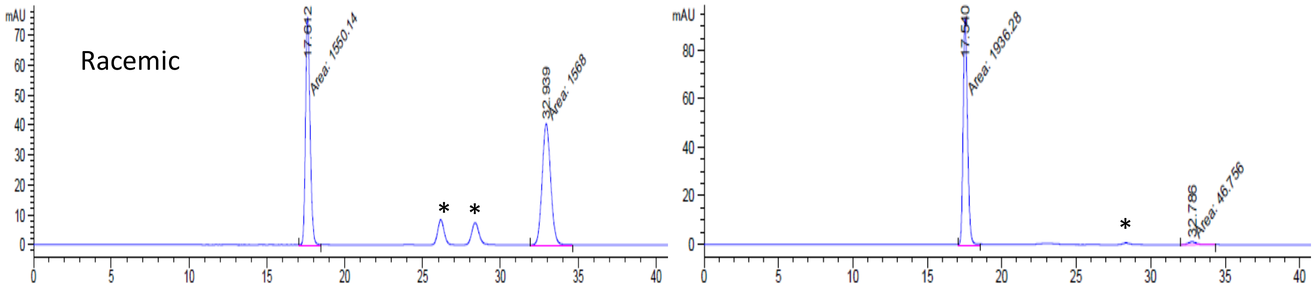

* = minor diastereoisomer

Peak #	RetTime [min]	Type	Width [min]	Area [mAU*s]	Height [mAU]	Area %
1	17.612	MM	0.3394	1550.13989	76.11987	49.7136
2	32.939	MM	0.6432	1568.00183	40.63127	50.2864

Peak #	RetTime [min]	Type	Width [min]	Area [mAU*s]	Height [mAU]	Area %
1	17.540	MM	0.3433	1936.27563	94.01144	97.6422
2	32.786	MM	0.6662	46.75599	1.16968	2.3578

**Peak #**	**Time (min)**	**Area (%)**	**Peak #**	**Time (min)**	**Area (%)**
1	17.612	49.7136	1	17.540	97.6422
2	32.939	50.2864	2	32.786	2.3578

***N*-((1*R*,2*R*)-2-Cyclohexyl-1-(3-methoxyphenyl)-3-(4,4,5,5-tetramethyl-1,3,2-dioxaborolan-2-yl)but-3-en-1-yl)-4-methoxyaniline (3g)**

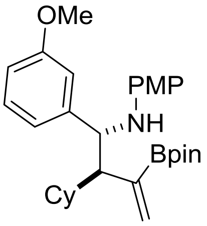

Prepared according to General Procedure 2, on a 0.258 mmol scale (82:18 d.r. of crude material), column chromatography (4% EtOAc in Hexanes) afforded the title compound as an orange gum (111 mg, 0.225 mmol, 87%).

A pure sample of the major diastereoisomer was obtained; MS (ES^+) *m/z*: 492 ($M+H^+$). HRMS calcd for $C_{30}H_{43}NBO_4$: 492.3280. Found: 492.3276; ν_{max} (thin film/cm^{-1}): 3407, 2976, 2925, 2849, 1600, 1511, 1486, 1435, 1371, 1300, 1233, 1168, 1140, 1081, 1042; ^{1}H NMR (500 MHz, $CDCl_3$) δ ppm 0.79 (q, *J* = 10.7 Hz, 1 H, CH_2), 1.01 (qd, *J* = 11.7, 3.0 Hz, 1 H, CH_2), 1.09 - 1.25 (m, 3 H, CH_2), 1.28 (s, 6 H, 2 x CH_3), 1.30 (s, 6 H, 2 x CH_3), 1.58 - 1.73 (m, 4 H, CH_2), 1.74 - 1.88 (m, 2 H, CH_2 + CH), 2.23 (dd, *J* = 9.0, 5.3 Hz, 1 H, C*H*C=CH_2), 3.68 (s, 3 H, OCH_3), 3.76 (s, 3 H, OCH_3), 4.63 (d, *J* = 5.2 Hz, 1 H, CHN), 5.08 (d, *J* = 3.5 Hz, 1 H, C=CH_2), 5.27 (br. s, 1 H, NH), 5.74 (d, *J* = 3.7 Hz, 1 H, C=CH_2), 6.38 (m, 2 H, ArCH), 6.63 - 6.71 (m, 3 H, ArCH), 6.75 (s, 1 H, ArCH), 6.80 (d, *J* = 7.6 Hz, 1 H, ArCH), 7.15 (t, *J* = 7.9 Hz, 1 H, ArCH); ^{13}C NMR (101 MHz, $CDCl_3$) δ ppm 24.4 (CH_3), 24.9 (CH_3), 26.3 (CH_2), 26.4 (CH_2), 26.6 (CH_2), 31.4 (CH_2), 32.2 (CH_2), 36.9 (CH), 55.1 (OCH_3), 55.8 (OCH_3), 58.2 (CHN), 61.2 (*C*HC=CH_2), 83.6 (OC), 111.4 (ArCH), 113.0 (ArCH), 113.3 (ArCH), 114.7 (ArCH), 119.8 (ArCH), 128.8 (ArCH), 134.5 (C=*C*H_2), 142.6 (ArC), 146.0 (ArC), 150.9 (ArC), 159.4 (ArC), (B*C*=CH_2 not observed); ^{11}B NMR (128 MHz, $CDCl_3$) δ ppm 22.4, 29.3; Specific rotation: $[\alpha]_D^{27}$ -28.5 (*c* 0.82, $CHCl_3$) for an enantiomerically enriched sample of 98:2 e.r.

Enantiomeric purity of **3g** was determined by HPLC analysis in comparison with authentic racemic material (98:2 e.r. shown; Lux 5 µm Amylose-1 column, 99:1 hexanes:*i*-PrOH, 0.3 mL/min, 10 °C, 254 nm).

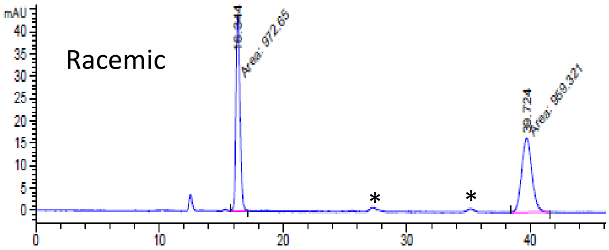

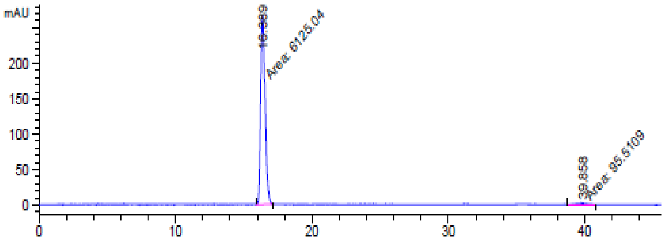

* = minor diastereoisomer

Peak #	RetTime [min]	Type	Width [min]	Area [mAU*s]	Height [mAU]	Area %
1	16.344	MM	0.3689	972.65021	43.94279	50.3450
2	39.724	MM	0.9625	959.32123	16.61178	49.6550

Peak #	RetTime [min]	Type	Width [min]	Area [mAU*s]	Height [mAU]	Area %
1	16.389	MM	0.3814	6125.04248	267.63364	98.4646
2	39.858	MM	0.9346	95.51088	1.70328	1.5354

Peak #	Time (min)	Area (%)	Peak #	Time (min)	Area (%)
1	16.344	50.3450	1	16.389	98.4646
2	39.724	49.6550	2	39.858	1.5354

N-((1R,2R)-2-Cyclohexyl-1-(4-methoxyphenyl)-3-(4,4,5,5-tetramethyl-1,3,2-dioxaborolan-2-yl)but-3-en-1-yl)-4-methoxyaniline (3h)

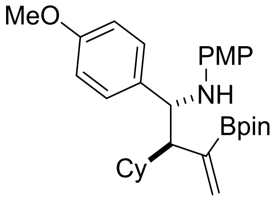

Prepared according to General Procedure 2, on a 0.258 mmol scale (70:30 d.r. of crude material), column chromatography (4% EtOAc in Hexanes) afforded the title compound as a mixture of diastereoisomers, as an orange gum (84 mg, 0.172 mmol, 66%, 78:22 d.r.).

Data for the major diastereoisomer: MS (ES$^+$) *m/z*: 492 (M+H$^+$). HRMS calcd for $C_{30}H_{43}NBO_4$: 492.3285. Found: 492.3305; ν_{max} (thin film/cm^{-1}): 3409, 2927, 2850, 1610, 1511, 1421, 1371, 1301, 1243, 1170, 1142; ^{1}H NMR (400 MHz, $CDCl_3$) δ ppm 0.68 - 0.92 (m, 1 H, CH_2), 0.93 - 1.08 (m, 1 H, CH_2), 1.09 - 1.24 (m, 4 H, CH_2), 1.27 (s, 6 H, 2 x CH_3), 1.29 (s, 6 H, 2 x CH_3), 1.53 - 1.92 (m, 5 H, CH_2 + CH), 2.19 (dd, *J* = 8.7, 5.4 Hz, 1 H, C*H*C=CH_2), 3.68 (s, 3 H, OCH_3), 3.77 (s, 3 H, OCH_3), 4.62 (d, *J* = 5.3 Hz, 1 H, CHN), 5.06 (d, *J* = 3.5 Hz, 1 H, C=CH_2), 5.75 (d, *J* = 3.8 Hz, 1 H, C=CH_2), 6.37 (d, *J* = 8.8 Hz, 2 H, ArCH), 6.63 - 6.67 (m, 2 H, ArCH), 6.78 (d, *J* = 8.5 Hz, 2 H, ArCH), 7.09 (d, *J* = 8.5 Hz, 2 H, ArCH), (NH not observed); ^{13}C NMR (101 MHz, $CDCl_3$) δ ppm 24.4 (CH_3), 24.9 (CH_3), 26.4 (CH_2), 26.4 (CH_2), 26.6 (CH_2), 31.4 (CH_2), 32.1 (CH_2), 36.9 (CH), 55.1 (OCH_3), 55.8 (OCH_3), 57.5 (CHN), 61.4 (*C*HC=CH_2), 83.6 (OC), 113.3 (ArCH), 113.4 (ArCH), 114.7 (ArCH), 128.2 (ArCH), 134.6 (C=*C*H_2), 136.0 (ArC), 142.6 (ArC), 150.9 (ArC), 157.9 (ArC), (B*C*=CH_2 not observed); ^{11}B NMR (128 MHz, $CDCl_3$) δ ppm 4.4; Specific rotation: $[\alpha]_D^{27}$ -18.9 (*c* 1.01, $CHCl_3$) for an enantiomerically enriched sample of 98:2 e.r.

Enantiomeric purity of **3h** was determined by HPLC analysis in comparison with authentic racemic material (98:2 e.r. shown; Lux 5 µm Amylose-1 column, 99:1 hexanes:*i*-PrOH, 0.3 mL/min, 20 °C, 254 nm).

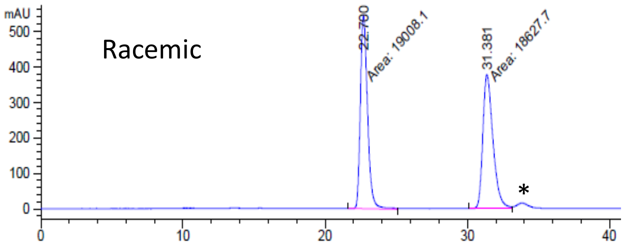

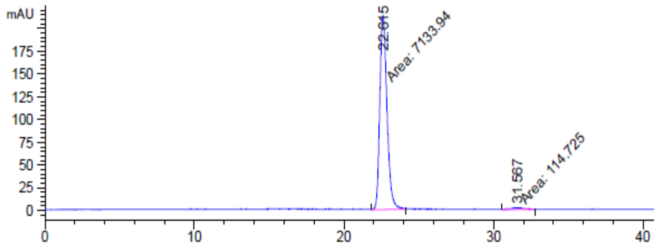

* = minor diastereoisomer

Peak #	RetTime [min]	Type	Width [min]	Area [mAU*s]	Height [mAU]	Area %
1	22.700	MM	0.5834	1.90081e4	543.05115	50.5053
2	31.381	MF	0.8268	1.86277e4	375.49292	49.4947

Peak #	RetTime [min]	Type	Width [min]	Area [mAU*s]	Height [mAU]	Area %
1	22.615	MM	0.5573	7133.93555	213.36166	98.4173
2	31.567	MM	0.9809	114.72469	1.94921	1.5827

Peak #	Time (min)	Area (%)	Peak #	Time (min)	Area (%)
1	22.700	50.5053	1	22.615	98.4173
2	31.381	49.4947	2	31.567	1.5827

***N*-((1*R*,2*R*)-2-Cyclohexyl-3-(4,4,5,5-tetramethyl-1,3,2-dioxaborolan-2-yl)-1-(4-(trifluoromethyl)phenyl)but-3-en-1-yl)-4-methoxyaniline (3i)**

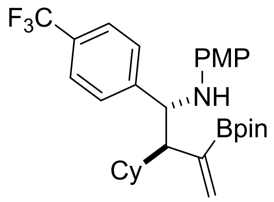

Prepared according to General Procedure 2, on a 0.258 mmol scale (76:24 d.r. of crude material), column chromatography (4% EtOAc in Hexanes) afforded the title compound as an orange gum (117 mg, 0.220 mmol, 85%).

A pure sample of the major diastereoisomer was obtained; MS (ES^+) *m/z*: 530 ($M+H^+$). HRMS calcd for $C_{30}H_{40}NBO_2F_3$: 530.3062. Found: 530.3059; ν_{max} (thin film/cm^{-1}): 3405, 2977, 2927, 2851, 1617, 1511, 1420, 1371, 1334, 1234, 1066, 1040, 1016; ^{1}H NMR (400 MHz, $CDCl_3$) δ ppm 0.73 - 0.85 (m, 1 H, CH_2), 0.95 - 1.08 (m, 1 H, CH_2), 1.10 - 1.24 (m, 3 H, CH_2), 1.29 (s, 6 H, 2 x CH_3), 1.32 (s, 6 H, 2 x CH_3), 1.57 - 1.77 (m, 4 H, CH_2 + CH), 1.78 – 1.94 (m, 2 H, CH_2), 2.23 (dd, *J* = 9.4, 4.6 Hz, 1 H, C*H*C=CH_2), 3.69 (s, 3 H, OCH_3), 4.72 (br. s, 1 H, CHN), 4.99 (d, *J* = 3.5 Hz, 1 H, C=CH_2), 5.43 (br. s, 1 H, NH), 5.72 (d, *J* = 3.5 Hz, 1 H, C=CH_2), 6.34 (d, *J* = 9.0 Hz, 2 H, ArCH), 6.67 (d, *J* = 8.8 Hz, 2 H, ArCH), 7.29 (d, *J* = 8.3 Hz, 2 H, ArCH), 7.49 (d, *J* = 8.0 Hz, 2 H, ArCH); ^{13}C NMR (101 MHz, $CDCl_3$) δ ppm 24.4 (CH_3), 24.9 (CH_3), 26.3 (CH_2), 26.4 (CH_2), 26.5 (CH_2), 31.2 (CH_2), 32.4 (CH_2), 36.8 (CH), 55.8 (CHN), 57.9 (OCH_3), 61.1 (*C*HC=CH_2), 83.8 (OC), 113.3 (ArCH), 114.8 (ArCH), 124.4 (q, *J* = 272.0 Hz, CF_3), 124.9 (ArCH), 127.5 (ArCH), 128.4 (q, *J* = 31.7 Hz, *C*CF_3), 135.1 (C=*C*H_2), 142.0 (ArC), 148.5 (ArC), 151.2 (ArC), (B*C*=CH_2 not observed); ^{11}B NMR (128 MHz, $CDCl_3$) δ ppm 29.2; ^{19}F NMR (376 MHz, $CDCl_3$) δ ppm -62.2; Specific rotation: $[\alpha]_D^{27}$ -26.2 (*c* 1.06, $CHCl_3$) for an enantiomerically enriched sample of 96:4 e.r.

Enantiomeric purity of **3i** was determined by HPLC analysis in comparison with authentic racemic material (96:4 e.r. shown; Lux 5 µm Amylose-1 column, 99:1 hexanes:*i*-PrOH, 0.3 mL/min, 20 °C, 254 nm).

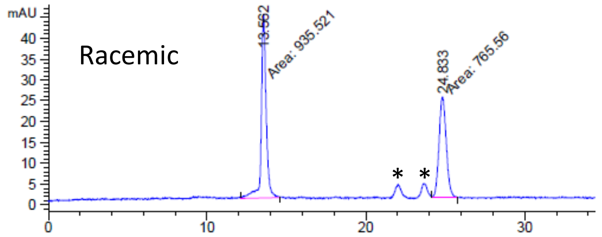

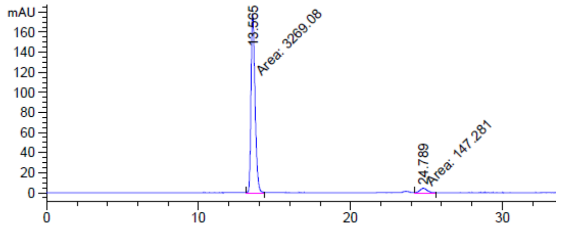

* = minor diastereoisomer

Peak #	RetTime [min]	Type	Width [min]	Area [mAU*s]	Height [mAU]	Area %
1	13.944	BB	0.2714	423.63309	23.59918	50.5866
2	26.738	BB	0.5190	413.80804	12.04012	49.4134

Peak #	RetTime [min]	Type	Width [min]	Area [mAU*s]	Height [mAU]	Area %
1	13.836	BB	0.2735	2363.40601	130.36607	96.0079
2	26.385	BB	0.5097	98.27366	2.82595	3.9921

**Peak #**	**Time (min)**	**Area (%)**	**Peak #**	**Time (min)**	**Area (%)**
1	13.944	50.5866	1	13.836	96.0079
2	26.738	49.4134	2	26.385	3.9921

***N*-((1*R*,2*R*)-1-(4-Bromophenyl)-2-cyclohexyl-3-(4,4,5,5-tetramethyl-1,3,2-dioxaborolan-2-yl)but-3-en-1-yl)-4-methoxyaniline (3j)**

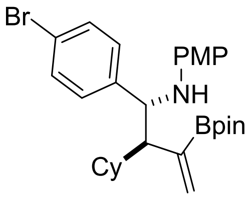

Prepared according to General Procedure 2, on a 0.258 mmol scale (76:24 d.r. of crude material), column chromatography (4% EtOAc in Hexanes) afforded the title compound as a yellow gum (120 mg, 0.222 mmol, 86%).

A pure sample of the major diastereoisomer was obtained; MS (ES$^+$) *m/z*: 542 (M+H$^+$). HRMS calcd for $C_{29}H_{40}NBO_3Br$: 540.2285. Found: 540.2273; ν_{max} (thin film/cm^{-1}): 3405, 2976, 2926, 2850, 1515, 1484, 1421, 1371, 1302, 1234, 1167, 1141, 1071, 1040, 1009; ^{1}H NMR (400 MHz, $CDCl_3$) δ ppm 0.72 - 0.85 (m, 1 H, CH_2), 0.93 - 1.06 (m, 1 H, CH_2), 1.08 - 1.24 (m, 3 H, CH_2), 1.28 (s, 6 H, 2 x CH_3), 1.31 (s, 6 H, 2 x CH_3), 1.59 - 1.74 (m, 4 H, CH_2), 1.75 - 1.88 (m, 2 H, CH + CH_2), 2.18 (dd, *J* = 9.3, 5.0 Hz, 1 H, C*H*C=CH_2), 3.69 (s, 3 H, OCH_3), 4.62 (t, *J* = 4.9 Hz, 1 H, CHN), 5.03 (d, *J* = 3.5 Hz, 1 H, C=CH_2), 5.33 (d, *J* = 4.8 Hz, 1 H, NH), 5.74 (d, *J* = 3.5 Hz, 1 H, C=CH_2), 6.34 (d, *J* = 8.8 Hz, 2 H, ArCH), 6.66 (d, *J* = 9.0 Hz, 2 H, ArCH), 7.06 (d, *J* = 8.3 Hz, 2 H, ArCH), 7.35 (d, *J* = 8.3 Hz, 2 H, ArCH); ^{13}C NMR (101 MHz, $CDCl_3$) δ ppm 24.4 (CH_3), 24.9 (CH_3), 26.3 (CH_2), 26.4 (CH_2), 26.5 (CH_2), 31.3 (CH_2), 32.3 (CH_2), 36.8 (CH), 55.8 (OCH_3), 57.7 (CHN), 61.2 (*C*HC=CH_2), 83.7 (OC), 113.3 (ArCH), 114.8 (ArCH), 119.7 (ArC), 129.0 (ArCH), 131.0 (ArCH), 135.0 (C=*C*H_2), 142.1 (ArC), 143.2 (ArC), 151.1 (ArC), (B*C*=CH_2 not observed); ^{11}B NMR

(128 MHz, $CDCl_3$) δ ppm 9.7; Specific rotation: $[\alpha]_D^{27}$ -7.23 (*c* 0.50, $CHCl_3$) for an enantiomerically enriched sample of 98:2 e.r.

Enantiomeric purity of **3j** was determined by HPLC analysis in comparison with authentic racemic material (98:2 e.r. shown; Lux 5 µm Amylose-1 column, 99:1 hexanes:*i*-PrOH, 0.3 mL/min, 20 °C, 254 nm).

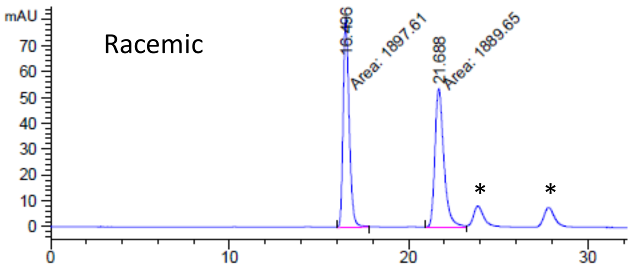

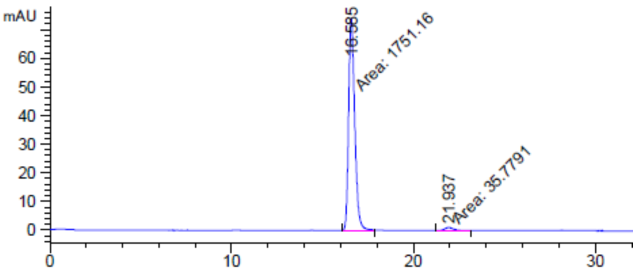

* = minor diastereoisomer

Peak #	RetTime [min]	Type	Width [min]	Area [mAU*s]	Height [mAU]	Area %
1	16.496	BB	0.3566	1889.26074	80.84824	50.2879
2	21.688	MF	0.5825	1867.62842	53.43529	49.7121

Peak #	RetTime [min]	Type	Width [min]	Area [mAU*s]	Height [mAU]	Area %
1	16.585	BB	0.3611	1751.65149	74.27771	97.8695
2	21.937	MM	0.6022	38.13092	1.05533	2.1305

Peak #	Time (min)	Area (%)	Peak #	Time (min)	Area (%)
1	16.495	50.2879	1	16.585	97.8695
2	21.688	49.7121	2	21.937	2.1305

***N*-((1*R*,2*R*)-2-Cyclohexyl-1-(furan-2-yl)-3-(4,4,5,5-tetramethyl-1,3,2-dioxaborolan-2-yl)but-3-en-1-yl)-4-methoxyaniline (3k)**

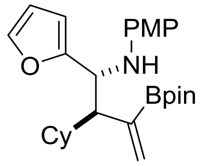

Prepared according to General Procedure 2, on a 0.258 mmol scale (68:32 d.r. of crude material), column chromatography (3% EtOAc in Hexanes) afforded the title compound as a brown gum (70.0 mg, 0.155 mmol, 60%).

A pure sample of the major diastereoisomer was obtained; MS (ES^+) *m/z*: 452 ($M+H^+$). HRMS calcd for $C_{27}H_{39}NBO_4$: 452.2972. Found: 452.2976; ν_{max} (thin film/cm^{-1}): 3404, 2976, 2926, 2850, 1516, 1448, 1422, 1371, 1301, 1233, 1142, 1039; ^1H NMR (500 MHz, $CDCl_3$) δ ppm 0.75 - 0.86 (m, 1 H, CH_2), 0.99 (qd, *J* = 12.2, 2.7 Hz, 1 H, CH_2), 1.07 - 1.20 (m, 3 H, CH_2), 1.25 (s, 6 H, 2 x CH_3), 1.26 (s, 6 H, 2 x

CH_3), 1.60 - 1.77 (m, 6 H, CH_2 + CH), 2.23 (apparent t, J = 7.0 Hz, 1 H, $C\mathit{H}C{=}CH_2$), 3.71 (s, 3 H, OCH_3), 4.65 (d, J = 6.1 Hz, 1 H, CHN), 4.84 (br. s, 1 H, NH), 5.37 (d, J = 3.5 Hz, 1 H, $C{=}CH_2$), 5.88 (d, J = 3.5 Hz, 1 H, $C{=}CH_2$), 6.23 (apparent s, 1 H, ArCH), 6.47 (d, J = 8.7 Hz, 2 H, ArCH), 6.70 (d, J = 8.7 Hz, 2 H, ArCH), 7.18 (apparent s, 1 H, ArCH), 7.30 (apparent s, 1 H, ArCH); ^{13}C NMR (126 MHz, $CDCl_3$) δ ppm 24.4 (CH_3), 24.9 (CH_3), 26.4 (CH_2), 26.5 (CH_2), 26.5 (CH_2), 31.5 (CH_2), 31.7 (CH_2), 36.9 (CH), 50.7 (CHN), 55.8 (OCH_3), 59.6 ($\mathit{C}HC{=}CH_2$), 83.5 (OC), 109.6 (ArCH), 113.6 (ArCH), 114.7 (ArCH), 128.3 (ArCH), 134.2 ($C{=}\mathit{C}H_2$), 140.1 (ArC), 142.6 (ArC), 142.6 (ArCH), 151.2 (ArC), ($B\mathit{C}{=}CH_2$ not observed); ^{11}B NMR (128 MHz, $CDCl_3$) δ ppm 30.6; Specific rotation: $[\alpha]_D^{27}$ -18.7 (*c* 0.65, $CHCl_3$) for an enantiomerically enriched sample of 97:3 e.r.

Enantiomeric purity of **3k** was determined by HPLC analysis in comparison with authentic racemic material (97:3 e.r. shown; Lux 5 µm Amylose-1 column, 99:1 hexanes:*i*-PrOH, 0.3 mL/min, 20 °C, 254 nm).

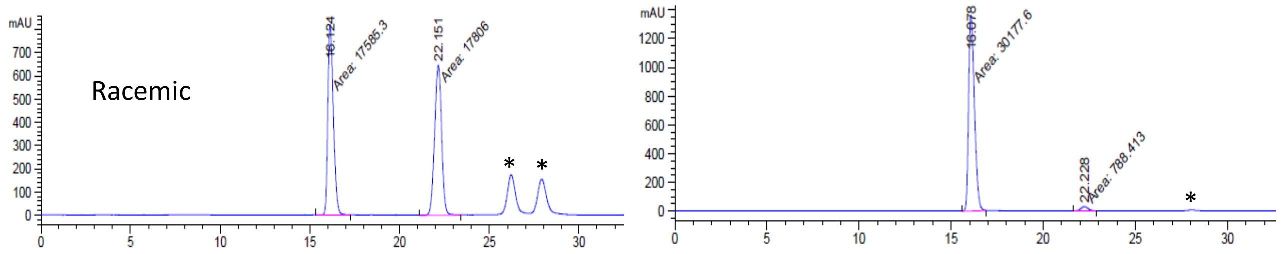

* = minor diastereoisomer

Peak #	RetTime [min]	Type	Width [min]	Area [mAU*s]	Height [mAU]	Area %
1	16.124	MM	0.3576	1.75853e4	819.48541	49.6882
2	22.151	MM	0.4587	1.78060e4	646.94922	50.3118

Peak #	RetTime [min]	Type	Width [min]	Area [mAU*s]	Height [mAU]	Area %
1	16.078	MM	0.3685	3.01776e4	1364.87659	97.4539
2	22.228	MM	0.4413	788.41272	29.77478	2.5461

Peak #	Time (min)	Area (%)	Peak #	Time (min)	Area (%)
1	16.124	49.6882	1	16.078	97.4539
2	22.151	50.3118	2	22.228	2.5461

***N*-((1*R*,2*R*)-2-Cyclohexyl-1-(furan-3-yl)-3-(4,4,5,5-tetramethyl-1,3,2-dioxaborolan-2-yl)but-3-en-1-yl)-4-methoxyaniline (3l)**

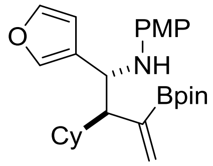

Prepared according to General Procedure 2, on a 0.258 mmol scale (68:32 d.r. of crude material), column chromatography (3% EtOAc in Hexanes) afforded the title compound as a mixture of diastereoisomers, as a brown gum (40 mg, 0.0886 mmol, 34%, 81:19 d.r.).

Data for the major diastereoisomer: MS (ES$^+$) *m/z*: 452 (M+H$^+$). HRMS calcd for $C_{27}H_{39}NBO_4$: 452.2967. Found: 452.2965; ν_{max} (thin film/cm^{-1}): 3404, 2978, 2926, 2851, 1617, 1603, 1511, 1465, 1447, 1422, 1390, 1379, 1371, 1363, 1339, 1301, 1274, 1233, 1214, 1165, 1141, 1113, 1079, 1063, 1039; ^{1}H NMR (400 MHz, $CDCl_3$) δ ppm 0.74 - 0.87 (m, 1 H, CH_2), 0.95 - 1.06 (m, 1 H, CH_2), 1.08 - 1.18 (m, 3 H, CH_2), 1.25 (s, 6 H, 2 x CH_3), 1.26 (s, 6 H, 2 x CH_3), 1.58 - 1.78 (m, 6 H, CH_2 + CH), 2.23 (dd, *J* = 7.8, 6.3 Hz, 1 H, C*H*C=CH_2), 3.71 (s, 3 H, OCH_3), 4.65 (d, *J* = 6.0 Hz, 1 H, CHN), 5.37 (d, *J* = 3.5 Hz, 1 H, C=CH_2), 5.88 (d, *J* = 3.5 Hz, 1 H, C=CH_2), 6.23 (dd, *J* = 1.8, 0.8 Hz, 1 H, ArCH), 6.47 (d, *J* = 9.0 Hz, 2 H, ArCH), 6.70 (d, *J* = 9.0 Hz, 2 H, ArCH), 7.17 - 7.19 (m, 1 H, ArCH), 7.30 (t, *J* = 1.8 Hz, 1 H, ArCH), (NH not observed); ^{13}C NMR (101 MHz, $CDCl_3$) δ ppm 24.4 (CH_3), 24.9 (CH_3), 26.4 (CH_2), 26.5 (CH_2), 26.5 (CH_2), 31.5 (CH_2), 31.7 (CH_2), 36.9 (CH), 50.7 (CHN), 55.8 (OCH_3), 59.6 (*C*HC=CH_2), 83.5 (OC), 109.6 (ArCH), 113.6 (ArCH), 114.7 (ArCH), 128.3 (ArC), 134.2 (C=*C*H_2), 140.1 (ArCH), 142.6 (ArCH), 142.6 (ArC), 151.2 (ArC), (B*C*=CH_2 not observed); ^{11}B NMR (128 MHz, $CDCl_3$) δ ppm -4.3, 30.3; Specific rotation: $[\alpha]_D^{27}$ -9.41 (*c* 0.18, $CHCl_3$) for an enantiomerically enriched sample of 93:7 e.r.

Enantiomeric purity of **3l** was determined by HPLC analysis in comparison with authentic racemic material (93:7 e.r. shown; Lux 5 µm Amylose-1 column, 99:1 hexanes:*i*-PrOH, 0.3 mL/min, 20 °C, 254 nm).

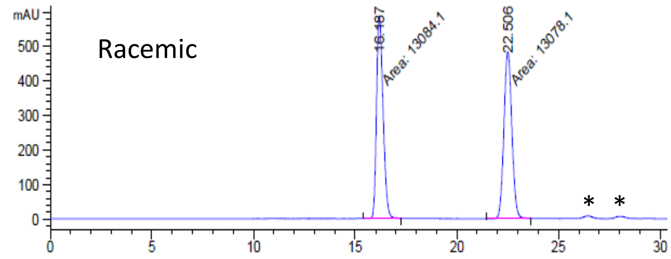

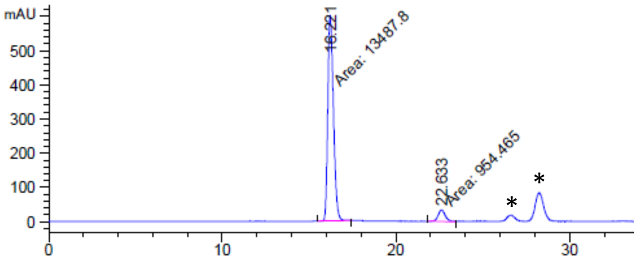

* = minor diastereoisomer

Peak #	RetTime [min]	Type	Width [min]	Area [mAU*s]	Height [mAU]	Area %
1	16.187	MM	0.3726	1.30841e4	585.24603	50.0114
2	22.506	MM	0.4524	1.30781e4	481.83466	49.9886

Peak #	RetTime [min]	Type	Width [min]	Area [mAU*s]	Height [mAU]	Area %
1	16.221	MM	0.3751	1.34878e4	599.31647	93.3912
2	22.633	MM	0.4695	954.46497	33.88182	6.6088

**Peak #**	**Time (min)**	**Area (%)**	**Peak #**	**Time (min)**	**Area (%)**
1	16.187	50.0114	1	16.221	93.3912
2	22.506	49.9886	2	22.633	6.6088

4-Methoxy-*N*-((1*R*,2*R*)-2-(1-(4,4,5,5-tetramethyl-1,3,2-dioxaborolan-2-yl)vinyl)-1-(*o*-tolyl) decyl)aniline (3m)

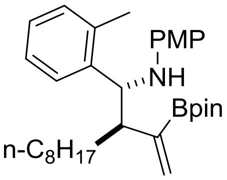

Prepared according to General Procedure 2, on a 0.258 mmol scale (93:7 d.r. of crude material), column chromatography (5% EtOAc in Hexanes) afforded the title compound as an orange gum (89 mg, 0.177 mmol, 69%).

A pure sample of the major diastereoisomer was obtained; MS (ES$^+$) *m/z*: 505 (M+H$^+$). HRMS calcd for $C_{32}H_{49}NBO_3Na$: 506.3806. Found: 506.3802; ν_{max} (thin film/cm^{-1}): 3406, 2924, 2854, 1606, 1510, 1463, 1417, 1365, 1307, 1237, 1167, 1141, 1110, 1041; ^{1}H NMR (500 MHz, $CDCl_3$) δ ppm 0.87 (t, *J* = 7.0 Hz, 3 H, CH_3), 1.01 - 1.21 (m, 10 H, CH_2), 1.23 (s, 6 H, 2 x CH_3), 1.24 (s, 6 H, 2 x CH_3), 1.26 - 1.37 (m, 3 H, CH_2), 1.65 - 1.76 (m, 1 H, CH_2), 2.33 - 2.44 (m, 1 H, C*H*C=CH_2), 2.49 (s, 3 H, Ar-CH_3), 3.66 (s, 3 H, OCH_3), 4.54 (br. s, 1 H, NH), 4.63 (d, *J* = 7.2 Hz, 1 H, CHN), 5.44 (d, *J* = 2.9 Hz, 1 H, C=CH_2), 5.90 (d, *J* = 2.9 Hz, 1 H, C=CH_2), 6.32 (d, *J* = 8.4 Hz, 2 H, ArCH), 6.62 (d, *J* = 8.4 Hz, 2 H, ArCH), 7.04 - 7.15 (m, 3 H, ArCH), 7.24 - 7.27 (m, 1 H, ArCH); ^{13}C NMR (126 MHz, $CDCl_3$) δ ppm 14.1 (CH_3), 19.6 (Ar-CH_3), 22.6 (CH_2), 24.7 (CH_3), 24.8 (CH_3), 27.9 (CH_2), 29.2 (CH_2), 29.4 (CH_2), 29.4 (CH_2), 30.4 (CH_2), 31.8 (CH_2), 54.1 (CHN), 55.8 (OCH_3), 58.1 (*C*HC=CH_2), 83.4 (OC), 113.8 (ArCH), 114.6 (ArCH), 126.0 (ArCH), 126.1 (ArCH), 126.8 (ArCH), 130.0 (ArCH), 132.8 (C=*C*H_2), 135.4 (ArC), 141.8 (ArC), 142.6 (ArC), 151.3 (ArC), (B*C*=CH_2 not observed); ^{11}B NMR (128 MHz, $CDCl_3$) δ ppm 10.2; Specific rotation: $[\alpha]_D^{27}$ -35.5 (*c* 0.91, $CHCl_3$) for an enantiomerically enriched sample of 97:3 e.r.

Enantiomeric purity of **3m** was determined by HPLC analysis in comparison with authentic racemic material (97:3 e.r. shown; Chiralcel OD-H column, 99.5:0.5 hexanes:*i*-PrOH, 0.2 mL/min, 20 °C, 254 nm).

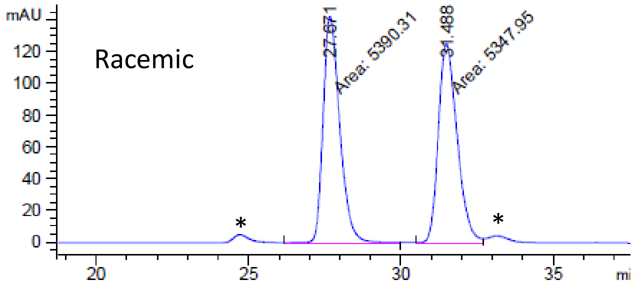

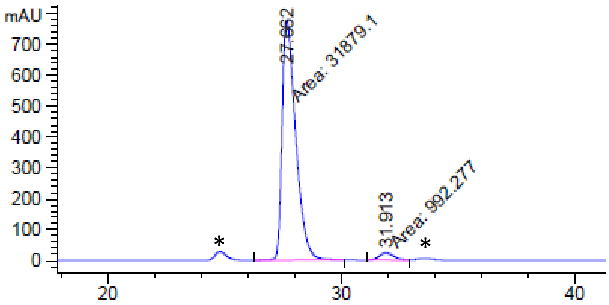

* = minor diastereoisomer

Peak #	RetTime [min]	Type	Width [min]	Area [mAU*s]	Height [mAU]	Area %
1	27.671	MM	0.6318	5390.31396	142.20291	50.1973
2	31.488	MF	0.7143	5347.94873	124.78006	49.8027

Peak #	RetTime [min]	Type	Width [min]	Area [mAU*s]	Height [mAU]	Area %
1	27.662	MM	0.6747	3.18791e4	787.50946	96.9813
2	31.913	MM	0.7222	992.27704	22.89993	3.0187

Peak #	Time (min)	Area (%)	Peak #	Time (min)	Area (%)
1	27.671	50.1973	1	27.662	96.9813
2	31.488	49.8027	2	31.913	3.0187

***N*-((1*R*,2*R*)-6-((*tert*-Butyldimethylsilyl)oxy)-2-(1-(4,4,5,5-tetramethyl-1,3,2-dioxaborolan-2-yl)vinyl)-1-(*o*-tolyl)hexyl)-4-methoxyaniline (3n)**

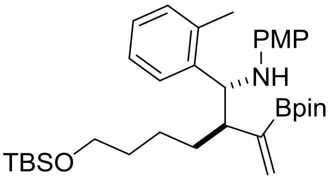

Prepared according to General Procedure 2, on a 0.129 mmol scale (93:7 d.r. of crude material), column chromatography (3% EtOAc in Hexanes) afforded the title compound as an orange gum (39 mg, 0.0645 mmol, 50%).

A pure sample of the major diastereoisomer was obtained; MS (ES^+) *m/z*: 580 ($M+H^+$). HRMS calcd for $C_{34}H_{55}NBO_4Si$: 580.3988. Found: 580.3975; ν_{max} (thin film/cm^{-1}): 3407, 2929, 2857, 1606, 1511, 1463, 1441, 1417, 1379, 1363, 1308, 1237, 1179, 1167, 1141, 1105, 1041, 1005; ^1H NMR (400 MHz, $CDCl_3$) δ ppm 0.00 (s, 6 H, $Si(CH_3)_2$), 0.88 (s, 9 H, $SiC(CH_3)_3$), 1.03 - 1.15 (m, 1 H, CH_2), 1.22 (s, 6 H, 2 x CH_3), 1.23 (s, 6 H, 2 x CH_3), 1.28 - 1.46 (m, 4 H, CH_2), 1.65 - 1.79 (m, 1 H, CH_2), 2.35 - 2.44 (m, 1 H, C*H*C=CH_2), 2.49 (s, 3 H, Ar-CH_3), 3.49 (t, *J* = 6.5 Hz, 2 H, OCH_2), 3.66 (s, 3 H, OCH_3), 4.51 (br. s, 1 H, NH), 4.64 (d, *J* = 7.3 Hz, 1 H, CHN), 5.46 (d, *J* = 2.8 Hz, 1 H, C=CH_2), 5.91 (d, *J* = 3.3 Hz, 1 H, C=CH_2), 6.32 (d, *J* = 8.8 Hz, 2 H, ArCH), 6.62 (d, *J* = 8.8 Hz, 2 H, ArCH), 7.03 - 7.19 (m, 3 H, ArCH), 7.23 - 7.27 (m, 1 H, ArCH); ^{13}C NMR (101 MHz, $CDCl_3$) δ ppm -5.3 ($Si(CH_3)_2$), 18.3 (Si*C*(CH_3)$_3$), 19.6 (Ar-CH_3), 24.1 (CH_2), 24.7 (CH_3), 24.8 (CH_3), 26.0 (SiC(*C*H_3)$_3$), 30.1 (CH_2), 32.6 (CH_2), 54.2 (*C*HC=CH_2), 55.8 (OCH_3), 58.0 (CHN), 63.1 (OCH_2), 83.4 (OC), 113.9 (ArCH), 114.6 (ArCH), 126.1 (ArCH), 126.2 (ArCH), 126.8 (ArCH), 130.1 (ArCH), 132.8 (C=*C*H_2), 135.4 (ArC), 141.7 (ArC), 142.5 (ArC), 151.4 (ArC), (B*C*=CH_2 not observed); ^{11}B NMR (128 MHz, $CDCl_3$) δ ppm -3.3, 31.0; Specific rotation: $[\alpha]_D^{27}$ -15.5 (*c* 0.43, $CHCl_3$) for an enantiomerically enriched sample of 93:7 e.r.

Enantiomeric purity of **3n** was determined by HPLC analysis in comparison with authentic racemic material (93:7 e.r. shown; Lux 5 µm Amylose-1 column, 99.5:0.5 hexanes:*i*-PrOH, 0.2 mL/min, 30 °C, 254 nm).

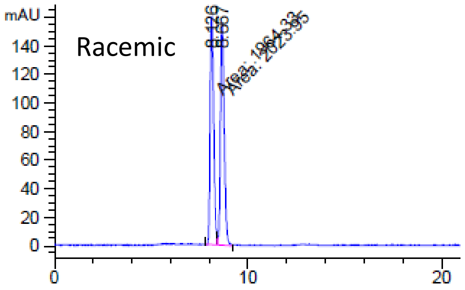

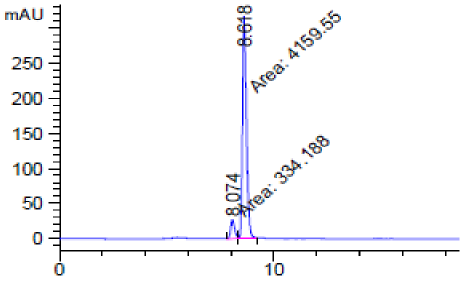

Peak #	RetTime [min]	Type	Width [min]	Area [mAU*s]	Height [mAU]	Area %
1	8.126	MF	0.2054	1964.33484	159.41408	49.2527
2	8.667	FM	0.2176	2023.94519	155.00246	50.7473

Peak #	RetTime [min]	Type	Width [min]	Area [mAU*s]	Height [mAU]	Area %
1	8.074	MF	0.2111	334.18808	26.39030	7.4367
2	8.618	FM	0.2187	4159.55078	316.92255	92.5633

**Peak #**	**Time (min)**	**Area (%)**	**Peak #**	**Time (min)**	**Area (%)**
1	8.126	49.2527	1	8.074	7.4367
2	8.667	50.7473	2	8.618	92.5633

(*R*)-5-((*R*)-((4-Methoxyphenyl)amino)(*o*-tolyl)methyl)-6-(4,4,5,5-tetramethyl-1,3,2-dioxaborolan-2-yl)hept-6-en-1-ol (3o)

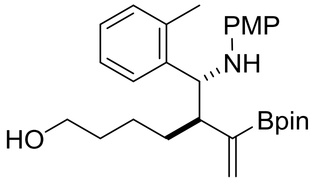

Prepared according to General Procedure 2, on a 0.258 mmol scale (91:9 d.r. of crude material), column chromatography (2-50% EtOAc in Hexanes) afforded the title compound as a yellow gum (43 mg, 0.093 mmol, 36%).

A pure sample of the major diastereoisomer was obtained; MS (ES$^+$) *m/z*: 466 (M+H$^+$). HRMS calcd for $C_{28}H_{41}NBO_4$: 466.3123. Found: 466.3125; ν_{max} (thin film/cm^{-1}): 3404, 2976, 2933, 1607, 1501, 1370, 1307, 1237, 1140, 1039; ^{1}H NMR (400 MHz, $CDCl_3$) δ ppm 1.07 - 1.16 (m, 2 H, CH_2), 1.21 – 1.24 (2 x s, 12 H, 6 x CH_3) 1.28 - 1.32 (m, 1 H, CH_2), 1.38 - 1.54 (m, 2 H, CH_2), 1.69 - 1.78 (m, 1 H, CH_2), 2.36 - 2.44 (m, 1 H, *C*HC=CH_2), 2.48 (s, 3 H, Ar-CH_3), 3.54 (t, *J* = 6.7 Hz, 2 H, C*H*$_2$OH), 3.66 (s, 3 H, OCH_3), 4.63 (d, *J* = 7.5 Hz, 1 H, CHN), 5.47 (d, *J* = 2.8 Hz, 1 H, C=CH_2), 5.91 (d, *J* = 3.3 Hz, 1 H, C=CH_2), 6.33 (d, *J* = 9.0 Hz, 2 H, ArCH), 6.62 (d, *J* = 9.0 Hz, 2 H, ArCH), 7.07 - 7.15 (m, 4 H, ArCH); ^{13}C NMR (101 MHz, $CDCl_3$) δ ppm 19.6 (Ar-CH_3), 24.0 (CH_2), 24.7 (CH_3), 24.8 (CH_3), 30.1 (CH_2), 32.5 (CH_2), 54.3 (CH), 55.8 (CHN), 58.1 (OCH_3), 62.8 (CH_2OH), 83.6 (OC), 114.0 (ArCH), 114.68 (ArCH), 126.12 (ArCH), 126.26 (ArCH), 126.84 (ArCH), 130.16 (ArCH), 132.93 (ArC), 135.42 (C=*C*H_2), 141.68 (ArC), 142.50 (ArC),

151.45 (ArC), (B*C*=CH_2 not observed); ^{11}B NMR (128 MHz, $CDCl_3$) δ ppm -15.0, 29.0; Specific rotation: $[\alpha]_D^{27}$ -32.6 (*c* 0.72, $CHCl_3$) for an enantiomerically enriched sample of 95:5 e.r.

Enantiomeric purity of **3o** was determined by HPLC analysis in comparison with authentic racemic material (95:5 e.r. shown; Lux 5 µm Amylose-1 column, 98:2 hexanes:*i*-PrOH, 0.5 mL/min, 20 °C, 254 nm).

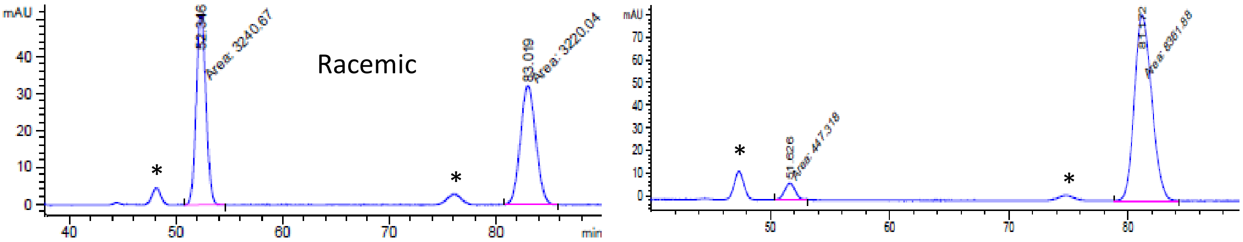

* = minor diastereoisomer

Peak #	RetTime [min]	Type	Width [min]	Area [mAU*s]	Height [mAU]	Area %
1	52.346	MM	1.0538	3240.67383	51.25257	50.1597
2	83.019	MM	1.6808	3220.03540	31.92910	49.8403

Peak #	RetTime [min]	Type	Width [min]	Area [mAU*s]	Height [mAU]	Area %
1	51.626	MM	1.0331	447.31760	7.21677	5.0778
2	81.172	MM	1.6938	8361.87988	82.28037	94.9222

**Peak #**	**Time (min)**	**Area (%)**	**Peak #**	**Time (min)**	**Area (%)**
1	52.346	50.1597	1	51.626	5.0778
2	83.019	49.8403	2	81.172	94.9222

4-Methoxy-*N*-((1*R*,2*R*)-2-phenethyl-3-(4,4,5,5-tetramethyl-1,3,2-dioxaborolan-2-yl)-1-(*o*-tolyl)but-3-en-1-yl)aniline (3p)

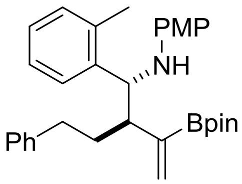

Prepared according to General Procedure 2, on a 0.258 mmol scale (94:6 d.r. from crude material), column chromatography (3% EtOAc in Hexanes) afforded the title compound as a yellow gum (63 mg, 0.126 mmol, 49%).

A pure sample of the major diastereoisomer was obtained; MS (ES^+) *m/z*: 498 ($M+H^+$). HRMS calcd for $C_{32}H_{41}NBO_3$: 498.3174. Found: 498.3157; ν_{max} (thin film/cm^{-1}): 3374, 2950, 1715, 1625, 1592, 1434, 1285, 1167, 1111; ^{1}H NMR (400 MHz, $CDCl_3$) δ ppm 1.22 – 1.29 (2 x s, 12 H, 4 x CH_3), 1.64 - 1.74 (m, 1 H, CH_2), 2.02 - 2.14 (m, 1 H, CH_2), 2.30 - 2.39 (m, 1 H, CH_2), 2.43 (s, 3 H, Ar-CH_3), 2.44 - 2.48

(m, 1 H, C*H*C=CH$_2$), 2.55 - 2.64 (m, 1 H, CH$_2$), 3.67 (s, 3 H, OCH$_3$), 4.51 (br. s, 1 H, NH), 4.66 (d, *J* = 7.5 Hz, 1 H, CHN), 5.50 (d, *J* = 3.3 Hz, 1 H, C=CH$_2$), 5.98 (d, *J* = 3.5 Hz, 1 H, C=CH$_2$), 6.33 (d, *J* = 8.8 Hz, 2 H, ArCH), 6.63 (d, *J* = 8.8 Hz, 2 H, ArCH), 6.99 - 7.26 (m, 9 H, ArCH); ^{13}C NMR (101 MHz, CDCl$_3$) δ ppm 19.5 (Ar-CH$_3$), 24.8 (CH$_3$), 24.9 (CH$_3$), 31.9 (CH$_2$), 34.0 (CH$_2$), 53.6 (*C*HC=CH$_2$), 55.8 (CHN), 58.3 (OCH$_3$), 83.6 (OC), 114.0 (ArCH), 114.7 (ArCH), 125.6 (ArCH), 126.1 (ArCH), 126.3 (ArCH), 126.9 (ArCH), 128.2 (ArCH), 128.4 (ArCH) 130.2 (ArCH), 133.4 (C=*C*H$_2$), 135.4 (ArC), 141.5 (ArC), 142.3 (ArC), 142.3 (ArC), 151.4 (ArC), (B*C*=CH$_2$ not observed); ^{11}B NMR (128 MHz, CDCl$_3$) δ ppm -3.0, 31.0; Specific rotation: $[\alpha]_D^{27}$ -20.6 (*c* 0.43, CHCl$_3$) for an enantiomerically enriched sample of 97:3 e.r.

Enantiomeric purity of **3p** was determined by HPLC analysis in comparison with authentic racemic material (97:3 e.r. shown; Chiralpak IA column, 99:1 hexanes:*i*-PrOH, 0.3 mL/min, 20 °C, 254 nm).

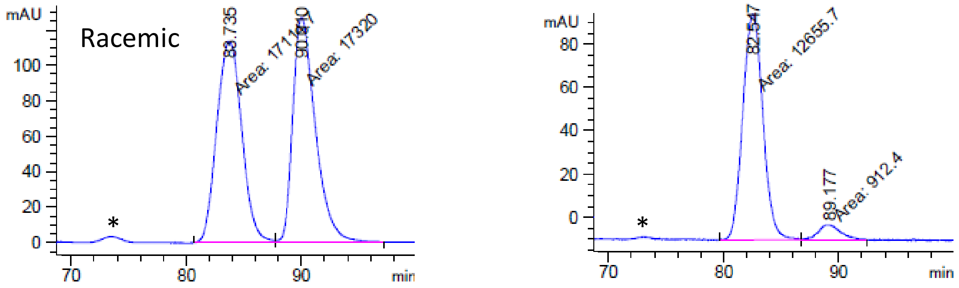

* = minor diastereoisomer

Peak #	RetTime [min]	Type	Width [min]	Area [mAU*s]	Height [mAU]	Area %
1	83.735	MF	2.5198	1.71147e4	113.19962	49.7019
2	90.010	FM	2.2834	1.73200e4	126.41716	50.2981

Peak #	RetTime [min]	Type	Width [min]	Area [mAU*s]	Height [mAU]	Area %
1	82.547	MF	2.0257	1.26557e4	104.12508	93.2754
2	89.177	FM	2.1999	912.39984	6.91228	6.7246

**Peak #**	**Time (min)**	**Area (%)**	**Peak #**	**Time (min)**	**Area (%)**
1	83.735	49.7019	1	82.547	93.2754
2	90.010	50.2981	2	89.177	6.7246

4-Methoxy-*N*-((1*R*,2*S*)-2-phenyl-3-(4,4,5,5-tetramethyl-1,3,2-dioxaborolan-2-yl)-1-(*o*-tolyl)but-3-en-1-yl)aniline (3q)

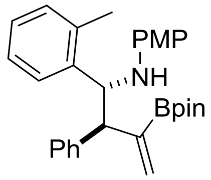

To a solution of CuI (2.5 mg, 0.013 mmol, 5 mol%) and NHC ligand **5** (7.3 mg, 0.014 mmol, 5.5 mol%) in THF (0.8 mL), was added *t*-BuOK (0.26 mL of a 1 M THF solution, 0.258 mmol, 1 equiv), and the reaction was stirred for 85 minutes at room temperature. B_2Pin_2 (72 mg, 0.284 mmol, 1.1 equiv) in THF (0.75 mL) was then added and the resulting mixture stirred for 50 minutes. The reaction mixture was cooled to -15 °C. A solution of propa-1,2-dien-1-ylbenzene (**2q**) (45 mg, 0.387 mmol, 1.5 equiv) and (*E*)-*N*-(4-methoxyphenyl)-1-(*o*-tolyl)methanimine (**1a**) (58 mg, 0.258 mmol, 1 equiv) in THF (1 mL) was then added dropwise at -15 °C with stirring overnight. The mixture was then filtered through a silica plug, concentrated *in vacuo* and the crude product mixture (83:17 d.r. of crude material) was purified by chromatography (3% EtOAc in hexanes) to afford the title compound as a yellow gum (38 mg, 0.081 mmol, 32%).

A pure sample of the major diastereoisomer was obtained; MS (ES^+) *m/z*: 470 ($M+H^+$). HRMS calcd for $C_{30}H_{36}NBO_3$: 470.2861. Found: 470.2860; ν_{max} (thin film/cm^{-1}): 3061, 3030, 2978, 2907, 1619, 1511, 1452, 1360, 1311, 1239, 1141, 1037; ^1H NMR (500 MHz, $CDCl_3$) δ ppm 1.02 (s, 6 H, 2 x CH_3), 1.09 (s, 6 H, 2 x CH_3), 2.50 (s, 3 H, Ar-CH_3), 3.67 (s, 3 H, OCH_3), 3.83 (br. s, 1 H, NH), 3.98 (d, *J* = 6.0 Hz, 1 H, C*H*C=CH_2), 5.12 (d, *J* = 6.1 Hz, 1 H, CHN), 5.72 (apparent s, 1 H, C=CH_2), 5.89 (apparent s, 1 H, C=CH_2), 6.30 (d, *J* = 8.7 Hz, 2 H, ArCH), 6.62 (d, *J* = 8.7 Hz, 2 H, ArCH), 6.98 - 7.07 (m, 2 H, ArCH), 7.07 - 7.12 (m, 3 H, ArCH), 7.14 (d, *J* = 7.3 Hz, 1 H, ArCH), 7.18 - 7.27 (m, 3 H, ArCH); ^{13}C NMR (126 MHz, $CDCl_3$) δ ppm 19.7 (Ar-CH_3), 24.4 (CH_3), 24.6 (CH_3), 55.3 (*C*HC=CH_2), 55.7 (OCH_3), 56.5 (CHN), 83.4 (OC), 114.4 (ArCH), 114.6 (ArCH), 125.7 (ArCH), 126.4 (ArCH), 126.6 (ArCH), 127.6 (ArCH), 127.9 (ArCH), 129.8 (ArCH), 130.1 (C=*C*H_2), 130.3 (ArCH), 135.3 (ArC), 139.6 (ArC), 140.8 (ArC), 141.6 (ArC), 151.7 (ArC), (B*C*=CH_2 not observed); ^{11}B NMR (128 MHz, $CDCl_3$) δ ppm 22.5, 29.5; Specific rotation: $[\alpha]_D^{27}$ -19.7 (*c* 0.70, $CHCl_3$) for an enantiomerically enriched sample of 81:19 e.r.

Enantiomeric purity of **3q** was determined by HPLC analysis in comparison with authentic racemic material (81:19 e.r. shown; Lux 5 µm Amylose-1 column, 99:1 hexanes:*i*-PrOH, 0.3 mL/min, 20 °C, 254 nm).

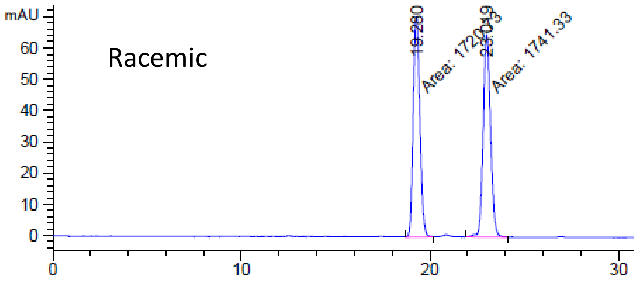

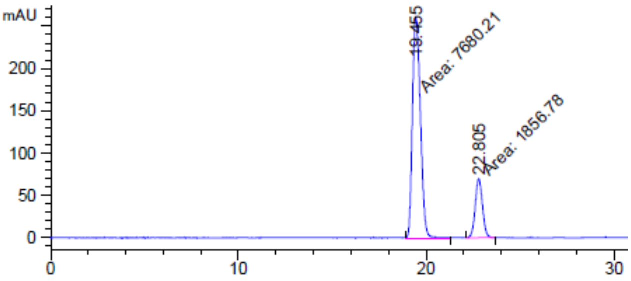

Peak #	RetTime [min]	Type	Width [min]	Area [mAU*s]	Height [mAU]	Area %
1	19.280	MM	0.4067	1720.72644	70.50817	49.7025
2	23.019	MM	0.4488	1741.32886	64.66728	50.2975

Peak #	RetTime [min]	Type	Width [min]	Area [mAU*s]	Height [mAU]	Area %
1	19.455	MM	0.4907	7680.20605	260.84500	80.5307
2	22.805	MM	0.4455	1856.78381	69.46232	19.4693

Peak #	Time (min)	Area (%)	Peak #	Time (min)	Area (%)
1	19.280	49.7025	1	19.455	80.5307
2	23.019	50.2975	2	22.805	19.4693

(*R*)-4-Methoxy-*N*-((1-(1-(4,4,5,5-tetramethyl-1,3,2-dioxaborolan-2-yl)vinyl)cyclohexyl)(o-tolyl)methyl)aniline (3r)

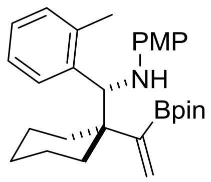

Prepared according to General Procedure 2, on a 0.258 mmol scale, column chromatography (2-4% EtOAc in Hexanes) afforded the title compound as a brown gum (87 mg, 0.189 mmol, 73%).
MS (ES^+) *m/z*: 462 ($M+H^+$). HRMS calcd for $C_{29}H_{41}NBO_3$: 462.3174. Found: 462.3153; ν_{max} (thin film/cm^{-1}): 3414, 2976, 2930, 2855, 1605, 1505, 1450, 1408, 1371, 1354, 1298, 1235, 1141, 1118, 1032; ^{1}H NMR (500 MHz, $CDCl_3$) δ ppm 0.98 - 1.05 (m, 1 H, CH_2), 1.10 (td, *J* = 13.1, 2.9 Hz, 1 H, CH_2), 1.27 (d, *J* = 2.1 Hz, 1 H, CH_2), 1.30 (s, 6 H, 2 x CH_3), 1.31 (s, 6 H, 2 x CH_3), 1.36 - 1.49 (m, 4 H, CH_2), 1.50 - 1.54 (m, 1 H, CH_2), 2.10 (d, *J* = 11.1 Hz, 1 H, CH_2), 2.41 (dd, *J* = 12.8, 1.8 Hz, 1 H, CH_2), 2.55 (s, 3 H, Ar-CH_3), 3.65 (s, 3 H, OCH_3), 4.43 (s, 1 H, CHN), 4.95 (br. s, 1 H, NH), 5.59 (apparent s., 1 H, C=CH_2), 6.16 (d, *J* = 2.0 Hz, 1 H, C=CH_2), 6.28 (d, *J* = 9.0 Hz, 2 H, ArCH), 6.61 (d, *J* = 8.9 Hz, 2 H, ArCH), 7.05 - 7.16 (m, 3 H, ArCH), 7.29 - 7.34 (m, 1 H, ArCH); ^{13}C NMR (126 MHz, $CDCl_3$) δ ppm 20.7 (Ar-CH_3), 22.0 (CH_2), 22.4 (CH_2), 24.6 (CH_3), 24.8 (CH_3), 26.5 (CH_2), 29.6 (CH_2), 33.9 (CH_2), 48.6 (*C*C=CH_2), 55.8 (OCH_3), 63.8 (CHN), 83.7 (OC), 113.5 (ArCH), 114.7 (ArCH), 125.0 (ArCH), 126.2 (ArCH), 129.5 (ArCH), 130.0 (ArCH), 132.3 (C=*C*H_2), 136.8 (ArC), 139.4 (ArC), 143.1 (ArC), 151.1 (ArC), (B*C*=CH_2 not observed); ^{11}B NMR (160 MHz, $CDCl_3$) δ ppm -3.8, 31.3; Specific rotation: $[\alpha]_D^{27}$ -20.6 (*c* 0.54, $CHCl_3$) for an enantiomerically enriched sample of 95:5 e.r.

Enantiomeric purity of **3r** was determined by HPLC analysis in comparison with authentic racemic material (95:5 e.r. shown; Chiralcel OD-H column, 99.5:0.5 hexanes:*i*-PrOH, 0.5 mL/min, 20 °C, 254 nm).

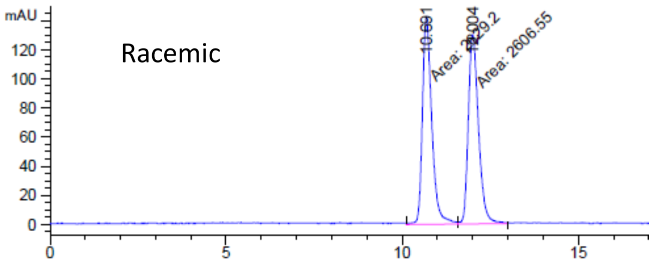

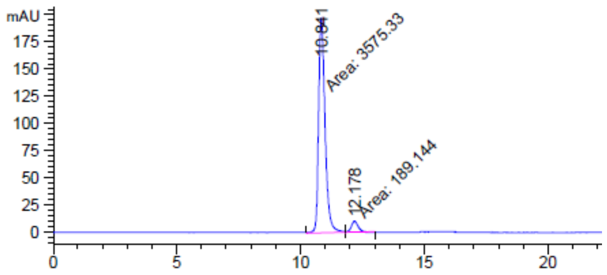

Peak #	RetTime [min]	Type	Width [min]	Area [mAU*s]	Height [mAU]	Area %
1	10.691	MF	0.3076	2629.20435	142.44136	50.2164
2	12.004	FM	0.3340	2606.54785	130.06255	49.7836

Peak #	RetTime [min]	Type	Width [min]	Area [mAU*s]	Height [mAU]	Area %
1	10.841	MF	0.3016	3575.32935	197.56100	94.9755
2	12.178	FM	0.3152	189.14421	10.00220	5.0245

**Peak #**	**Time (min)**	**Area (%)**	**Peak #**	**Time (min)**	**Area (%)**
1	10.691	50.2164	1	10.841	94.9755
2	12.004	49.7836	2	12.178	5.0245

(*R*)-*N*-(Furan-2-yl(1-(1-(4,4,5,5-tetramethyl-1,3,2-dioxaborolan-2-yl)vinyl)cyclohexyl)methyl)-4-methoxyaniline (3s)

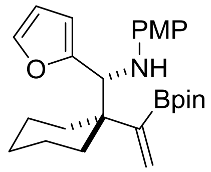

Prepared according to General Procedure 2, on a 0.258 mmol scale, column chromatography (5% EtOAc in Hexanes) afforded the title compound as a brown gum (50 mg, 0.114 mmol, 44%).

MS (ES$^+$) *m/z*: 438 (M+H$^+$). HRMS calcd for $C_{26}H_{37}NBO_4$: 438.2816. Found: 438.2824; ν_{max} (thin film/cm^{-1}): 3410, 2975, 2931, 2855, 1631, 1511, 1453, 1371, 1244, 1142, 1119, 1036; ^{1}H NMR (400 MHz, $CDCl_3$) δ ppm 1.10 - 1.22 (m, 1 H, CH_2), 1.30 (s, 12 H, CH_3), 1.32 - 1.56 (m, 7 H, CH_2), 2.06 - 2.13 (m, 1 H, CH_2), 2.20 - 2.31 (m, 1 H, CH_2), 3.69 (s, 3 H, OCH_3), 4.06 (s, 1 H, CHN), 4.87 (br. s, 1 H, NH), 5.50 (d, *J* = 2.5 Hz, 1 H, C=CH_2), 6.08 (d, *J* = 2.3 Hz, 1 H, C=CH_2), 6.28 - 6.31 (m, 1 H, ArCH), 6.40 - 6.46 (m, 2 H, ArCH), 6.65 - 6.70 (m, 2 H, ArCH), 7.22 - 7.24 (m, 1 H, ArCH), 7.29 (t, *J* = 1.6 Hz, 1 H, ArCH); ^{13}C NMR (101 MHz, $CDCl_3$) δ ppm 22.0 (CH_2), 22.5 (CH_2), 24.7 (CH_3), 24.7 (CH_3), 26.6 (CH_2), 32.0 (CH_2), 34.2 (CH_2), 46.4 (*C*C=CH_2), 55.8 (OCH_3), 61.2 (CHN), 83.7 (OC), 111.6 (ArCH), 113.7 (ArCH), 114.7 (ArCH), 122.0 (ArCH), 125.6 (C=*C*H_2), 132.2 (ArCH), 141.0 (ArC), 141.7 (ArC), 151.2 (ArC), (B*C*=CH_2 not observed); ^{11}B NMR (128 MHz, $CDCl_3$) δ ppm -2.94; Specific rotation: $[\alpha]_D^{27}$ -11.2 (*c* 0.45, $CHCl_3$) for an enantiomerically enriched sample of 97:3 e.r.

Enantiomeric purity of **3s** was determined by HPLC analysis in comparison with authentic racemic material (97:3 e.r. shown; Lux 5 µm Amylose-1 column, 99:1 hexanes:*i*-PrOH, 1.2 mL/min, 40 °C, 254 nm).

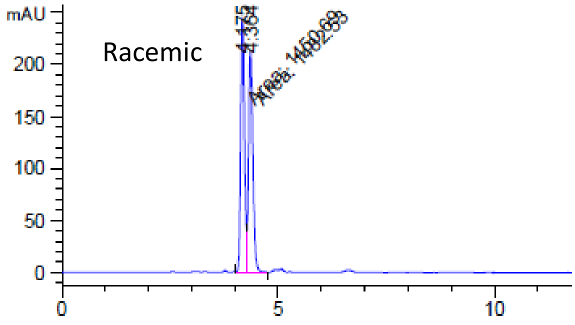

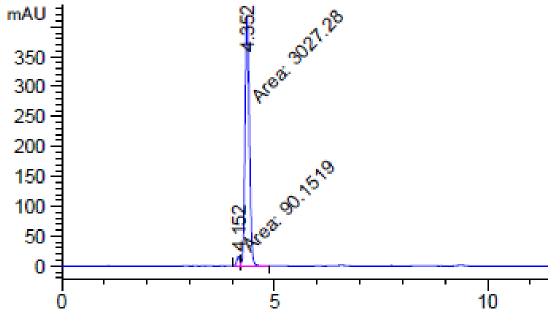

Peak #	RetTime [min]	Type	Width [min]	Area [mAU*s]	Height [mAU]	Area %
1	4.175	MF	0.0985	1450.68689	245.41954	49.4572
2	4.364	FM	0.1160	1482.52991	213.09689	50.5428

Peak #	RetTime [min]	Type	Width [min]	Area [mAU*s]	Height [mAU]	Area %
1	4.152	MF	0.0966	90.15189	15.55558	2.8919
2	4.352	FM	0.1208	3027.27515	417.54025	97.1081

**Peak #**	**Time (min)**	**Area (%)**	**Peak #**	**Time (min)**	**Area (%)**
1	4.175	49.4572	1	4.152	2.8919
2	4.364	50.5428	2	4.352	97.1081

(*R*)-4-Methoxy-*N*-((1-(1-(4,4,5,5-tetramethyl-1,3,2-dioxaborolan-2-yl)vinyl)cyclohexyl)(thiophen-2-yl)methyl)aniline (3t)

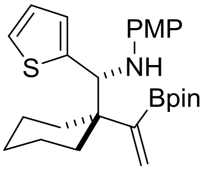

Prepared according to General Procedure 2, on a 0.258 mmol scale, column chromatography (3% EtOAc in Hexanes) afforded the title compound as a brown gum (40 mg, 0.0882 mmol, 34%).

MS (ES$^+$) *m/z*: 454 (M+H$^+$). HRMS calcd for $C_{26}H_{37}NBO_3S$: 454.2587. Found: 454.2568; ν_{max} (thin film/cm^{-1}): 3405, 2975, 2930, 2855, 1616, 1510, 1451, 1371, 1296, 1275, 1246, 1194, 1141, 1118, 1036; ^{1}H NMR (400 MHz, $CDCl_3$) δ ppm 1.08 - 1.22 (m, 1 H, CH_2), 1.31 (s, 6 H, 2 x CH_3), 1.31 (s, 6 H, 2 x CH_3), 1.35 - 1.58 (m, 7 H, CH_2), 2.13 - 2.22 (m, 1 H, CH_2), 2.24 - 2.33 (m, 1 H, CH_2), 3.68 (s, 3 H, OCH_3), 4.35 (d, *J* = 4.3 Hz, 1 H, CHN), 5.22 (d, *J* = 4.0 Hz, 1 H, NH), 5.52 (d, *J* = 2.3 Hz, 1 H, C=CH_2), 6.11 (d, *J* = 2.3 Hz, 1 H, C=CH_2), 6.40 - 6.47 (m, 2 H, ArCH), 6.63 - 6.70 (m, 2 H, ArCH), 6.91 - 6.96 (m, 2 H, ArCH), 7.13 (dd, *J* = 4.8, 1.5 Hz, 1 H, ArCH); ^{13}C NMR (101 MHz, $CDCl_3$) δ ppm 22.1 (CH_2), 22.5 (CH_2), 24.7 (CH_3), 24.7 (CH_3), 26.5 (CH_2), 31.7 (CH_2), 34.4 (CH_2), 46.7 (*C*C=CH_2), 55.7 (OCH_3), 65.4 (CHN), 83.8

(OC), 113.7 (ArCH), 114.6 (ArCH), 123.7 (ArCH), 125.3 (ArCH), 126.0 (C=*C*H_2), 132.8 (ArCH), 142.7 (ArC), 147.3 (ArC), 151.4 (ArC), (B*C*=CH_2 not observed); ^{11}B NMR (128 MHz, $CDCl_3$) δ ppm 6.4; Specific rotation: $[\alpha]_D^{27}$ -1.69 (*c* 0.71, $CHCl_3$) for an enantiomerically enriched sample of 96:4 e.r.

Enantiomeric purity of **3t** was determined by HPLC analysis in comparison with authentic racemic material (96:4 e.r. shown; Chiralcel OD-H column, 99.5:0.5 hexanes:*i*-PrOH, 0.5 mL/min, 20 °C, 254 nm).

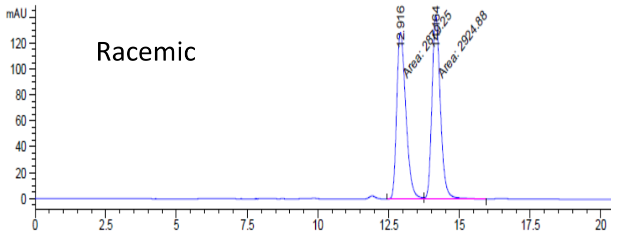

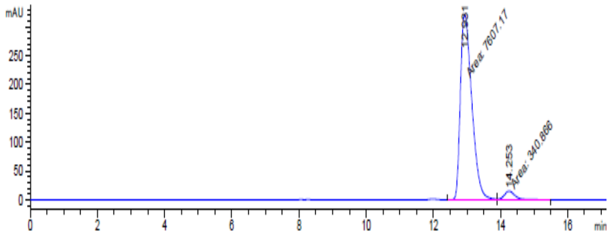

Peak #	RetTime [min]	Type	Width [min]	Area [mAU*s]	Height [mAU]	Area %
1	12.916	MF	0.3736	2879.25024	128.42960	49.6069
2	14.164	FM	0.3436	2924.88232	141.87025	50.3931

Peak #	RetTime [min]	Type	Width [min]	Area [mAU*s]	Height [mAU]	Area %
1	12.931	MF	0.3940	7607.16553	321.78543	95.7113
2	14.253	FM	0.3801	340.86597	14.94472	4.2887

**Peak #**	**Time (min)**	**Area (%)**	**Peak #**	**Time (min)**	**Area (%)**
1	12.916	49.6069	1	12.931	95.7113
2	14.164	50.3931	2	14.253	4.2887

(*R*)-*N*-(2,2-Dimethyl-3-(4,4,5,5-tetramethyl-1,3,2-dioxaborolan-2-yl)-1-(*o*-tolyl)but-3-en-1-yl)-4-methoxyaniline (3u)

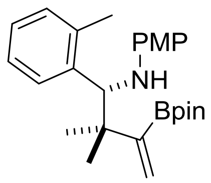

Prepared according to General Procedure 2, on a 0.258 mmol scale, column chromatography (3% EtOAc in Hexanes) afforded the title compound as a brown gum (98 mg, 0.232 mmol, 90%).

MS (ES$^+$) *m/z*: 422 (M+H$^+$). HRMS calcd for $C_{26}H_{37}NBO_3$: 422.2861. Found: 422.2850; ν_{max} (thin film/cm^{-1}): 2976, 1510, 1464, 1411, 1353, 1302, 1235, 1218, 1145, 1124, 1101, 1039; ^{1}H NMR (500 MHz, $CDCl_3$) δ ppm 1.06 (s, 3 H, CH_3), 1.09 (s, 3 H, CH_3), 1.16 (s, 12 H, 4 x CH_3), 2.57 (s, 3 H, Ar-CH_3), 3.65 (s, 3 H, OCH_3), 3.97 (br. s, 1 H, NH), 4.92 (s, 1 H, CHN), 5.75 (d, *J* = 2.4 Hz, 1 H, C=CH_2), 5.97 (d, *J* = 2.6 Hz, 1 H, C=CH_2), 6.30 (d, *J* = 8.9 Hz, 2 H, ArCH), 6.59 (d, *J* = 8.9 Hz, 2 H, ArCH), 7.10 - 7.14 (m, 2 H,

ArCH), 7.14 - 7.18 (m, 1 H, ArCH), 7.40 - 7.42 (m, 1 H, ArCH); ^{13}C NMR (126 MHz, $CDCl_3$) δ ppm 20.4 (Ar-CH_3), 20.6 (CH_3), 24.7 (CH_3), 24.7 (CH_3), 26.2 (CH_3), 44.8 (*C*C=CH_2), 55.8 (OCH_3), 60.3 (CHN), 83.3 (OC), 114.3 (ArCH), 114.5 (ArCH), 125.3 (ArCH), 126.3 (ArCH), 127.7 (C=*C*H_2), 129.1 (ArCH), 130.2 (ArCH), 137.0 (ArC), 139.3 (ArC), 142.8 (ArC), 151.7 (ArC), (B*C*=CH_2 not observed); ^{11}B NMR (160 MHz, $CDCl_3$) δ ppm -2.63; Specific rotation: $[\alpha]_D^{27}$ -55.3 (*c* 1.06, $CHCl_3$) for an enantiomerically enriched sample of 94:6 e.r.

Enantiomeric purity of **3u** was determined by HPLC analysis in comparison with authentic racemic material (94:6 e.r. shown; Chiralcel OD-H column, 99.5:0.5 hexanes:*i*-PrOH, 0.5 mL/min, 20 °C, 254 nm).

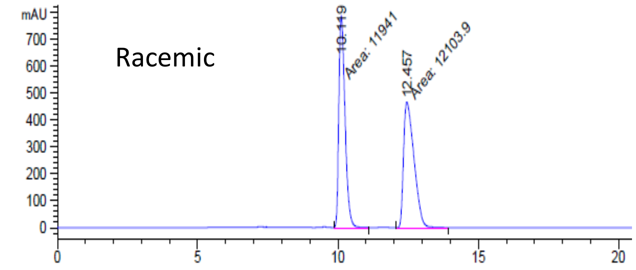

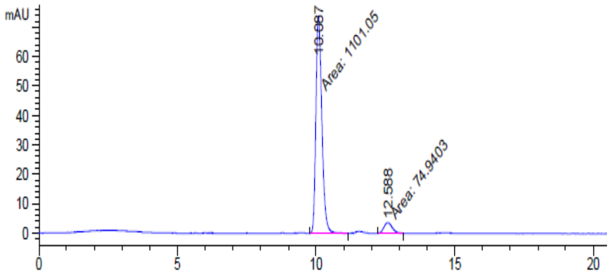

Peak #	RetTime [min]	Type	Width [min]	Area [mAU*s]	Height [mAU]	Area %
1	10.119	MM	0.2539	1.19410e4	783.94434	49.6613
2	12.457	MM	0.4331	1.21039e4	465.74948	50.3387

Peak #	RetTime [min]	Type	Width [min]	Area [mAU*s]	Height [mAU]	Area %
1	10.087	MM	0.2485	1101.04968	73.84858	93.6275
2	12.588	MM	0.3420	74.94032	3.65226	6.3725

**Peak #**	**Time (min)**	**Area (%)**	**Peak #**	**Time (min)**	**Area (%)**
1	10.119	49.6613	1	10.087	93.6275
2	12.457	50.3387	2	12.588	6.3725

(*R*)-*N*-(1-(Furan-2-yl)-2,2-dimethyl-3-(4,4,5,5-tetramethyl-1,3,2-dioxaborolan-2-yl)but-3-en-1-yl)-4-methoxyaniline (3v)

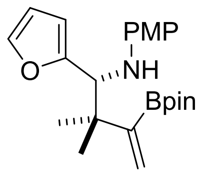

Prepared according to General Procedure 2, on a 0.258 mmol scale, column chromatography (3% EtOAc in Hexanes) afforded the title compound as a brown gum (59 mg, 0.148 mmol, 57%).

MS (ES^+) *m/z*: 398 ($M+H^+$). HRMS calcd for $C_{23}H_{33}NBO_4$: 398.2503. Found: 398.2505; ν_{max} (thin film/cm^{-1}): 2975, 1512, 1411, 1354, 1300, 1237, 1144, 1118, 1024; ^{1}H NMR (500 MHz, $CDCl_3$) δ ppm 1.14 (s, 3 H, CH_3), 1.16 (s, 3 H, CH_3), 1.17 (s, 6 H, 2 x CH_3), 1.19 (s, 6 H, 2 x CH_3), 3.69 (s, 3 H, OCH_3),

3.94 (br. s, 1 H, NH), 4.69 (s, 1 H, CHN), 5.67 (d, J = 1.7 Hz, 1 H, C=CH_2), 5.88 (d, J = 1.8 Hz, 1 H, C=CH_2), 6.13 (d, J = 2.9 Hz, 1 H, ArCH), 6.27 (t, J = 1.8 Hz, 1 H, ArCH), 6.48 (d, J = 8.5 Hz, 2 H, ArCH), 6.67 (d, J = 8.5 Hz, 2 H, ArCH), 7.33 (apparent s, 1 H, ArCH); ^{13}C NMR (126 MHz, $CDCl_3$) δ ppm 22.2 (CH_3), 24.6 (CH_3), 24.8 (CH_3), 25.8 (CH_3), 43.4 (*C*C=CH_2), 55.7 (OCH_3), 60.4 (CHN), 83.3 (OC), 107.7 (ArCH), 110.0 (ArCH), 114.5 (ArCH), 114.8 (ArCH), 127.3 (C=*C*H_2), 141.0 (ArCH), 142.1 (ArC), 152.0 (ArC), 155.5 (ArC), (B*C*=CH_2 not observed); ^{11}B NMR (160 MHz, $CDCl_3$) δ ppm -3.5, 30.4; Specific rotation: $[\alpha]_D^{27}$ +26.3 (*c* 0.33, $CHCl_3$) for an enantiomerically enriched sample of 90:10 e.r.

Enantiomeric purity of **3v** was determined by HPLC analysis in comparison with authentic racemic material (90:10 e.r. shown; Chiralcel OD-H column, 99.5:0.5 hexanes:*i*-PrOH, 0.5 mL/min, 20 °C, 254 nm).

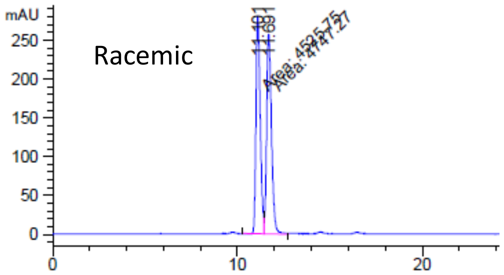

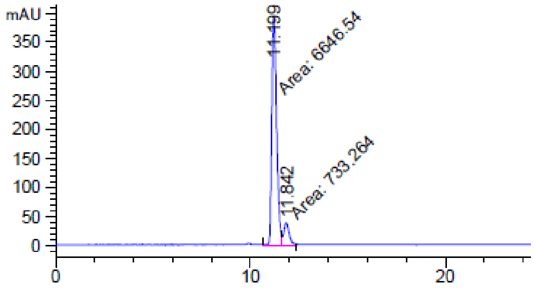

Peak #	RetTime [min]	Type	Width [min]	Area [mAU*s]	Height [mAU]	Area %
1	11.101	MF	0.2698	4525.74805	279.52780	48.8055
2	11.691	FM	0.3072	4747.27344	257.57852	51.1945

Peak #	RetTime [min]	Type	Width [min]	Area [mAU*s]	Height [mAU]	Area %
1	11.199	MF	0.2810	6646.53516	394.14954	90.0639
2	11.842	FM	0.3163	733.26385	38.63419	9.9361

Peak #	Time (min)	Area (%)	Peak #	Time (min)	Area (%)
1	11.101	48.8055	1	11.199	90.0639
2	11.691	51.1945	2	11.842	9.9361

(*R*)-*N*-(2,2-Dimethyl-3-(4,4,5,5-tetramethyl-1,3,2-dioxaborolan-2-yl)-1-(thiophen-2-yl)but-3-en-1-yl)-4-methoxyaniline (3w)

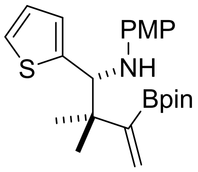

Prepared according to General Procedure 2, on a 0.258 mmol scale, column chromatography (5% EtOAc in Hexanes) afforded the title compound as a brown gum (45 mg, 0.109 mmol, 42%).

MS (ES^+) *m/z*: 414 ($M+H^+$). HRMS calcd for $C_{23}H_{33}NBO_3S$: 414.2269. Found: 414.2259; ν_{max} (thin film/cm^{-1}): 3389, 2976, 2359, 1602, 1503, 1465, 1411, 1372, 1353, 1300, 1235, 1144, 1105, 1039; ^{1}H NMR (400 MHz, $CDCl_3$) δ ppm 1.03 (s, 3 H, CH_3), 1.05 (s, 6 H, 2 x CH_3), 1.08 (s, 6 H, 2 x CH_3), 1.14 (s, 3 H, CH_3), 3.60 (s, 3 H, OCH_3), 4.01 (d, *J* = 2.8 Hz, 1 H, NH), 4.75 (d, *J* = 3.3 Hz, 1 H, CHN), 5.64 (d, *J* = 2.5 Hz, 1 H, C=CH_2), 5.87 (d, *J* = 2.5 Hz, 1 H, C=CH_2), 6.38 (d, *J* = 9.0 Hz, 2 H, ArCH), 6.57 (d, *J* = 9.0 Hz, 2 H, ArCH), 6.86 - 6.92 (m, 2 H, ArCH), 7.08 (dd, *J* = 4.8, 1.5 Hz, 1 H, ArCH); ^{13}C NMR (101 MHz, $CDCl_3$) δ ppm 21.7 (CH_3), 24.6 (CH_3), 24.7 (CH_3), 26.3 (CH_3), 43.4 (*C*C=CH_2), 55.7 (OCH_3), 62.2 (CHN), 83.4 (OC), 114.5 (ArCH), 114.6 (ArCH), 123.8 (ArCH), 125.1 (ArCH), 126.2 (ArCH), 128.3 (C=*C*H_2), 142.3 (ArC), 147.4 (ArC), 152.0 (ArC), (B*C*=CH_2 not observed); ^{11}B NMR (128 MHz, $CDCl_3$) δ ppm 30.3; Specific rotation: $[\alpha]_D^{27}$ -37.4 (*c* 0.60, $CHCl_3$) for an enantiomerically enriched sample of 93:7 e.r.

Enantiomeric purity of **3w** was determined by HPLC analysis in comparison with authentic racemic material (93:7 e.r. shown; Chiralcel OD-H column, 99:1 hexanes:*i*-PrOH, 0.3 mL/min, 20 °C, 254 nm).

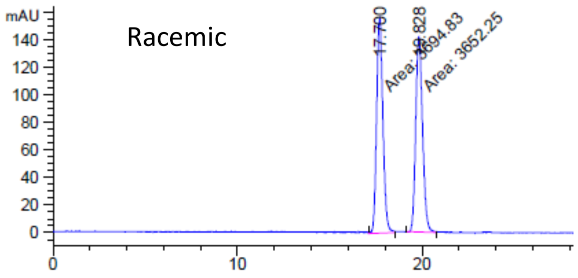

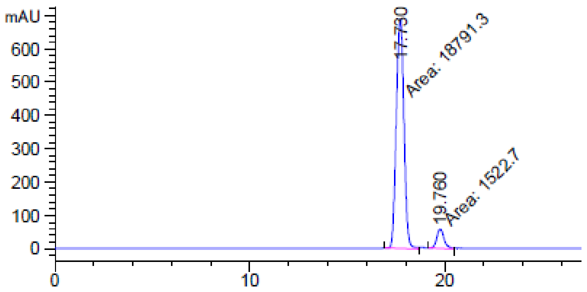

Peak #	RetTime [min]	Type	Width [min]	Area [mAU*s]	Height [mAU]	Area %
1	17.700	MM	0.3939	3694.83301	156.32742	50.2898
2	19.828	MM	0.4298	3652.25024	141.62860	49.7102

Peak #	RetTime [min]	Type	Width [min]	Area [mAU*s]	Height [mAU]	Area %
1	17.730	MM	0.4536	1.87913e4	690.40002	92.5042
2	19.760	MM	0.4383	1522.70288	57.89712	7.4958

Peak #	Time (min)	Area (%)	Peak #	Time (min)	Area (%)
1	17.700	50.2898	1	17.730	92.5042
2	19.828	49.7102	2	19.760	7.4958

Methyl 4-(((1*R*,2*R*)-2-cyclohexyl-3-(4,4,5,5-tetramethyl-1,3,2-dioxaborolan-2-yl)-1-(*o*-tolyl)but-3-en-1-yl)amino)benzoate (3x)

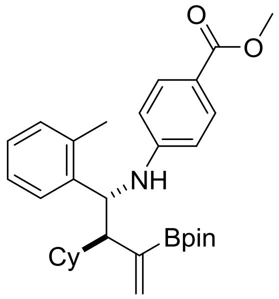

Prepared according to General Procedure 2, on a 0.258 mmol scale (93:7 d.r. from crude material), column chromatography (5-10% EtOAc in Hexanes) afforded the title compound as a yellow gum (112 mg, 0.223 mmol, 86%).

A pure sample of the major diastereoisomer was obtained; MS (ES^+) *m/z*: 504 ($M+H^+$). HRMS calcd for $C_{31}H_{43}NBO_4$: 504.3280. Found: 504.3271; ν_{max} (thin film/cm^{-1}): 3397, 2977, 2927, 2851, 1602, 1531, 1358, 1311, 1276, 1216, 1141, 1090; ^1H NMR (400 MHz, $CDCl_3$) δ ppm 0.79-0.84 (m, 1 H, CH_2), 0.98 - 1.10 (m, 1 H, CH_2), 1.13 - 1.24 (m, 3 H, CH_2), 1.34 (s, 6 H, 2 x CH_3), 1.38 (s, 6 H, 2 x CH_3), 1.64 - 1.78 (m, 4 H, CH_2), 1.89 - 2.02 (m, 2 H, CH_2), 2.25 (dd, *J* = 10.2, 4.1 Hz, 1 H, C*H*C=CH_2), 2.48 (s, 3 H, Ar-CH_3). 3.81 (s, 3 H, CO_2CH_3), 4.89 (d, *J* = 3.5 Hz, 1 H, C=CH_2), 5.02 (br. s, 1 H, CHN), 5.72 (d, *J* = 3.5 Hz, 1 H, C=CH_2), 6.33 (d, *J* = 8.8 Hz, 2 H, ArCH), 6.62 (br. s, 1 H, NH), 6.90 - 6.95 (m, 1 H, ArCH), 6.99 (td, *J* = 7.4, 1.3 Hz, 1 H, ArCH), 7.06 (td, *J* = 7.3, 1.5 Hz, 1 H, ArCH), 7.11 - 7.16 (m, 1 H, ArCH), 7.75 (d, *J* = 8.8 Hz, 2 H, 2 x ArCH); ^{13}C NMR (101 MHz, $CDCl_3$) δ ppm 19.1 (Ar-CH_3), 24.4 (CH_3), 25.1 (CH_3), 26.3 (CH_2), 26.4 (CH_2), 26.5 (CH_2), 30.9 (CH_2), 33.1 (CH_2), 37.0 (CH), 51.4 (CO_2*C*H_3), 53.7 (CHN), 58.0 (*C*HC=CH_2), 84.1 (OC), 111.2 (ArCH), 117.0 (ArC), 125.5 (ArCH), 126.3 (ArCH), 127.5 (ArCH), 130.3 (ArCH), 131.4 (ArCH), 134.2 (ArC), 135.9 (C=*C*H_2), 139.8 (ArC), 151.8 (ArC), 167.4 (C=O), (B*C*=CH_2 not observed); ^{11}B NMR (128 MHz, $CDCl_3$) δ ppm 30.0; Specific rotation: $[\alpha]_D^{27}$ -27.2 (*c* 1.64, $CHCl_3$) for an enantiomerically enriched sample of 97:3 e.r.

Enantiomeric purity of **3x** was determined by HPLC analysis in comparison with authentic racemic material (97:3 e.r. shown; Chiralpak IA column, 99:1 hexanes:*i*-PrOH, 0.3 mL/min, 20 °C, 254 nm).

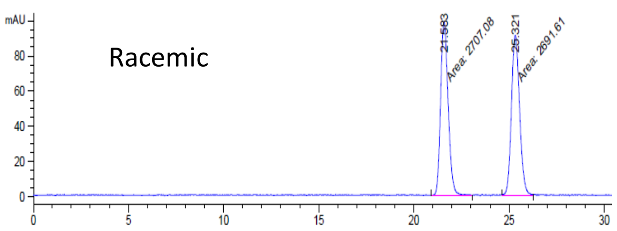

Peak #	RetTime [min]	Type	Width [min]	Area [mAU*s]	Height [mAU]	Area %
1	21.583	MM	0.4572	2707.08179	98.67846	50.1433
2	25.321	MM	0.4942	2691.61182	90.77528	49.8567

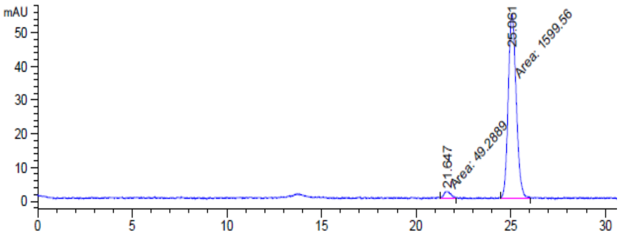

Peak #	RetTime [min]	Type	Width [min]	Area [mAU*s]	Height [mAU]	Area %
1	21.647	MM	0.4278	49.28886	1.92009	2.9893
2	25.061	MM	0.4884	1599.55957	54.58958	97.0107

**Peak #**	**Time (min)**	**Area (%)**	**Peak #**	**Time (min)**	**Area (%)**
1	21.583	50.1433	1	21.647	2.9893
2	25.321	49.8567	2	25.061	97.0107

***N*-((1*R*,2*R*)-2-Cyclohexyl-3-(4,4,5,5-tetramethyl-1,3,2-dioxaborolan-2-yl)-1-(*o*-tolyl)but-3-en-1-yl)quinolin-5-amine (3y)**

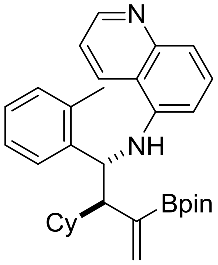

Prepared according to General Procedure 2, on a 0.258 mmol scale (94:6 d.r. of crude material), column chromatography (10% EtOAc in Hexanes) afforded the title compound as a brown gum (87 mg, 0.175 mmol, 68%).

A pure sample of the major diastereoisomer was obtained; MS (ES^+) *m/z*: 497 ($M+H^+$). HRMS calcd for $C_{32}H_{42}N_2BO_2$: 497.3334. Found: 497.3346; ν_{max} (thin film/cm^{-1}): 3421, 2976, 2925, 2850, 2244, 1587, 1535, 1415, 1371, 1330, 1140; ^{1}H NMR (400 MHz, $CDCl_3$) δ ppm 0.80 - 0.92 (m, 1 H, CH_2), 1.16 - 1.29 (m, 3 H, CH_2), 1.38 (s, 6 H, 2 x CH_3), 1.41 (s, 6 H, 2 x CH_3), 1.60 - 1.77 (m, 5 H, CH_2), 1.93 - 2.08 (m, 2 H, CH_2 + CH), 2.37 (dd, *J* = 10.5, 4.3 Hz, 1 H, C*H*C=CH_2), 2.53 (s, 3 H, Ar-CH_3), 4.89 (d, *J* = 3.3 Hz, 1 H, C=CH_2), 5.10 (t, *J* = 5.1 Hz, 1 H, CHN), 5.73 (d, *J* = 3.5 Hz, 1 H, C=CH_2), 6.08 (dd, *J* = 5.7, 3.1 Hz, 1 H, ArCH), 6.50 (d, *J* = 5.8 Hz, 1 H, NH), 6.93 - 7.01 (m, 2 H, ArCH), 7.06 (m, 1 H, ArCH), 7.16 (d, *J* = 7.5 Hz, 1 H, ArCH), 7.30 - 7.36 (m, 3 H, ArCH), 8.64 (dd, *J* = 8.5, 1.5 Hz, 1 H, ArCH), 8.88 (dd, *J* = 4.3, 1.5 Hz, 1 H, ArCH); ^{13}C NMR (101 MHz, $CDCl_3$) δ ppm 19.1 (Ar-CH_3), 24.3 (CH_3), 25.1 (CH_3), 26.4 (CH_2), 26.4 (CH_2), 26.5 (CH_2), 30.7 (CH_2), 33.3 (CH_2), 37.3 (CH), 54.8 (CHN), 57.7 (*C*HC=CH_2), 84.2 (OC), 104.6 (ArCH), 116.8 (ArCH), 118.4 (ArCH), 118.6 (ArC), 125.5 (ArCH), 126.3 (ArCH), 127.3 (ArCH), 129.8 (ArCH), 130.4 (ArCH), 130.8 (ArCH), 134.2 (ArC), 136.2 (C=*C*H_2), 134.0 (ArC), 143.6 (ArC), 149.3 (ArCH), 149.7 (ArC-N), (*C*=CH_2 not observed); ^{11}B NMR (128 MHz, $CDCl_3$) δ ppm -6.0, 30.0; Specific rotation: $[\alpha]_D^{27}$ -129 (*c* 0.33, $CHCl_3$) for an enantiomerically enriched sample of 96:4 e.r.

Enantiomeric purity of **3y** was determined by HPLC analysis in comparison with authentic racemic material (96:4 e.r. shown; Lux 5 µm Amylose-1 column, 95:5 hexanes:*i*-PrOH (+1% diethylamine), 0.5 mL/min, 20 °C, 254 nm).

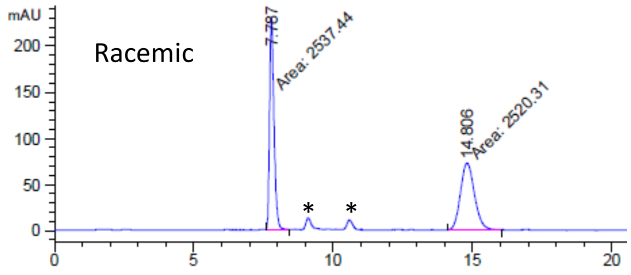

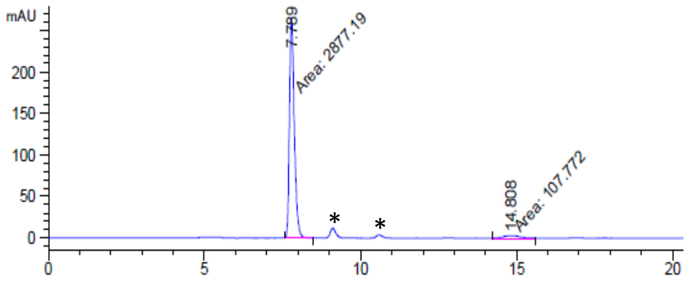

* = minor diastereoisomer

Peak #	RetTime [min]	Type	Width [min]	Area [mAU*s]	Height [mAU]	Area %
1	7.787	MM	0.1834	2537.44141	230.55040	50.1693
2	14.806	MM	0.5804	2520.31274	72.37698	49.8307

Peak #	RetTime [min]	Type	Width [min]	Area [mAU*s]	Height [mAU]	Area %
1	7.789	MM	0.1805	2877.18848	265.65210	96.3895
2	14.808	MM	0.5766	107.77163	3.11503	3.6105

**Peak #**	**Time (min)**	**Area (%)**	**Peak #**	**Time (min)**	**Area (%)**
1	7.787	50.1693	1	7.789	96.3895
2	14.806	49.8307	2	14.808	3.6105

***N*-((1*R*,2*R*)-2-Cyclohexyl-3-(4,4,5,5-tetramethyl-1,3,2-dioxaborolan-2-yl)-1-(*o*-tolyl)but-3-en-1-yl)-4-morpholinoaniline (3z)**

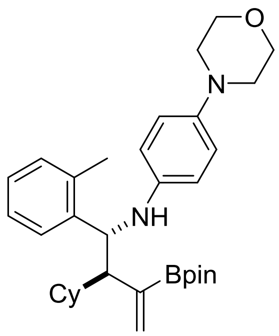

Prepared according to General Procedure 2, on a 0.258 mmol scale (>95:5 d.r. of crude material), column chromatography (10-20% EtOAc in Hexanes) afforded the title compound as a yellow/green gum (125 mg, 0.235 mmol, 91%).

A pure sample of the major diastereoisomer was obtained; MS (ES^+) *m/z*: 531 ($M+H^+$). HRMS calcd for $C_{33}H_{48}N_2BO_3$: 531.3753. Found: 531.3756; ν_{max} (thin film/cm^{-1}): 3408, 2924, 2851, 1514, 1421, 1371, 1299, 1218, 1140, 1069; ^1H NMR (400 MHz, $CDCl_3$) δ ppm 0.73 - 0.85 (m, 1 H, CH_2), 0.97 - 1.08 (m, 1 H, CH_2), 1.13 - 1.24 (m, 2 H, CH_2), 1.30 – 1.38 (2 x s, 12 H, 6 x CH_3), 1.58 - 1.74 (m, 5 H, CH_2), 1.92 - 2.07 (m, 2 H, CH_2 + CH), 2.21 (dd, *J* = 10.0, 3.8 Hz, 1 H, C*H*C=CH_2), 2.47 (s, 3 H, Ar-CH_3), 2.88 - 2.98 (m, 4 H, CH_2), 3.80 - 3.85 (m, 4 H, CH_2), 4.87 - 4.93 (m, 2 H, C=CH_2 + CHN), 5.58 (br. s, 1 H, NH), 5.71 (d, *J* = 3.5 Hz, 1 H, C=CH_2), 6.33 (d, *J* = 8.8 Hz, 2 H, ArCH), 6.72 (d, *J* = 8.8 Hz, 2 H, ArCH), 6.97 -

7.07 (m, 3 H, ArCH), 7.09 - 7.14 (m, 1 H, ArCH); ^{13}C NMR (101 MHz, $CDCl_3$) δ ppm 19.1 (Ar-CH_3), 24.4 (CH_3), 25.1 (CH_3), 26.4 (CH_2), 26.5 (CH_2), 26.6 (CH_2), 31.0 (CH_2), 33.1 (CH_2), 37.0 (CH), 51.5 (CH_2), 58.2 (CH_2), 67.2 (*C*HC=CH_2), 83.7 (OC), 112.9 (ArCH), 118.5 (ArCH), 125.4 (ArCH), 125.9 (ArCH), 127.9 (ArCH), 130.1 (ArCH), 134.2 (ArC), 134.9 (C=*C*H_2), 141.0 (ArC), 142.1 (ArC), 143.0 (ArC), (*C*=CH_2 not observed); ^{11}B NMR (128 MHz, $CDCl_3$) δ ppm -1.0, 33.0; Specific rotation: $[\alpha]_D^{27}$ -31.1 (*c* 1.19, $CHCl_3$) for an enantiomerically enriched sample of >99:1 e.r.

Enantiomeric purity of **3z** was determined by HPLC analysis in comparison with authentic racemic material (>99:1 e.r. shown; Lux 5 µm Amylose-1 column, 98:2 hexanes:*i*-PrOH, 0.7 mL/min, 20 °C, 254 nm).

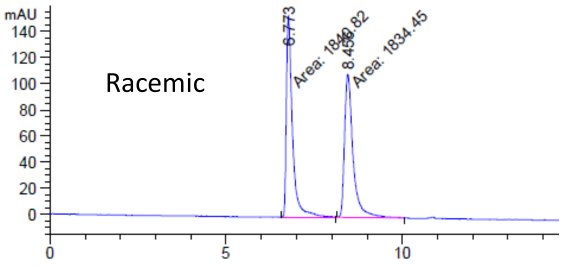

Peak #	RetTime [min]	Type	Width [min]	Area [mAU*s]	Height [mAU]	Area %
1	6.773	MM	0.1998	1840.81885	153.54163	50.0867
2	8.456	MM	0.2794	1834.44800	109.41995	49.9133

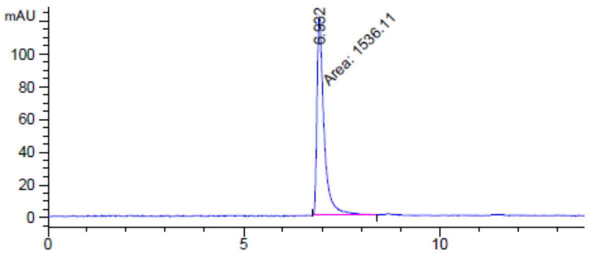

Peak #	RetTime [min]	Type	Width [min]	Area [mAU*s]	Height [mAU]	Area %
1	6.932	MM	0.2117	1536.11475	120.93266	100.0000

Peak #	Time (min)	Area (%)	Peak #	Time (min)	Area (%)
1	6.773	50.0867	1	6.932	100.0000
2	8.456	49.9133	2	-	-

***N*-((1*R*,2*R*)-2-Cyclohexyl-3-(4,4,5,5-tetramethyl-1,3,2-dioxaborolan-2-yl)-1-(*o*-tolyl)but-3-en-1-yl)-4-(4,4,5,5-tetramethyl-1,3,2-dioxaborolan-2-yl)aniline (3aa)**

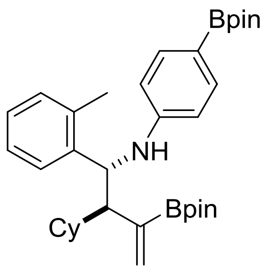

Prepared according to General Procedure 2, on a 0.258 mmol scale (94:6 d.r. of crude material), column chromatography (3% EtOAc in Hexanes) afforded the title compound as a yellow foam (102 mg, 0.179 mmol, 69%).

A pure sample of the major diastereoisomer was obtained; MS (ES$^+$) *m/z*: 572 (M+H$^+$). HRMS calcd for $C_{35}H_{52}NB_2O_4$: 572.4077. Found: 572.4079; ν_{max} (thin film/cm^{-1}): 3397, 2977, 2927, 2851, 1602, 1358, 1311, 1275, 1216, 1141, 1090; ^{1}H NMR (400 MHz, $CDCl_3$) δ ppm 0.73 - 0.84 (m, 1 H, CH_2), 0.94 - 1.05 (m, 1 H, CH_2), 1.09 - 1.23 (m, 3 H, CH_2 + CH), 1.23 - 1.39 (m, 24 H, 8 x CH_3), 1.60 - 1.73 (m, 4 H, CH_2), 1.87 - 2.03 (m, 2 H, CH_2), 2.23 (dd, *J* = 10.3, 4.0 Hz, 1 H, C*H*C=CH_2), 2.45 (s, 3 H, OCH_3), 4.88 (d, *J* = 3.5 Hz, 1 H, C=CH_2), 4.95 - 5.01 (m, 1 H, CHN), 5.68 (d, *J* = 3.5 Hz, 1 H, C=CH_2), 6.24 - 6.28 (apparent m, 1 H, NH), 6.33 (d, *J* = 8.8 Hz, 2 H, ArCH), 6.95 (d, *J* = 3.8 Hz, 2 H, ArCH), 7.01 (m, 1 H, ArCH), 7.07 - 7.12 (m, 1 H, ArCH), 7.50 (d, *J* = 8.5 Hz, 2 H, ArCH); ^{13}C NMR (101 MHz, $CDCl_3$) δ ppm 19.1 (Ar-CH_3), 24.4 (CH_3), 24.8 (CH_3), 24.8 (CH_3), 25.0 (CH_3), 26.4 (CH_2), 26.5 (CH_2), 26.6 (CH_2), 30.9 (CH_2), 33.1 (CH_2), 36.9 (CH), 53.5 (CHN), 58.0 (*C*HC=CH_2), 83.0 (OC), 83.9 (OC), 111.5 (ArCH), 125.5 (ArCH), 126.0 (ArCH), 127.6 (ArCH), 130.1 (ArCH), 134.2 (ArC), 135.4 (C=*C*H_2), 136.2 (ArCH), 140.3 (ArC), 150.5 (ArC), (B*C*=CH_2 and ArC-Bpin not observed); ^{11}B NMR (128 MHz, $CDCl_3$) δ ppm -17.0, 30.0; Specific rotation: $[\alpha]_D^{27}$ -23.3 (*c* 1.45, $CHCl_3$) for an enantiomerically enriched sample of 98:2 e.r.

Enantiomeric purity of **3aa** was determined by HPLC analysis in comparison with authentic racemic material (98:2 e.r. shown; Lux 5 µm Amylose-1 column, 99:1 hexanes:*i*-PrOH, 0.3 mL/min, 20 °C, 254 nm).

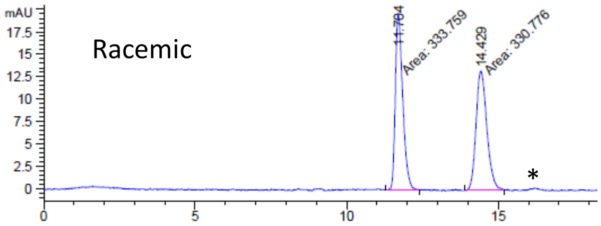

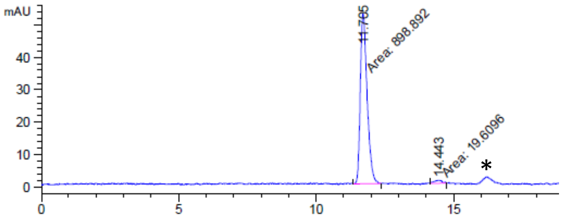

* = minor diastereoisomer

Peak #	RetTime [min]	Type	Width [min]	Area [mAU*s]	Height [mAU]	Area %
1	11.704	MM	0.2838	333.75864	19.60228	50.2244
2	14.429	MM	0.4140	330.77625	13.31750	49.7756

Peak #	RetTime [min]	Type	Width [min]	Area [mAU*s]	Height [mAU]	Area %
1	11.705	MM	0.2857	898.89154	52.43394	97.8650
2	14.443	MM	0.3340	19.60959	9.78414e-1	2.1350

**Peak #**	**Time (min)**	**Area (%)**	**Peak #**	**Time (min)**	**Area (%)**
1	11.704	50.2244	1	11.705	97.8650
2	14.429	49.7756	2	14.443	2.1350

2-(Diethylamino)ethyl 4-(((1*R*,2*R*)-2-cyclohexyl-3-(4,4,5,5-tetramethyl-1,3,2-dioxaborolan-2-yl)-1-(*o*-tolyl)but-3-en-1-yl)amino)benzoate (3ab)

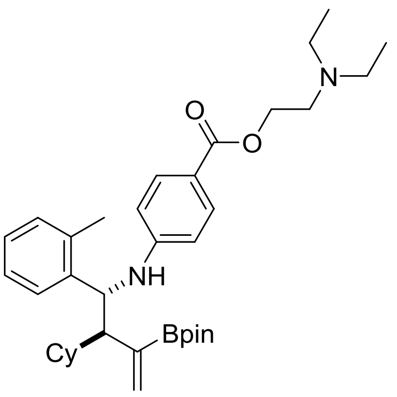

Prepared according to General Procedure 2, on a 0.258 mmol scale (94:6 d.r. of crude material), column chromatography (10-100% EtOAc in Hexanes) afforded the title compound as a yellow gum (48 mg, 0.081 mmol, 31%).

A pure sample of the major diastereoisomer was obtained; MS (ES^+) *m/z*: 589 ($M+H^+$). HRMS calcd for $C_{36}H_{54}N_2BO_4$: 589.4171. Found: 589.4177; ν_{max} (thin film/cm^{-1}): 3384, 2972, 2927, 2850, 1702, 1601, 1527, 1447, 1371, 1301, 1269, 1171, 1140, 1106, 1075; ^{1}H NMR (400 MHz, $CDCl_3$) δ ppm 1.06 (t, *J* = 7.2 Hz, 6 H, 2 x CH_2CH_3), 1.13 - 1.26 (m, 2 H, CH_2), 1.32 - 1.41 (2 x s, 12 H, 4 x CH_3), 1.56 - 2.00 (m, 9 H, CH_2 + CH), 2.22 (dd, *J* = 10.3, 4.0 Hz, 1 H, C*H*C=CH_2), 2.46 (s, 3 H, Ar-CH_3), 2.62 (q, *J* = 7.1 Hz, 4 H, 2 x C$H_2$$CH_3$), 2.81 (t, *J* = 6.3 Hz, 2 H, OCH_2CH_2), 4.30 (t, *J* = 6.3 Hz, 2 H, OCH_2), 4.86 (d, *J* = 3.5 Hz, 1 H, C=CH_2), 5.00 (dd, *J* = 5.7, 4.4 Hz, 1 H, CHN), 5.69 (d, *J* = 3.5 Hz, 1 H, C=CH_2), 6.31 (d, *J* = 8.8 Hz, 2 H, ArCH), 6.66 (d, *J* = 5.5 Hz, 1 H, NH), 6.87 - 6.94 (m, 1 H, ArCH), 6.95 - 7.01 (m, 1 H, ArCH), 7.05 (td, *J* = 7.4, 1.51 Hz, 1 H, ArCH), 7.10 - 7.14 (m, 1 H, ArCH), 7.74 (d, *J* = 8.8 Hz, 2 H, ArCH); ^{13}C NMR (101 MHz, $CDCl_3$) δ ppm 12.0 (CH_2CH_3), 19.1 (Ar-CH_3), 24.4 (CH_3), 25.1 (CH_3), 26.3 (CH_2), 26.4 (CH_2), 26.5 (CH_2), 30.8 (CH_2), 33.1 (CH_2), 36.9 (CH), 47.7 (*C*H_2CH_3), 51.0 (OCH_2*C*H_2), 53.7 (CHN), 58.0 (*C*HC=CH_2), 62.3 (OCH_2), 84.1 (OC), 111.2 (ArCH), 117.0 (ArC), 125.5 (ArCH), 126.3 (ArCH), 127.5 (ArCH), 130.3 (ArCH), 131.5 (ArCH), 134.2 (ArC), 136.0 (C=*C*H_2), 139.7 (ArC), 151.8 (ArC), 166.9 (C=O), (B*C*=CH_2 not observed); ^{11}B NMR (128 MHz, $CDCl_3$) δ ppm -6.0, 30.0; Specific rotation: $[\alpha]_D^{27}$ -36.5 (*c* 0.49, $CHCl_3$) for an enantiomerically enriched sample of 96:4 e.r.

Enantiomeric purity of **3ab** was determined by HPLC analysis in comparison with authentic racemic material (96:4 e.r. shown; Lux 5 µm Amylose-1 column, 94:6 hexanes:*i*-PrOH (+0.1% diethylamine), 0.3 mL/min, 10 °C, 220 nm).

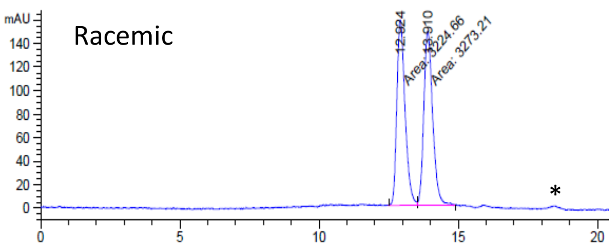

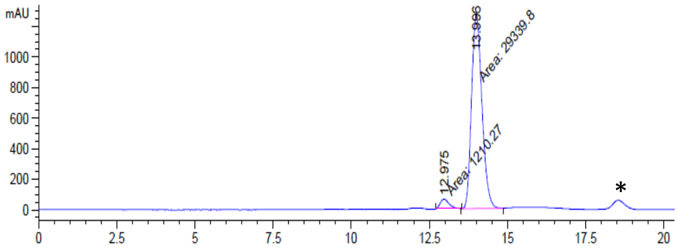

* = minor diastereoisomer

Peak #	RetTime [min]	Type	Width [min]	Area [mAU*s]	Height [mAU]	Area %
1	12.924	MF	0.3406	3224.65918	157.80739	49.6264
2	13.910	FM	0.3682	3273.21338	148.15764	50.3736

Peak #	RetTime [min]	Type	Width [min]	Area [mAU*s]	Height [mAU]	Area %
1	12.975	MM	0.3265	1210.26660	61.78379	3.9616
2	13.996	MM	0.3865	2.93398e4	1265.30859	96.0384

**Peak #**	**Time (min)**	**Area (%)**	**Peak #**	**Time (min)**	**Area (%)**
1	12.924	49.6264	1	12.975	3.9616
2	13.910	50.3736	2	13.996	96.0384

Gram scale procedure for the synthesis of *N*-((1*R*,2*R*)-2-Cyclohexyl-3-(4,4,5,5-tetramethyl-1,3,2-dioxaborolan-2-yl)-1-(*o*-tolyl)but-3-en-1-yl)-4-methoxyaniline (3a)

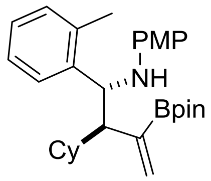

To a solution of CuI (8.5 mg, 0.044 mmol, 1 mol%) and NHC ligand **6** (28.7 mg, 0.049 mmol, 1.1 mol%) in THF (2.8 mL), was added *t*-BuOK (4.40 mL of a 1 M THF solution, 4.440 mmol, 1 equiv), and the reaction was stirred for 100 minutes at room temperature. B_2Pin_2 (1.24 g, 4.883 mmol, 1.1 equiv) in THF (12.9 mL) was then added and the resulting mixture stirred for 60 minutes. A solution of propa-1,2-dien-1-ylcyclohexane (**2a**) (814 mg, 6.658 mmol, 1.5 equiv) and (*E*)-*N*-(4-methoxyphenyl)-1-(*o*-tolyl)methanimine (**1a**) (1.00 g, 4.439 mmol, 1 equiv) in THF (17.2 mL) was then added dropwise at room temperature with stirring overnight. The mixture was then filtered through a silica plug, concentrated *in vacuo* and the crude product mixture (>95:5 d.r.) was purified by chromatography (3% EtOAc in hexanes) to afford the title compound as an orange gum (2.08 g, 4.35 mmol, 98%).

Enantiomeric purity of **3a** (gram scale) was determined by HPLC analysis in comparison with authentic racemic material (98:2 e.r. shown; Lux 5 µm Amylose-1 column, 99:1 hexanes:*i*-PrOH, 0.3 mL/min, 20 °C, 254 nm).

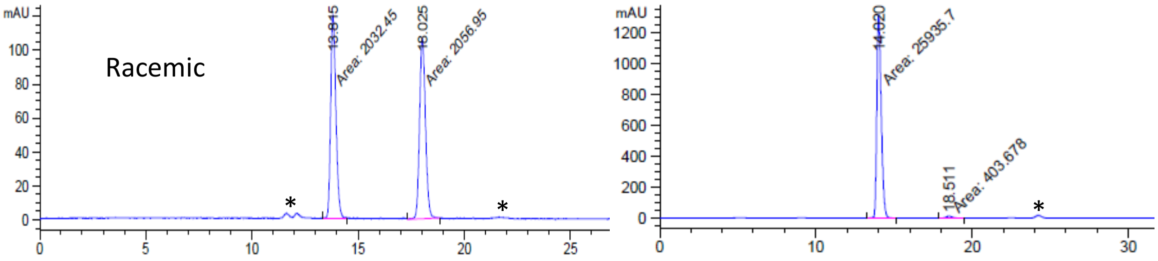

* = minor diastereoisomer

Peak #	RetTime [min]	Type	Width [min]	Area [mAU*s]	Height [mAU]	Area %
1	13.815	MM	0.2837	2032.45325	119.39373	49.7005
2	18.025	MM	0.3244	2056.94873	105.68186	50.2995

Peak #	RetTime [min]	Type	Width [min]	Area [mAU*s]	Height [mAU]	Area %
1	14.020	MM	0.3282	2.59357e4	1317.19519	98.4674
2	18.511	MM	0.5164	403.67761	13.02794	1.5326

Peak #	Time (min)	Area (%)	Peak #	Time (min)	Area (%)
1	13.815	49.7005	1	14.020	98.4674
2	18.025	50.2995	2	18.511	1.5326

Derivatisation of 3a

Procedure for oxidation of the vinyl boronate product

The racemate of the methyl ketone was prepared according to literature procedures.[16]

(3*R*,4*S*)-3-Cyclohexyl-4-((4-methoxyphenyl)amino)-4-(*o*-tolyl)butan-2-one (7)

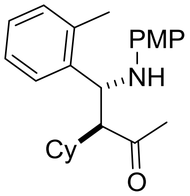

To *N*-((1*R*,2*R*)-2-cyclohexyl-3-(4,4,5,5-tetramethyl-1,3,2-dioxaborolan-2-yl)-1-(*o*-tolyl)but-3-en-1-yl)-4-methoxyaniline (**3a**) (44 mg, 0.093 mmol, 1 equiv, 98:2 e.r., >95:5 d.r.) in THF (0.47 mL) at 0 °C was added H_2O_2 (0.05 mL of a 30% w:v aqueous solution, 0.467 mmol, 5 equiv) and NaOH (0.23 mL of a 2 M aqueous solution, 0.467 mmol, 5 equiv) and stirred for 30 min. The aqueous layer was then washed with Et_2O (3 x 2 mL), dried over $MgSO_4$ and concentration *in vacuo*. The crude product mixture (>95:5 d.r.) was purified by chromatography (3% EtOAc in hexanes) to afford the title compound as a colourless gum (28 mg, 0.077 mmol, 82%).

MS (ES^+) *m/z*: 366 ($M+H^+$). HRMS calcd for $C_{24}H_{32}NO_2$: 366.2433. Found: 366.2415; ν_{max} (thin film/cm^{-1}): 3407, 2924, 2851, 1702, 1511, 1484, 1359, 1241, 1225, 1168, 1110, 1039; ^1H NMR (400 MHz,

$CDCl_3$) δ ppm 0.79 - 1.42 (m, 6 H, CH_2), 1.54 (s, 3 H, CH_3), 1.57 - 1.80 (m, 3 H, CH_2), 1.93 - 2.13 (m, 2 H, CH_2 + CH), 2.50 (s, 3 H, Ar-CH_3), 2.78 (dd, *J* = 10.3, 3.3 Hz, 1 H, CHC=O), 3.68 (s, 3 H, OCH_3), 4.86 (d, *J* = 3.5 Hz, 1 H, CHN), 5.25 (br. s, 1 H, NH), 6.36 (d, *J* = 8.8 Hz, 2 H, ArCH), 6.62 - 6.71 (m, 2 H, ArCH), 7.02 - 7.15 (m, 3 H, ArCH), 7.16 - 7.21 (m, 1 H, ArCH); ^{13}C NMR (101 MHz, $CDCl_3$) δ ppm 18.9 (Ar-CH_3), 26.0 (2 x CH_2), 26.3 (CH_2), 29.8 (CH_2), 32.2 (CH_3C=O), 35.5 (CH_2), 37.6 (CH), 51.8 (CHN), 55.7 (OCH_3), 60.6 (*C*HC=O), 113.3 (ArCH), 114.8 (ArCH), 125.9 (ArCH), 126.5 (ArCH), 126.9 (ArCH), 130.8 (ArCH), 134.1 (ArC), 139.3 (ArC), 141.0 (ArC), 151.2 (ArC), 215.4 (C=O); Specific rotation: $[\alpha]_D^{26}$ -37.0 (*c* 0.98, $CHCl_3$) for an enantiomerically enriched sample of 98:2 e.r.

Enantiomeric purity of **7** was determined by HPLC analysis in comparison with authentic racemic material (98:2 e.r. shown; Lux 5 µm Amylose-1 column, 99:1 hexanes:*i*-PrOH, 0.3 mL/min, 30 °C, 254 nm).

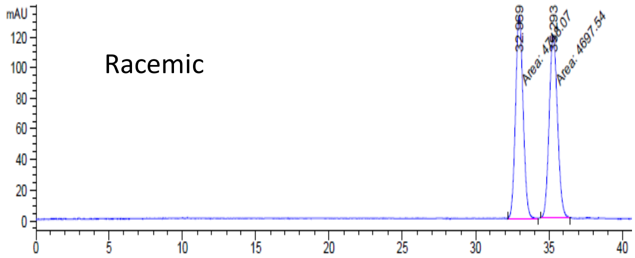

Peak #	RetTime [min]	Type	Width [min]	Area [mAU*s]	Height [mAU]	Area %
1	32.969	MM	0.5966	4748.06934	132.64490	50.2675
2	35.293	MM	0.6582	4697.53662	118.95356	49.7325

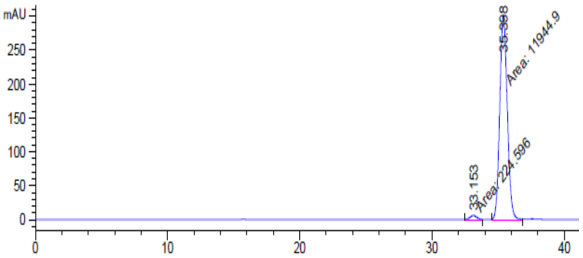

Peak #	RetTime [min]	Type	Width [min]	Area [mAU*s]	Height [mAU]	Area %
1	33.153	MM	0.6213	224.59634	6.02535	1.8456
2	35.398	MM	0.6622	1.19449e4	300.64908	98.1544

Peak #	Time (min)	Area (%)	Peak #	Time (min)	Area (%)
1	32.969	50.2675	1	33.153	1.8456
2	35.293	49.7325	2	35.398	98.1544

Procedure for hydrogenation of the vinyl boronate product

The racemate of the methyl ketone was prepared according to literature procedures.[16]

***N*-((1*R*,2*S*,3*S*)-2-Cyclohexyl-3-(4,4,5,5-tetramethyl-1,3,2-dioxaborolan-2-yl)-1-(*o*-tolyl)butyl)-4-methoxyaniline (8)**

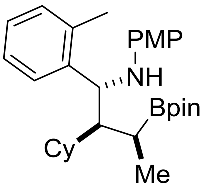

A suspension of *N*-((1*R*,2*R*)-2-cyclohexyl-3-(4,4,5,5-tetramethyl-1,3,2-dioxaborolan-2-yl)-1-(*o*-tolyl)but-3-en-1-yl)-4-methoxyaniline (**3a**) (30 mg, 0.063 mmol, 1 equiv, 98:2 e.r., >95:5 d.r.) and palladium (6.7 mg, 10 wt.% loading on carbon) in ethanol (1 mL) was stirred for 16 hours at 20 °C in a Parr reactor at 49 bar of H_2. The reaction mixture was filtered through a pad of Celite® and concentrated *in vacuo*. The crude product mixture (>95:5 d.r.) was purified by chromatography (5% EtOAc in hexanes) to afford the title compound as a colourless gum (16 mg, 0.034 mmol, 54%).

MS (ES$^+$) *m/z*: 478 (M+H$^+$). HRMS calcd for $C_{30}H_{45}NBO_3$: 478.3487. Found: 478.3469; ν_{max} (thin film/cm^{-1}): 3405, 2924, 2851, 1511, 1461, 1371, 1306, 1233, 1142, 1110, 1041; ^{1}H NMR (400 MHz, $CDCl_3$) δ ppm 0.75 (d, *J* = 7.8 Hz, 3 H, CH(C*H*$_3$)), 0.96 - 1.09 (m, 2 H, CH_2), 1.10 - 1.21 (m, 3 H, CH_2), 1.33 (s, 12 H, 4 x CH_3), 1.35 - 1.42 (m, 1 H, C*H*(CH_3)), 1.50 (dd, *J* = 9.3, 3.8 Hz, 1 H, C*H*CH(CH_3)), 1.61 - 1.86 (m, 5 H, CH_2 + CH), 1.95 (d, *J* = 12.5 Hz, 1 H, CH_2), 2.41 (s, 3 H, Ar-CH_3), 3.68 (s, 3 H, OCH_3), 4.73 (apparent s, 1 H, CHN), 5.45 (br. s, 1 H, NH), 6.29 (d, *J* = 9.0 Hz, 2 H, ArCH), 6.66 (d, *J* = 9.0 Hz, 2 H, ArCH), 7.06 - 7.15 (m, 3 H, ArCH), 7.32 - 7.39 (m, 1 H, ArCH); ^{13}C NMR (101 MHz, $CDCl_3$) δ ppm 18.5 (CH(*C*H$_3$)), 19.3 (Ar-CH_3), 25.0 (CH_3), 25.1 (CH_3), 26.5 (CH_2), 26.7 (CH_2), 26.7 (CH_2), 31.5 (CH_2), 32.9 (CH_2), 39.5 (CH), 53.0 (*C*HCH(CH_3)), 55.3 (CHN), 55.8 (OCH_3), 83.4 (OC), 113.3 (ArCH), 114.8 (ArCH), 125.7 (ArCH), 126.1 (ArCH), 127.4 (ArCH), 130.6 (ArCH), 134.8 (ArC), 141.2 (ArC), 142.3 (ArC), 150.9 (ArC), (B*C*H(CH_3) not observed); ^{11}B NMR (128 MHz, $CDCl_3$) δ ppm -5.5, 34.3; Specific rotation: $[\alpha]_D^{28}$ -14.9 (*c* 0.78, $CHCl_3$) for an enantiomerically enriched sample of >99:1 e.r.

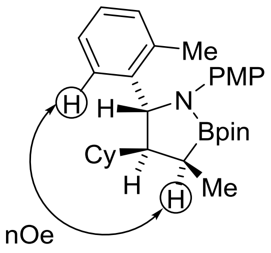

Enantiomeric purity of **8** was determined by HPLC analysis in comparison with authentic racemic material (>99:1 e.r. shown; Lux 5 µm Amylose-1 column, 100% hexanes, 0.5 mL/min, 20 °C, 254 nm).

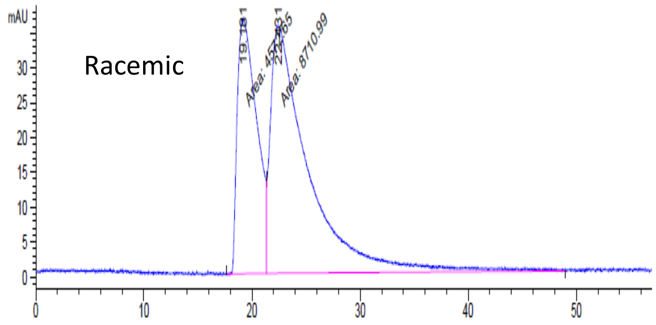

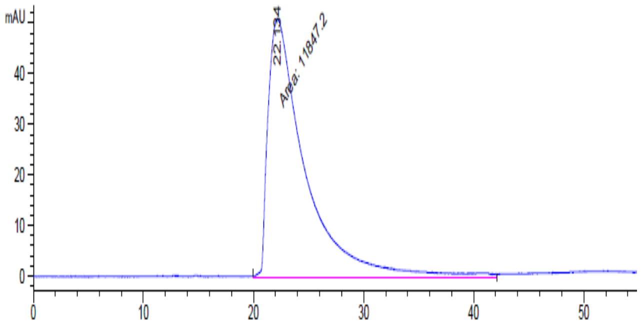

^{1}H and ^{13}C NMR spectra

(*E*)-1-(Benzo[*d*][1,3]dioxol-4-yl)-*N*-(4-methoxyphenyl)methanimine (1c)

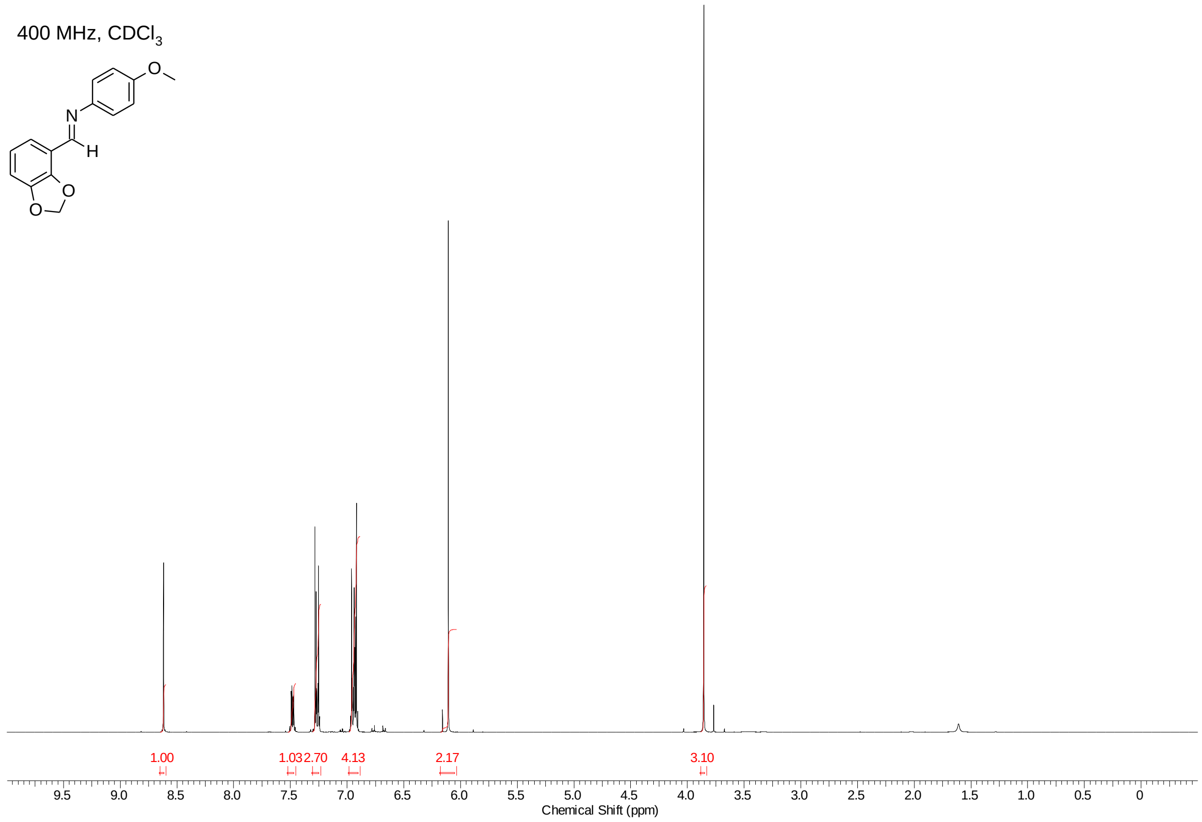

(*E*)-1-(Benzo[*d*][1,3]dioxol-4-yl)-*N*-(4-methoxyphenyl)methanimine (1c)

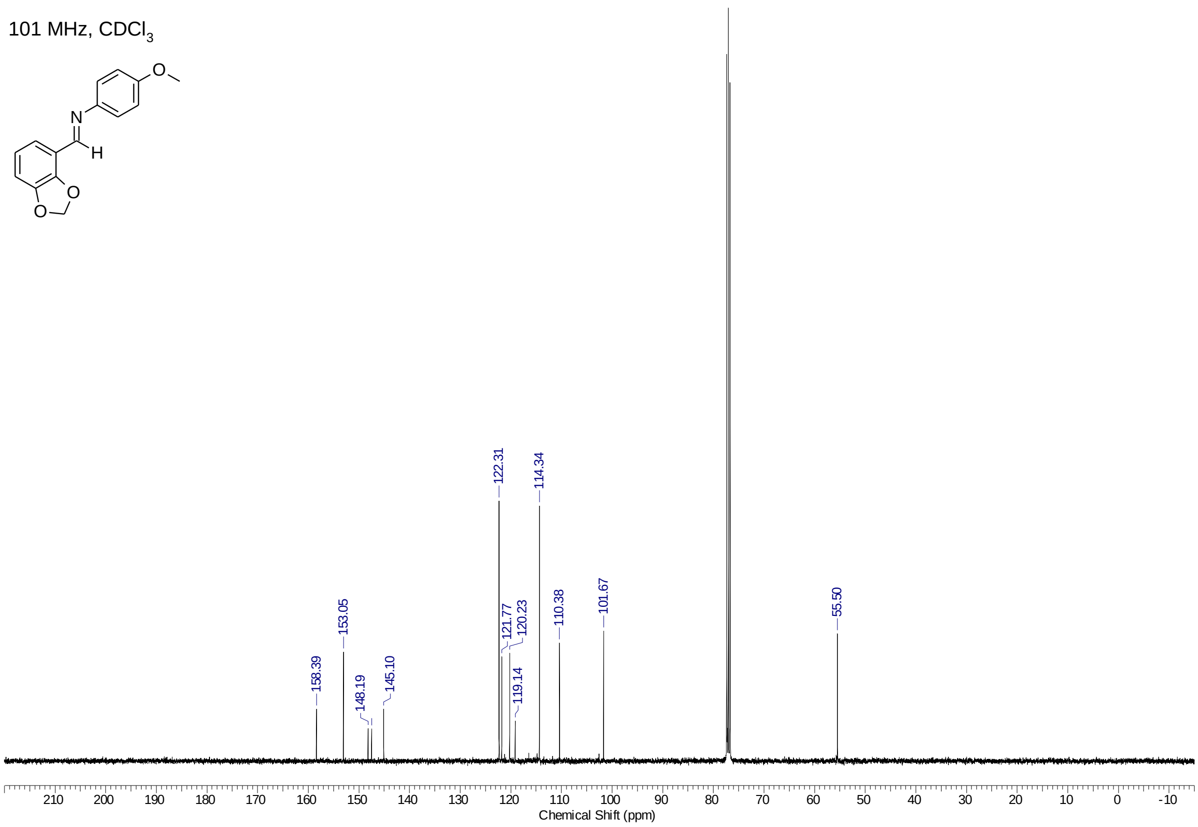

(*E*)-*N*-(4-Methoxyphenyl)-1-(2-(methylthio)phenyl)methanimine (1e)

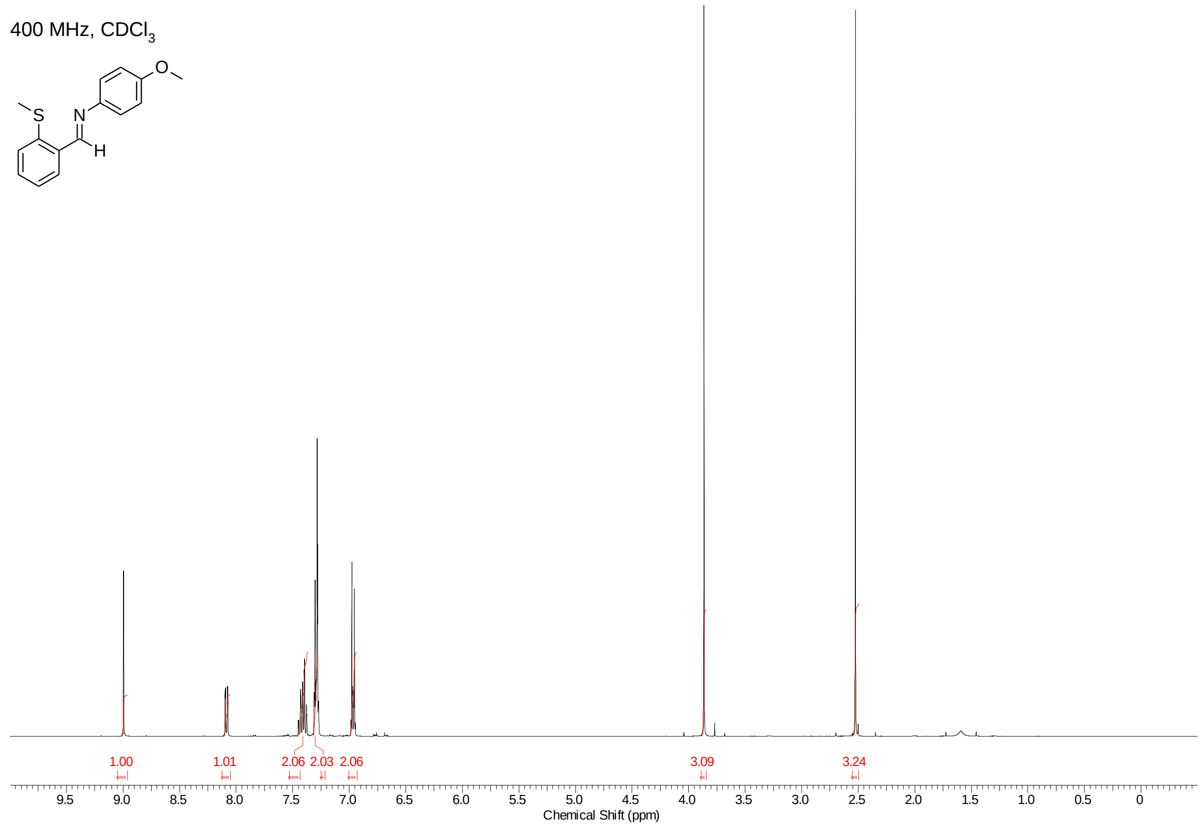

(*E*)-*N*-(4-Methoxyphenyl)-1-(2-(methylthio)phenyl)methanimine (1e)

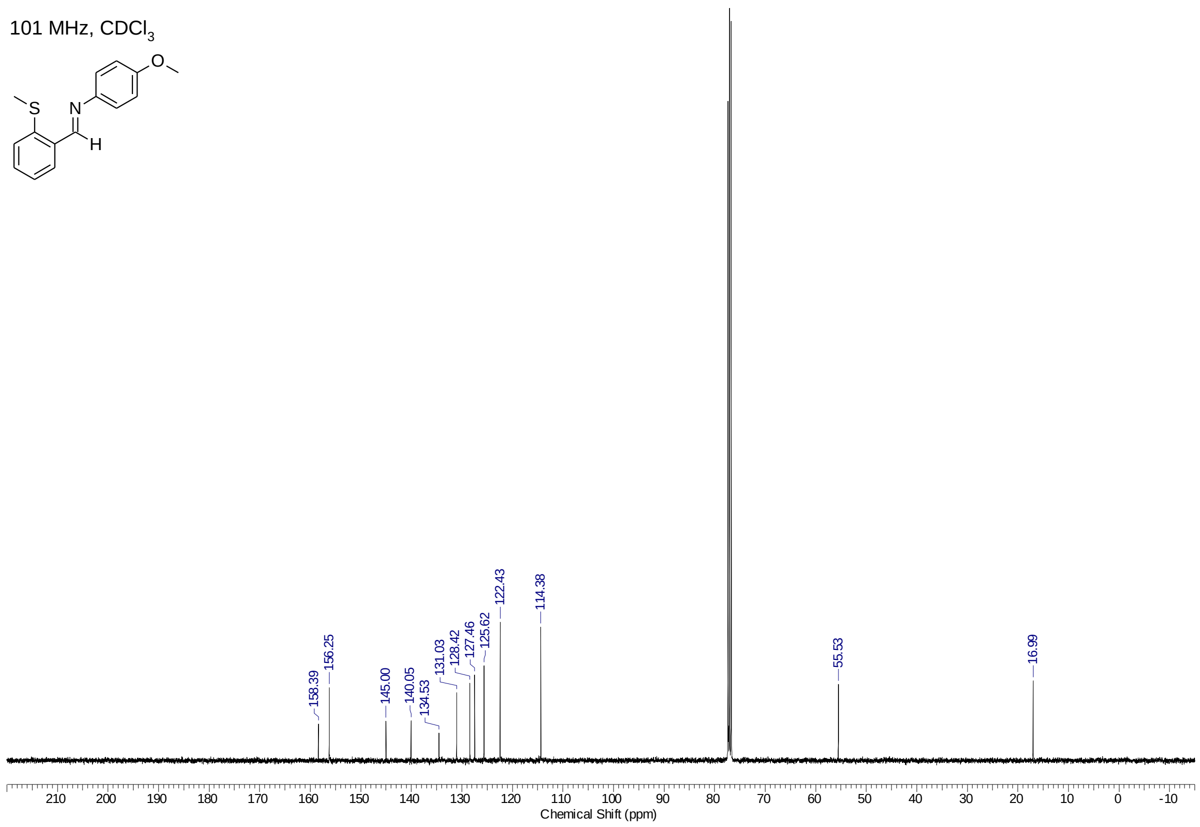

Methyl (*E*)-4-((2-methylbenzylidene)amino)benzoate (1x)

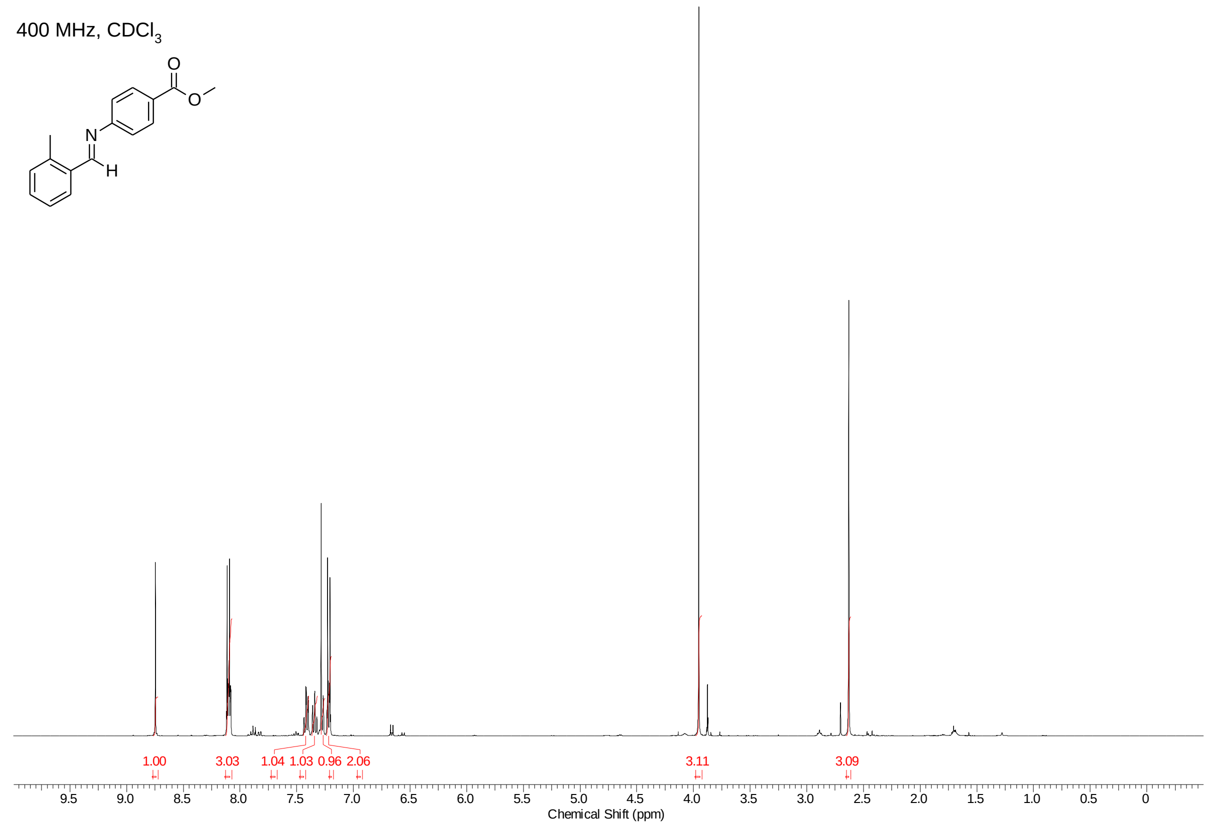

Methyl (*E*)-4-((2-methylbenzylidene)amino)benzoate (1x)

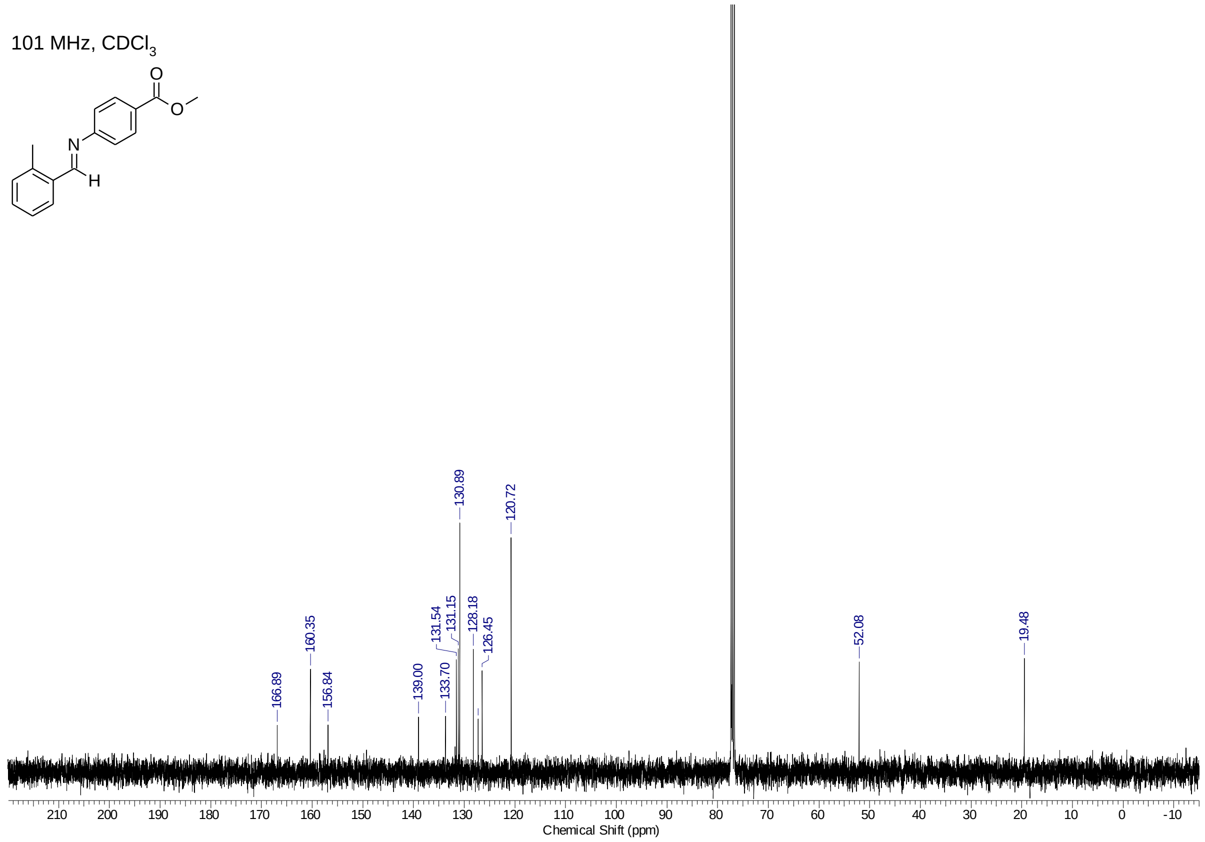

(*E*)-*N*-(4-Morpholinophenyl)-1-(*o*-tolyl)methanimine (1z)

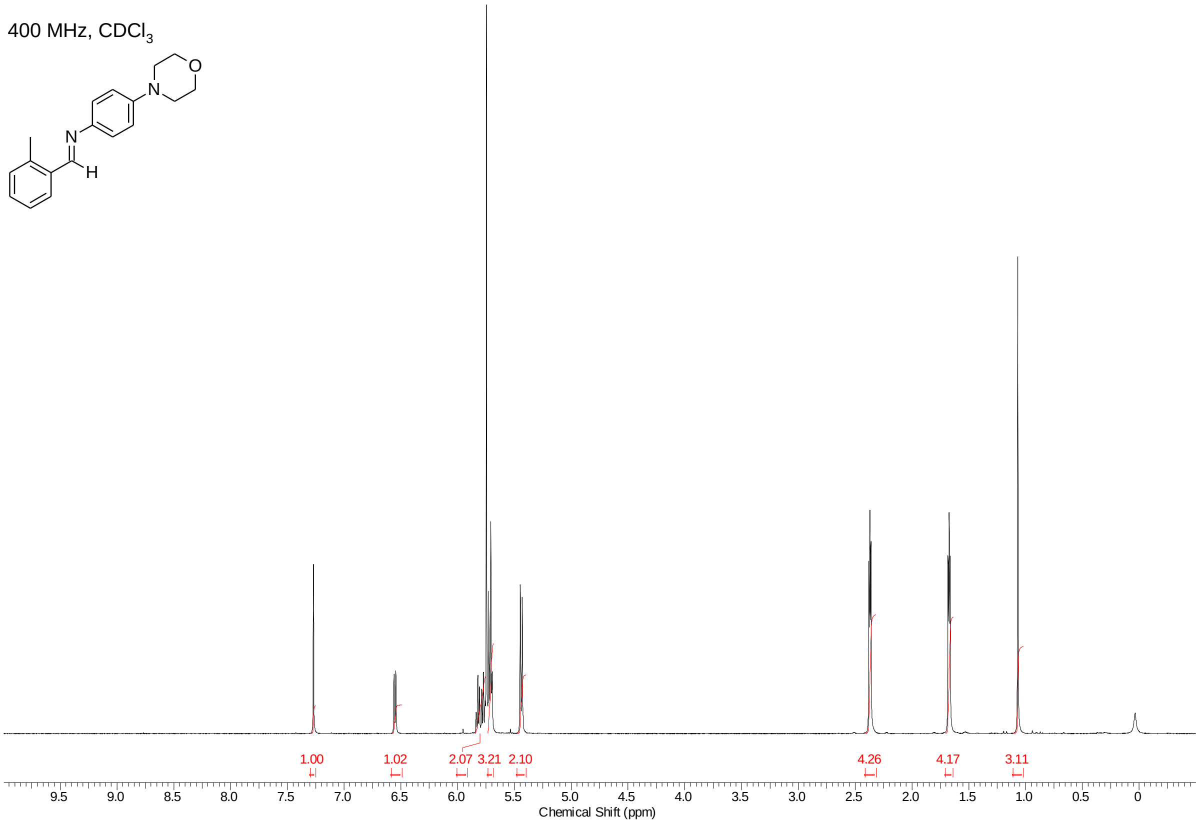

(*E*)-*N*-(4-Morpholinophenyl)-1-(*o*-tolyl)methanimine (1z)

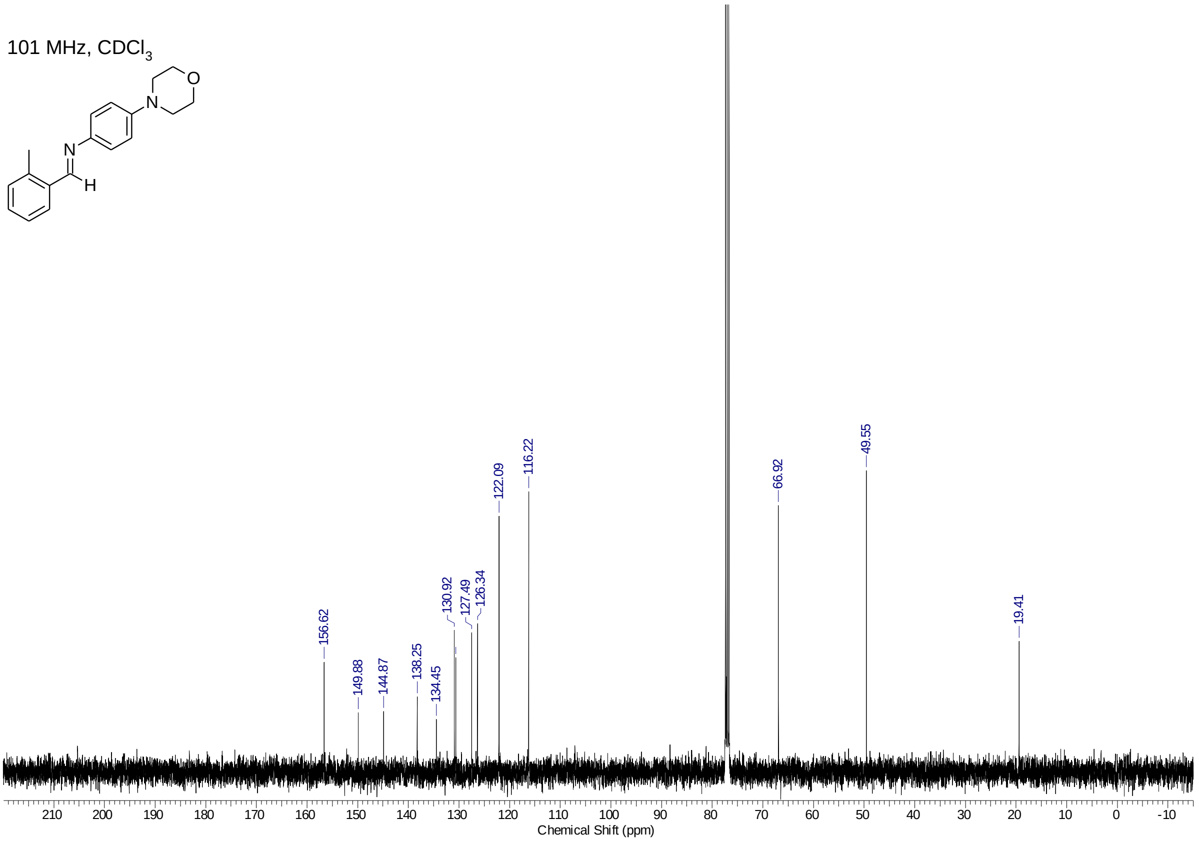

(*E*)-*N*-(4-(4,4,5,5-Tetramethyl-1,3,2-dioxaborolan-2-yl)phenyl)-1-(*o*-tolyl)methanimine (1aa)

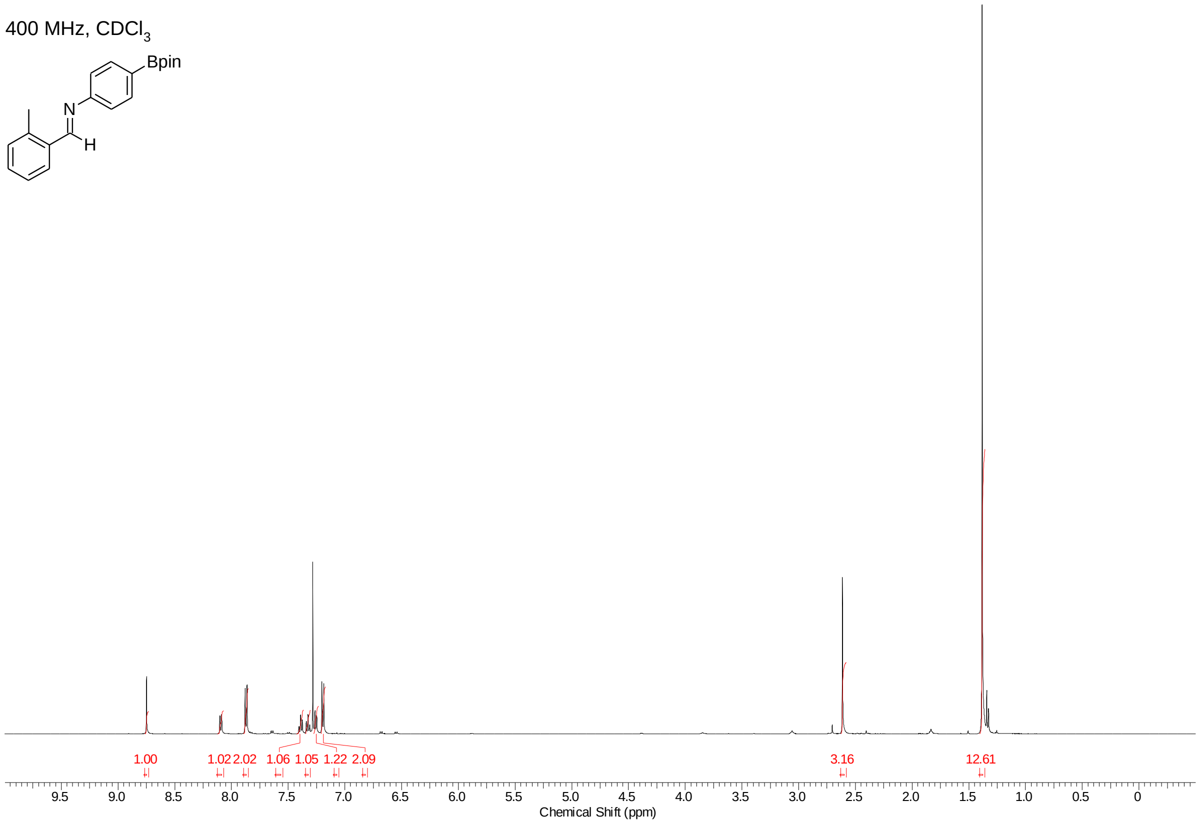

(*E*)-*N*-(4-(4,4,5,5-tetramethyl-1,3,2-dioxaborolan-2-yl)phenyl)-1-(*o*-tolyl)methanimine (1aa)

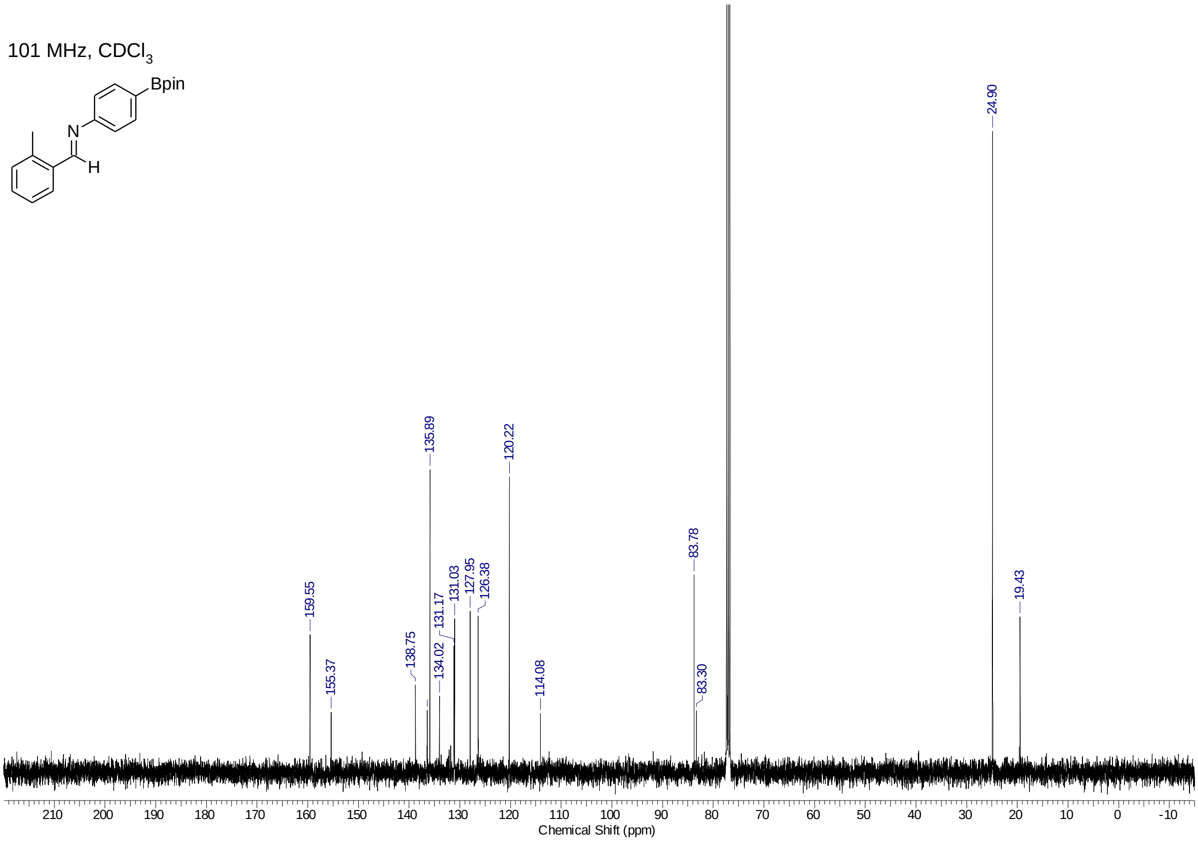

2-(Diethylamino)ethyl (*E*)-4-((2-methylbenzylidene)amino)benzoate (1ab)

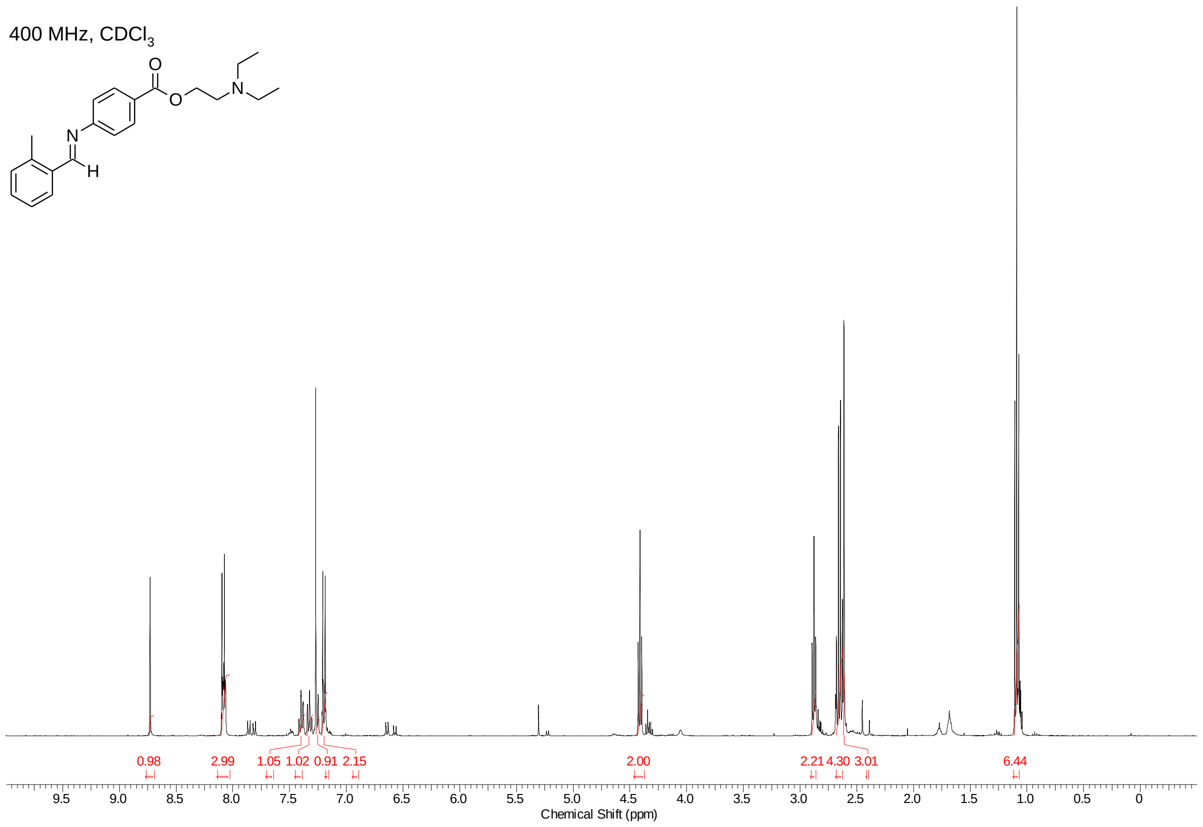

2-(Diethylamino)ethyl (*E*)-4-((2-methylbenzylidene)amino)benzoate (1ab)

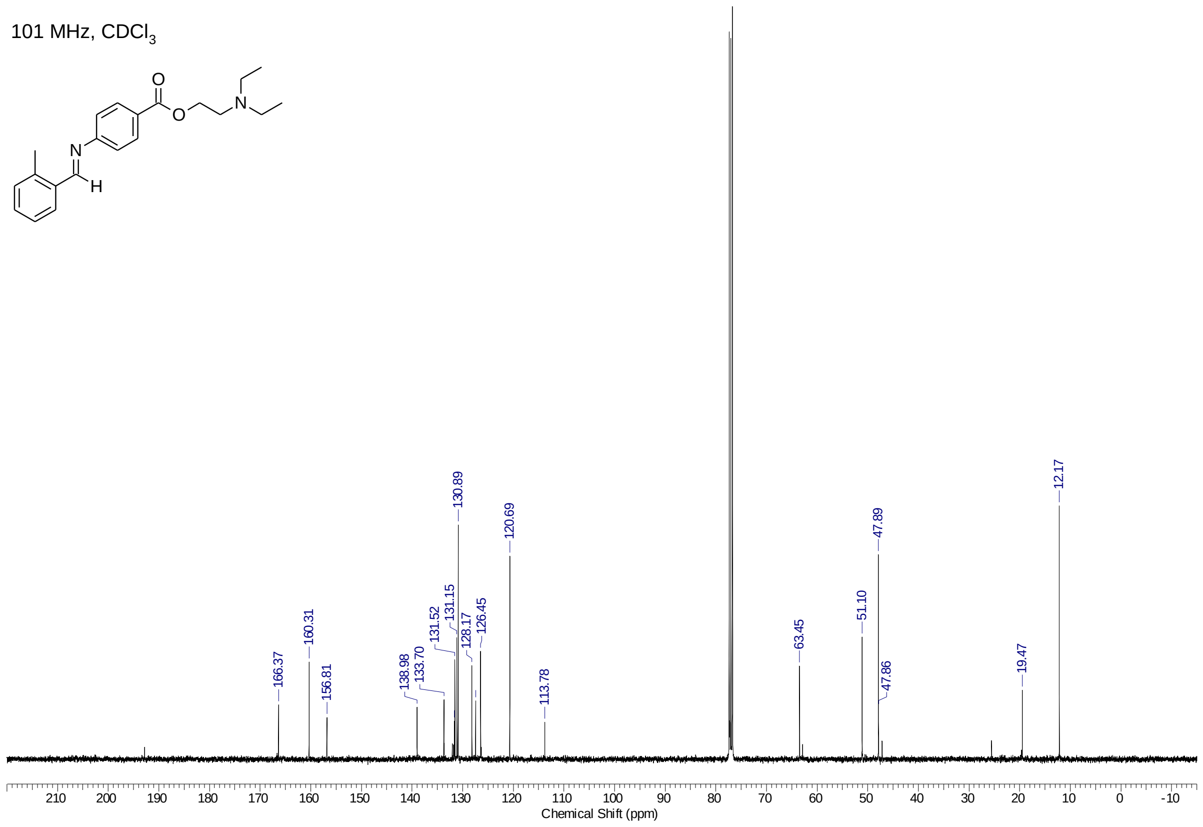

N-((1R,2R)-2-Cyclohexyl-3-(4,4,5,5-tetramethyl-1,3,2-dioxaborolan-2-yl)-1-(o-tolyl)but-3-en-1-yl)-4-methoxyaniline (3a)

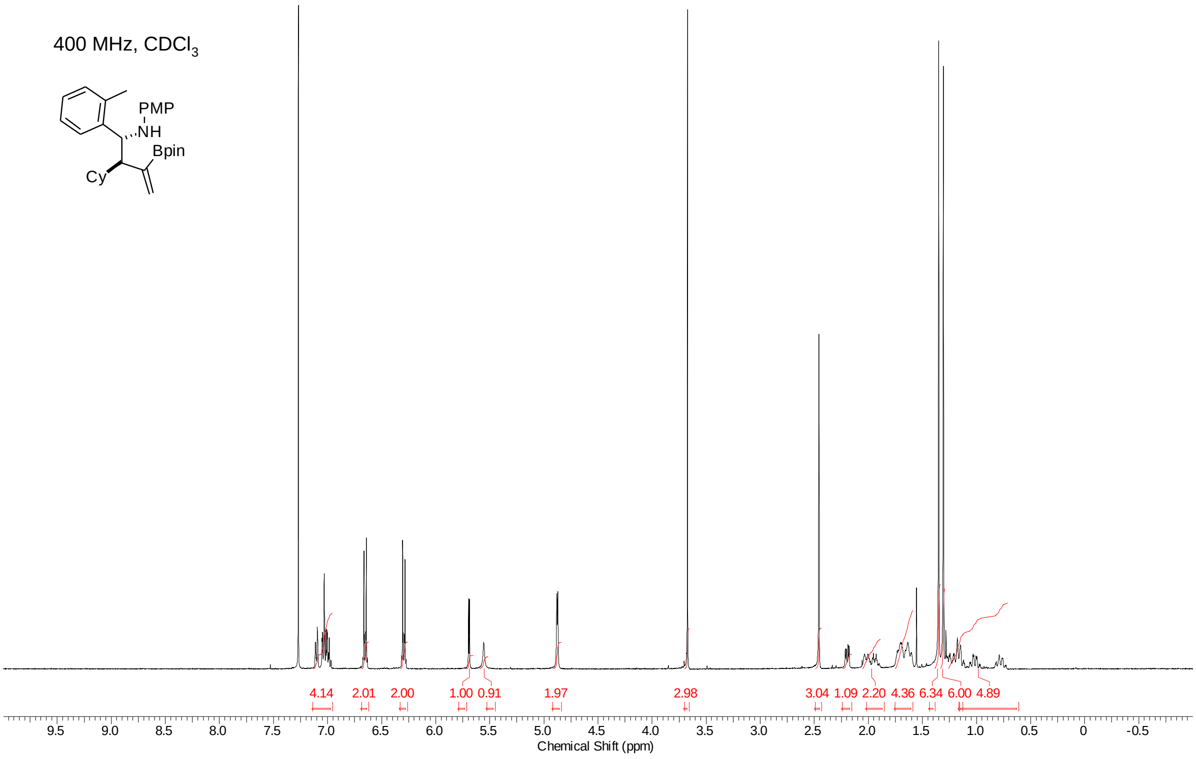

N-((1R,2R)-2-Cyclohexyl-3-(4,4,5,5-tetramethyl-1,3,2-dioxaborolan-2-yl)-1-(o-tolyl)but-3-en-1-yl)-4-methoxyaniline (3a)

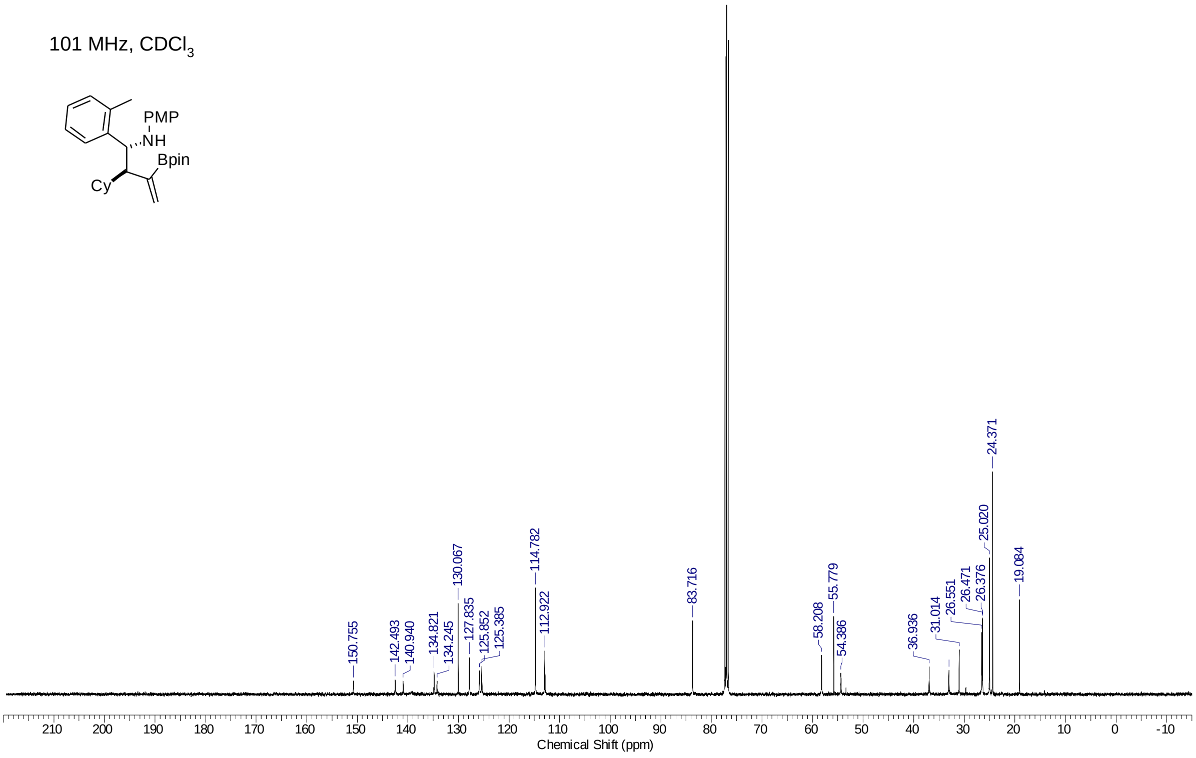

_N_-((1_R_,2_R_)-2-Cyclohexyl-1-(2-methoxyphenyl)-3-(4,4,5,5-tetramethyl-1,3,2-dioxaborolan-2-yl)but-3-en-1-yl)-4-methoxyaniline (3b)

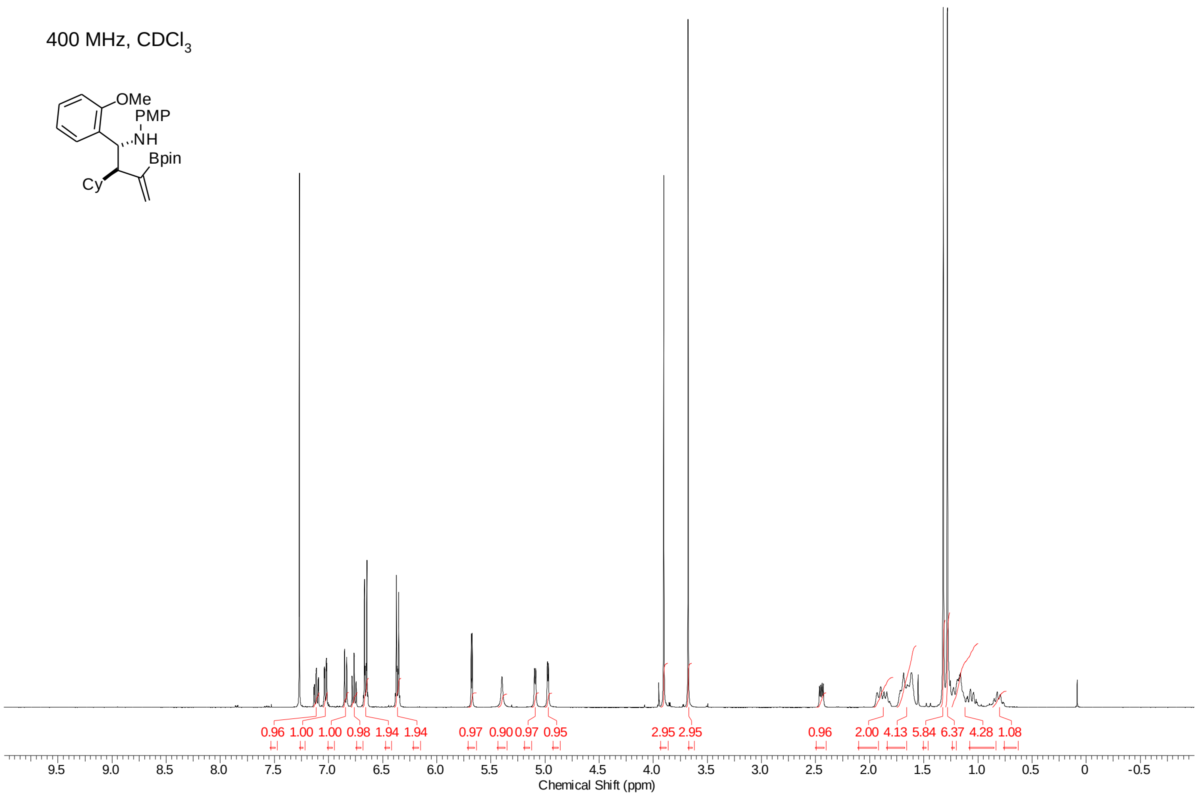

_N_-((1_R_,2_R_)-2-Cyclohexyl-1-(2-methoxyphenyl)-3-(4,4,5,5-tetramethyl-1,3,2-dioxaborolan-2-yl)but-3-en-1-yl)-4-methoxyaniline (3b)

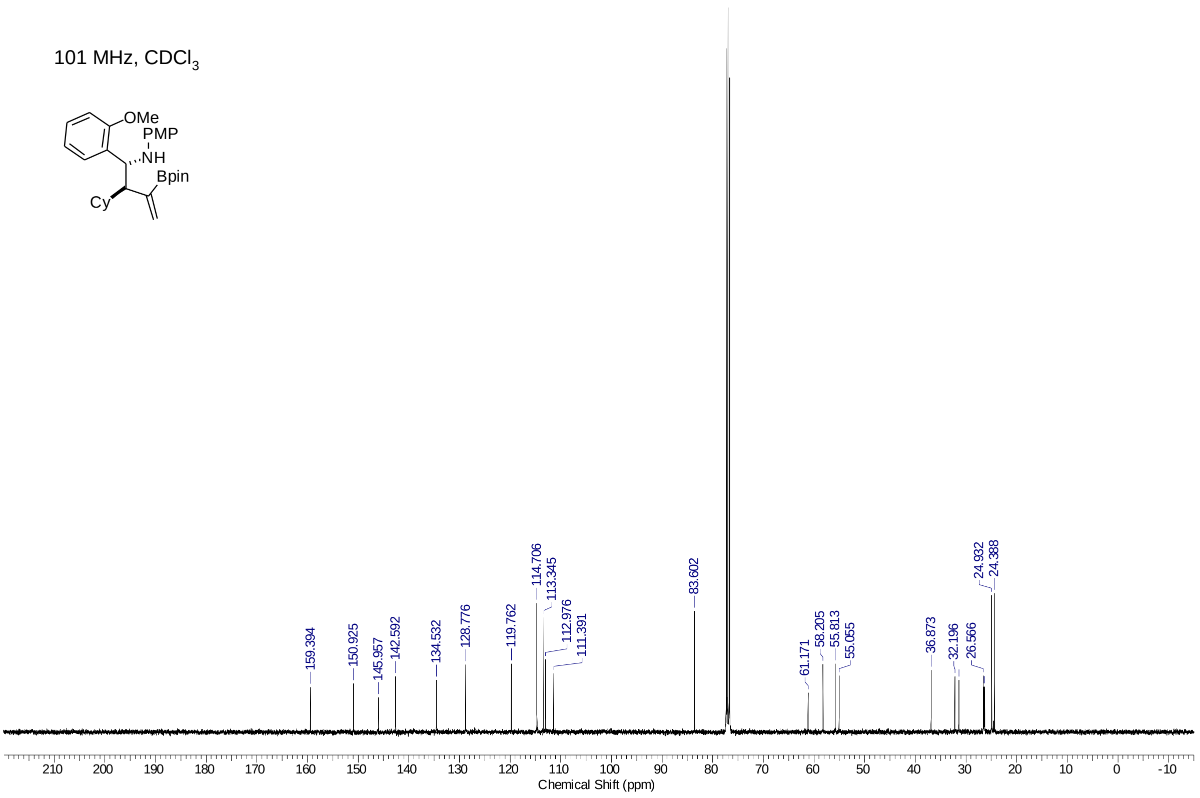

***N*-((1*R*,2*R*)-1-(Benzo[*d*][1,3]dioxol-4-yl)-2-cyclohexyl-3-(4,4,5,5-tetramethyl-1,3,2-dioxaborolan-2-yl)but-3-en-1-yl)-4-methoxyaniline (3c)**

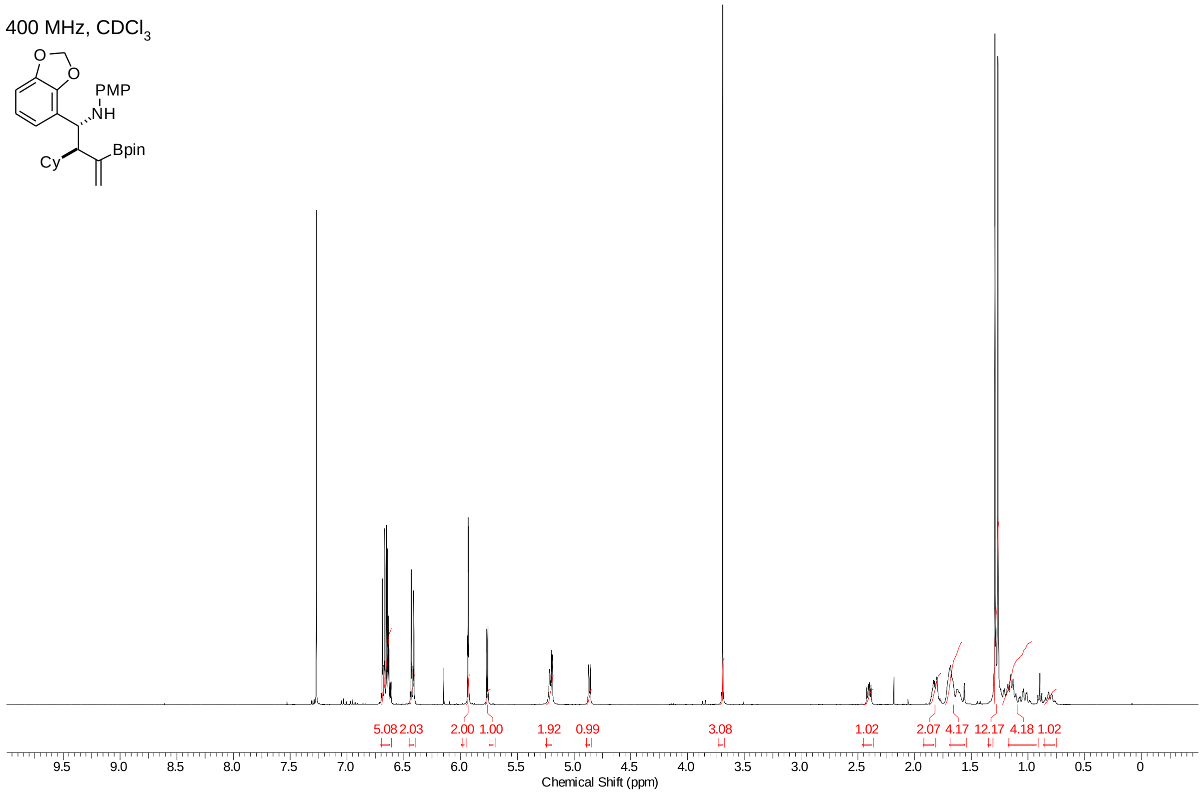

***N*-((1*R*,2*R*)-1-(Benzo[*d*][1,3]dioxol-4-yl)-2-cyclohexyl-3-(4,4,5,5-tetramethyl-1,3,2-dioxaborolan-2-yl)but-3-en-1-yl)-4-methoxyaniline (3c)**

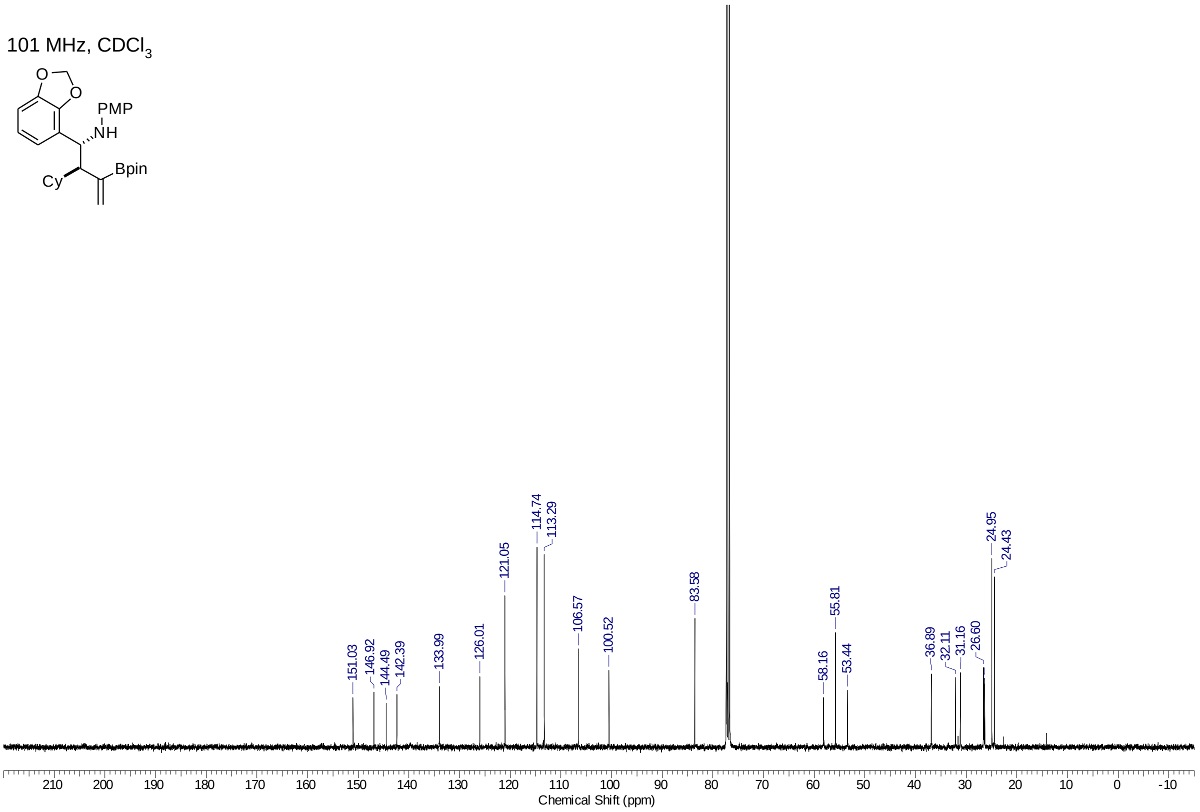

_N_-((1_R_,2_R_)-2-Cyclohexyl-1-(naphthalen-1-yl)-3-(4,4,5,5-tetramethyl-1,3,2-dioxaborolan-2-yl)but-3-en-1-yl)-4-methoxyaniline (3d)

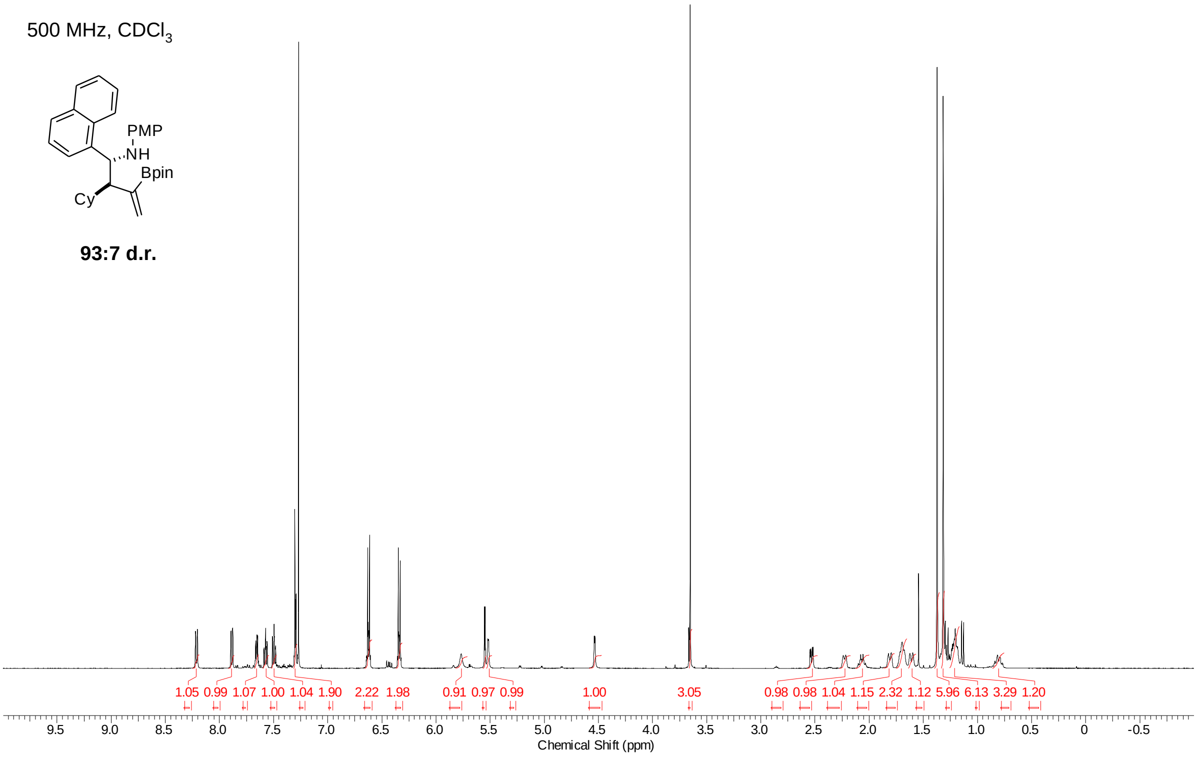

_N_-((1_R_,2_R_)-2-Cyclohexyl-1-(naphthalen-1-yl)-3-(4,4,5,5-tetramethyl-1,3,2-dioxaborolan-2-yl)but-3-en-1-yl)-4-methoxyaniline (3d)

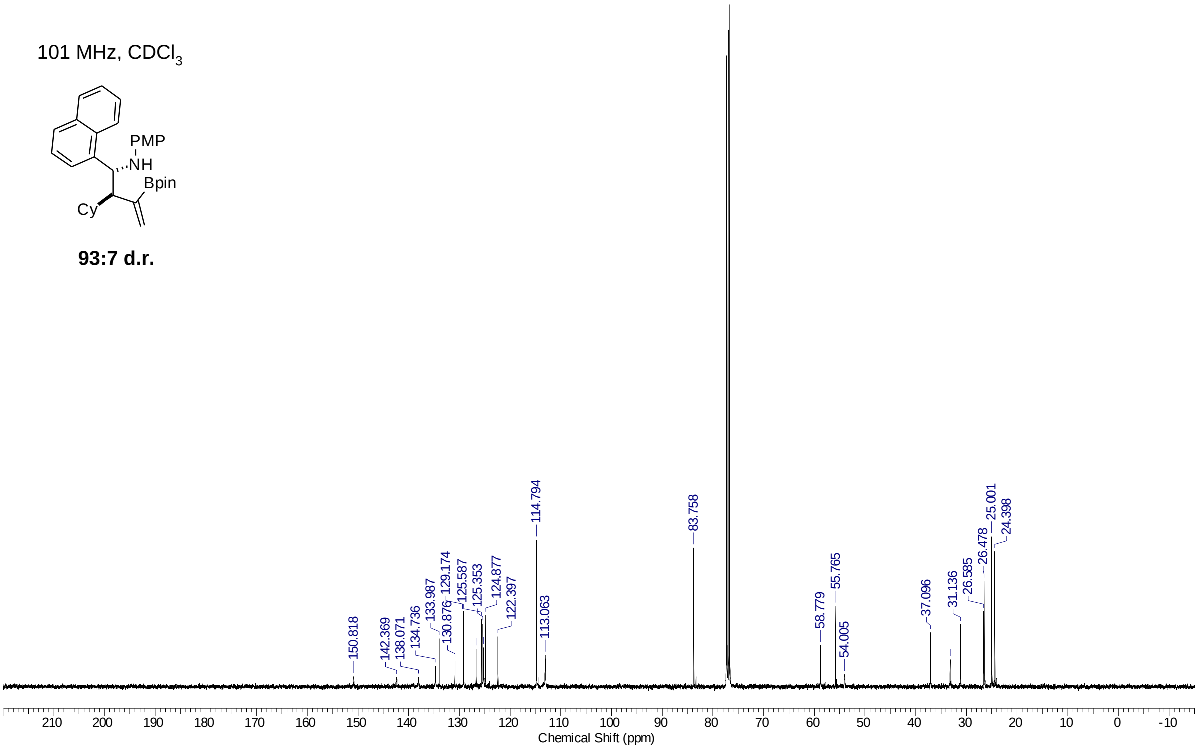

N-((1*R*,2*R*)-2-Cyclohexyl-1-(2-(methylthio)phenyl)-3-(4,4,5,5-tetramethyl-1,3,2-dioxaborolan-2-yl) but-3-en-1-yl)-4-methoxyaniline (3e)

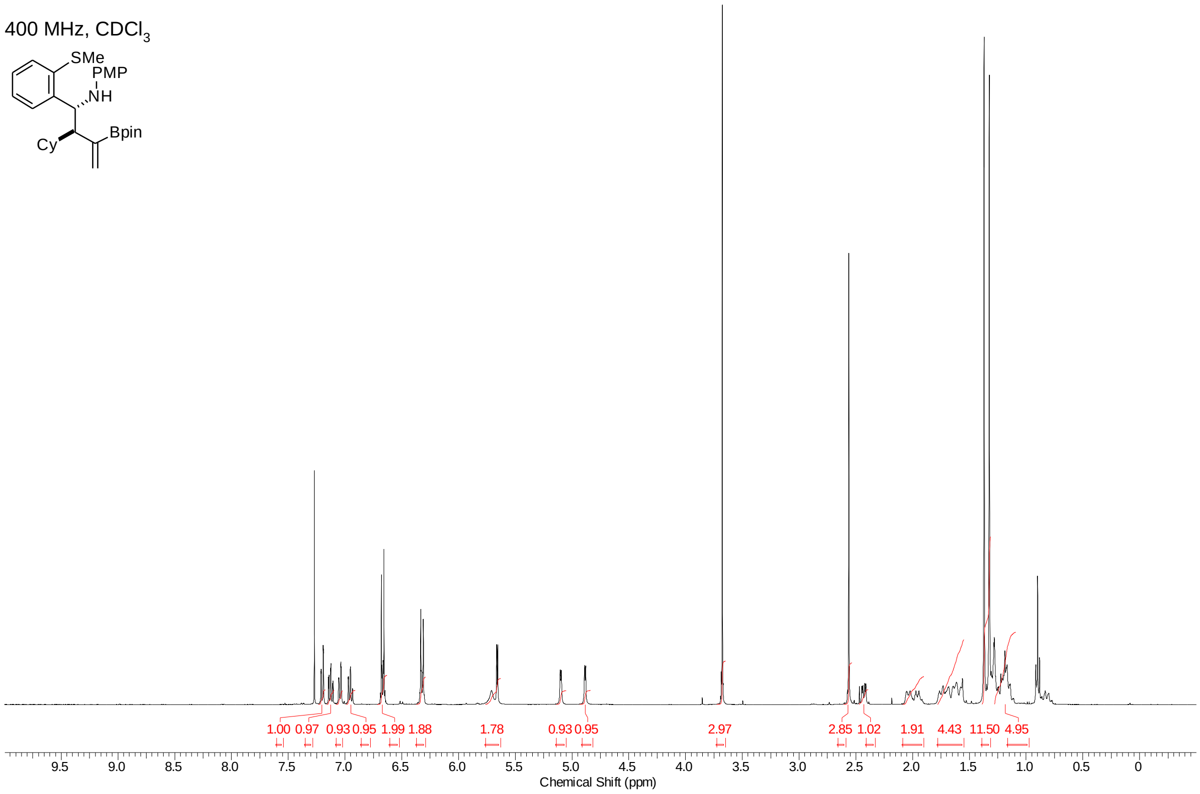

N-((1*R*,2*R*)-2-Cyclohexyl-1-(2-(methylthio)phenyl)-3-(4,4,5,5-tetramethyl-1,3,2-dioxaborolan-2-yl) but-3-en-1-yl)-4-methoxyaniline (3e)

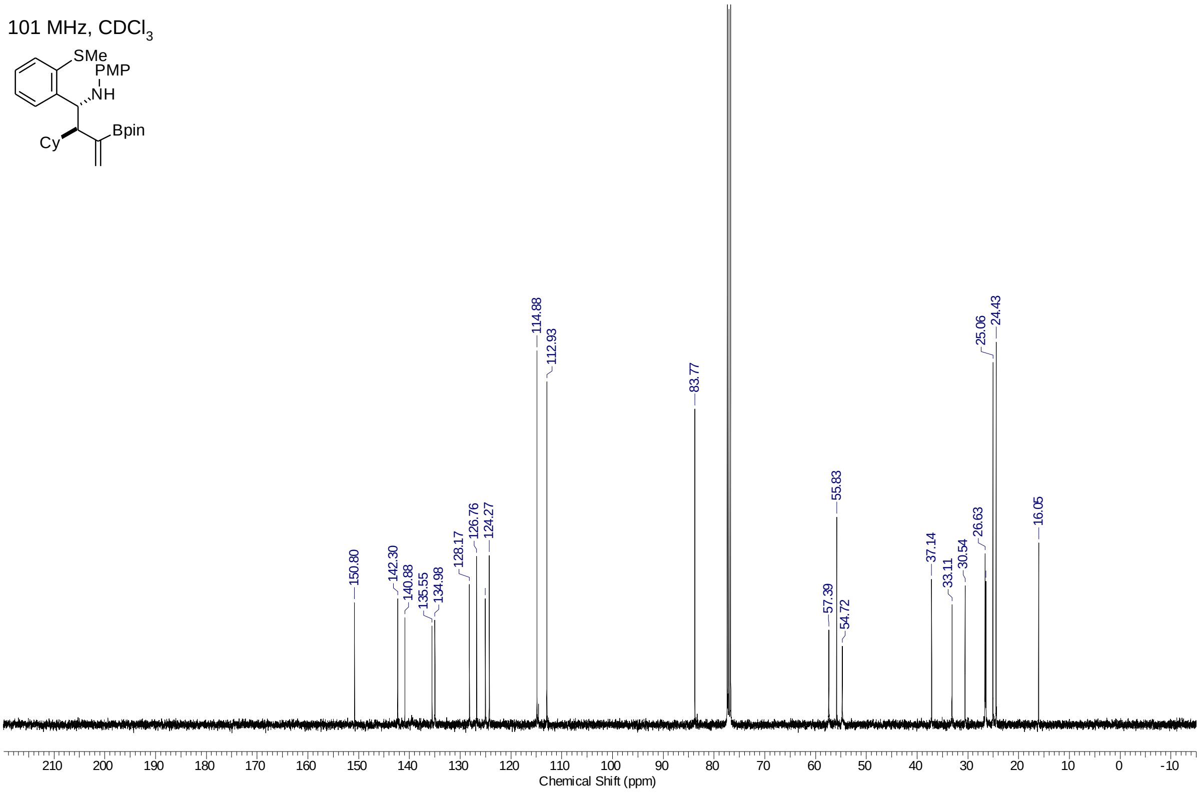

N-((1*R*,2*R*)-2-Cyclohexyl-1-phenyl-3-(4,4,5,5-tetramethyl-1,3,2-dioxaborolan-2-yl)but-3-en-1-yl)-4-methoxyaniline (3f)

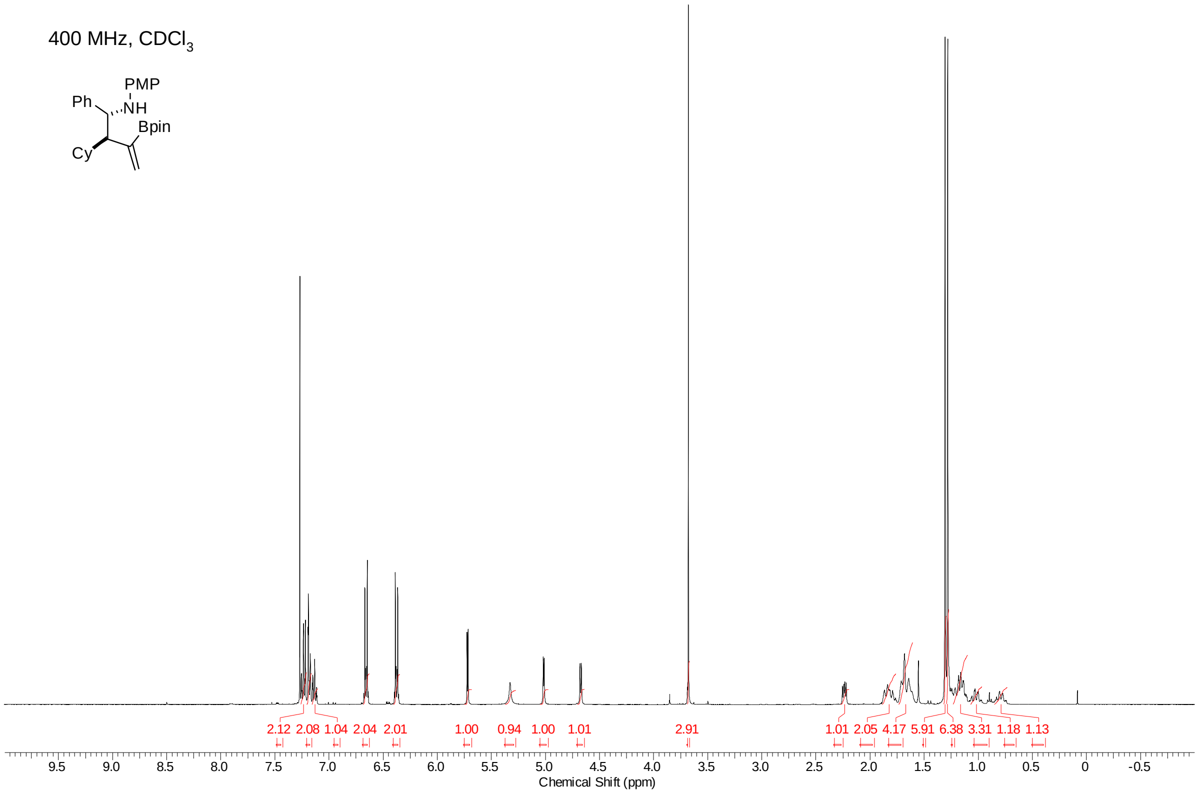

N-((1*R*,2*R*)-2-Cyclohexyl-1-phenyl-3-(4,4,5,5-tetramethyl-1,3,2-dioxaborolan-2-yl)but-3-en-1-yl)-4-methoxyaniline (3f)

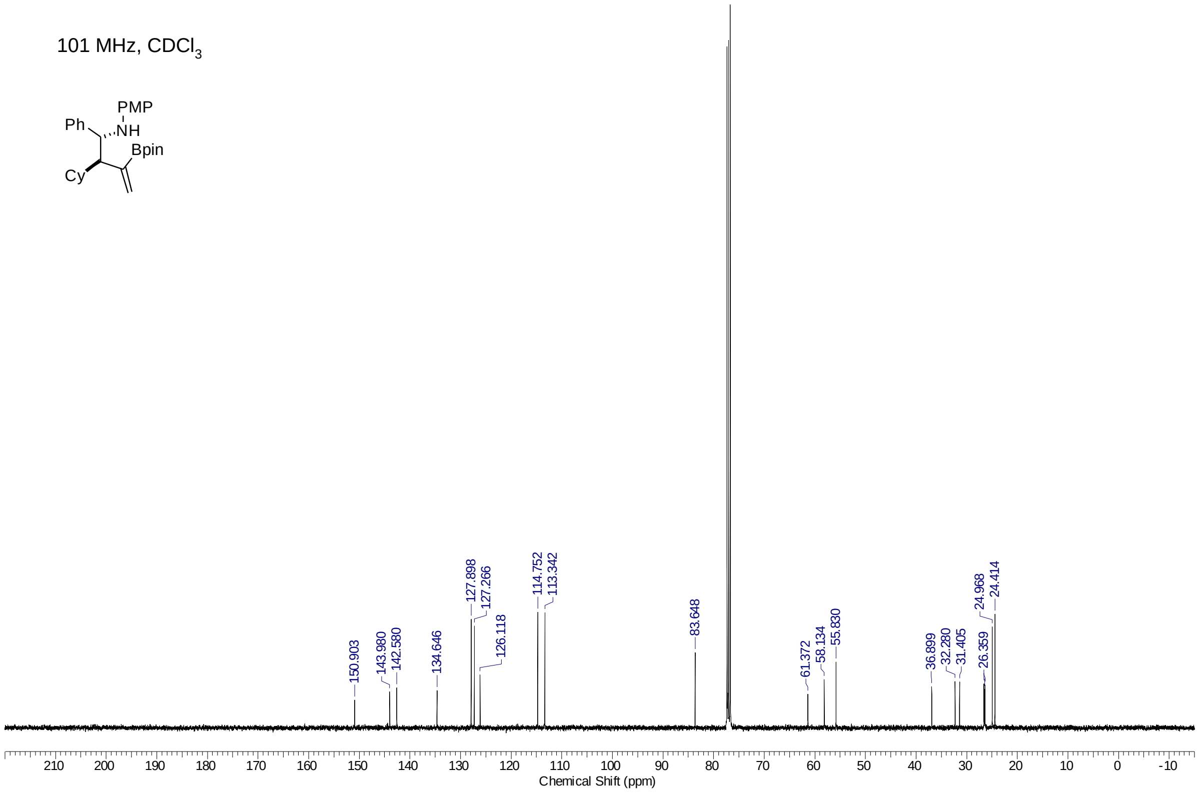

N-((1R,2R)-2-Cyclohexyl-1-(3-methoxyphenyl)-3-(4,4,5,5-tetramethyl-1,3,2-dioxaborolan-2-yl)but-3-en-1-yl)-4-methoxyaniline (3g)

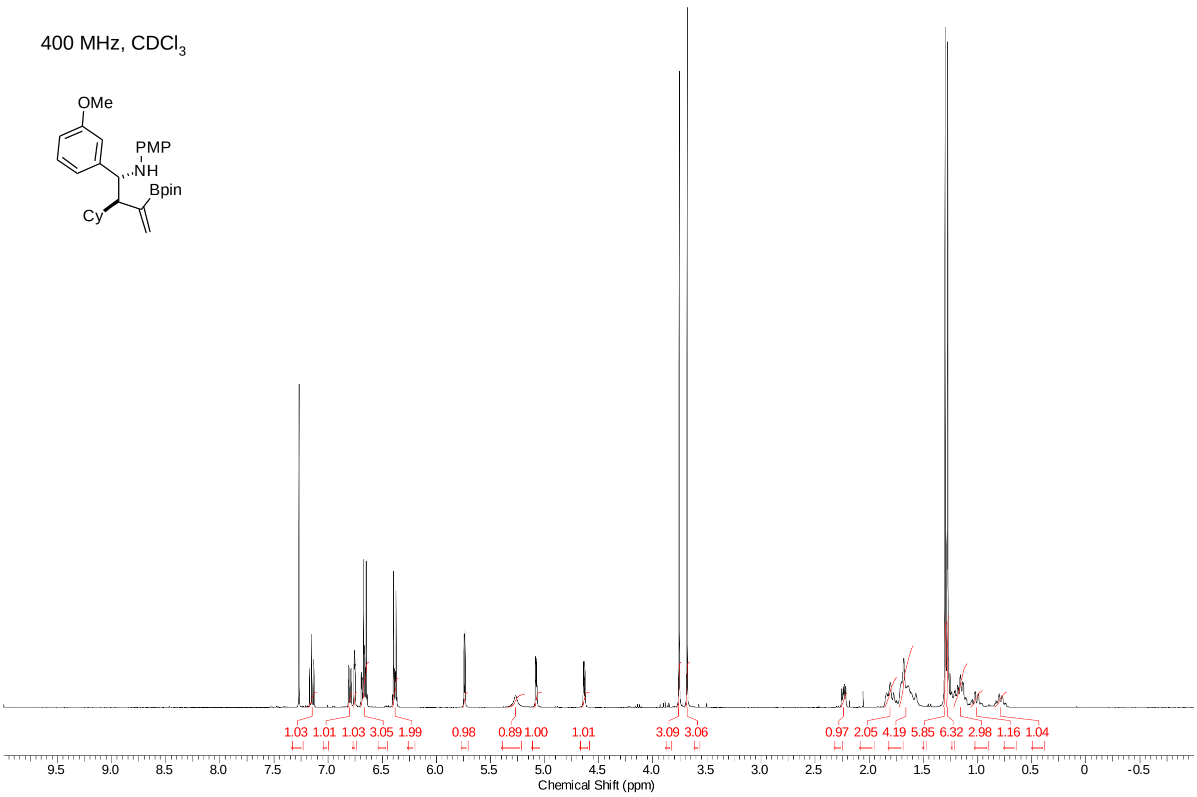

N-((1R,2R)-2-Cyclohexyl-1-(3-methoxyphenyl)-3-(4,4,5,5-tetramethyl-1,3,2-dioxaborolan-2-yl)but-3-en-1-yl)-4-methoxyaniline (3g)

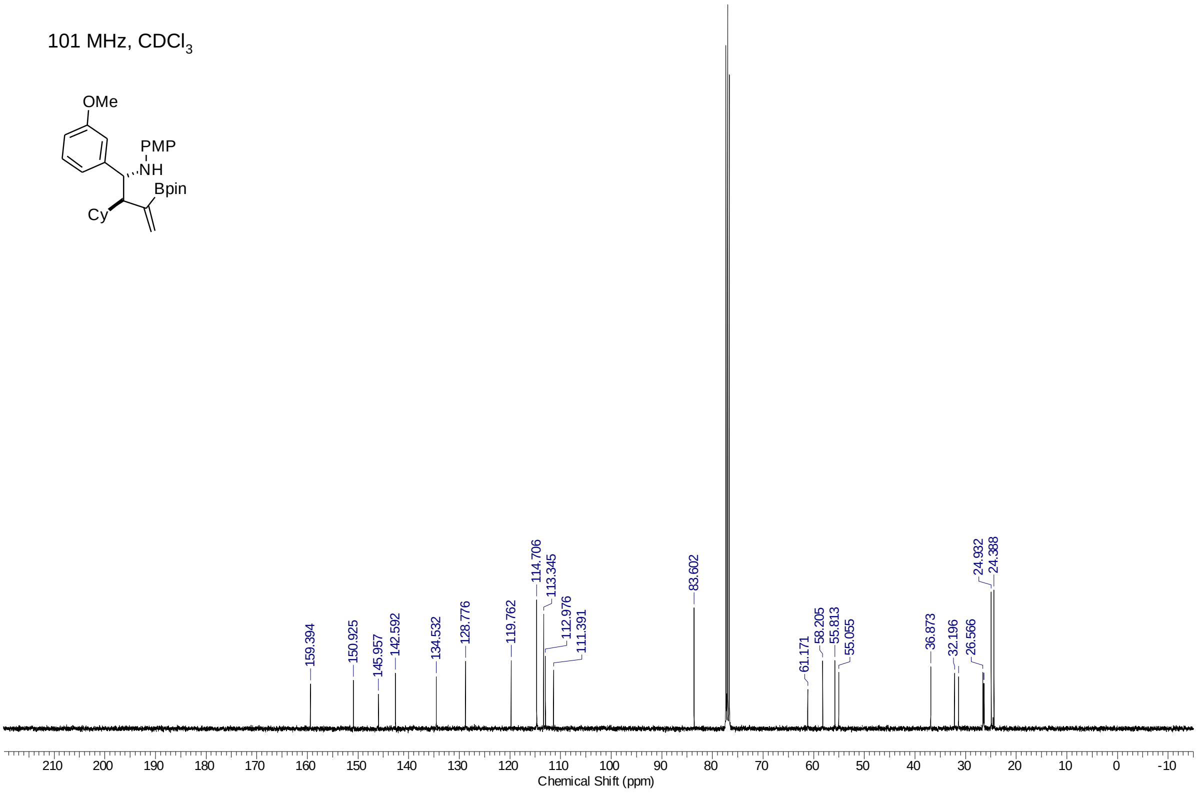

N-((1R,2R)-2-Cyclohexyl-1-(4-methoxyphenyl)-3-(4,4,5,5-tetramethyl-1,3,2-dioxaborolan-2-yl)but-3-en-1-yl)-4-methoxyaniline (3h)

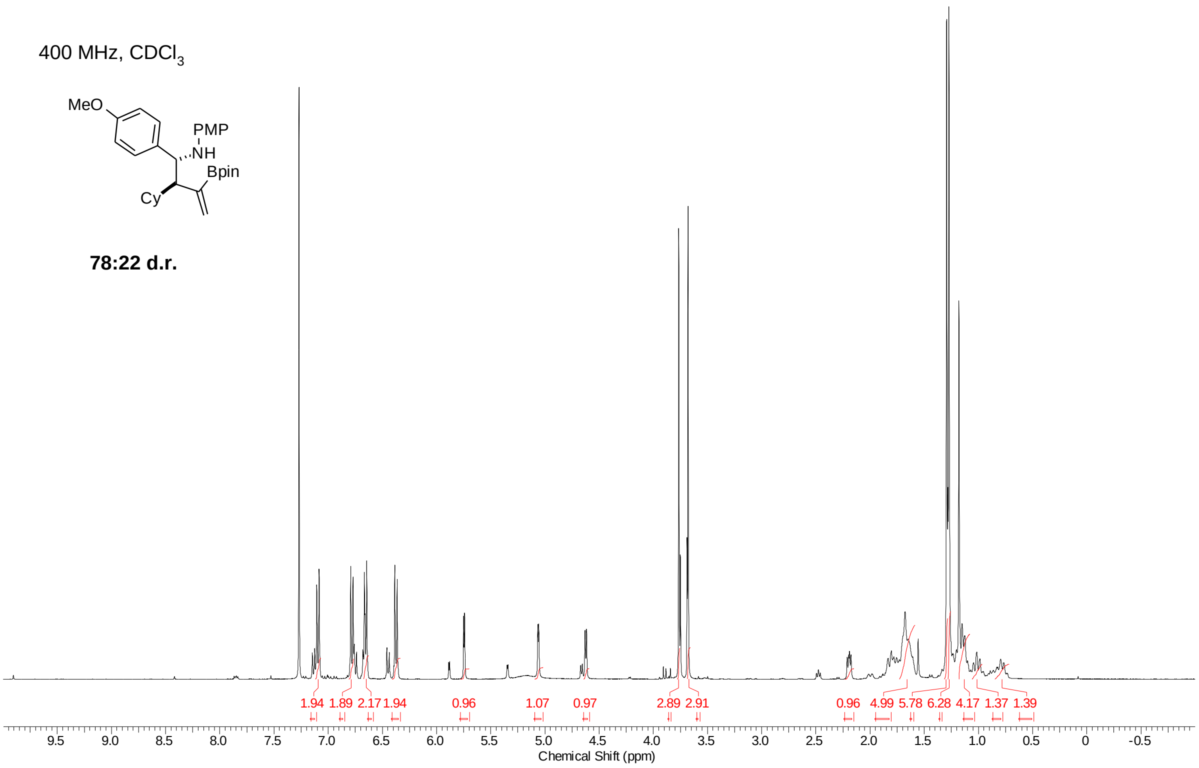

N-((1R,2R)-2-Cyclohexyl-1-(4-methoxyphenyl)-3-(4,4,5,5-tetramethyl-1,3,2-dioxaborolan-2-yl)but-3-en-1-yl)-4-methoxyaniline (3h)

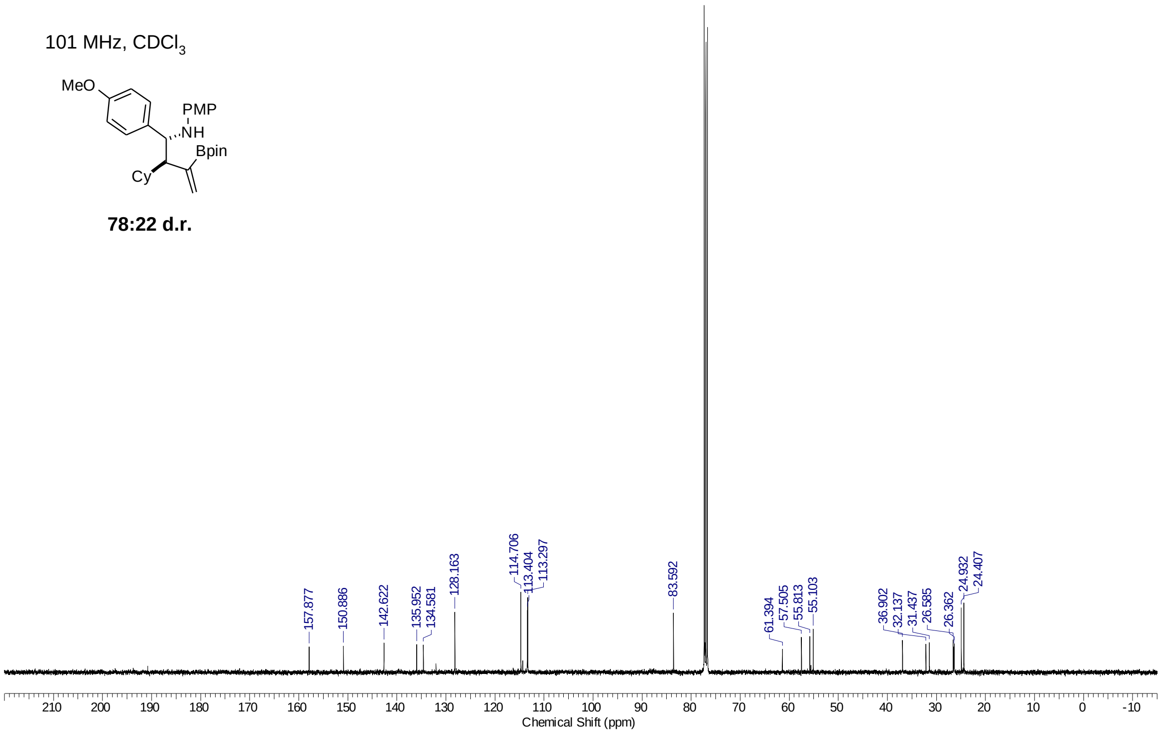

_N_-((1_R_,2_R_)-2-Cyclohexyl-3-(4,4,5,5-tetramethyl-1,3,2-dioxaborolan-2-yl)-1-(4-(trifluoromethyl)phenyl)but-3-en-1-yl)-4-methoxyaniline (3i)

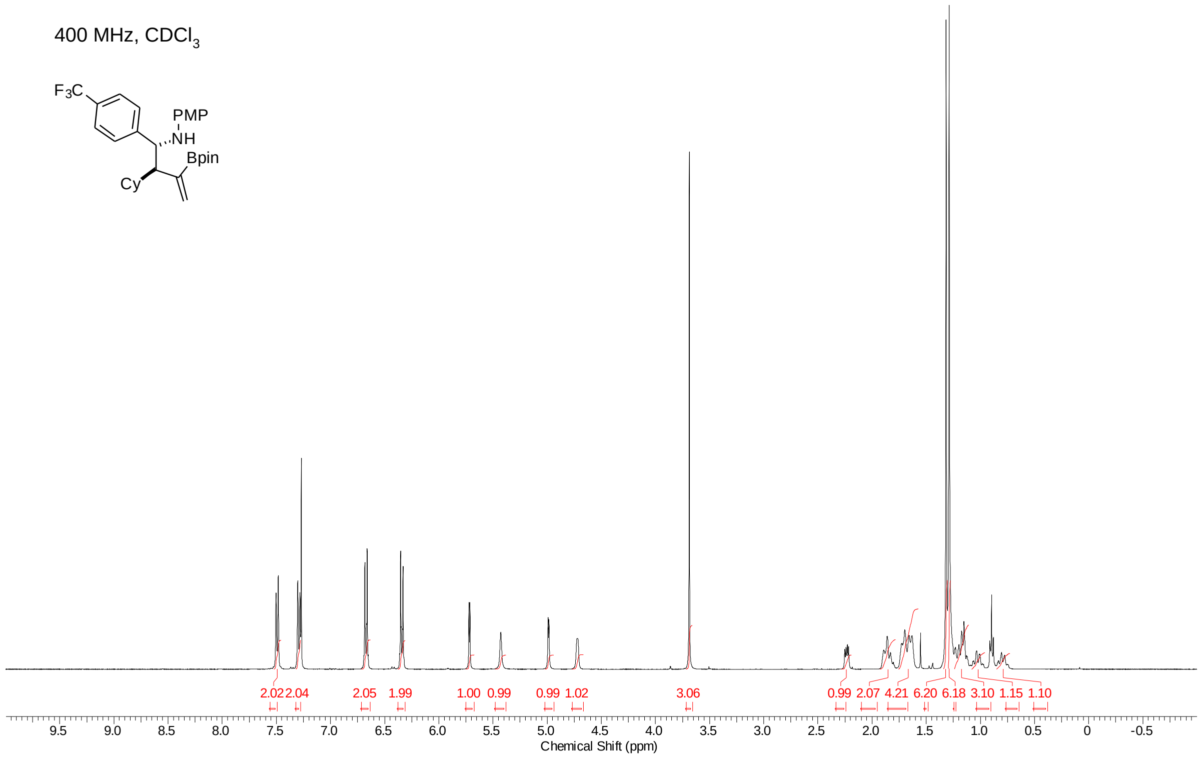

_N_-((1_R_,2_R_)-2-Cyclohexyl-3-(4,4,5,5-tetramethyl-1,3,2-dioxaborolan-2-yl)-1-(4-(trifluoromethyl)phenyl)but-3-en-1-yl)-4-methoxyaniline (3i)

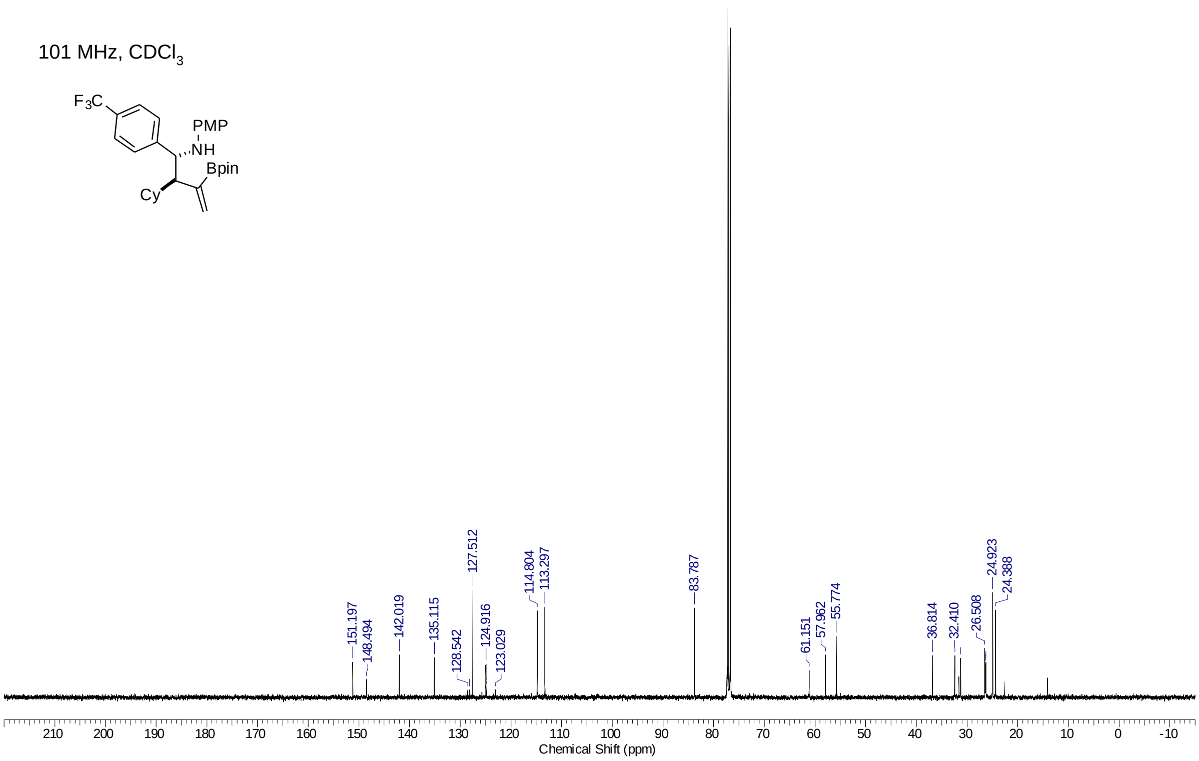

_N_-((1_R_,2_R_)-1-(4-Bromophenyl)-2-cyclohexyl-3-(4,4,5,5-tetramethyl-1,3,2-dioxaborolan-2-yl)but-3-en-1-yl)-4-methoxyaniline (3j)

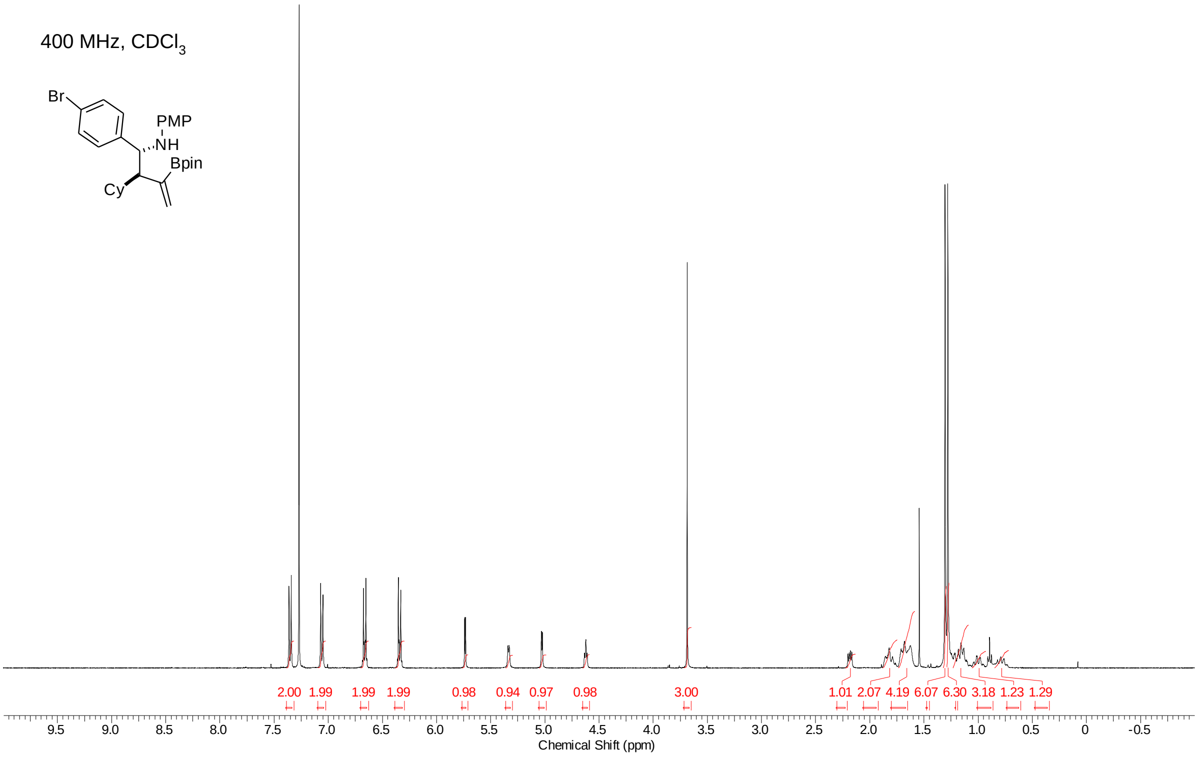

_N_-((1_R_,2_R_)-1-(4-Bromophenyl)-2-cyclohexyl-3-(4,4,5,5-tetramethyl-1,3,2-dioxaborolan-2-yl)but-3-en-1-yl)-4-methoxyaniline (3j)

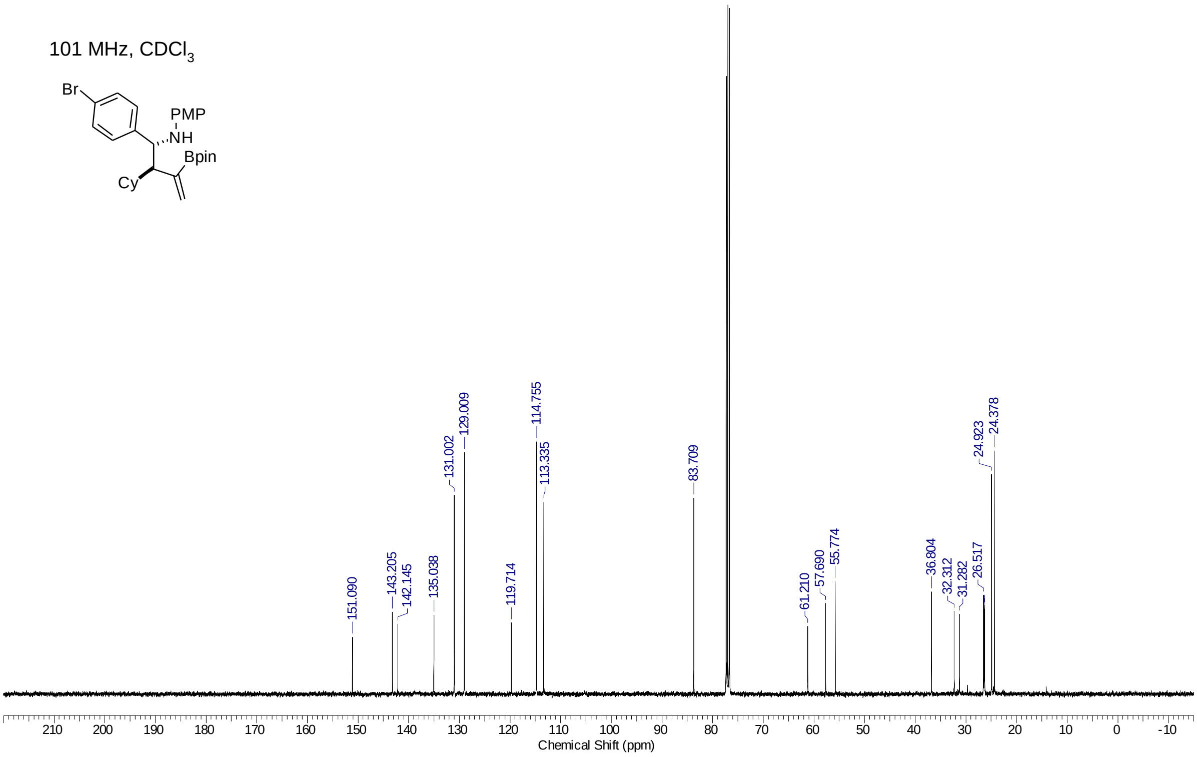

_N_-((1_R_,2_R_)-2-Cyclohexyl-1-(furan-2-yl)-3-(4,4,5,5-tetramethyl-1,3,2-dioxaborolan-2-yl)but-3-en-1-yl)-4-methoxyaniline (3k)

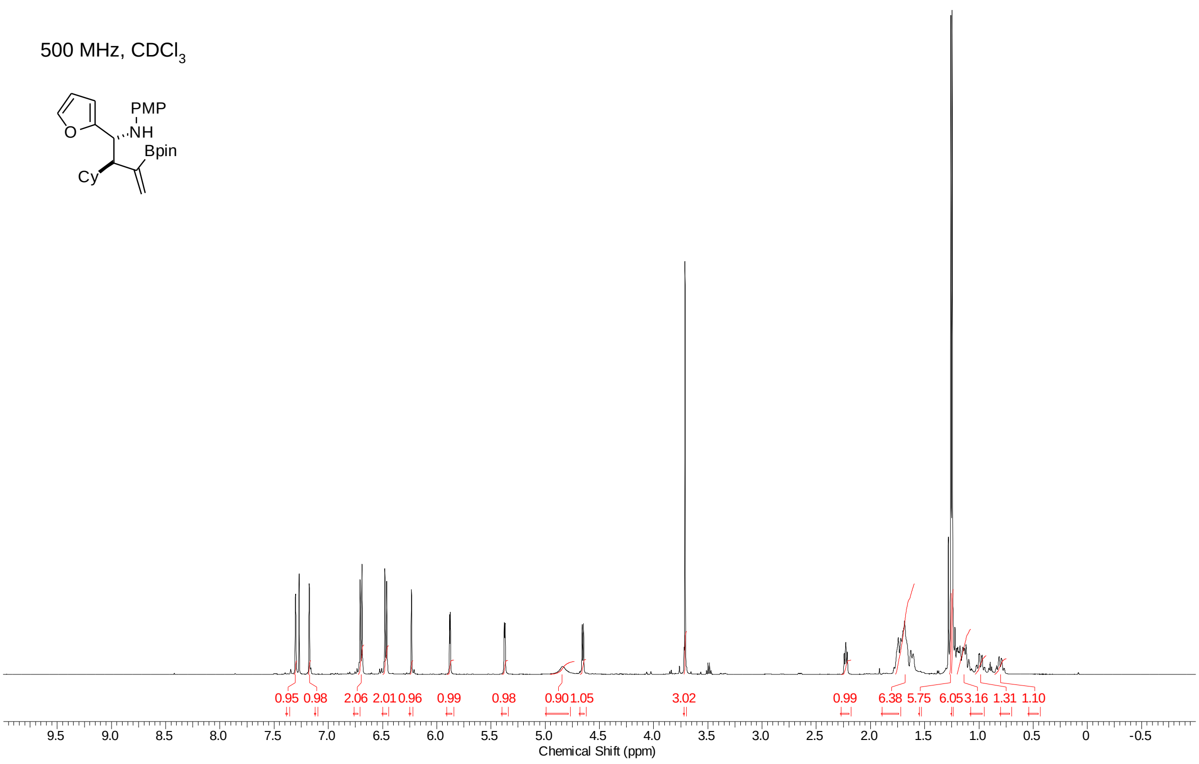

_N_-((1_R_,2_R_)-2-Cyclohexyl-1-(furan-2-yl)-3-(4,4,5,5-tetramethyl-1,3,2-dioxaborolan-2-yl)but-3-en-1-yl)-4-methoxyaniline (3k)

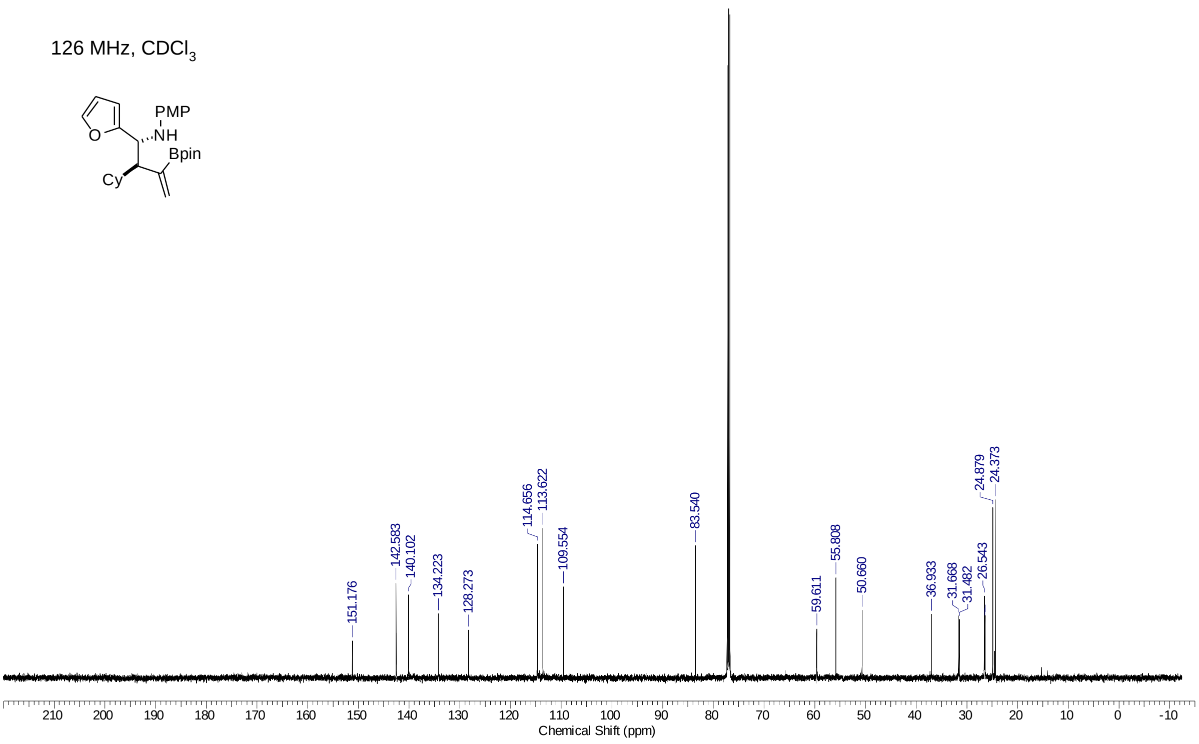

N-((1*R*,2*R*)-2-Cyclohexyl-1-(furan-3-yl)-3-(4,4,5,5-tetramethyl-1,3,2-dioxaborolan-2-yl)but-3-en-1-yl)-4-methoxyaniline (3l)

400 MHz, $CDCl_3$

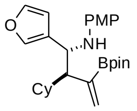

81:19 d.r.

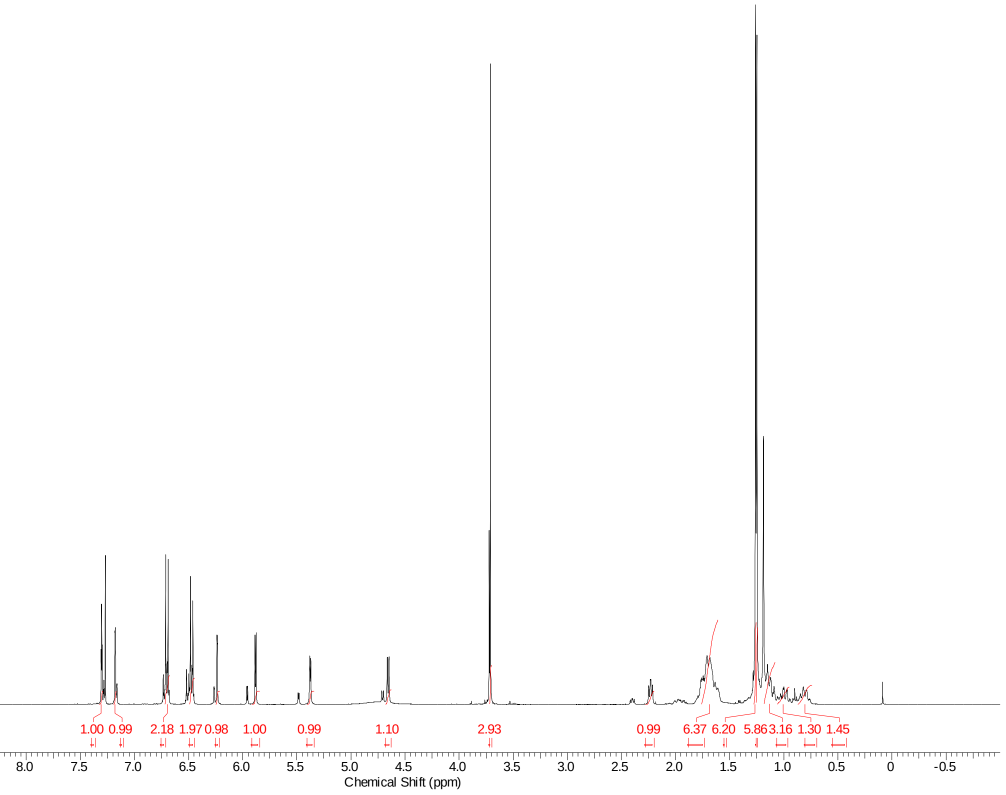

N-((1*R*,2*R*)-2-Cyclohexyl-1-(furan-3-yl)-3-(4,4,5,5-tetramethyl-1,3,2-dioxaborolan-2-yl)but-3-en-1-yl)-4-methoxyaniline (3l)

101 MHz, $CDCl_3$

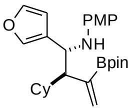

81:19 d.r.

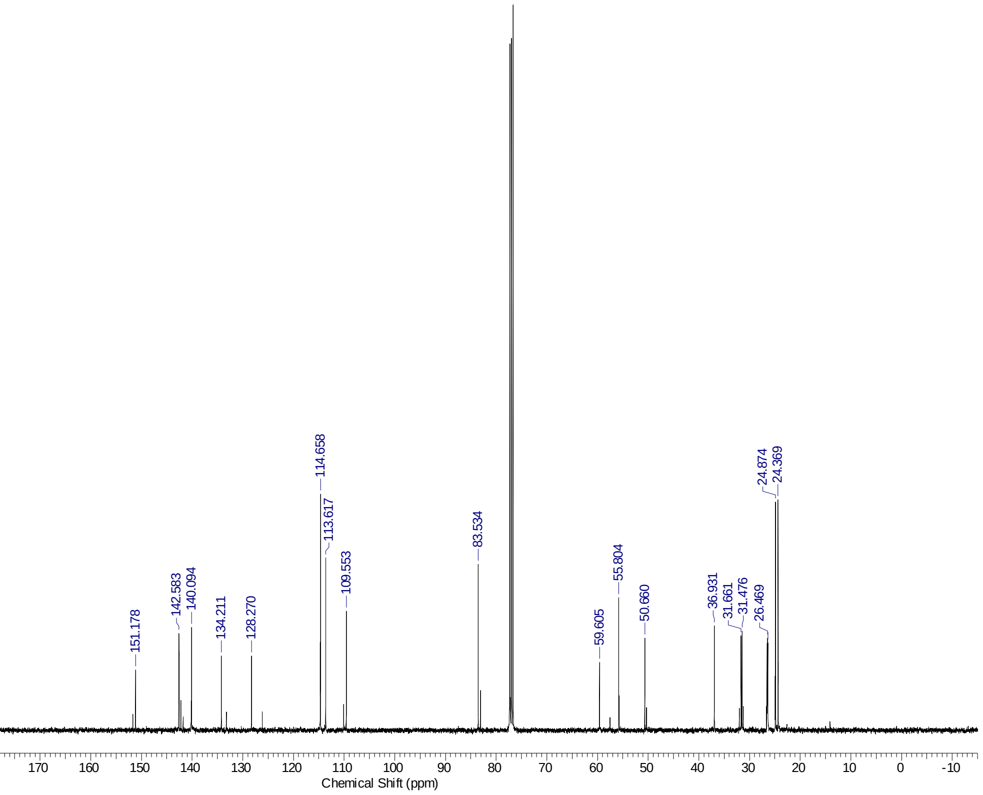

4-Methoxy-*N*-((1*R*,2*R*)-2-(1-(4,4,5,5-tetramethyl-1,3,2-dioxaborolan-2-yl)vinyl)-1-(*o*-tolyl)decyl)aniline (3m)

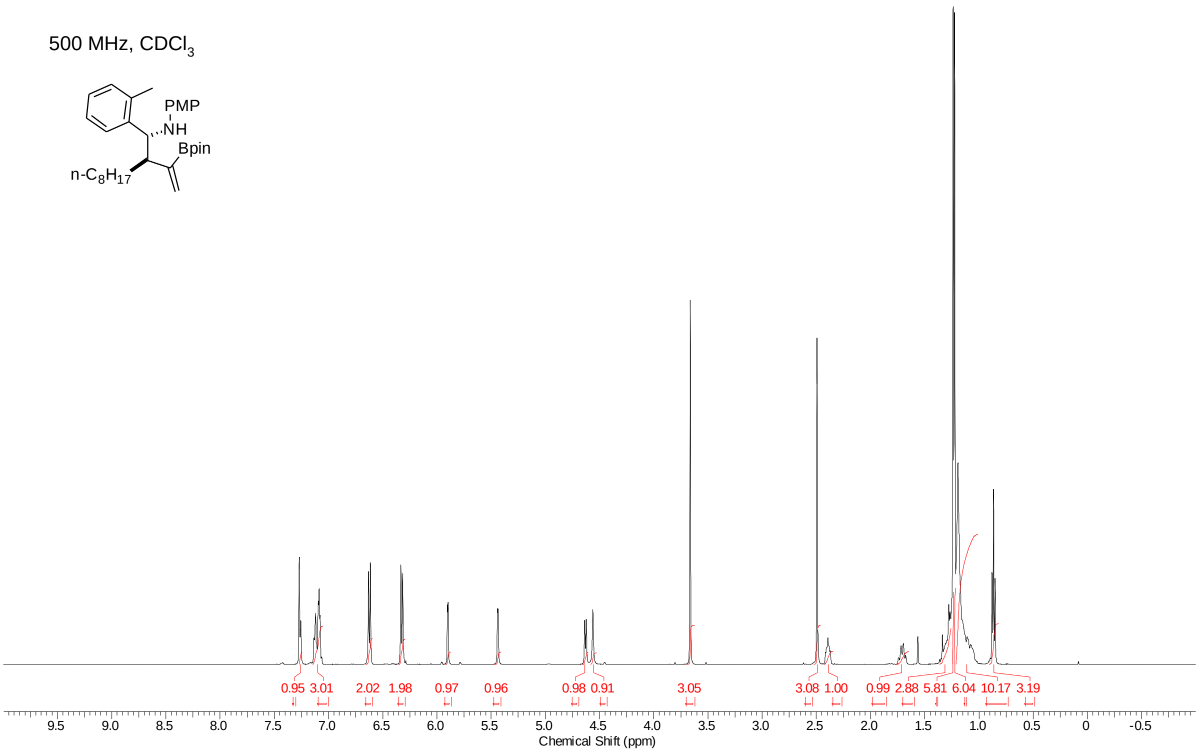

4-Methoxy-*N*-((1*R*,2*R*)-2-(1-(4,4,5,5-tetramethyl-1,3,2-dioxaborolan-2-yl)vinyl)-1-(*o*-tolyl)decyl)aniline (3m)

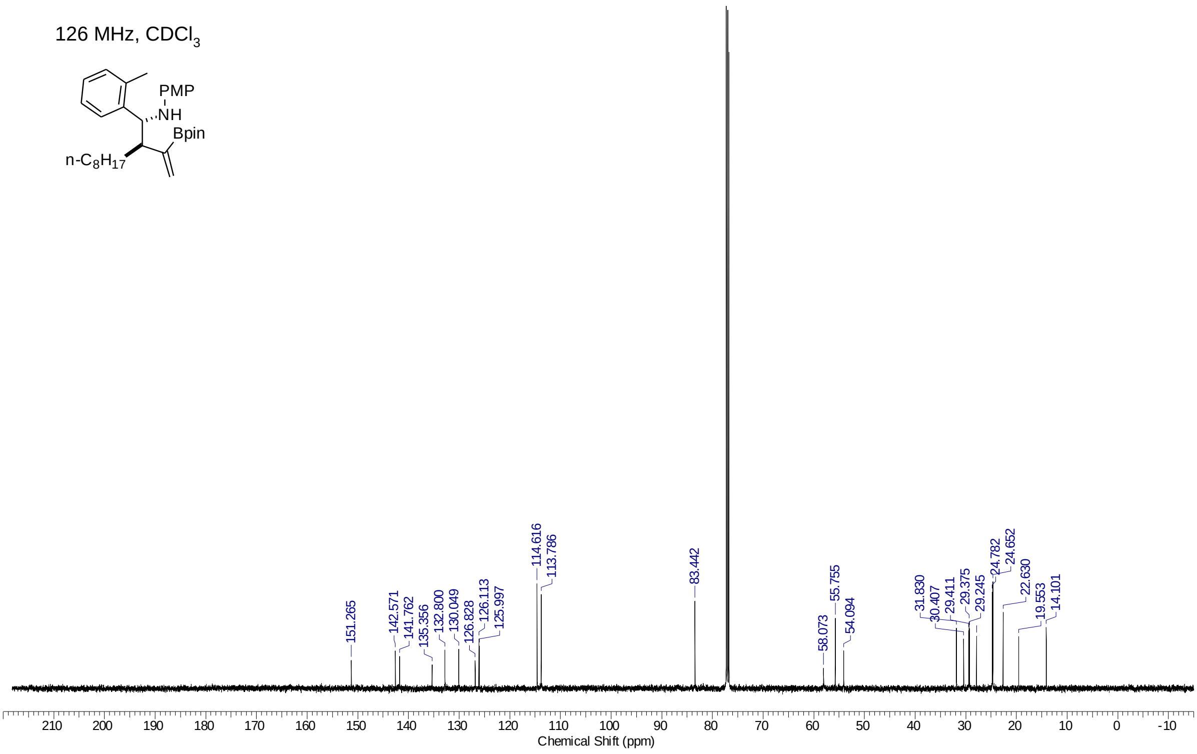

N-((1R,2R)-6-((tert-Butyldimethylsilyl)oxy)-2-(1-(4,4,5,5-tetramethyl-1,3,2-dioxaborolan-2-yl)vinyl)-1-(o-tolyl)hexyl)-4-methoxyaniline (3n)

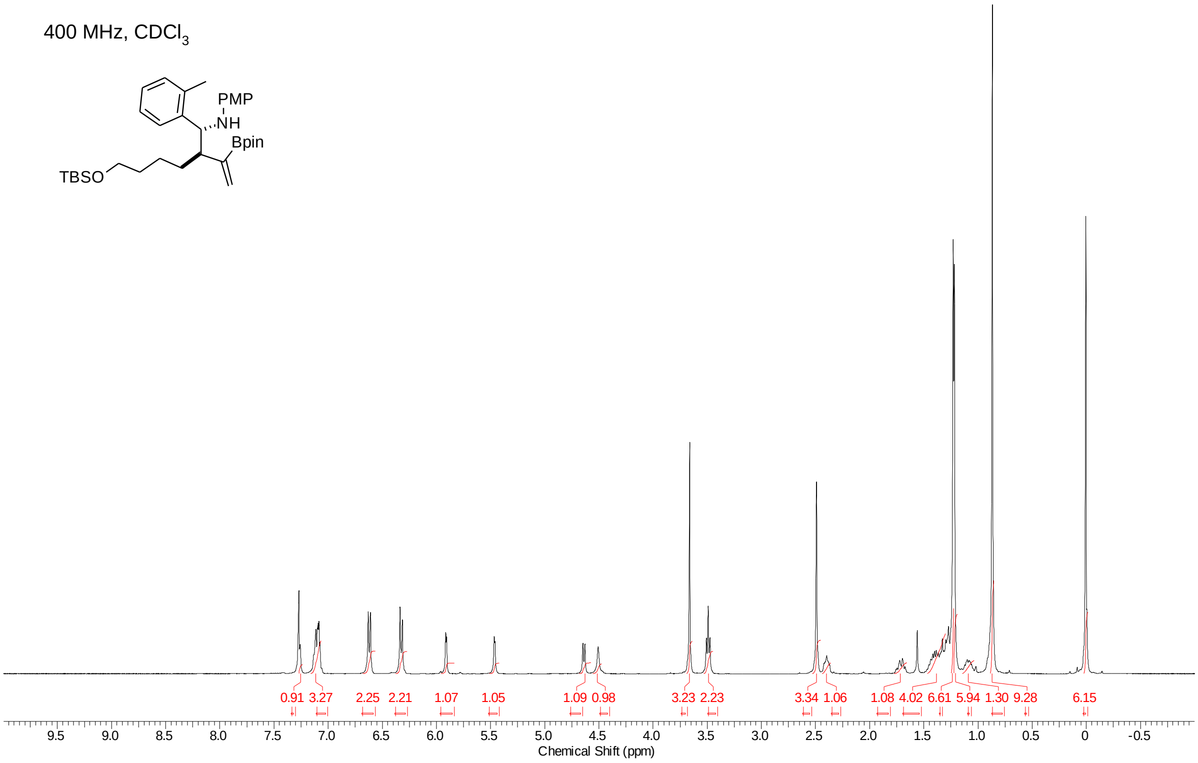

N-((1R,2R)-6-((tert-Butyldimethylsilyl)oxy)-2-(1-(4,4,5,5-tetramethyl-1,3,2-dioxaborolan-2-yl)vinyl)-1-(o-tolyl)hexyl)-4-methoxyaniline (3n)

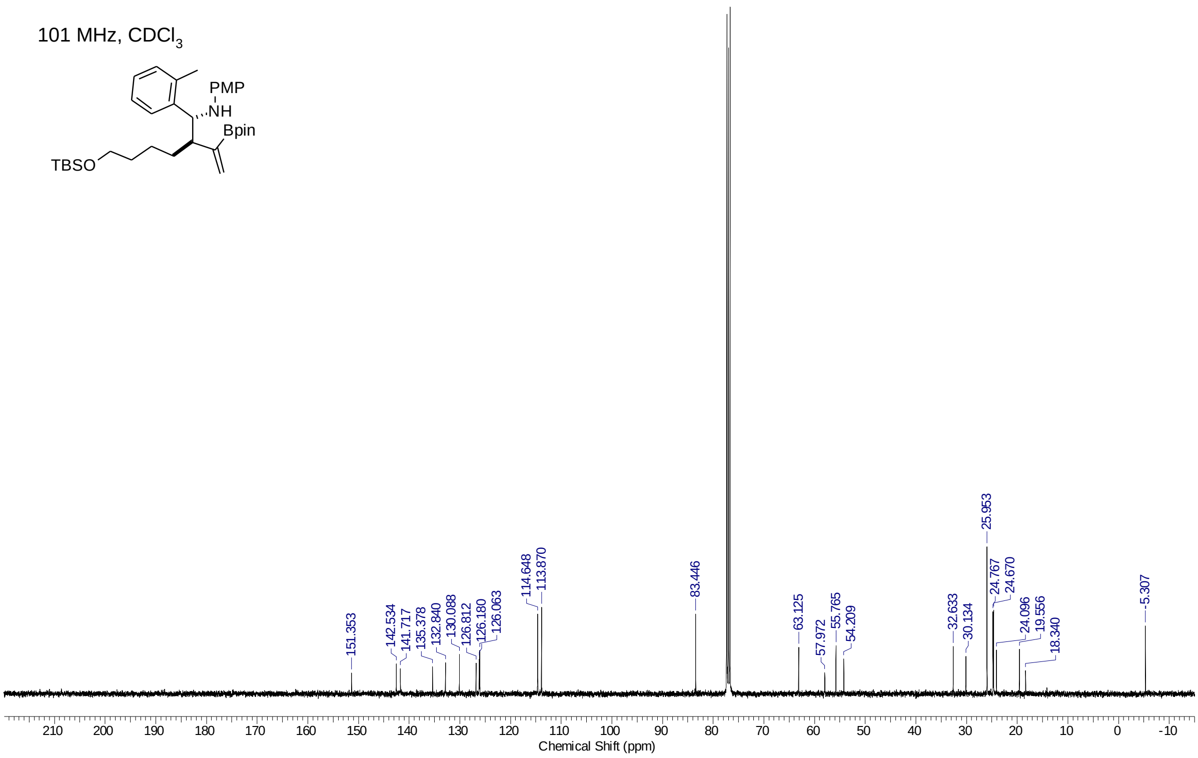

(*R*)-5-((*R*)-((4-Methoxyphenyl)amino)(*o*-tolyl)methyl)-6-(4,4,5,5-tetramethyl-1,3,2-dioxaborolan-2-yl)hept-6-en-1-ol (3o)

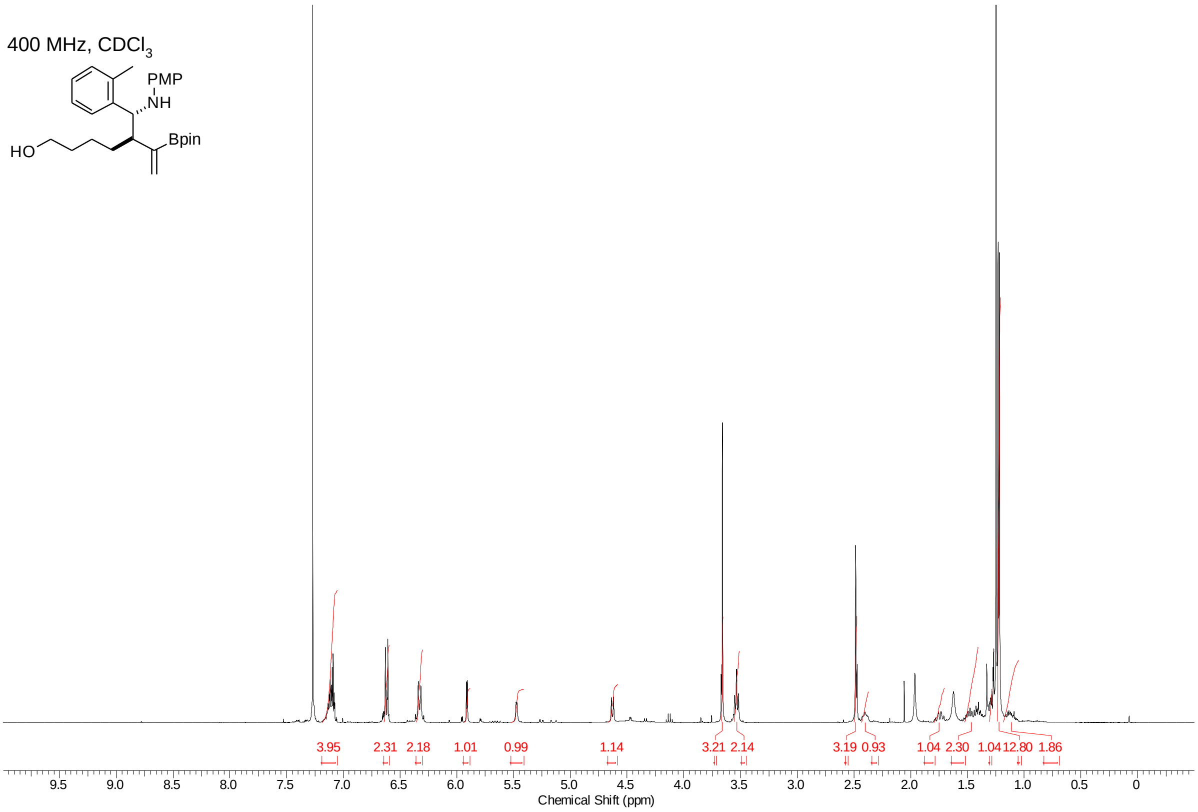

(*R*)-5-((*R*)-((4-Methoxyphenyl)amino)(*o*-tolyl)methyl)-6-(4,4,5,5-tetramethyl-1,3,2-dioxaborolan-2-yl)hept-6-en-1-ol (3o)

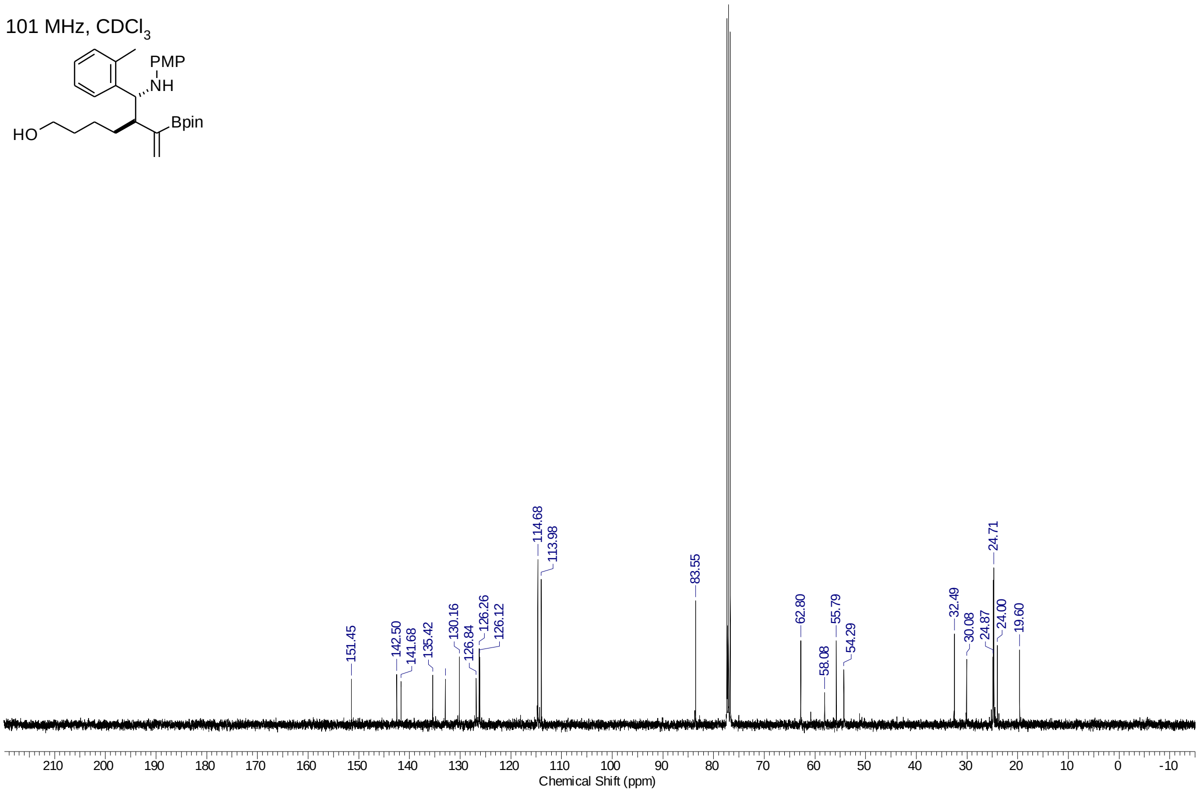

4-Methoxy-*N*-((1*R*,2*R*)-2-phenethyl-3-(4,4,5,5-tetramethyl-1,3,2-dioxaborolan-2-yl)-1-(*o*-tolyl)but-3-en-1-yl)aniline (3p)

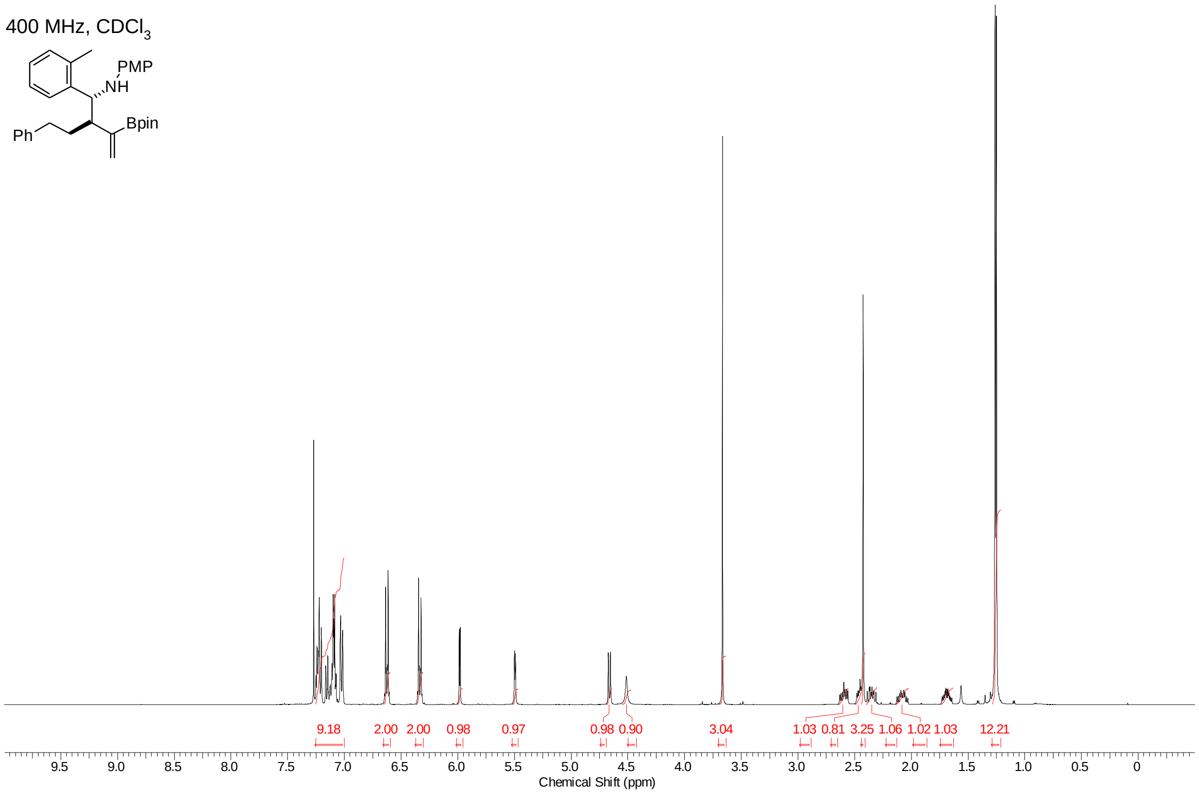

4-Methoxy-*N*-((1*R*,2*R*)-2-phenethyl-3-(4,4,5,5-tetramethyl-1,3,2-dioxaborolan-2-yl)-1-(*o*-tolyl)but-3-en-1-yl)aniline (3p)

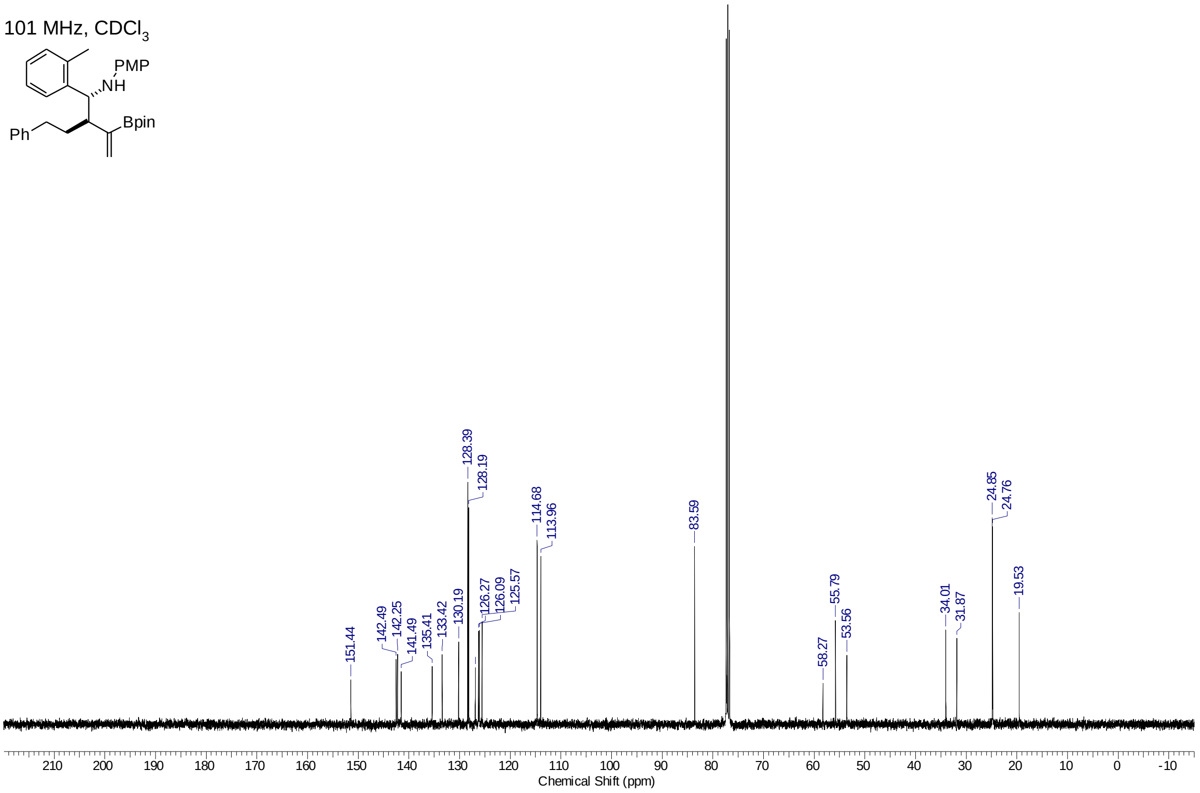

4-Methoxy-*N*-((1*R*,2*S*)-2-phenyl-3-(4,4,5,5-tetramethyl-1,3,2-dioxaborolan-2-yl)-1-(*o*-tolyl)but-3-en-1-yl)aniline (3q)

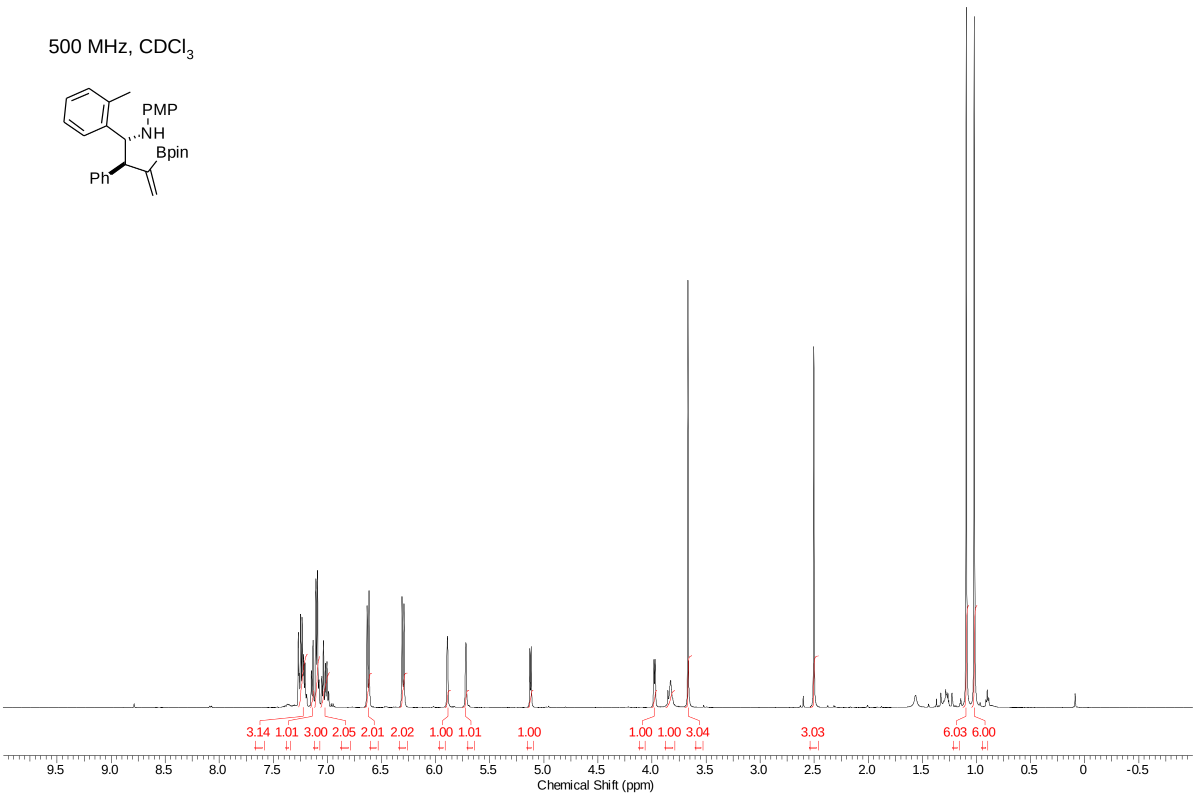

4-Methoxy-*N*-((1*R*,2*S*)-2-phenyl-3-(4,4,5,5-tetramethyl-1,3,2-dioxaborolan-2-yl)-1-(*o*-tolyl)but-3-en-1-yl)aniline (3q)

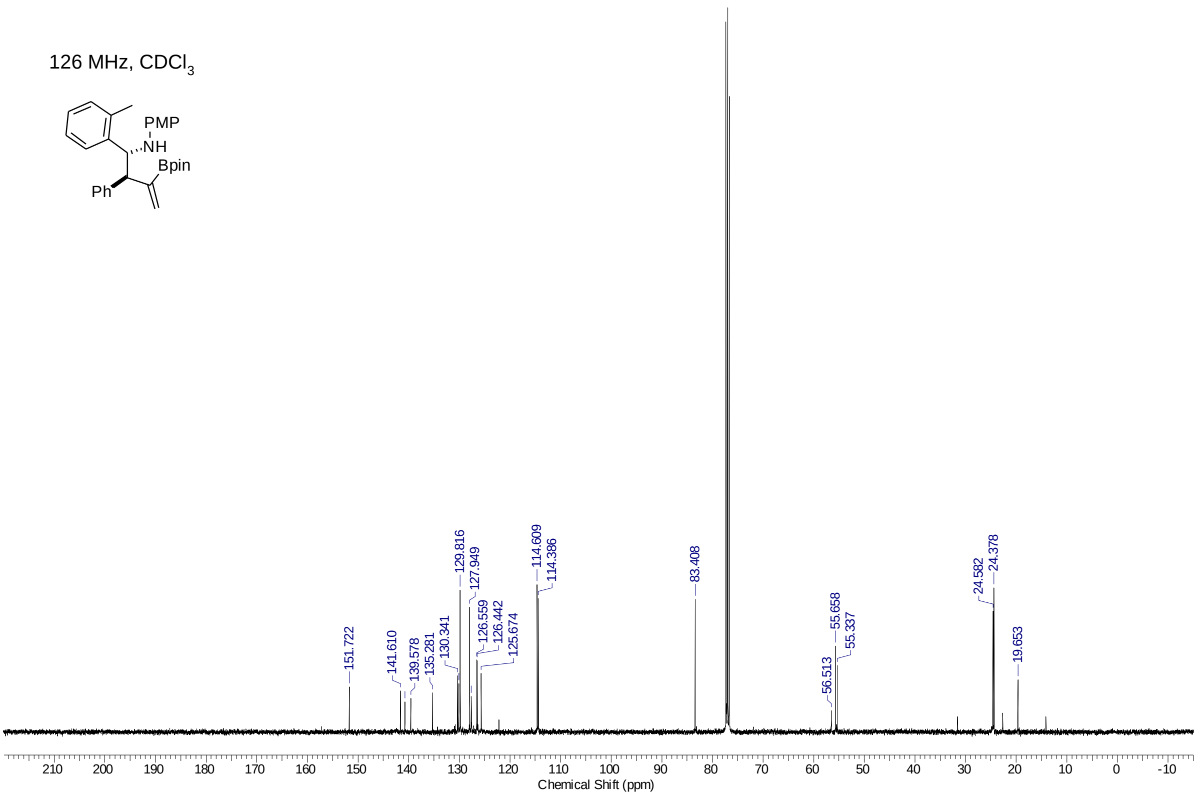

(*R*)-4-Methoxy-*N*-((1-(1-(4,4,5,5-tetramethyl-1,3,2-dioxaborolan-2-yl)vinyl)cyclohexyl)(*o*-tolyl)methyl)aniline (3r)

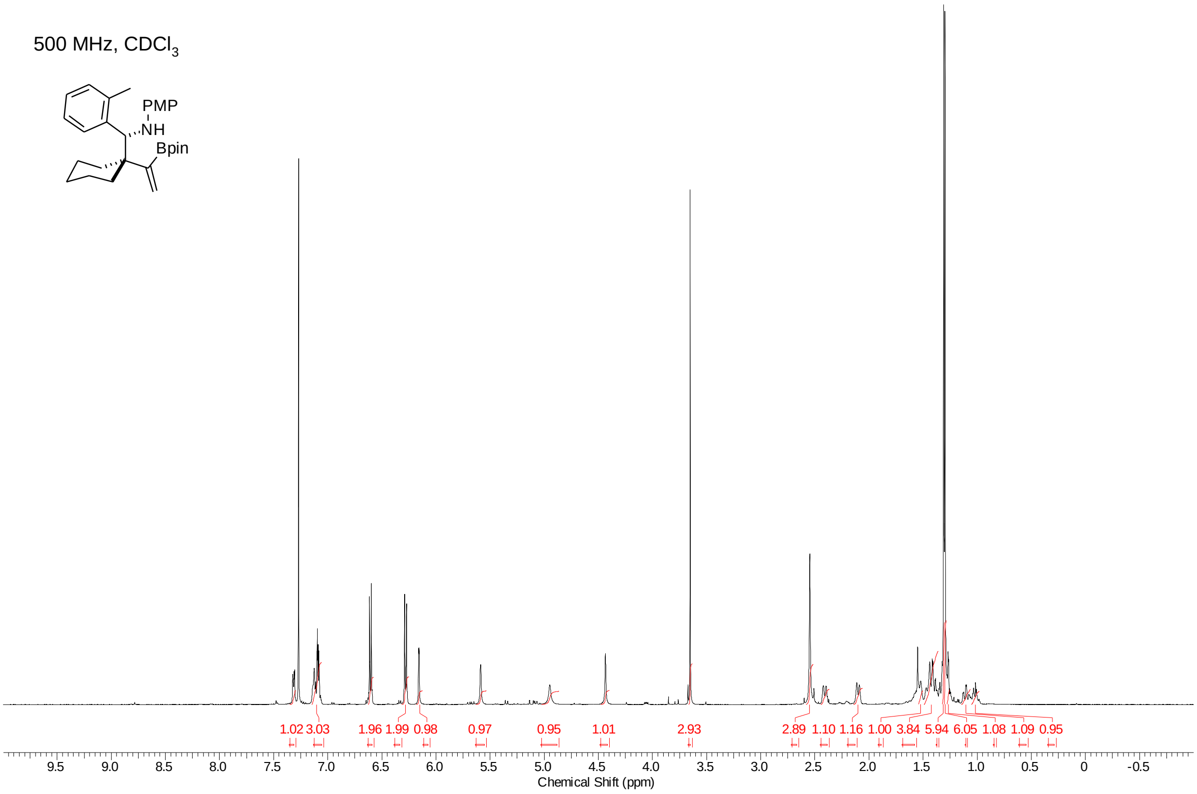

(*R*)-4-Methoxy-*N*-((1-(1-(4,4,5,5-tetramethyl-1,3,2-dioxaborolan-2-yl)vinyl)cyclohexyl)(*o*-tolyl)methyl)aniline (3r)

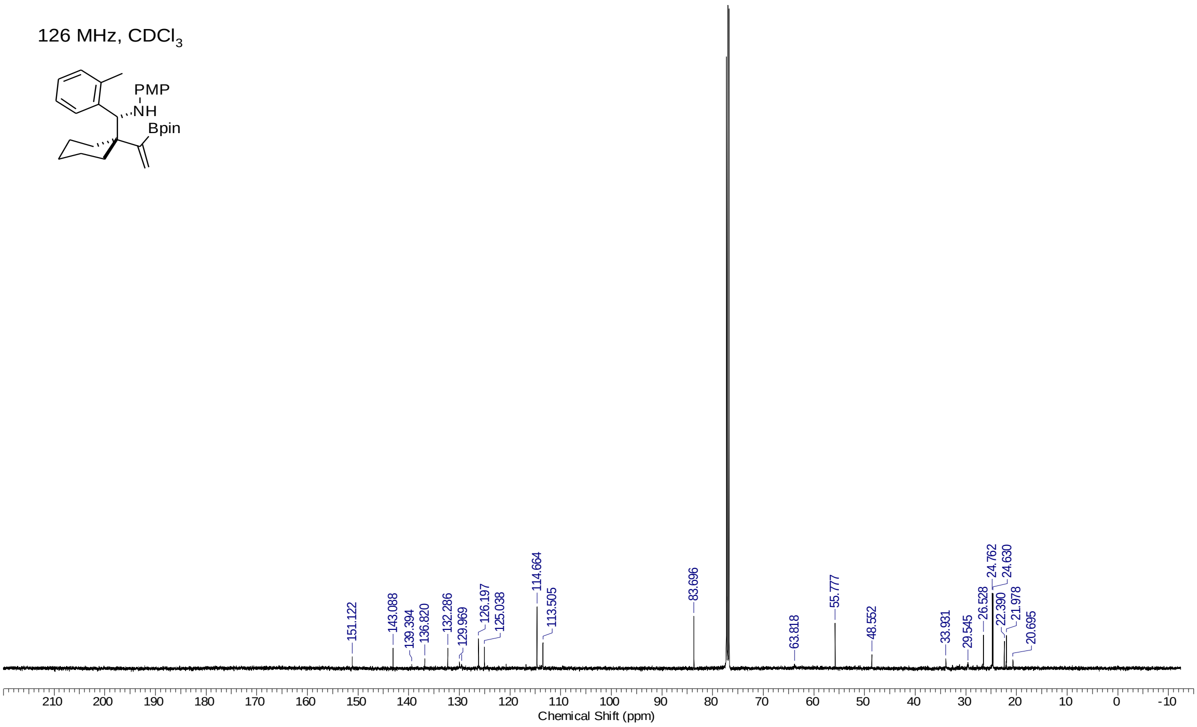

(*R*)-*N*-(Furan-2-yl(1-(1-(4,4,5,5-tetramethyl-1,3,2-dioxaborolan-2-yl)vinyl)cyclohexyl)methyl)-4-methoxyaniline (3s)

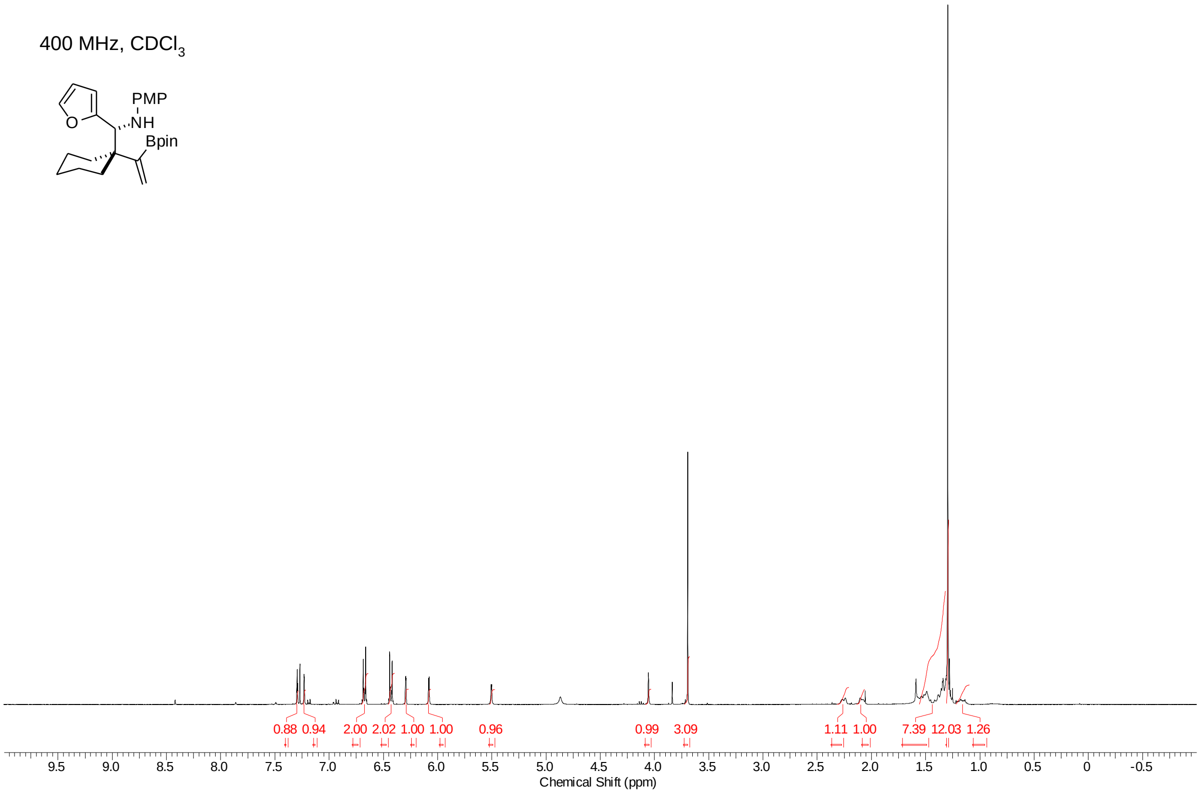

(*R*)-*N*-(Furan-2-yl(1-(1-(4,4,5,5-tetramethyl-1,3,2-dioxaborolan-2-yl)vinyl)cyclohexyl)methyl)-4-methoxyaniline (3s)

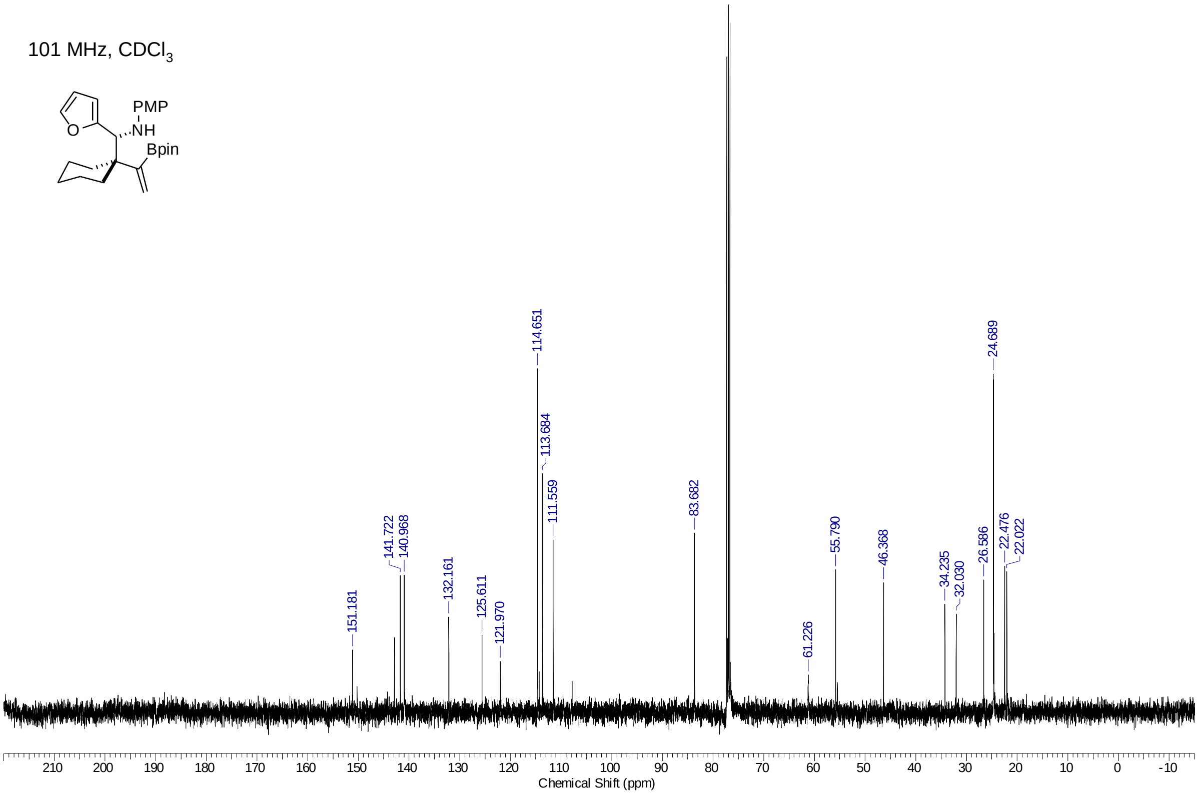

(*R*)-4-Methoxy-*N*-((1-(1-(4,4,5,5-tetramethyl-1,3,2-dioxaborolan-2-yl)vinyl)cyclohexyl)(thiophen-2-yl)methyl)aniline (3t)

400 MHz, $CDCl_3$

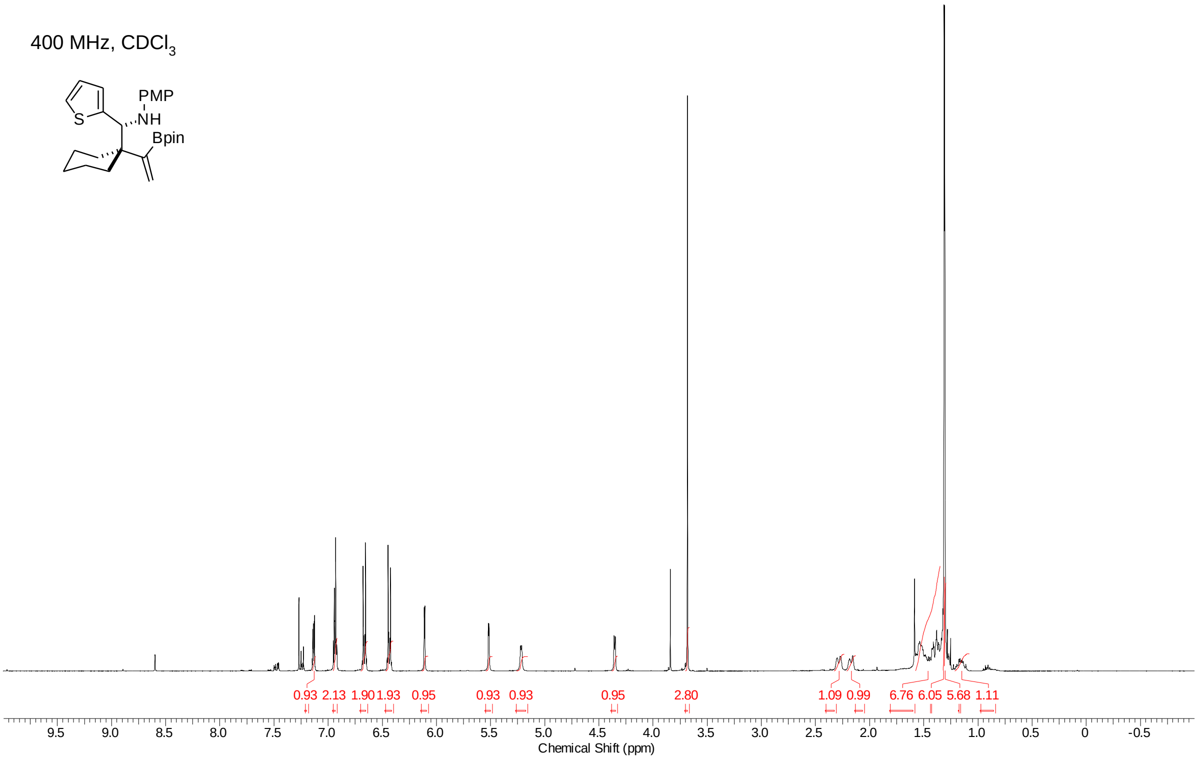

(*R*)-4-Methoxy-*N*-((1-(1-(4,4,5,5-tetramethyl-1,3,2-dioxaborolan-2-yl)vinyl)cyclohexyl)(thiophen-2-yl)methyl)aniline (3t)

101 MHz, $CDCl_3$

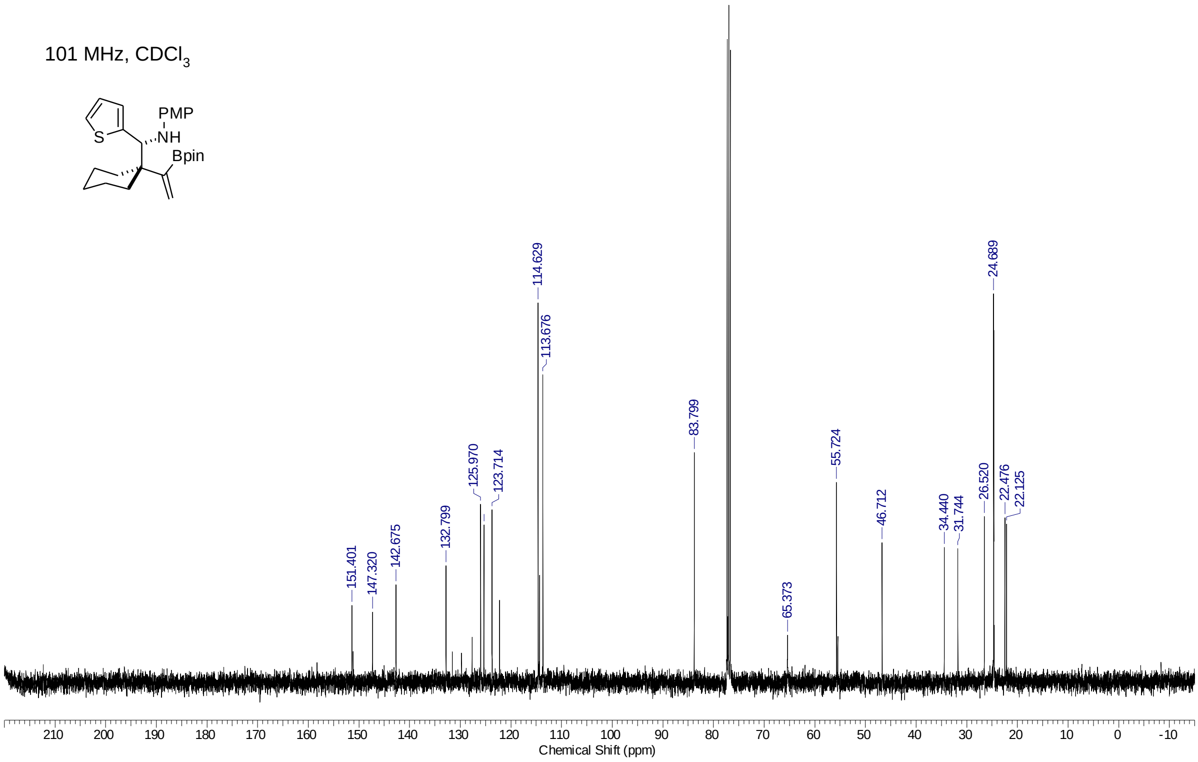

(*R*)-*N*-(2,2-Dimethyl-3-(4,4,5,5-tetramethyl-1,3,2-dioxaborolan-2-yl)-1-(*o*-tolyl)but-3-en-1-yl)-4-methoxyaniline (3u)

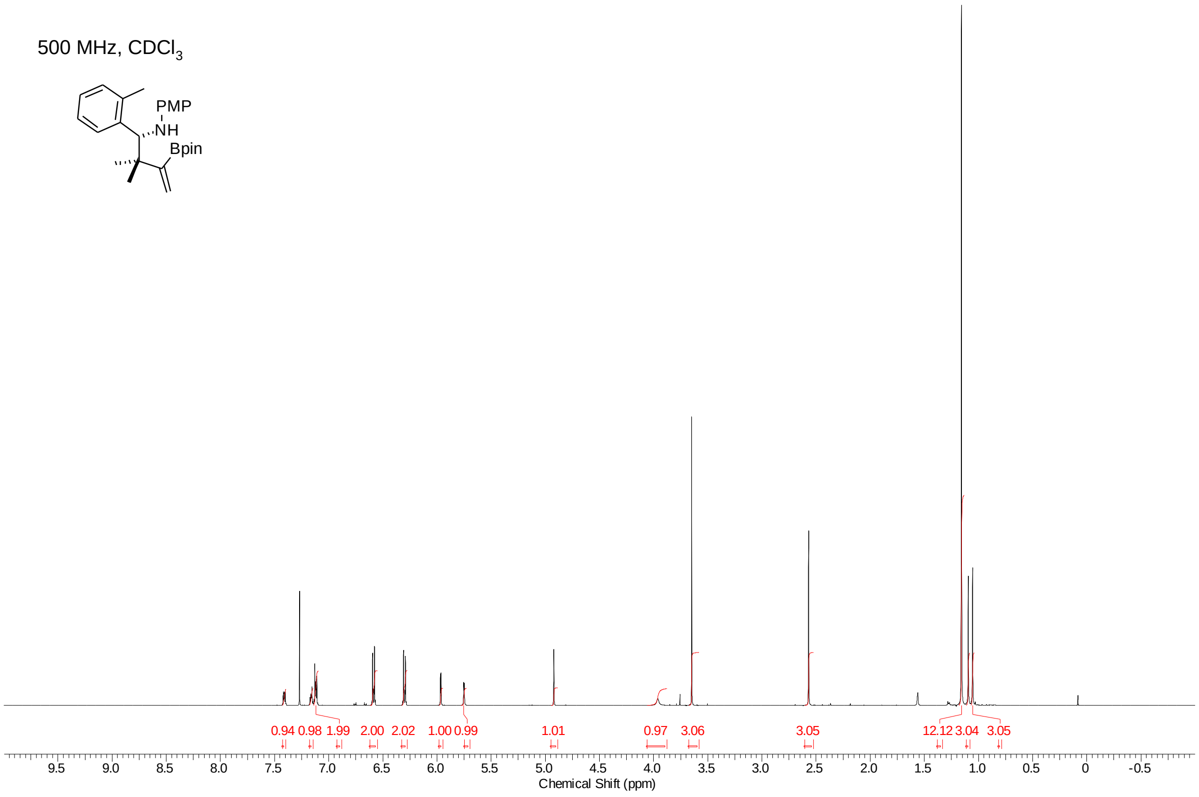

(*R*)-*N*-(2,2-Dimethyl-3-(4,4,5,5-tetramethyl-1,3,2-dioxaborolan-2-yl)-1-(*o*-tolyl)but-3-en-1-yl)-4-methoxyaniline (3u)

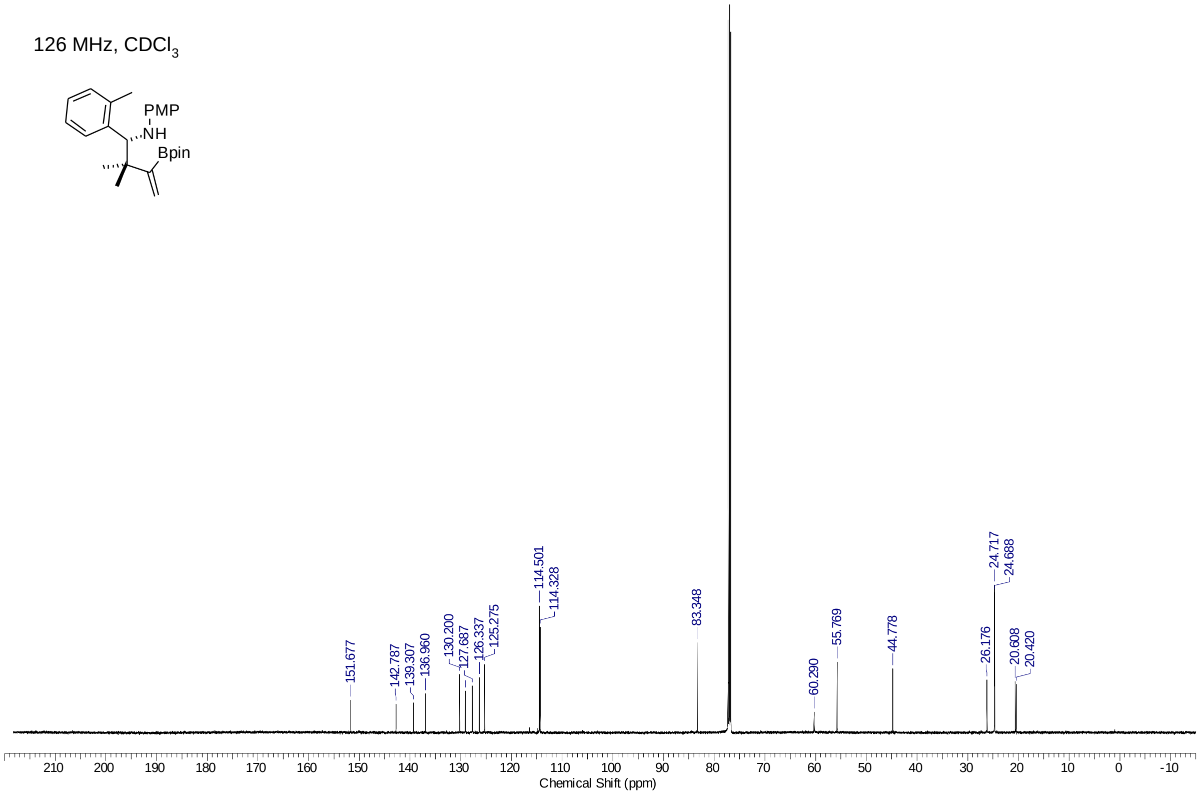

(*R*)-*N*-(1-(Furan-2-yl)-2,2-dimethyl-3-(4,4,5,5-tetramethyl-1,3,2-dioxaborolan-2-yl)but-3-en-1-yl)-4-methoxyaniline (3v)

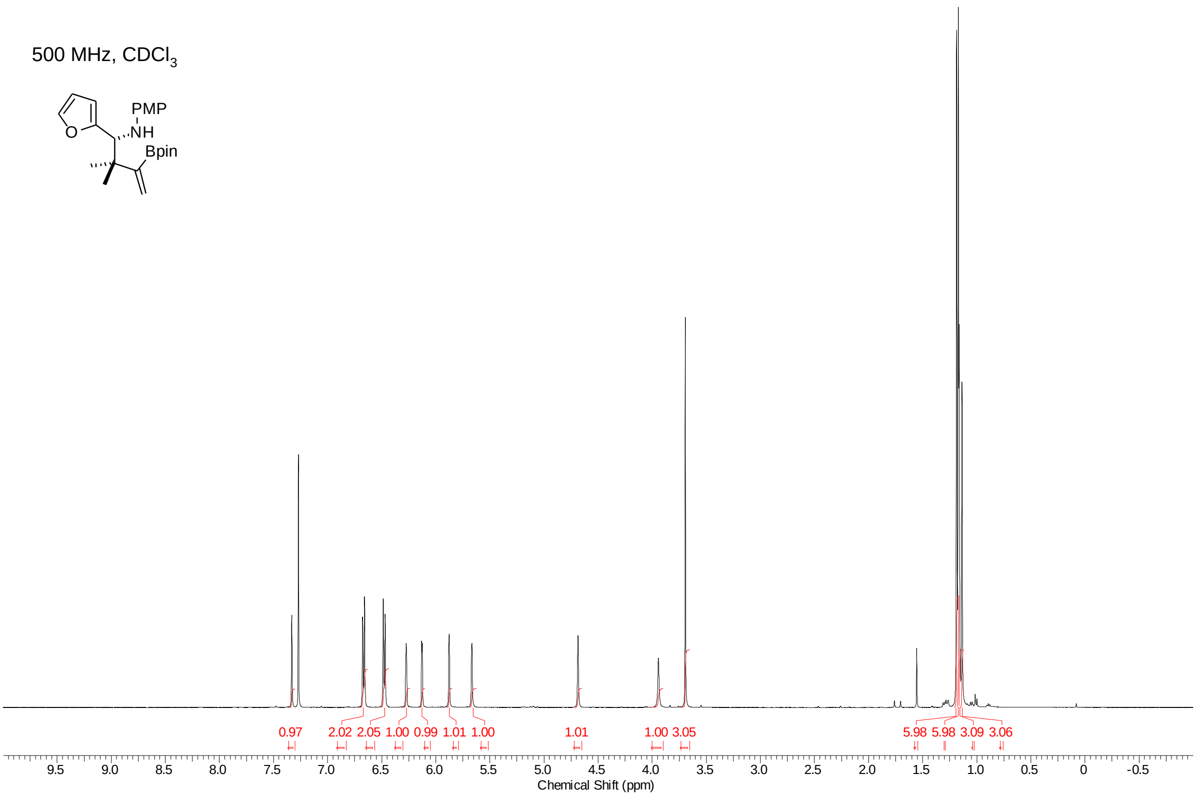

(*R*)-*N*-(1-(Furan-2-yl)-2,2-dimethyl-3-(4,4,5,5-tetramethyl-1,3,2-dioxaborolan-2-yl)but-3-en-1-yl)-4-methoxyaniline (3v)

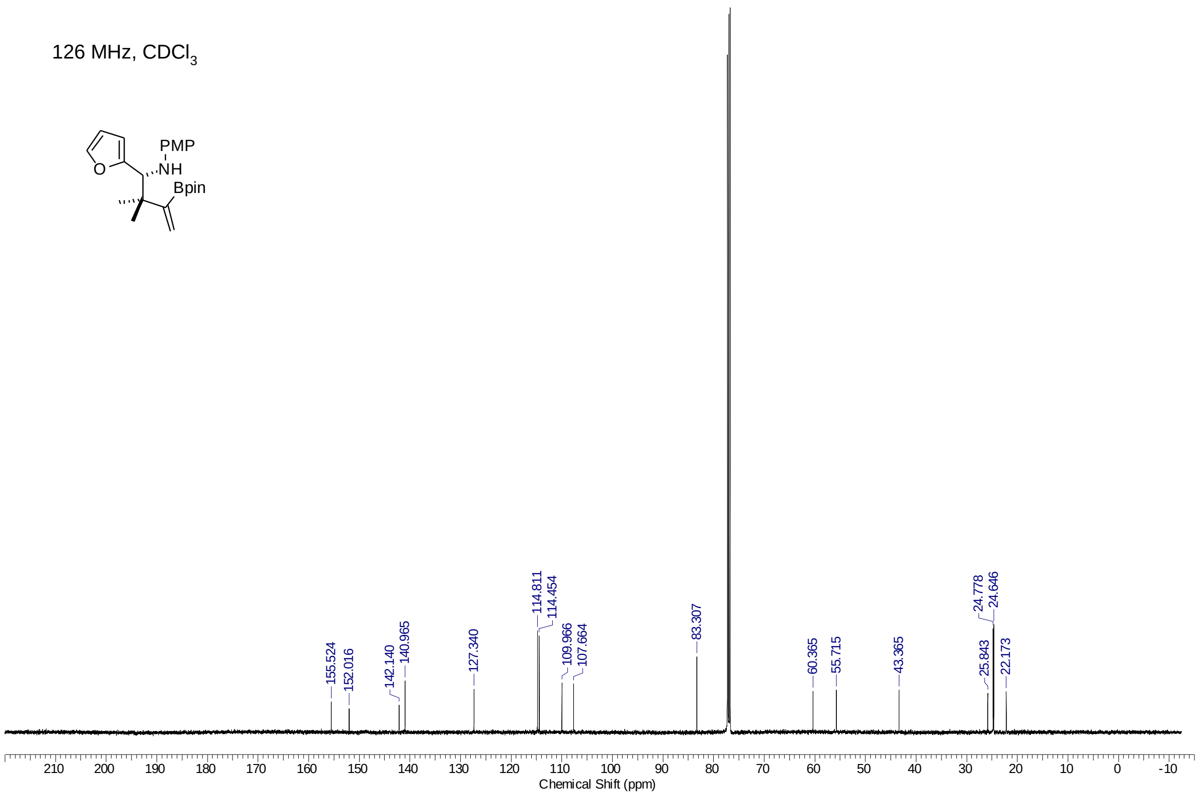

(*R*)-*N*-(2,2-Dimethyl-3-(4,4,5,5-tetramethyl-1,3,2-dioxaborolan-2-yl)-1-(thiophen-2-yl)but-3-en-1-yl)-4-methoxyaniline (3w)

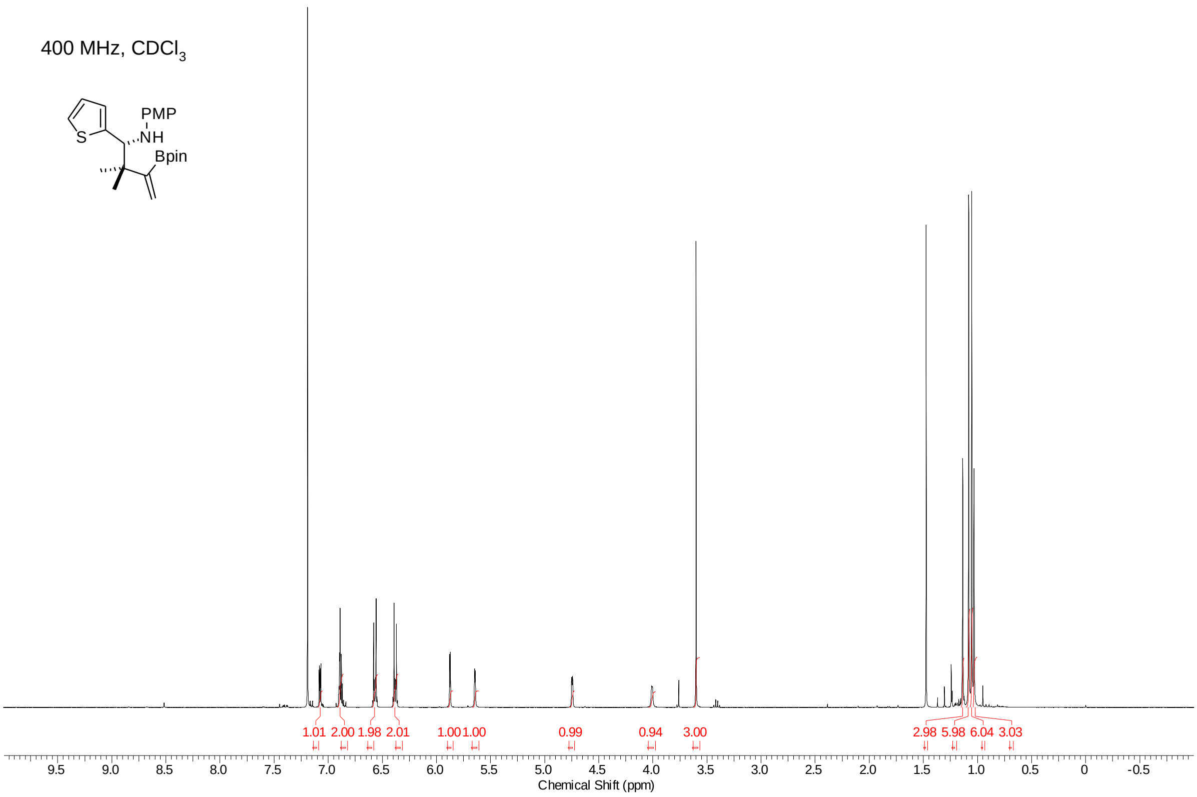

(*R*)-*N*-(2,2-Dimethyl-3-(4,4,5,5-tetramethyl-1,3,2-dioxaborolan-2-yl)-1-(thiophen-2-yl)but-3-en-1-yl)-4-methoxyaniline (3w)

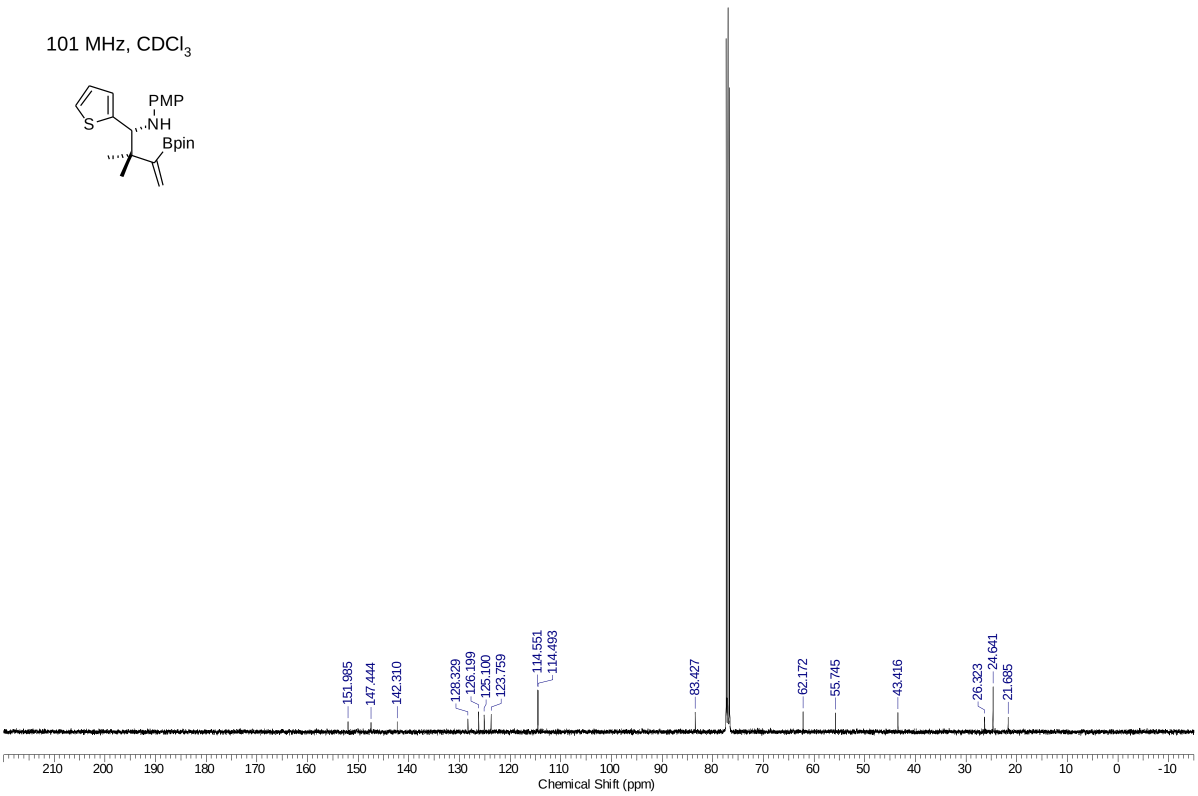

Methyl 4-(((1*R*,2*R*)-2-cyclohexyl-3-(4,4,5,5-tetramethyl-1,3,2-dioxaborolan-2-yl)-1-(*o*-tolyl)but-3-en-1-yl)amino)benzoate (3x)

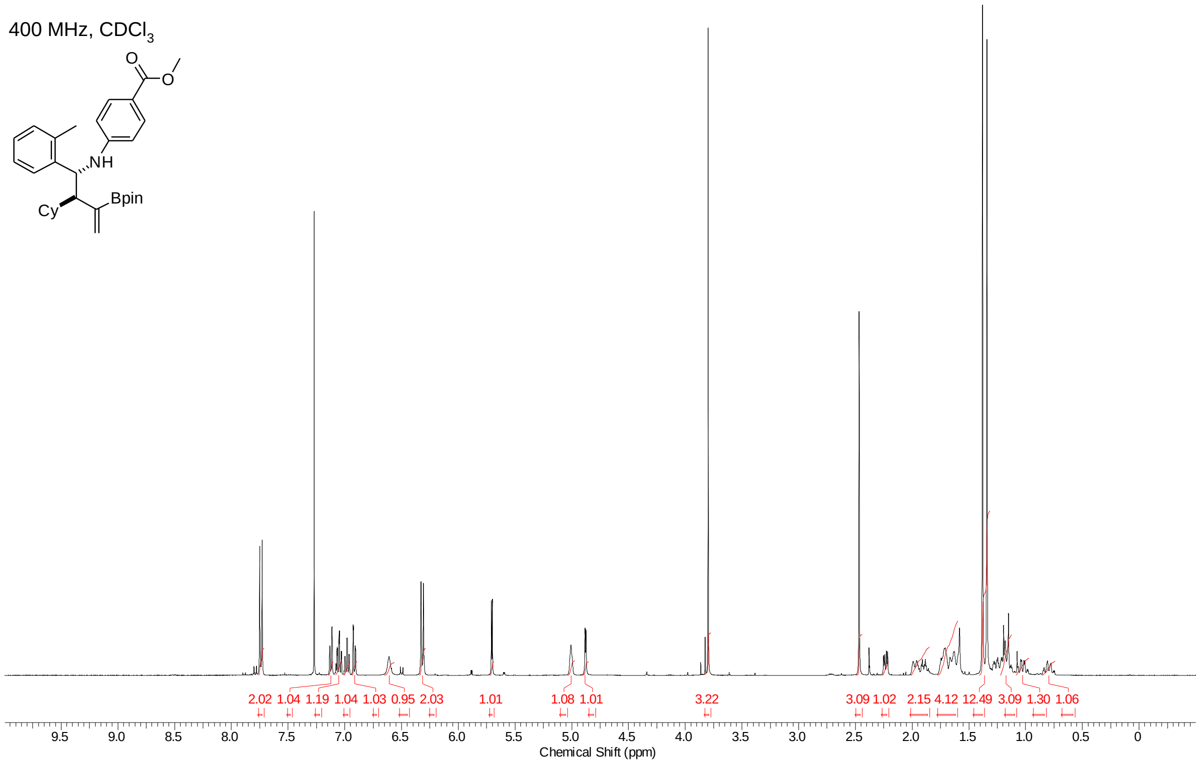

Methyl 4-(((1*R*,2*R*)-2-cyclohexyl-3-(4,4,5,5-tetramethyl-1,3,2-dioxaborolan-2-yl)-1-(*o*-tolyl)but-3-en-1-yl)amino)benzoate (3x)

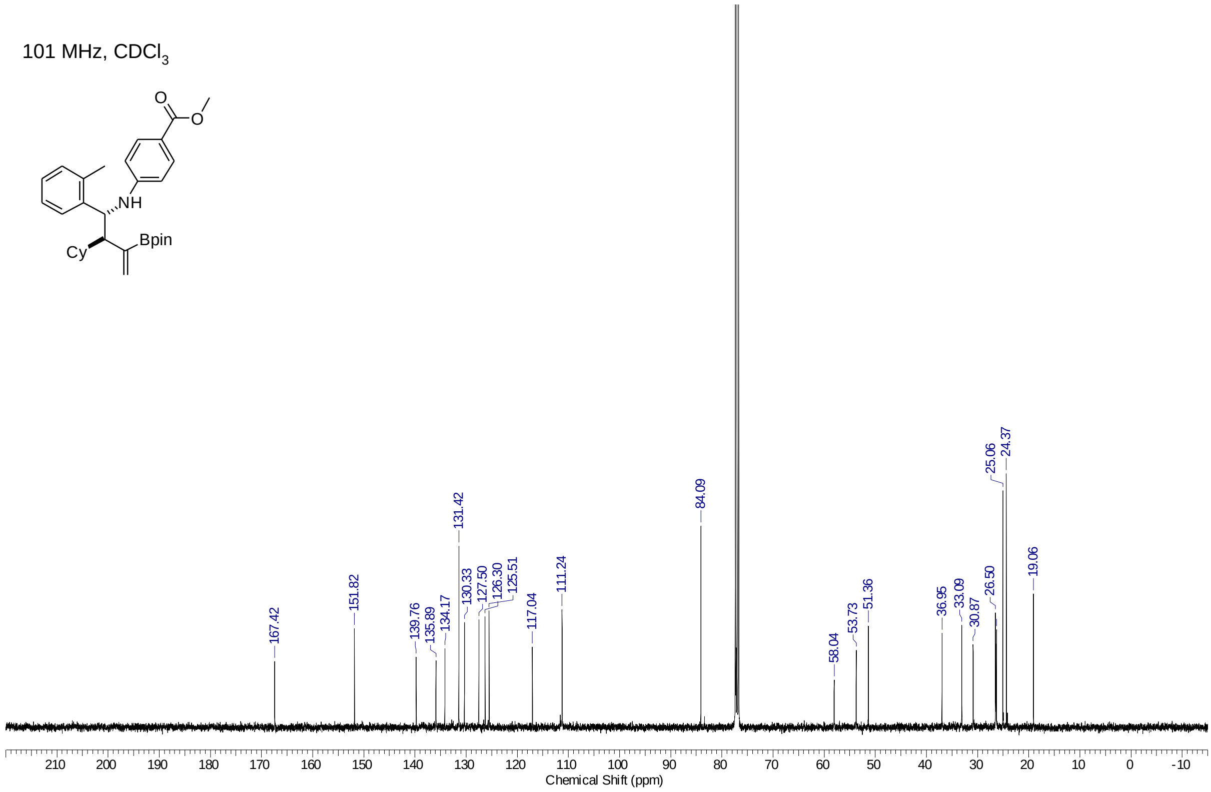

N-((1*R*,2*R*)-2-Cyclohexyl-3-(4,4,5,5-tetramethyl-1,3,2-dioxaborolan-2-yl)-1-(*o*-tolyl)but-3-en-1-yl)quinolin-5-amine (3y)

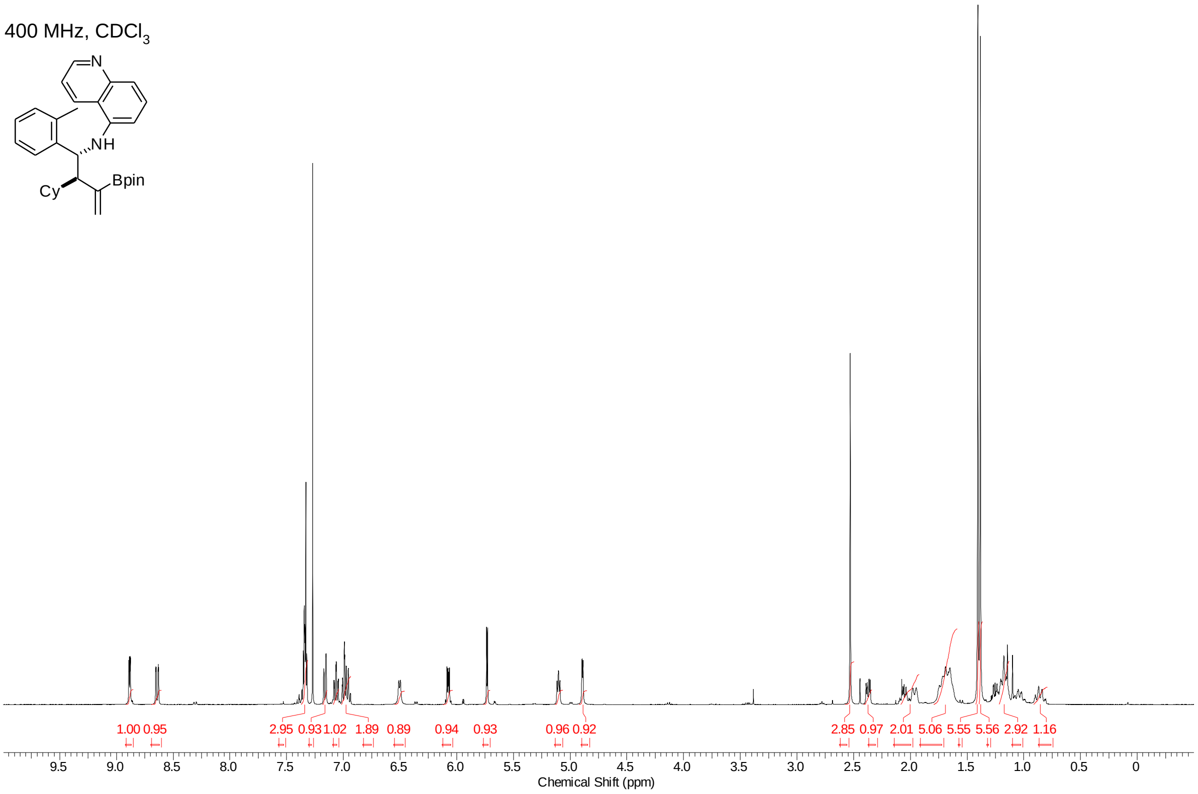

N-((1*R*,2*R*)-2-Cyclohexyl-3-(4,4,5,5-tetramethyl-1,3,2-dioxaborolan-2-yl)-1-(*o*-tolyl)but-3-en-1-yl)quinolin-5-amine (3y)

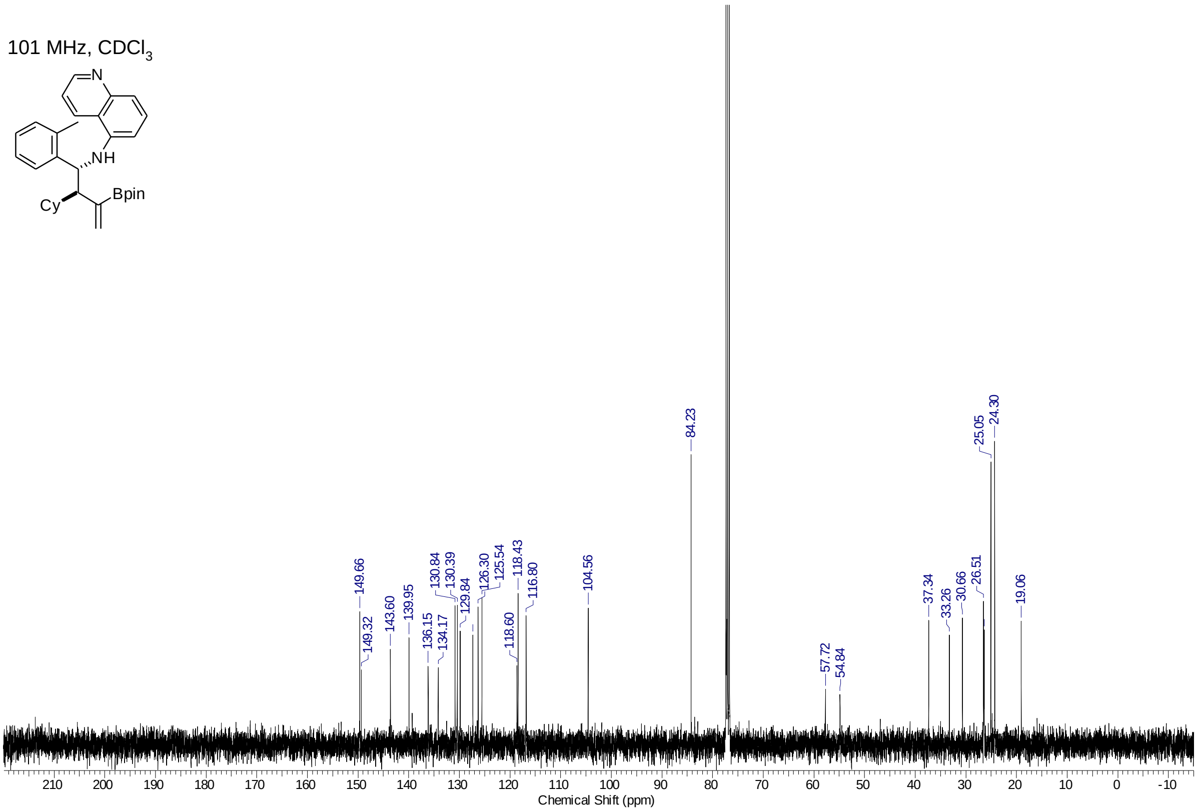

_N_-((1_R_,2_R_)-2-Cyclohexyl-3-(4,4,5,5-tetramethyl-1,3,2-dioxaborolan-2-yl)-1-(_o_-tolyl)but-3-en-1-yl)-4-morpholinoaniline (3z)

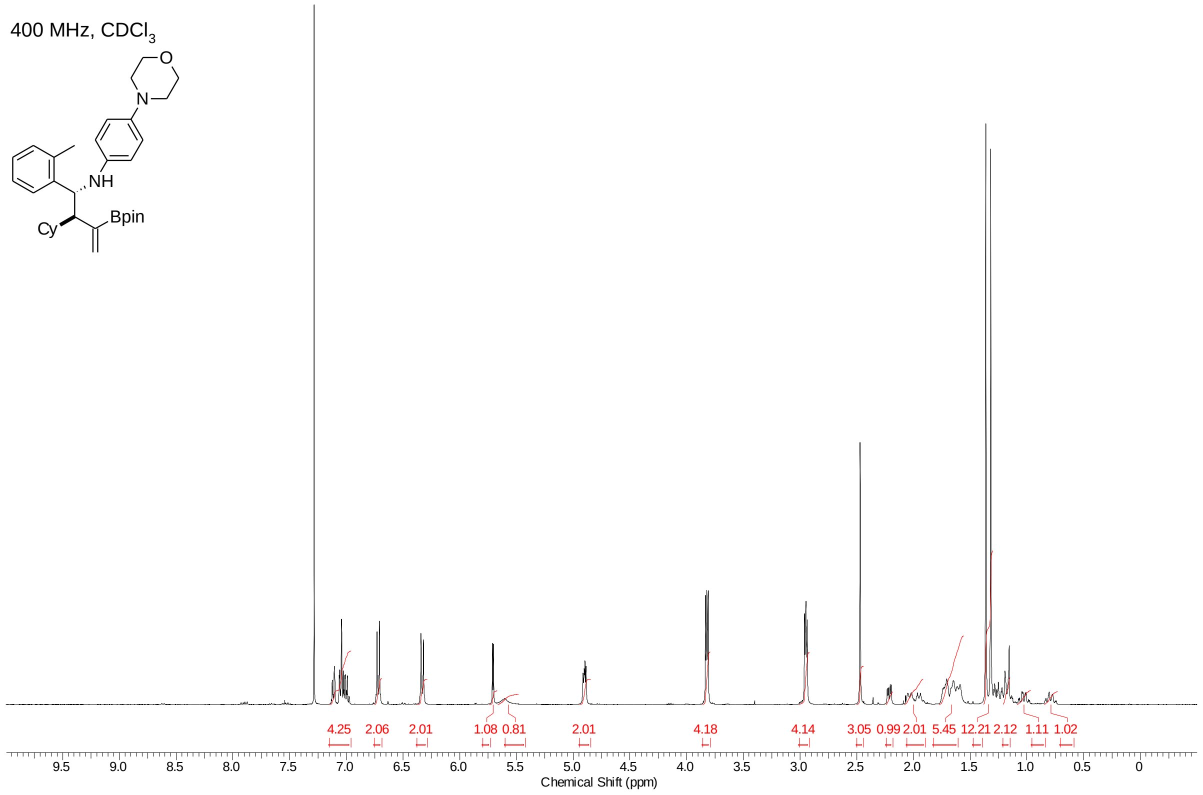

_N_-((1_R_,2_R_)-2-Cyclohexyl-3-(4,4,5,5-tetramethyl-1,3,2-dioxaborolan-2-yl)-1-(_o_-tolyl)but-3-en-1-yl)-4-morpholinoaniline (3z)

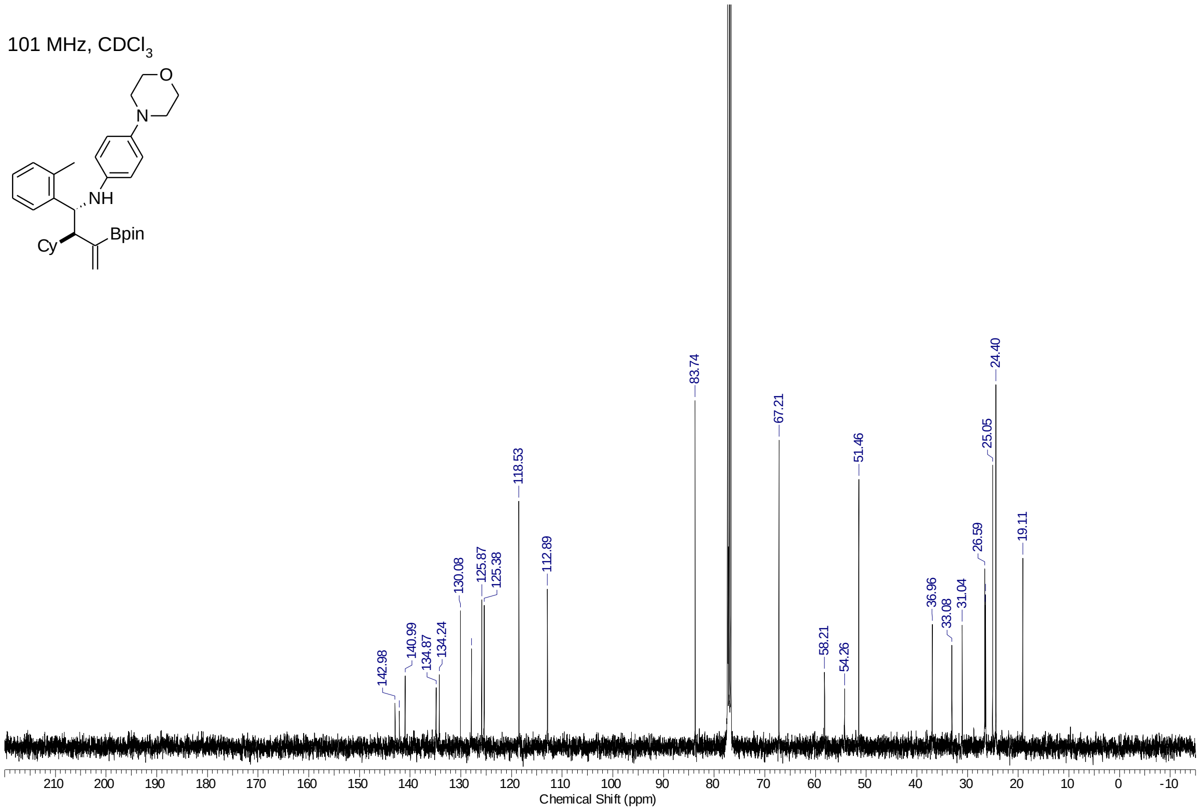

N-((1*R*,2*R*)-2-Cyclohexyl-3-(4,4,5,5-tetramethyl-1,3,2-dioxaborolan-2-yl)-1-(*o*-tolyl)but-3-en-1-yl)-4-(4,4,5,5-tetramethyl-1,3,2-dioxaborolan-2-yl)aniline (3aa)

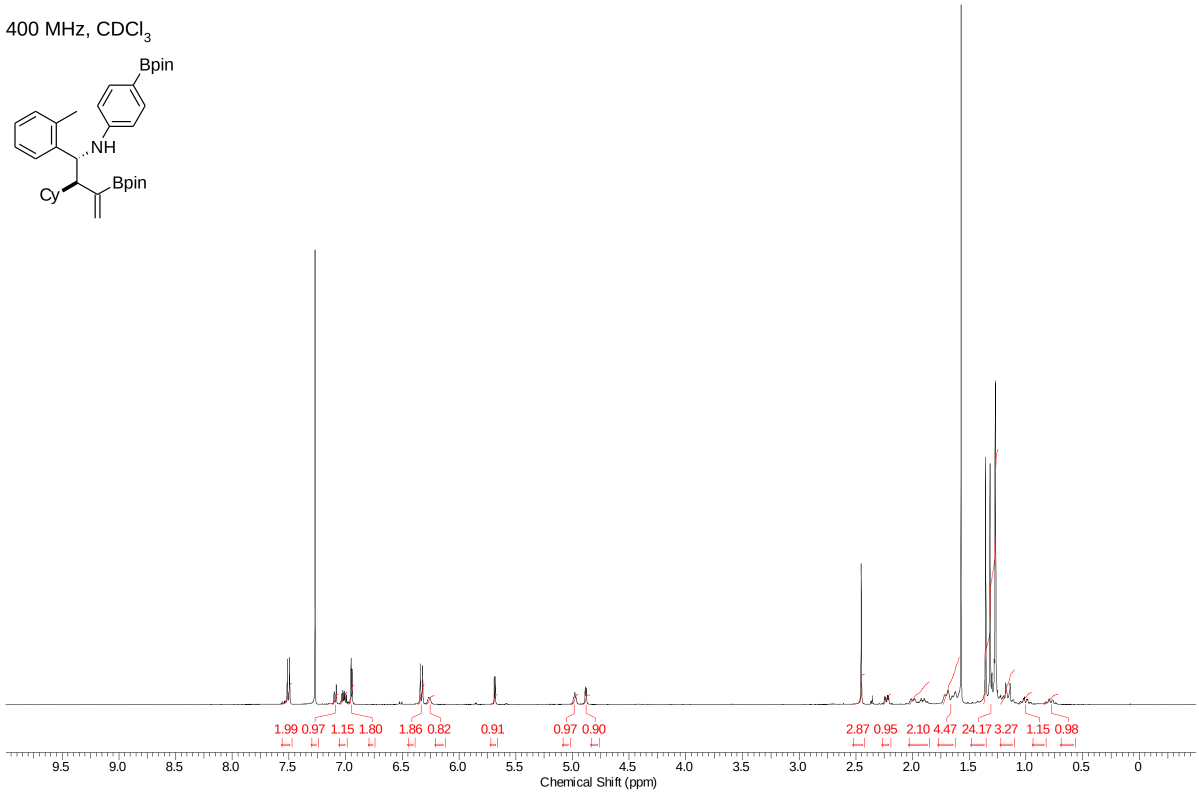

N-((1*R*,2*R*)-2-Cyclohexyl-3-(4,4,5,5-tetramethyl-1,3,2-dioxaborolan-2-yl)-1-(*o*-tolyl)but-3-en-1-yl)-4-(4,4,5,5-tetramethyl-1,3,2-dioxaborolan-2-yl)aniline (3aa)

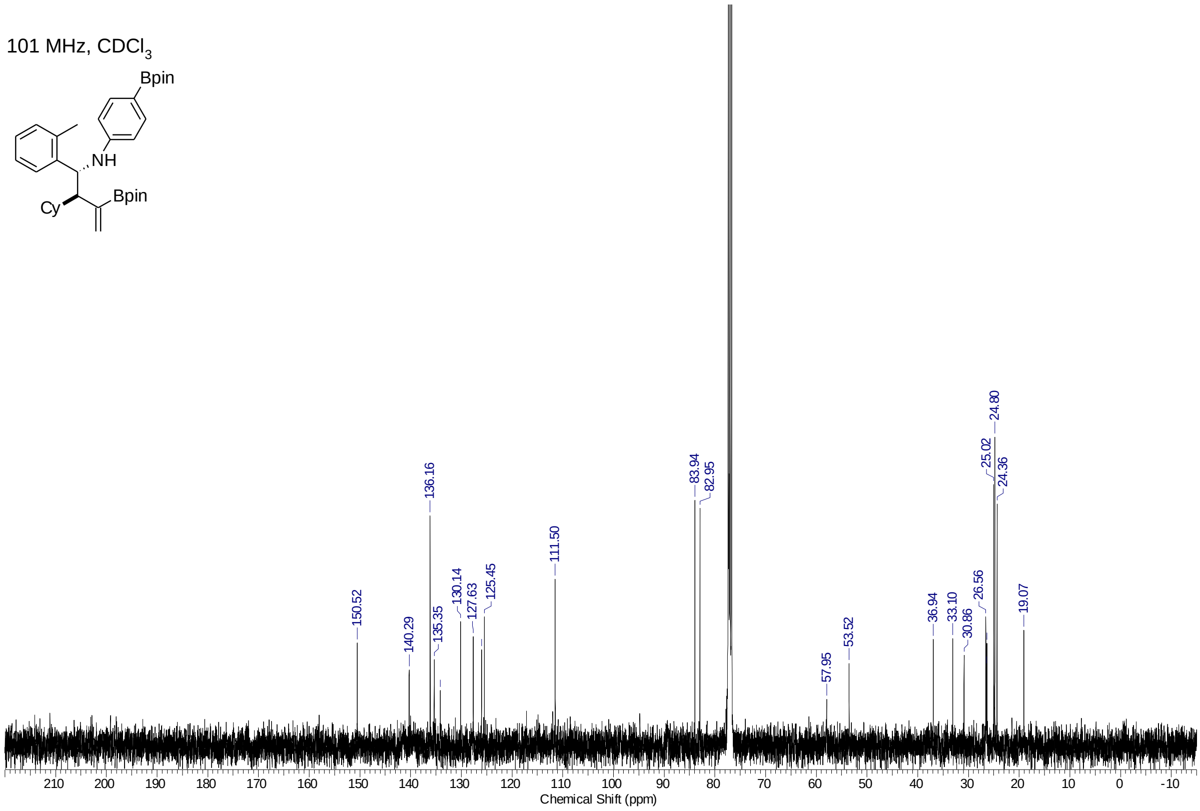

2-(Diethylamino)ethyl 4-(((1*R*,2*R*)-2-cyclohexyl-3-(4,4,5,5-tetramethyl-1,3,2-dioxaborolan-2-yl)-1-(*o*-tolyl)but-3-en-1-yl)amino)benzoate (3ab)

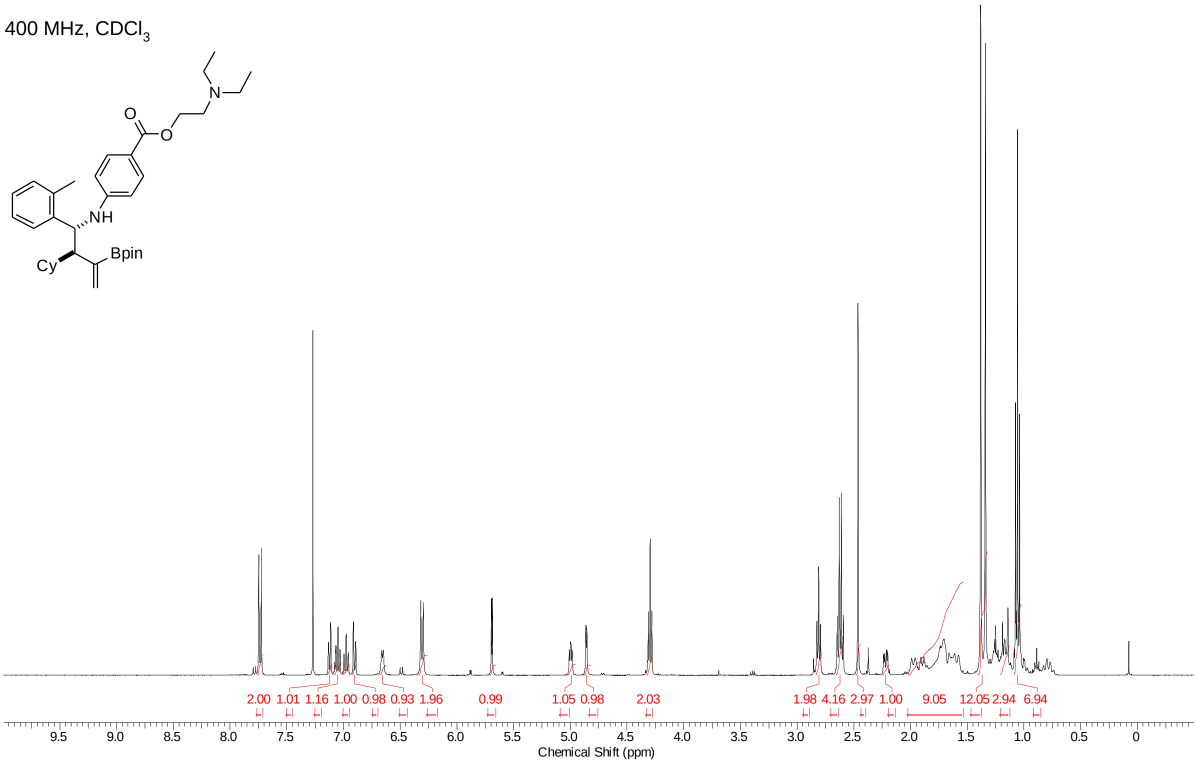

2-(Diethylamino)ethyl 4-(((1*R*,2*R*)-2-cyclohexyl-3-(4,4,5,5-tetramethyl-1,3,2-dioxaborolan-2-yl)-1-(*o*-tolyl)but-3-en-1-yl)amino)benzoate (3ab)

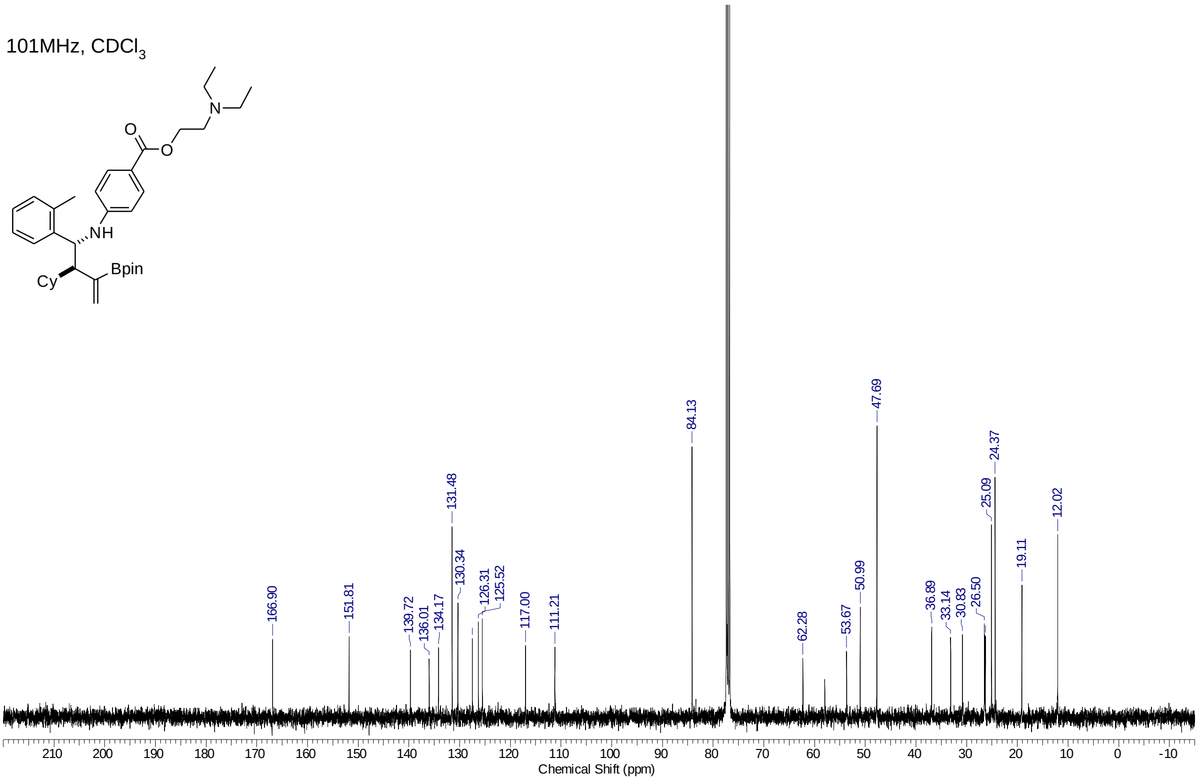

(3*R*,4*S*)-3-Cyclohexyl-4-((4-methoxyphenyl)amino)-4-(*o*-tolyl)butan-2-one (7)

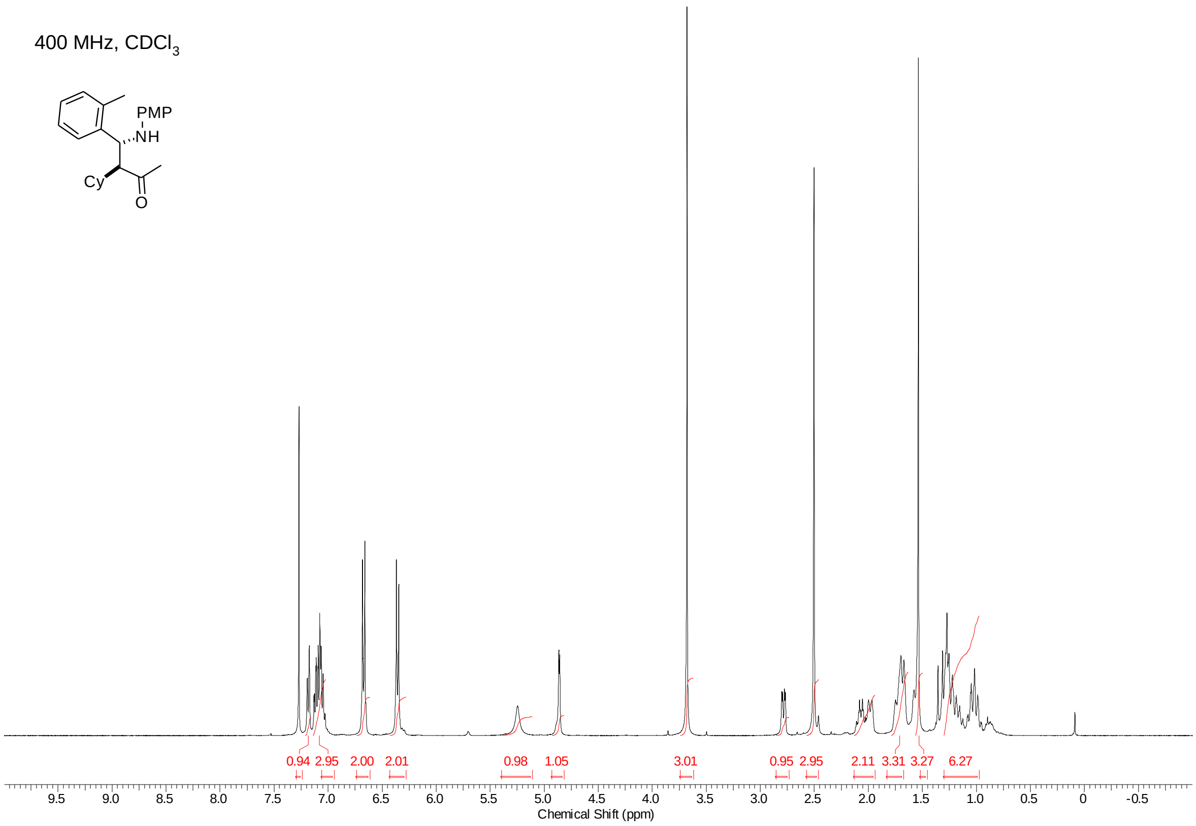

(3*R*,4*S*)-3-Cyclohexyl-4-((4-methoxyphenyl)amino)-4-(*o*-tolyl)butan-2-one (7)

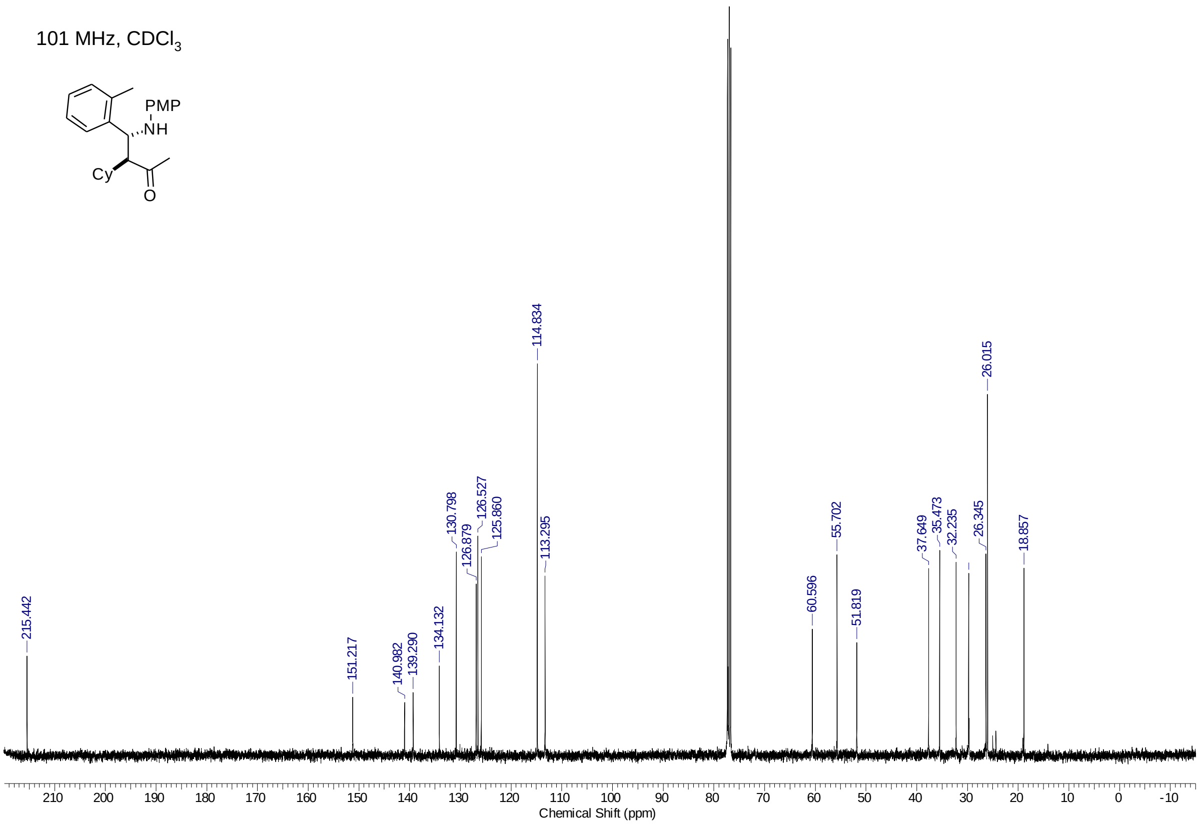

N-((1*R*,2*S*,3*S*)-2-Cyclohexyl-3-(4,4,5,5-tetramethyl-1,3,2-dioxaborolan-2-yl)-1-(*o*-tolyl)butyl)-4-methoxyaniline (8)

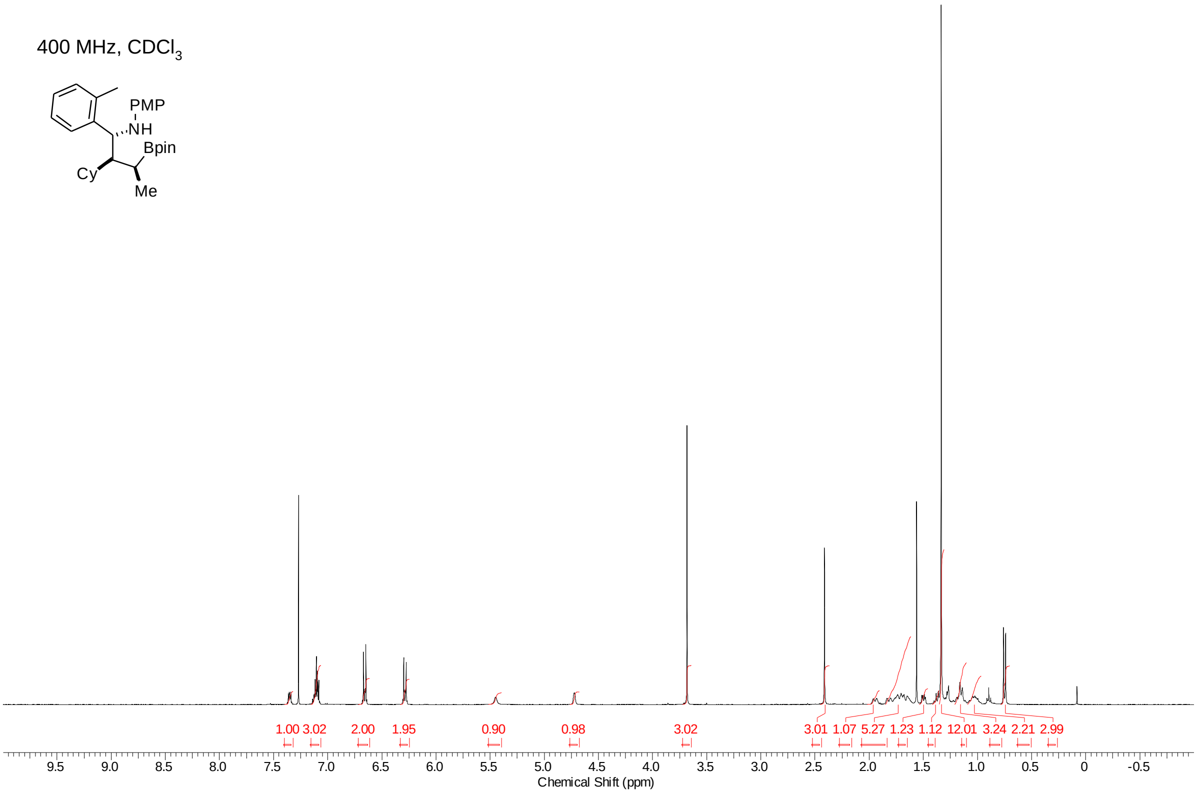

N-((1*R*,2*S*,3*S*)-2-Cyclohexyl-3-(4,4,5,5-tetramethyl-1,3,2-dioxaborolan-2-yl)-1-(*o*-tolyl)butyl)-4-methoxyaniline (8)

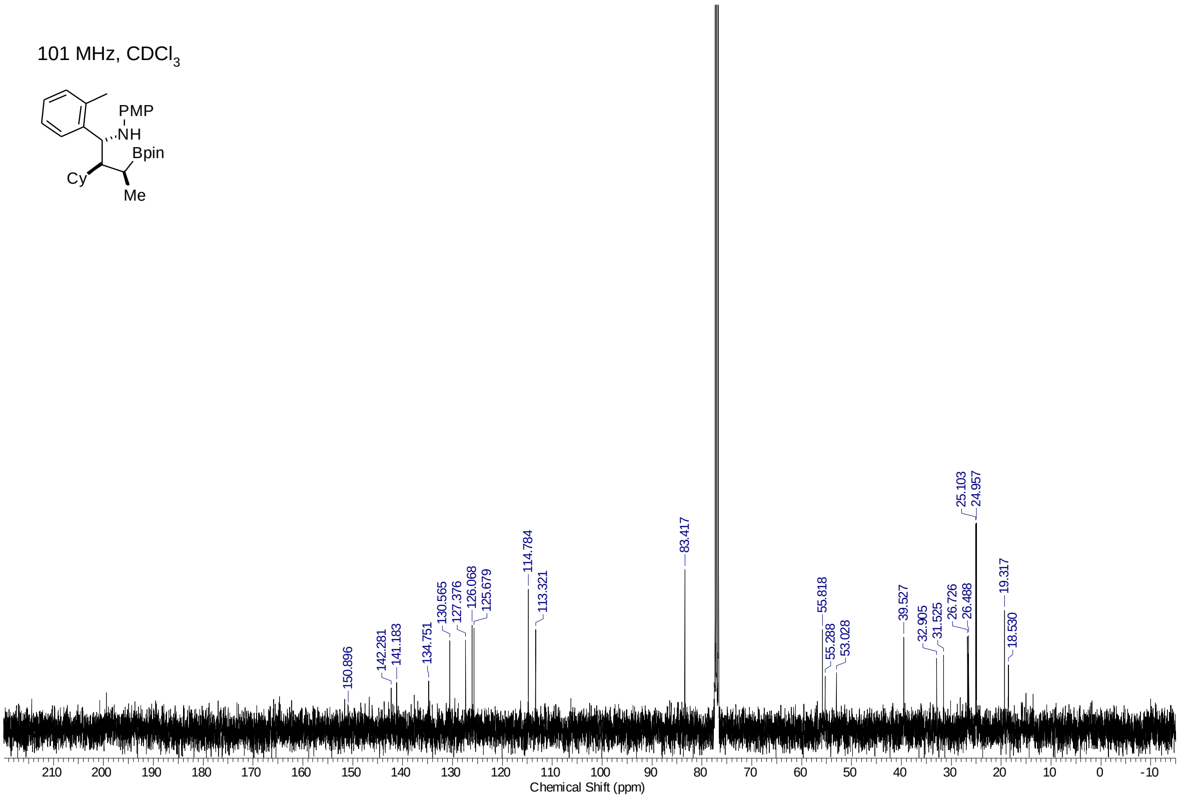

X-ray structure:

(*R*)-*N*-(2,2-Dimethyl-3-(4,4,5,5-tetramethyl-1,3,2-dioxaborolan-2-yl)-1-(thiophen-2-yl)but-3-en-1-yl)-4-methoxyaniline (3w)

CCDC 1489264

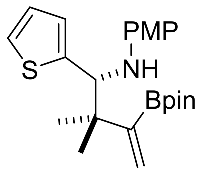

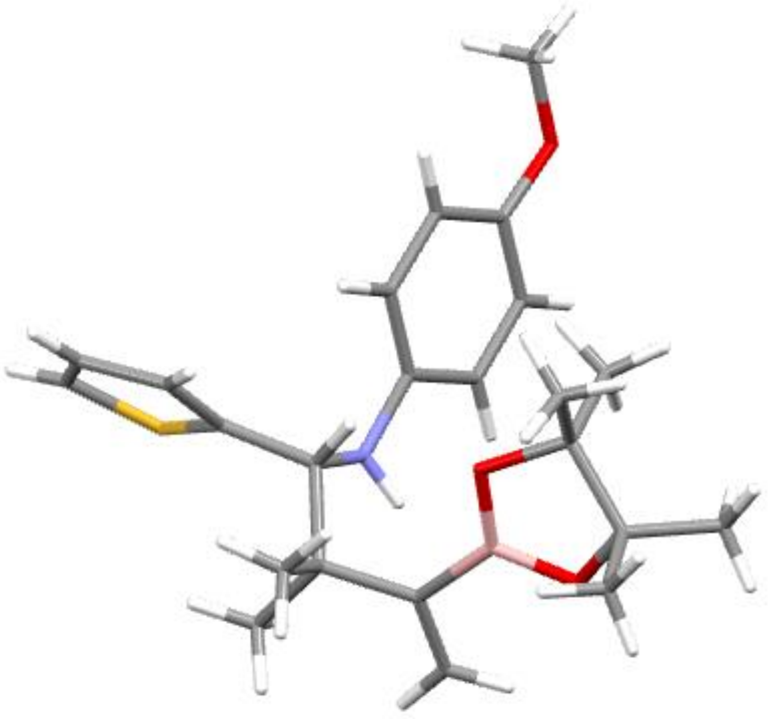

Supplement: Supplementary file 1 — Supplementary [file ANIE-55-11912-s001.pdf]
